# Completing the Spectral Mosaic of Chloromethane by Adding the CHD_2_Cl Missing Piece Through the Interplay of Rotational/Vibrational Spectroscopy and Quantum Chemical Calculations

**DOI:** 10.3390/molecules30071604

**Published:** 2025-04-03

**Authors:** Mattia Melosso, Paolo Stoppa, Daniela Alvarado-Jiménez, Filippo Tamassia, Carlotta Sapienza, Luca Bizzocchi, Luca Dore, Cristina Puzzarini, Andrea Pietropolli Charmet, Nicola Tasinato

**Affiliations:** 1 Dipartimento di Chimica “Giacomo Ciamician”, Università di Bologna, Via F. Selmi 2, 40126 Bologna, Italy; mattia.melosso2@unibo.it (M.M.); carlotta.sapienza@studio.unibo.it (C.S.); luca.bizzocchi@unibo.it (L.B.); luca.dore@unibo.it (L.D.); 2 Dipartimento di Scienze Molecolari e Nanosistemi, Università Ca’ Foscari Venezia, Via Torino 155, 30172 Venezia Mestre, Italy; stoppa@unive.it; 3 Scuola Normale Superiore di Pisa, Piazza dei Cavalieri 7, 56126 Pisa, Italy; daniela.alvaradojimenez@sns.it; 4 IUSS Pavia, Piazza della Vittoria 15, 27100 Pavia, Italy; 5 Dipartimento di Chimica Industriale “Toso Montanari”, Università di Bologna, Via Gobetti 85, 40129 Bologna, Italy; filippo.tamassia@unibo.it

**Keywords:** chlorocarbons, rotational spectroscopy, vibrational spectroscopy, equilibrium structure, quantum chemistry, climate metrics

## Abstract

Chloromethane (CH_3_Cl) is a key chlorinated organic compound not only in atmospheric chemistry, but also in the field of molecular astrophysics and a possible biosignature in exoplanetary atmospheres. While the spectroscopic characterization of the main isotopic species has been addressed in great detail, that of its isotopologues remains incomplete. This work aims at filling this gap by focusing on the bideuterated species, CHD_2_Cl, and exploiting both rotational and vibrational spectroscopy in combination with state-of-the-art quantum-chemical (QC) calculations. First, the rotational spectrum of CHD_2_Cl has been measured in the millimeter-wave domain, allowing the accurate determination of several spectroscopic constants for four isotopologues, namely ^12^CHD_2_^35^Cl, ^12^CHD_2_^37^Cl, ^13^CHD_2_^35^Cl, and ^13^CHD_2_^37^Cl. The newly determined rotational constants have been used to refine the semi-experimental equilibrium structure of chloromethane. Secondly, the vibrational analysis, supported by high-level QC predictions of vibrational energies, has been conducted in the 500–6200 cm^−1^ infrared (IR) region, enabling the identification of more than 30 bands including fundamental, overtone, and combination transitions. Finally, chloromethane’s radiative efficiency has been simulated using the QC IR absorption cross-sections, and the effects of isotopologue distribution on the predicted radiative properties have been investigated. All these findings greatly improve the comprehension of the spectroscopic properties of bideuterated chloromethane isotopologues, and of chloromethane in general, and facilitate future terrestrial and extraterrestrial studies.

## 1. Introduction

Among the Earth’s atmospheric components, chlorinated organic compounds are particularly relevant because they act both as greenhouse gases and sources of reactive chlorine responsible for stratospheric ozone depletion (for some recent literature, see refs. [1,2,3] and references therein). Therefore, their detection and monitoring are extremely relevant. Data on their atmospheric abundances can also be used for assessing the efficiency of climate mitigation policies [4]. The detection and quantification of the different gaseous components can be performed using specialized software packages (e.g., see refs. [5,6]) that exploit accurate data obtained from line-shape and ro-vibrational analyses [7], the latter being greatly assisted by quantum-chemical (QC) calculations. Besides providing reliable predictions of several spectroscopic parameters for halocarbons (see, for example, refs. [8,9] and references therein), QC methods also allow the computation of their radiative efficiency (RE) and other relevant environmental metrics, such as the Global Warming Potential (GWP) [10,11]. Recently, they have also been used to train machine-learning models [12]. Due to the adverse effects above mentioned, the use and production of many halocarbons are currently banned by the Montreal Protocol (MP) and its amendments, but chlorinated methanes like chloromethane (methyl chloride, R40, CH_3_Cl), dichloromethane (methylene chloride, R30, CH_2_Cl_2_) and trichloromethane (chloroform, R20, CHCl_3_) are not yet included. Chloromethane is considered to be the most predominant source of reactive chlorine in the atmosphere. Characterized by a global mixing ratio of about 553(5) pptv, with a global emission estimated in the 4–5 Tg year^−1^ range and an atmospheric lifetime of 0.9 years [13], it contributes to 17% of the tropospheric load [14]. The major natural sources of CH_3_Cl are tropical and subtropical vegetation [15,16,17], oceans [18,19], soil and seawater [20], tropical wood-rot fungi [21], plants of salt marshes [22], leaf litter [23] and biomass burning [24,25]. Among the most important anthropogenic sources, there are the combustion of fossil fuels, food production [26], cattle [27], human breath [28], and the iron and steel industry [29].

In addition to its relevance for the Earth atmosphere, chloromethane, due to its high biological specificity and low false-positive potential, has also been proposed as “capstone” biosignature in the investigation of the atmospheres of Earth-like exoplanets [30,31,32,33] and, in particular, super-Earth planets (see refs. [34,35]).

Recently, its detectability using the James Webb Space Telescope has also been assessed [36]. Besides, the Rosetta mission led to the discovery of signals due to both the chlorine isotopologues of CH_3_Cl in the protostar IRAS 16293-2422 and in the coma of the 67P/Churyumov-Gerasimenko comet [37], thus pointing out that the chemistry of chlorinated organic compounds should be properly taken into account also in planet-forming regions. It should be highlighted that, in the same protostar region, for several molecules (such as CH_3_CN, NH_2_, H_2_O), radioastronomical observations allowed the detection not only of the main isotopic species but also of the corresponding deuterated and bideuterated derivatives, such as CH_2_DCN/CHD_2_CN [38], NHD/ND_2_ [39,40], and HDO/D_2_O [41]. These discoveries suggest that also mono- and bideuterated isotopologues of chloromethane (CH_2_DCl and CHD_2_Cl) might be present as well, but their detection requires accurate spectroscopic data, which are also needed for the determination of the corresponding ^35/37^Cl isotopic ratio [42].

Given its aforementioned relevance in Earth’s atmospheric processes and its role as biomarker in astrochemistry, chloromethane has been the subject of many experimental and theoretical spectroscopic investigations. Several studies focused on line-shape and line-position analyses (see, for example, refs. [43,44,45,46,47,48,49,50,51,52,53]), others concerned its dipole moment surface and vibrational energies evaluated by means of *ab initio* methods [54,55], while microwave measurements provided accurate values for ground-state rotational parameters [56,57]. In comparison with the large amount of data available for the main isotopic species of chloromethane, so far the mono- and bideuterated isotopologues have received less attention. This prompted us to focus our effort on their spectroscopic characterization in order to help and guide their search in the interstellar medium. We started by studying CH_2_DCl. For both its chlorine isotopologues, we obtained precise rest frequencies at mm-wavelengths and accurate ground-state spectroscopic parameters [58]. Then, we proceeded by carrying out the ro-vibrational analysis of the bands falling in the 15.4–8 μm region [59,60]. Finally, we provided accurate *ab initio* predictions for the spectroscopic parameters of its isotopologues (^12^CH_2_D^35^Cl, ^13^CH_2_D^35^Cl, ^12^CH_2_D^37^Cl, ^13^CH_2_D^37^Cl) and an analysis of their rotational spectra [61].

The present work further extends the knowledge of the spectroscopic properties of chlorometane by focusing on CHD_2_Cl, its bideuterated isotopologue. The detailed spectroscopic characterization of this species starts with the analysis of its pure rotational spectrum from which accurate ground-state spectroscopic parameters are derived for the ^35/37^Cl and ^12/13^C isotopologues. The rotational constants obtained in this way are then employed, together with those available in the literature for a number of isotopically substituted species, to refine the equilibrium structure determination of chloromethane through the semi-experimental (SE) method [62,63]. The resulting equilibrium geometry is then compared with that evaluated using a recently proposed QC composite scheme rooted in the coupled-cluster (CC) theory. High-level QC computations are also carried out to predict CHD_2_Cl vibrational properties with the aim of assisting the analysis of its infrared (IR) spectrum, which is experimentally recorded in the 500–6200 cm^−1^ region. Finally, anharmonic QC simulations of the IR absorption cross sections are used to obtain the RE of the bideuterated species. This is then compared with those calculated for the parent and the most abundant isotopologues to assess the effect of isotopic substitution on the radiative properties of R40.

## 2. Results and Discussion

Bideuterated chlorometane is a near-prolate asymmetric top rotor, the asymmetry parameter κ being −0.993. The molecule belongs to the $C_{S}$ symmetry point group, with the symmetry plane identified by the *a*- and *c*-principal axes, while the *b*-axis is perpendicular to it. It possesses nine normal modes of vibration that, in terms of symmetry species, can be classified as $6A^{\prime }⨁3A^{\prime \prime }$, with $A^{\prime }$ vibrations giving rise to hybrid *a*/*c* bands, and vibrations of $A^{\prime \prime }$ symmetry producing *b*-type absorptions. The corresponding harmonic frequencies predicted at fc-CCSD(T)/V5Z-aV(5+*d*)Z level of theory are listed in Table 1 (for details about computations and acronyms the reader is referred to Section 3). In the next subsections, the CHD_2_Cl pure rotational spectra are first analyzed, providing a full set of spectroscopic parameters not only for both the ^35/37^Cl isotopologues but also for the corresponding ^13^C species. The rotational constants obtained in this way were then used together with literature data to refine the equilibrium geometry through the SE method. The attention is then moved to the interpretation of the IR spectrum, while in the last subsection, the RE of the molecule is evaluated from the simulated IR absorption cross section spectrum.

### 2.1. Rotational Analysis

The rotational spectrum of ^12^CHD_2_^35^Cl and ^12^CHD_2_^37^Cl was studied at low frequency—below 40 GHz—in the early 1950’s and in the late 1970’s [64,65,66]. The ^13^CHD_2_^35^Cl and ^13^CHD_2_^37^Cl isotopologues, instead, were not studied so far. With the aim of (i) enabling astronomical searches of these species at higher frequencies and (ii) enlarging the dataset for improving the SE equilibrium structure determination, we performed highly accurate measurements of their rotational transitions in the millimeter-wave domain.

Previously determined spectroscopic parameters [64,65,66] have been used to predict the spectra of ^12^CHD_2_^35^Cl and ^12^CHD_2_^37^Cl above 80 GHz. In this way, the *a*-type features between 80 and 330 GHz were accurately pinpointed and recorded for both species: they include transitions between levels with maximum *J* and $K_{a}$ values of 14 and 13, respectively. The newly recorded transitions (about 150 for each species) have then been analyzed in combination with literature data, thus allowing for the determination of a more accurate and reliable set of spectroscopic constants. These are reported in Table 2 along with their theoretical counterpart and the previous determination from ref. [66], the parameters being expressed in terms of an *A*-reduced Watson-type Hamiltonian in the $I^{r}$ representation.

Inspection of Table 2 reveals an excellent agreement between theory and experiment: rotational and quartic centrifugal distortion constants show a mean absolute deviation of 0.001% and 3.3%, respectively. A good agreement is also observed for the chlorine quadrupole coupling constants, $\chi_{aa}$ and $\chi_{bb}$, whose experimental determination is reported here for the first time. The comparison between the newly derived parameters and those previously determined [66] points out a great improvement in terms of the accuracy achieved for the spectroscopic constants, the uncertainty on the rotational and the centrifugal distortion constants being reduced by at least one order of magnitude. As far as the sextic centrifugal distortion terms are concerned, the use of theoretical values for the full set of constants made it unnecessary to float any of them in the final analysis, although their inclusion is crucial for correctly reproducing the observed transition frequencies. A last remark on the dataset used in the least-squares fitting procedure is deserved. Initially, the line list used in the analysis contained all the transitions coming from four different sources, which are refs. [64,65,66] and this work. However, during the final refinement of the spectroscopic parameters we noticed that (i) the lines from refs. [64,65] systematically deviate from the predicted positions and were therefore excluded throughout, and (ii) few lines from ref. [66] (3 for ^12^CHD_2_^35^Cl and 5 for ^12^CHD_2_^37^Cl) exhibit deviations more than three times greater than their declared uncertainty and were consequently excluded from the fit as well. The fit residuals of our transitions is in the order of 20 kHz, in line with their expected measurement accuracy.

**Figure 1:**
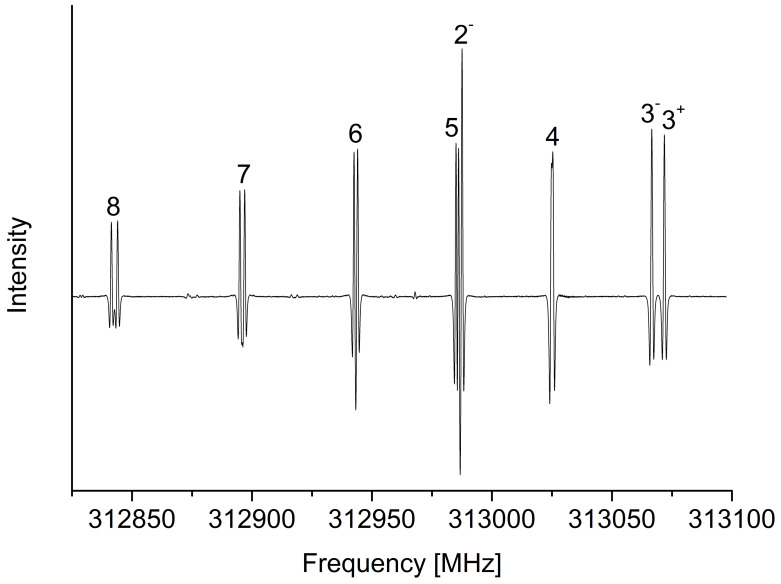
Portion of the J=14←13 pure rotational transition of ^13^CHD_2_^35^Cl between 312,825 and 313,100 MHz. The number above each spectral line represents the $K_{a}$ value of the corresponding transition (the + and − superscripts indicate whether $K_{a}+K_{c}$ is equal to *J* or J+1, respectively).

For the measurements of the ^13^CHD_2_^35^Cl and ^13^CHD_2_^37^Cl species, spectral predictions have been prepared using our set of computed rotational, centrifugal distortion, and chlorine quadrupole coupling constants. Given the low natural abundance of these species relative to the ^12^C-isotopologues, accurate predictions were essential for their correct identification in the spectrum, where lines associated with vibrationally excited states below 1000 cm^−1^ are expected to be more intense at room temperature. In line with what had been previously observed for the ^13^CH_2_D^35^Cl and ^13^CH_2_D^37^Cl species [61], *a*-type transitions were typically found within a few MHz from their predicted positions. Our measurements include *ca.* 200 distinct lines for each species, the maximum values reached for *J* and $K_{a}$ being 14 and 12, respectively. As an example, the ^13^CHD_2_^35^Cl spectrum in the region between 312.8 and 313.1 GHz is reported in Figure 1, where the typical $K_{a}$-structure associated to the *a*-type spectrum of nearly-prolate asymmetric rotors is visible. In particular, Figure 1 shows several $K_{a}$ components (between 2 and 8) of the J=14←13 transitions, where the asymmetry splitting is unresolved for $K_{a}>3$, while the chlorine hyperfine structure becomes partially resolved at $K_{a}\geq 5$. Fitting these transitions to an *A*-reduced Watson-type Hamiltonian allowed the first determination of the rotational constants, almost all the quartic centrifugal distortion terms, and the diagonal elements of the chlorine quadrupole tensor. The results are listed in Table 3.

The agreement between the experimental and theoretical constants is again excellent, similarly to what observed for the ^12^CHD_2_^35^Cl/^12^CHD_2_^37^Cl species and for the mono-deuterated isotopologues [61]. The accuracy achieved on the experimental spectroscopic constants is generally very good, the only exception being the *A* constants. This is due to the fact that only *a*-type transitions could be measured because of the low-abundance of ^13^CHD_2_^35^Cl and ^13^CHD_2_^37^Cl. The root-mean-square error of the transitions measured for the ^13^C isotopologues is around 35 kHz, in accordance with the small signal-to-noise ratio of their spectra. Outputs of the non-linear least square fitting of the measured transitions can be found as supplementary materials.

### 2.2. Semi-Experimental Equilibrium Structure

The vibrational ground-state rotational constants of ^12^CHD_2_^35^Cl, ^12^CHD_2_^37^Cl, ^13^CHD_2_^35^Cl and ^13^CHD_2_^37^Cl, determined in the previous section, have been used to refine the SE equilibrium structure of chloromethane together with the data already available in the literature for ^12^CH_3_^35^Cl [57], ^12^CH_3_^37^Cl [57], ^13^CH_3_^35^Cl [67], ^13^CH_3_^37^Cl [68], ^12^CD_3_^35^Cl [69], ^12^CD_3_^37^Cl [69], ^12^CH_2_D^35^Cl [61], ^12^CH_2_D^37^Cl [61], ^13^CH_2_D^35^Cl [61] and ^13^CH_2_D^37^Cl [61]. The rotational constants of this set of isotopologues have been corrected for vibrational and electronic contributions calculated as described in Section 2 and listed in Table 4, thus obtaining the SE equilibrium rotational constants used for the structural refinement. In addition, the SE equilibrium structure has also been determined by using PW6B95-D3/aug-cc-pVTZ electronic corrections here computed together with CCSD(T)/VQZ-V(Q+*d*)Z vibrational corrections [61]. The obtained SE equilibrium geometries are detailed in Table 5, where they are also compared with theoretical estimates at different levels of theory. The SE equilibrium structure here determined by using CCSD(T) vibrational corrections coincides, within the quoted statistical uncertainties, with the most recent one reported in the literature [61]. This result was expected, as the present structural refinement relies on the same experimental rotational constants used in ref. [61] and the main difference lies in the use of the improved rotational constants here determined for the ^12/13^CHD_2_^35/37^Cl isotopologues. The negligible differences can be attributed to the inclusion of electronic contributions that, in any case, are at most on the order of 0.01% of the equilibrium rotational constant value. In passing, it is worth noticing that the use of rev-DSDPBEP86-D3/jun-cc-pV(T+*d*)Z vibrational corrections delivers comparable geometrical parameters as those obtained considering CCSD(T) vibrational contributions, with differences of 0.1 and 0.2 mÅ for the C−Cl and C−H bond lengths, respectively, and the same value for the H-C-Cl angle. The only price to be payed for the lower computational cost, is a deterioration of the statistical uncertainty on the retrieved parameters.

The SE equilibrium structures closely match theoretical predictions based on different approaches rooted in the coupled-cluster theory. Inspection of Table 5 reveals an excellent agreement between the SE equilibrium geometries and that denoted as “CCSD(T)/CBS + CV + fT + fQ + SR”, indeed showing deviations of about 0.02 mÅ and 0.1 mÅ for the C−Cl and C−H distances, respectively, and 0.01° for the HCCl angle. This latter theoretical structure was computed by adding the fT and fQ corrections as well as the DBOC and SR contributions evaluated in this work to the CCSD(T)/CBS+CV equilibrium geometry, which accounts for extrapolation to the CBS limit and core-valence (CV) correlation effects at the CCSD(T) level, reported in ref. [61]. A good agreement is also noted with the equilibrium structure reported by Owen et al. [55] (based on explicitly correlated CC calculations with extrapolation to the CBS limit and contributions due to inclusion of CV correlation, higher-order coupled cluster excitations, scalar relativistic effects, and DBOC), which shows deviations within 0.2 mÅ for bond lengths, while the angle essentially coincides with the SE equilibrium value. The equilibrium structure determined in this work according to the MEDIUM-like composite recipe, delivers results similar to the other theoretical estimates for the C−H distance and the ClCH angles; however, it presents a larger deviation, −0.9 mÅ, for the C−Cl length. A possible explanation might be traced back to an underestimation of the CBS limit for this parameter: indeed, by considering the first two terms on the rhs of Equation (3), a value of 1.7757 Å is obtained, while application of the CCSD(T)/CBS+CV gradient scheme [61] leads to 1.7768 Å. However, it should be noted that the MEDIUM recipe introduced in ref. Sahoo et al. [70] was set up and validated for a test set of eleven molecules containing only first-row atoms. Therefore, inclusion of an extra *d* function of the Cl atoms might improve the accuracy of the C− Cl equilibrium bond length.

### 2.3. Vibrational Assignment of Gas-Phase Infrared Spectrum

**Figure 2:**
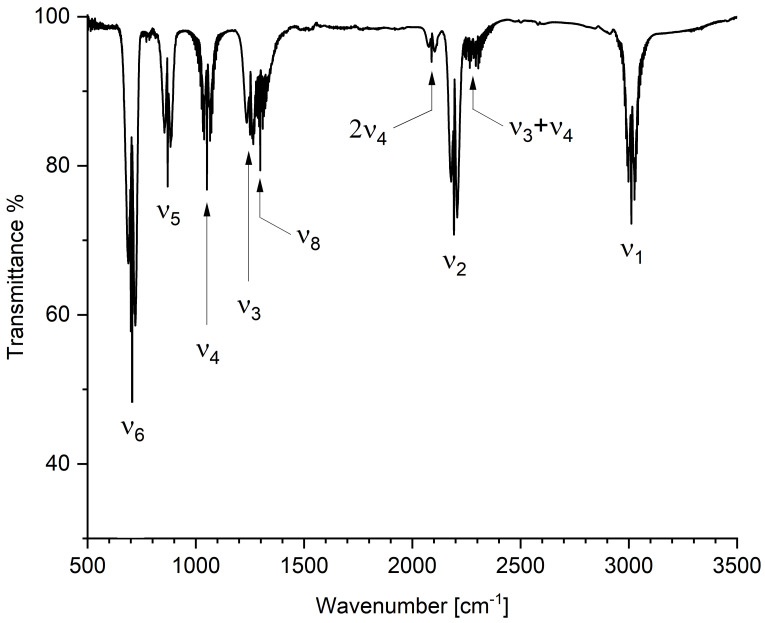
Gas-phase survey spectrum of CHD_2_Cl in the spectral region 500–3500 cm^−1^. Resolution = 1.0 cm^−1^, optical path length = 16.0 cm, room temperature, pressure = 2.0 kPa. Some relevant absorptions are labeled.

The vibrational analysis of CHD_2_Cl was carried out on the gas-phase infrared spectra recorded in the range 500–6200 cm^−1^. As first step, with the help of the predicted data listed in Table 6, all the fundamentals were assigned showing an excellent agreement between the experimental and computed data (mean absolute error equal to 1.1 cm^−1^). The values here determined are in good agreement (mean absolute deviation equal to 0.6 cm^−1^) with the previous data by Duncan et al. [71]. Once all the fundamentals were assigned, several weaker absorption features, mainly due to overtone and combination bands, were identified using the theoretical predictions, thus extending the vibrational assignments up to 6200 cm^−1^. The only interaction needed to properly assign the vibrational spectrum was the Fermi type I resonance occurring between $\nu_{2}$ and $2\nu_{4}$. This resonance was found not only for both ^12^CHD_2_^35^Cl and ^12^CHD_2_^37^Cl, but also for ^13^CHD_2_^35^Cl and ^13^CHD_2_^37^Cl species. A survey spectrum showing the relevant absorptions in the region up to 3500 cm^−1^ is reported in Figure 2.

The analysis of the medium-resolution IR spectra led to the assignment of all the fundamentals and of several overtone and combination bands. In Table 7, we report their experimental values together with the corresponding predictions. Notably, the overall agreement between experimental and computed data is remarkable, the mean absolute error over the whole 500–6200 cm^−1^ spectral range being equal to 3.9 cm^−1^.

#### 2.3.1. Vibrational Analysis of the 500–1500 cm^−1^ Spectral Region

**Figure 3:**
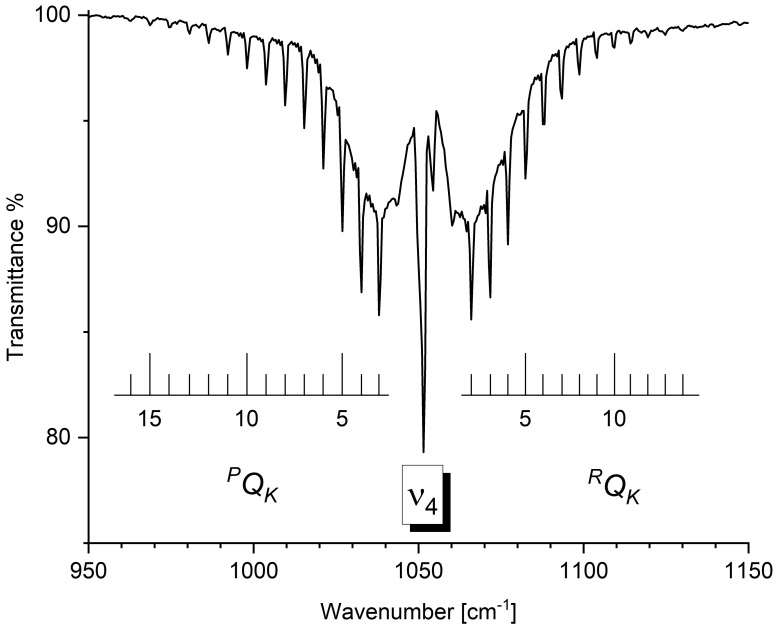
Gas-phase infrared spectrum of CHD_2_Cl in the spectral region 950–1150 cm^−1^. Resolution = 1.0 cm^−1^, room temperature, optical path length = 16.0 cm, pressure = 2.0 kPa. The assignments of ^*P*,*R*^*Q_K_* clusters of $\nu_{4}$ fundamental are reported.

**Figure 4:**
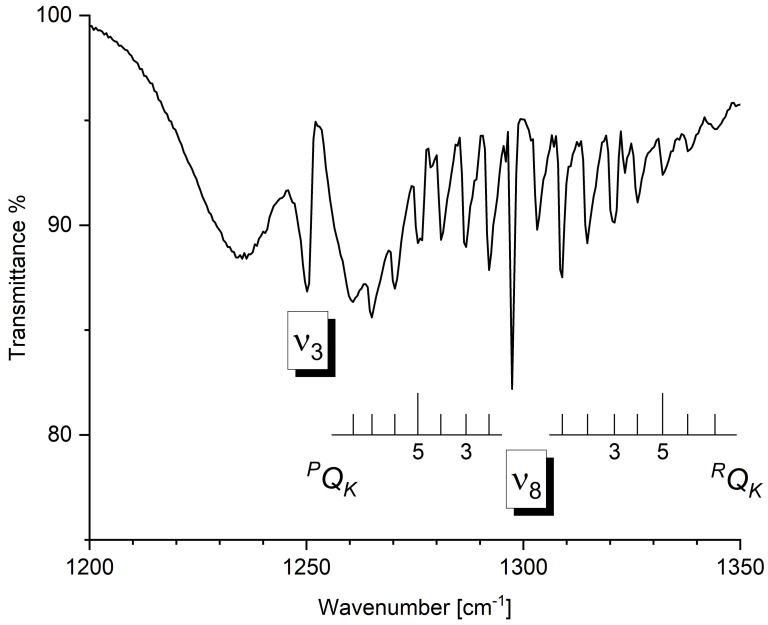
Gas-phase infrared spectrum of CHD_2_Cl in the spectral region 1200–1350 cm^−1^. Resolution = 1.0 cm^−1^, room temperature, optical path length = 16.0 cm, pressure = 2.0 kPa. The assignments of ^*P*,*R*^*Q_K_* clusters of $\nu_{8}$ fundamental are reported.

The 500–1500 cm^−1^ spectral region is dominated by the strong absorption of the $\nu_{6}$ band ($A^{\prime }$ symmetry; predicted anharmonic intensity: 18.13 km mol^−1^). For this fundamental, the ^35/37^Cl isotopologue splitting is predicted to be rather large (6 cm^−1^), and was found to be in remarkable agreement with the experimental data (705.9/700 cm^−1^). Moving to higher wavenumbers, the signals due to the $\nu_{5}$ ($A^{\prime }$ symmetry), $\nu_{4}$ ($A^{\prime }$ symmetry), $\nu_{3}$ ($A^{\prime }$ symmetry) and $\nu_{8}$ ($A^{\prime \prime }$ symmetry) bands are clearly visible. The agreement between the experimental and computed data for the assigned absorptions in this spectral region can be considered excellent, with a mean absolute error of 0.4 cm^−1^. For some of these fundamentals, a partially resolved rotational structure can be assigned. For example, the ^*P*,*R*^*Q_K_* clusters of $\nu_{4}$ ($A^{\prime }$ symmetry) and of $\nu_{8}$ ($A^{\prime \prime }$ symmetry) are labeled in Figure 3 and Figure 4, respectively. To analyze the partially resolved rotational structure of *b*- and *c*-type bands, a least-squares fit was carried out employing the following equation

(1) $$\nu^{P,R}=\nu_{0}+\left(A^{\prime }-{\bar{B}}^{\prime }\right)\mp 2\left(A^{\prime }-{\bar{B}}^{\prime }\right)K+\left[\left(A^{\prime }-{\bar{B}}^{\prime }\right)-\left(A^{\prime \prime }-{\bar{B}}^{\prime \prime }\right)\right]K^{2}\pm 4D_{K}^{\prime }K^{3}$$

where $\bar{B}=\left(B+C\right)/2$, and the upper and lower signs refer to the *P*- and *R*-branches, respectively. Table 8 reports the results obtained for all the bands analyzed.

#### 2.3.2. Vibrational Analysis of the 1500–3100 cm^−1^ Spectral Region

The two most relevant absorptions in this spectral region are the $\nu_{2}$ (centered at 2192 cm^−1^) and $\nu_{1}$ (centered at 3012 cm^−1^) fundamentals, both of $A^{\prime }$ symmetry. In addition, some signals due to two-quanta combination bands became visible and could be assigned: $2\nu_{4}$ (centered at 2089 cm^−1^), $\nu_{3}+\nu_{5}$ (centered at 2114 cm^−1^), $\nu_{7}$ (centered at 2278 cm^−1^) and $\nu_{3}+\nu_{4}$ (centered at 2296.33 cm^−1^). Even in this spectral region, the comparison between the experimental and computed data shows a remarkable agreement, the mean absolute error being 2.3 cm^−1^.

#### 2.3.3. Vibrational Analysis of the 3100–6200 cm^−1^ Spectral Region

This spectral region is characterized by the weak absorptions due to combination and overtone bands, mainly involving the $v_{1}=1$ vibrational state. The most intense signals are assigned to $\nu_{1}+\nu_{5}$ (centered at 3880 cm^−1^), $\nu_{1}+\nu_{3}$ (centered at 4242 cm^−1^), and $2\nu_{1}$ (centered at 5897 cm^−1^). For this spectral region, the overall agreement between the experimental data and the computed predictions can be considered satisfactory (mean absolute error equal to 7.0 cm^−1^).

### 2.4. Radiative Efficiency

The radiative efficiencies of R40 has been simulated by using the IR absorption cross section spectra computed at the rev-DSDPBEP86-D3/jun-cc-pV(T+*d*) level of theory employing the computational workflow recently developed in ref. [11]. This procedure accounts for non-empirical inclusion of both mechanical and electrical anharmonicity and, where needed, an automatic sampling of the conformational landscape through the CREST software [72]. In the present work, the approach has been further developed by including contribution from different isotopologues. In particular, anharmonic IR absorption cross section spectra of different R40 isotopologues have been weighted according to the relative isotopic abundance, and the resulting spectrum has been used to derive the istantaneous RE by means of the Pinnock’s narrowband model (NBM) [73]:

(2) $$IRE=\sum_{n=1}^{N}\int_{{\tilde{\nu }}_{i,1}}^{{\tilde{\nu }}_{i,2}}\sigma \left(\tilde{\nu }\right)d\tilde{\nu }]F_{\sigma }^{i}$$

where $\sigma (\tilde{\nu })$ is the IR absorbance cross section integrated over the spectral range between ${\tilde{\nu }}_{i,1}$ and ${\tilde{\nu }}_{i,2}$, and $F_{\sigma }^{i}$ is the radiative forcing per unit cross section of the global annual mean atmosphere (GAM). For the present work, the RF of the GAM by Shine and Myhre [74], which includes molecule dependent adjustments for stratospheric temperature, has been used. For all isotopologues, the RE evaluation has been conducted in the range from 500 cm^−1^ to 3000 cm^−1^, as none of them present IR absorptions in the low frequency region below ca. 600 cm^−1^.

**Figure 5:**
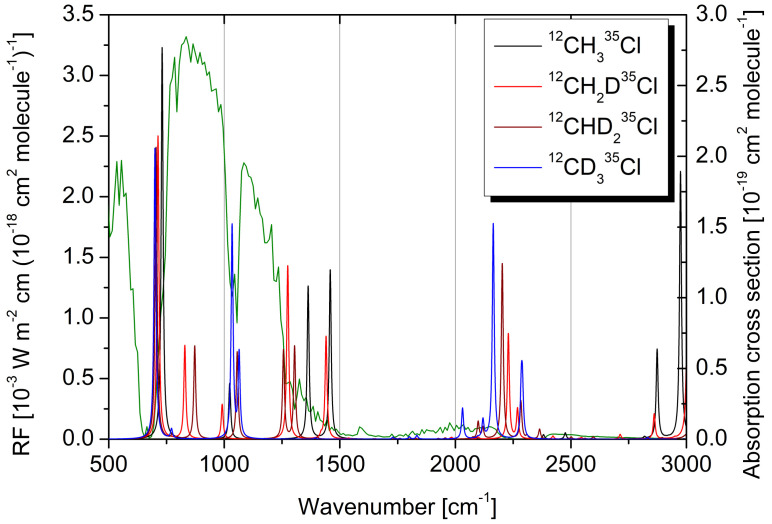
IR absorption cross section spectra of ^12^CH_3_^35^Cl, ^12^CH_2_D^35^Cl, ^12^CHD_2_^35^Cl and ^12^CD_3_^35^Cl (right axis scale) superimposed to the radiative forcing (RF) per unit cross section of the global annual mean atmosphere (green line, left axis scale).

The NBM assumes a well-mixed distribution of gases across altitude and latitude; however, when such conditions are not met, a correction accounting for the gas lifetime (τ) needs to be considered, thus providing the so-called effective RE (ERE). In our study, the RE has been adjusted for the R40 atmospheric lifetime of 0.9 years [13] considering the S-shaped curve [3] that assumes tropospheric OH degradation as the dominant removal process for this compound. The calculation of REs was performed using a home-made software, while further details about the methodology are reported in ref. [11]. The IRE of the main isotopic species, ^12^CH_3_^35^Cl, obtained using the simulated IR absorption cross section spectrum, has resulted to be 6.92 × $10^{-3}$ W m^−2^ ppbv^−1^, which compares positively with the value of 6.45 × $10^{-3}$ W m^−2^ ppbv^−1^ [13] determined from the experimentally measured cross section spectrum. By considering the lifetime correction, the QC IRE drops to an ERE of 5.00 × $10^{-3}$ W m^−2^ ppbv^−1^, again in good agreement with the value of 4.66 × $10^{-3}$ W m^−2^ ppbv^−1^ recommended in the last World Meteorological Organization (WMO) assessment on ozone depletion [13]. The theoretically estimated RE actually assumes that the totality of the R40 sample is constituted by the main isotopic species. While QC predictions of RE usually do not consider isotopologue distributions, and indeed, a good agreement with the experimental RE has been obtained for R40, in the present work, we exploited QC computations to explore the effects of isotopic substitution of the RE. Even though species containing more than one deuterium atom are expected to provide a negligible contribution to the RE, because of the low isotopic abundance of deuterium (0.0015) with respect to ^1^H (0.99985), it may be instructive to assess the effects of increasing deuteration on the radiative properties of R40. The IREs computed for isotopically pure samples (e.g., formed by 100% of ^12^CHD_2_^35^Cl) are listed in Table 9, while Figure 5 compares the simulated IR absorption cross section spectra of ^12^CH_3_^35^Cl, ^12^CH_2_D^35^Cl, ^12^CHD_2_^35^Cl and ^12^CD_3_^35^Cl, which are superimposed to the RF of the GAM. The RE varies non-linearly with the degree of deuteration because of the different number and intensities of isotopologues bands and the different overlaps with the Pinnock’s curve as shown in Figure 5.

**Figure 6:**
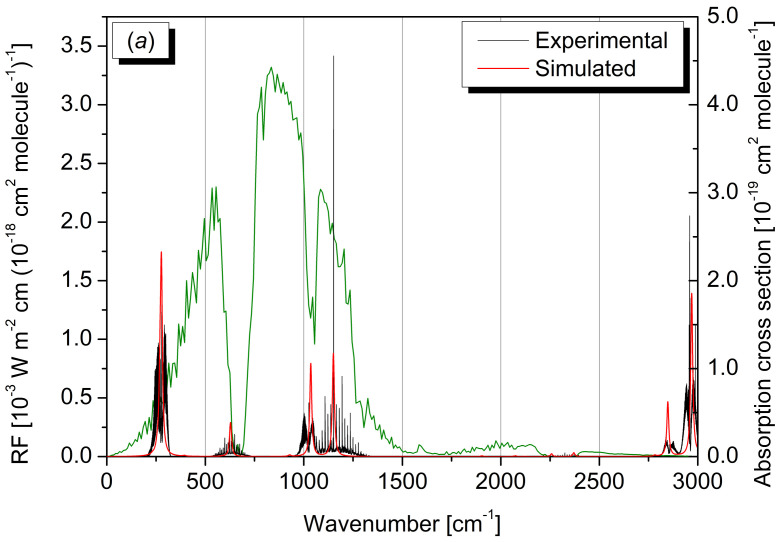

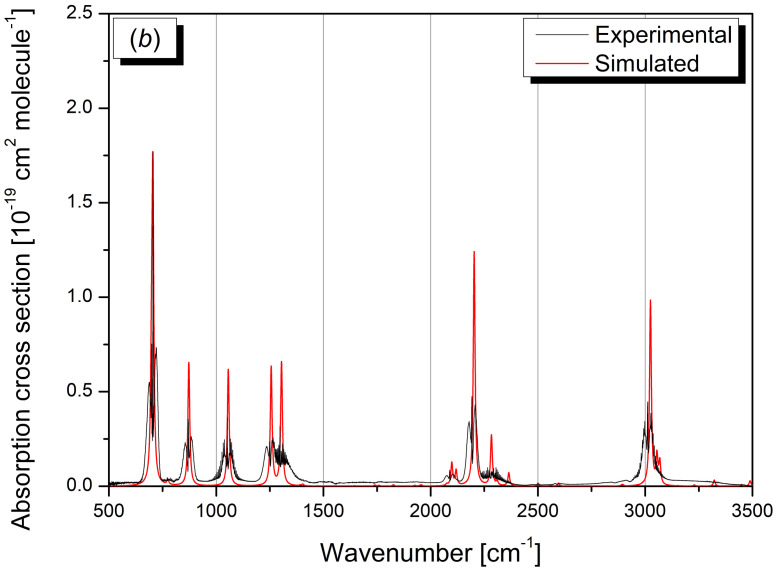
(**a**) IR absorption cross section spectrum of R40 in natural isotopic abundance (right axis scale) superimposed to the radiative forcing (RF) per unit cross section of the global annual mean atmosphere (green line, left axis scale); the black trace refers to the experimental spectrum, the red trace is the spectrum simulated at the rev-DSDPBEP86/jun-cc-pv(T+*d*)Z level of theory. (**b**) Comparison between the experimental (black) and simulated (red) spectra of CHD_2_^35/37^Cl.

The IR absorption cross section spectra computed for the different isotopologues have been weighted according to their relative abundances, thus providing the overall cross section spectrum reported in Figure 6, where it is also compared with the CH_3_Cl cross section spectrum determined experimentally [75]. The same Figure also compares the CHD_2_Cl FTIR spectrum here acquired experimentally, with the corresponding QC counterpart. In both cases, a very good agreement between the QC simulations and experimental traces can be noted. Finally, the simulated RE of R40 with natural isotopic composition has been derived from the corresponding QC absorption cross section spectrum. The IRE has resulted to be 6.74 $\times 10^{-3}$ W m^−2^ ppbv^−1^, which drops to 4.87 $\times 10^{-3}$ W m^−2^ ppbv^−1^ when accounting for the R40 lifetime correction. These values closely align with the best estimates reported in the last WMO ozone assessment report and, notably, the deviation of about 7% obtained by relying only on the IR absorption cross section spectrum of the ^12^CH_3_^35^Cl, reduces to 4% when accounting for the proper isotopic distribution, showing that, in addition to anharmonic effects and conformational equilibria [10,11], isotopologue contributions can also impact the accuracy in the *in silico* estimate of REs.

## 3. Materials and Methods

### 3.1. Computational Details

The theoretical equilibrium geometry of CH_3_Cl was derived by using a composite approach based on a hierarchy of geometry-based schemes recently proposed by Stanton and co-workers [70]. In particular, according to the variant denoted as “MEDIUM”, the best estimate of a generic geometrical parameter *r* can be obtained as follows:

(3) $$\begin{array}{c} r=r\left[HF/CV5Z\right]+\Delta r^{\infty }\left[CCSD\left(T\right)/CBS\left(Q,5\right)\right]+\Delta r^{fT}\left[CCSDT/VTZ\right]+\\ \Delta r^{fQ}\left[CCSDTQ/VDZ\right]+\Delta r^{DBOC}\left[HF/VTZ\right]+\Delta r^{SR}\left[CCSD\left(T\right)/CVTZ\right] \end{array}$$

where r[HF/CV5Z] is the value of the parameter evaluated at the Hartree-Fock level in conjunction with the cc-pwCV5Z basis set [76,77], while $\Delta r^{\infty }\left[CCSD\left(T\right)/CBS\left(Q,5\right)\right]$ is the correlation contribution at the CC singles, double and perturbative triples, CCSD(T) [78], with all electrons correlated, extrapolated to the complete basis set (CBS) limit. For this purpose, the two-point extrapolation formula proposed by Schwartz [79] was used in combination with the cc-pwCV5Z and cc-pwCVQZ basis sets [76,77]. The third and fourth terms on the rhs of Equation (3) represent, respectively, the contributions of full-triple, fT, and full-quadruple, fQ, excitations obtained using the CCSDT [80] and CCSDTQ [81] methods, respectively, and calculated as

(4) $$\Delta r^{fT}\left[CCSDT/VTZ\right]=r\left[CCSDT/VTZ\right]-r\left[CCSD\left(T\right)/VTZ\right]$$

(5) $$\Delta r^{fQ}\left[CCSDTQ/VDZ\right]=r\left[CCSDTQ/VDZ\right]-r\left[CCSDT/VDZ\right]$$

where VTZ and VDZ denote the cc-pVTZ and cc-pVDZ basis sets [76], respectively. Finally, $\Delta r^{DBOC}\left[HF/VDZ\right]$ and $\Delta r^{SR}\left[CCSD\left(T\right)/CVTZ\right]$ account for diagonal Born-Oppenheimer corrections (DBOC) and relativistic contributions, respectively, and are evaluated as

(6) $$\Delta r^{DBOC}\left[HF/VTZ\right]=r\left[DBOC-HF/VTZ\right]-r\left[HF/VTZ\right]$$

(7) $$\Delta r^{SR}\left[CCSD\left(T\right)/CVTZ\right]=\Delta r^{corr}\left[SFDC-CCSD\left(T\right)/CVTZ\right]-\Delta r^{corr}\left[CCSD\left(T\right)/CVTZ\right]$$

In Equation (6), r[DBOC−HF/VTZ] and r[HF/VTZ] represent the value of the geometrical parameter obtained at the HF/cc-pVTZ level accounting or not for DBOC [82], respectively. Relativistic corrections, Equation (7), were similarly evaluated as the difference between the structural parameter calculated by using the spin-free Dirac-Coulomb (SFDC) CCSD(T) method [83,84] and the corresponding non-relativistic value, both obtained using the uncontracted (unc) cc-pCVTZ basis set. In passing, it has to be noted that the only differences between the presently adopted composite method and the MEDIUM recipe proposed in ref. [70] lie in the use of the cc-pVDZ basis set, instead of the ANO0 one, for the estimation of the effects of quadruple excitations, and in the evaluation of the relativistic correction at the CCSD(T)/cc-pCVTZ-unc level of theory without the CCSD/cc-pCVQZ-unc correction.

The CH_3_Cl equilibrium geometry was also obtained through the SE approach [62,63]. This involves a non-linear least-squares fit of the structural parameters to the SE equilibrium moments of inertia, $I_{e,\beta }^{SE}$ (where β=a,b,c is one of the principal inertia axes of the molecule), of a set of isotopologues. The SE equilibrium moments of inertia are straightforwardly related to SE rotational constants, $B_{e,\beta }^{SE}$:

(8) $$I_{e,\beta }^{SE}=\frac{\hbar }{4\pi B_{e,\beta }^{SE}}$$

where *ℏ* is the reduced Planck constant and the SE equilibrium rotational constants are obtained as

(9) $$B_{e,\beta }^{SE}=B_{0,\beta }^{Exp}-\Delta B_{0,\beta }^{QC}$$

where $B_{0,\beta }^{Exp}$ denotes the experimental rotational constant for the vibrational ground state and $\Delta B_{vib,\beta }^{QC}$ is the computed correction term. This latter accounts for both the vibrational, $\Delta B_{vib,\beta }$, and electronic, $\Delta B_{ele,\beta }$, corrections:

(10) $$\Delta B_{0,\beta }^{QC}=\Delta B_{vib,\beta }+\Delta B_{ele,\beta }=-\sum_{k}\frac{d_{k}}{2}\alpha_{\beta }^{k}+\frac{m_{e}}{M_{P}}g_{\beta \beta }B_{e}^{\beta }$$

In the equation above, the sum runs over the *k* vibrational modes ($d_{k}$ denoting the corresponding degeneracies), $\alpha_{\beta }^{k}$ is the vibrational-rotational interaction constant, which depends on the semi-diagonal cubic force field, $m_{e}$ and $M_{P}$ are, respectively, the electron and proton mass, and $g_{\beta \beta }$ represents the elements of the rotational **g** tensor. The refinement of the SE equilibrium geometry was carried out using the MSR software [85]. Following previous works [86,87], all the terms appearing in Equation (10) were computed using methods rooted into density functional theory (DFT). Vibrational corrections to rotational constants ($\Delta B_{vib,\beta }$) were evaluated using the rev-DSDPBEP86 [88] double-hybrid functional in conjunction with the jun-cc-pVTZ basis set [89] supplemented by an additional set of *d* functions on the Cl atom to improve the accuracy of the results [8,10,90]. Furthermore, the SE equilibrium geometry was also derived by using vibrational corrections computed at the CCSD(T) level in conjunction with a quadruple-ζ basis set (see below). In all cases, electronic contributions ($\Delta B_{ele,\beta }$) were calculated employing the PW6B95 hybrid functional [91] in conjunction with the aug-cc-pV(T+*d*)Z basis set [76,92]. Both functionals were augmented for Grimme’s DFT-D3 correction, with Becke-Johnson damping [93,94], to treat dispersion effects.

The rev-DSDPBEP86-D3BJ/jun-cc-pVTZ level of theory was also employed to compute the semi-diagonal quartic force field required for the simulation of the IR absorption cross section spectrum, then used to estimate the RE of CH_3_Cl, CH_2_DCl, CHD_2_Cl and CD_3_Cl. Indeed, double-hybrid functionals like B2PLYP [95] and (rev-)DSDPBEP86 [88] combined with a triple-ζ basis set can be recommended for the purpose in view of their good performance in the prediction of structural and ro-vibrational spectroscopic properties [11,96,97,98,99]. In all cases geometry optimizations were performed at first and, then, harmonic frequencies and IR intensities were evaluated using analytical second-order derivatives of the potential energy surface (PES) and first-order derivatives of the dipole moment surface. Mechanical anharmonic effects were introduced using third and fourth-order derivatives of the PES, while second- and third-order derivatives of the dipole moment surface were computed to account for electrical anharmonicity. Higher order derivatives were calculated by numerical differentiation of analytical second- and first-order derivatives of the potential energy and dipole moments, respectively. Spectroscopic parameters were derived in the framework of vibrational perturbation theory to second-order (VPT2) [100,101] by using the computed equilibrium geometries, harmonic properties and anharmonic contributions.

Given the excellent agreement between predicted and experimental spectroscopic data established in the previous work on CH_2_DCl [61], the same computational strategy has been employed in this work to assist the rotational analysis in the millimeter-wave domain and to guide the vibrational assignments (in terms of fundamentals, combination and overtone bands) of the IR spectrum of CHD_2_Cl. Interested readers are referred to ref. [61] for a detailed account of the computational methodology. Shortly, harmonic data were obtained at the fc-CCSD(T) level of theory (fc standing for “frozen-core” approximation) using the cc-pV5Z basis set for H and C atoms, while for the Cl atom the corresponding *d*-augmented counterpart, aug-cc-pV(5+*d*)Z, was employed; this basis set is labeled as V5Z-aV(5+*d*)Z. In passing we note that, in the framework of rotational spectroscopy, harmonic force field calculations give access to the quartic centrifugal distortion terms. Cubic and quartic semi-diagonal force constants were calculated using the CCSD(T) method, within the fc approximation, in conjunction with the cc-pVQZ basis set (cc-pV(Q+*d*)Z for Cl); this basis set is labeled as VQZ-V(Q+*d*)Z. These computations provide the sextic centrifugal distortion constants. Concerning rotational constants, they were obtained by adding the vibrational corrections, computed at the fc-CCSD(T)/VQZ-V(Q+*d*)Z level of theory, to the equilibrium rotational constants issued from the previously determined SE equilibrium structure [61]. Moving to vibrational spectroscopy, a hybrid force field in a normal coordinate representation, obtained as implemented in an appropriate suite of programs [102], allowed the incorporation of the anharmonic fc-CCSD(T)/VQZ-V(Q+*d*)Z corrections into the harmonic fc-CCSD(T)/V5Z-aV(5+*d*)Z frequency values. This approach was already successfully used in the investigation of other chlorinated compounds [9,103]. The last spectroscopic parameters to be mentioned are the nuclear (chlorine) quadrupole coupling constants. Their values were obtained at the CCSD(T)/cc-pwCV5Z level of theory (with all electrons correlated) and augmented by vibrational corrections calculated at the fc-MP2/aug-cc-pVTZ level (MP2 standing for Møller-Plesset second-order theory [104]).

All DFT and MP2 calculations were carried out using the Gaussian16 software [105], which was also employed for applying VPT2 through its built-in generalized VPT2 engine [106,107], while coupled-cluster calculations were carried out using the CFOUR program suite [108]. Outputs of harmonic and anharmonic frequency computations carried with the CFOUR program can be found as supplementary materials.

### 3.2. Experimental Details

To prepare the CHD_2_Cl sample with natural isotopic composition for both C and Cl atoms, a synthesis similar to that previously carried out for obtaining CH_2_DCl was followed [61]. For more details, the reader is referred to ref. [109].

The rotational spectrum of CHD_2_Cl has been recorded using a frequency-modulation millimeter-wave spectrometer. The instrument has been described in detail elsewhere [110,111]; here, only a brief description is provided. The primary radiation source of the spectrometer is a Gunn diode (J. E. Carlstrom Co., Chicago, IL, USA) emitting between 80 and 115 GHz; higher frequencies are obtained by coupling the Gunn diode with passive or active frequency multipliers, in particular the WR5.1x2 one for the range 160–230 GHz and WR3.4x3 for the range 240–345 GHz (both from Virginia Diodes Inc., Charlottesville, VA, USA). The Gunn-diode radiation is frequency-stabilized by a phase-lock loop, in which the beating signal between the Gunn diode and a suitable harmonic of a centimeter-wave synthesizer (HP8672A, 2–18 GHz) is compared with a sine-wave modulated reference signal (IF) around 75 MHz. In this way, the Gunn-diode radiation is sine-wave modulated (at a frequency of *f* = 16.67 kHz) and can be scanned by sweeping the IF signal. The frequency accuracy is ensured by referencing all the signal generators to a rubidium atomic clock. The radiation is fed into a 3.25 m long glass absorption cell containing the vapors of bideuterated chloromethane. The pressure was maintained around 2–3 mTorr for the measurements of the most abundant ^12^CHD_2_^35^Cl and ^12^CHD_2_^37^Cl species, while for the rarer ^13^CHD_2_^35^Cl and ^13^CHD_2_^37^Cl isotopologues, a pressure of about 15 mTorr was used. The output radiation is then detected by a series of zero-biased Schottky barrier diodes (WR10ZBD for 80–115 GHz, WR5.1ZBD for 140–220 GHz, and WR3.4ZBD for 220–330 GHz). The detection signal is finally pre-amplified, filtered, and de-modulated at twice the modulation frequency (2f detection scheme) so that the recorded spectra are observed as the second derivative of the actual absorption profile.

IR spectra of CHD_2_Cl were recorded in the 500–6200 cm^−1^ spectral range employing a Bomem DA3.002 FTIR spectrometer [61] equipped with a KBr beam-splitter, mercury cadmium telluride and InSb detectors, and a Globar source. To improve the overall signal-to-noise ratio of the spectra, several hundred scans were co-added.

## 4. Conclusions

Methyl chloride, also denoted as R40, is a chlorinated organic compound bearing both atmospheric and astrophysical relevance. It is a relatively abundant trace gas in the atmosphere, with a mean tropospheric mixing ratio of about 550 ppt, which is mainly due to emissions from tropical vegetation and biomass burning [112]. Despite the fact that release from industrial activities is considered to be marginal, with the adoption of international agreements aimed at phasing out the production and usage of chlorofluorocarbons and hydro-chlorofluorocarbons, CH_3_Cl has become one of the major drivers of chlorine atoms into the stratosphere [113]. In addition to the atmospheric relevance, methyl chloride has been recently identified in the protostar IRAS 16,293–2422 and in the coma of comet 67P/Churyumov-Gerasimenko by the Rosetta mission [37]. These detections, on the one hand, have shown that even halogenated organics can be surprisingly synthesized near near young, Sun-like stars, on the other, they suggest the possibility of identifying its deuterated isotopologues.

In the present work, we have further deepened the spectroscopic characterization of methyl chloride by undertaking a detailed characterization of the rotational and vibrational spectroscopic features of the bideuterated isotopologue, CHD_2_Cl, using an integrated experimental and theoretical approach. More specifically, the analysis of the CHD_2_Cl pure rotational spectrum, acquired in the millimeter-wave spectral region, has led to the determination of an accurate set of spectroscopic parameters for the four ^12/13^CH$D_{2}^{35/37}$Cl isotopologues. In particular, rotational and centrifugal distortion constants of CH$D_{2}^{35/37}$Cl have been determined with an accuracy improved by at least one order of magnitude with respect to previous analyses. Furthermore, nuclear quadrupolar coupling constants as well as the spectroscopic parameters for the ^13^C-bearing bideuterated isotopologues have been here derived for the first time. These provide accurate predictions of the rotational transitions of the different CHD_2_Cl isotopologues, which in turn can be used for astronomical searches of these species. Under this point of view, it should be noted that the accuracy required for successful detections depends on the targeted source. On general grounds, however, line positions should be predicted with uncertainties similar to those that can be achieved under Doppler conditions in laboratory measurements (i.e., from few kHz to few tens of kHz). Hence, the transitions experimentally measured in this work are accurate enough to be employed in the interpretation of observational data.

The newly determined rotational constants together with those available in the literature for other 10 isotopologues, corrected for vibrational and electronic contributions, have been used to refine the SE equilibrium structure. In turn, this allowed us to assess the accuracy of different QC composite schemes rooted in the CC theory, which account for the extrapolation to the CBS limit, core-valence contribution, higher excitations in the CC expansion as well as DBOC and relativistic effects. The theoretical equilibrium structural parameters have resulted to be extremely accurate, with deviations smaller than 0.1 mÅ for bond lengths and 0.05° for the ClHC angle. Moving to the IR region, the characterization of the FTIR spectrum, supported by high-level QC predictions, has been carried out in the spectral region between 500 and 6200 cm^−1^, thus leading to the assignment of the fundamental bands and about 20 overtones and two-quanta combination transitions. In addition, the partially resolved rotational structure of some b- and c-type bands has been analyzed, thereby obtaining the corresponding molecular parameters. Finally, the RE of R40 has been simulated by means of a cost-effective computational procedure rooted into DFT. In this respect, a detailed analysis of isotopologue contributions to the molecule’s radiative properties have been carried out. The results show that, at variance with the commonly adopted QC procedures focusing only on the main isotopic species, proper consideration of isotopologues distribution, in addition to the non-empirical inclusion of anharmonic effects, improves the predictions and leads to climate metrics in close agreement with the most refined experimental determinations.

## Figures and Tables

**Table 1:** Computed harmonic vibrational frequencies of different isotopologues of CHD_2_Cl ^1^.

Normal Mode	Symmetry	Appr. Description	^12^CHD_2_^35^Cl	^12^CHD_2_^37^Cl	^13^CHD_2_^35^Cl	^13^CHD_2_^37^Cl
$\omega_{1}$	$A^{\prime }$	CH stretch	3152.9	3152.9	3143.3	3143.3
$\omega_{2}$	$A^{\prime }$	CD_2_ sym. stretch	2252.8	2252.8	2245.1	2245.1
$\omega_{3}$	$A^{\prime }$	CD_2_ deform	1279.4	1279.2	1275.4	1275.3
$\omega_{4}$	$A^{\prime }$	CD_2_ wag	1073.5	1073.5	1067.0	1067.0
$\omega_{5}$	$A^{\prime }$	H/D-C-Cl deform	883.8	883.3	873.3	872.8
$\omega_{6}$	$A^{\prime }$	C-Cl stretch	719.2	713.0	706.2	699.8
$\omega_{7}$	$A^{\prime \prime }$	CD_2_ asym. stretch	2363.8	2363.8	2346.6	2346.6
$\omega_{8}$	$A^{\prime \prime }$	CD_2_ twist	1332.6	1332.6	1329.1	1329.1
$\omega_{9}$	$A^{\prime \prime }$	CD_2_ rock	782.1	781.6	777.9	777.3

^1^ Obtained at fc-CCSD(T)/V5Z-aV(5+*d*)Z level of theory.

**Table 2:** Spectroscopic parameters determined for CH$D_{2}^{35}$Cl and CH$D_{2}^{37}$Cl.

Constant	Unit	Experiment ^1^	Theory ^2^	Previous	Experiment ^1^	Theory ^2^	Previous
		CH$D_{2}^{35}$Cl			CH$D_{2}^{37}$Cl		
*A*	MHz	95426.0588(23)	95423.147	95426.077(61)	95425.1307(67)	95422.232	95425.23(11)
*B*	MHz	11679.70196(17)	11679.727	11679.7051(79)	11485.12342(23)	11485.022	11485.129(14)
*C*	MHz	11370.06627(17)	11370.068	11370.0711(80)	11185.58835(23)	11185.684	11185.596(14)
$\Delta_{J}$	kHz	12.66567(41)	12.466	12.6698(92)	12.27402(72)	12.081	12.284(12)
$\Delta_{JK}$	kHz	127.1323(16)	126.232	127.36(19)	123.3620(20)	122.474	123.90(21)
$\Delta_{K}$	kHz	959.74(19)	925.802	955.3(12)	963.28(37)	929.752	958.3(14)
$\delta_{J}$	Hz	310.662(40)	299.671	310.2(4)	296.208(41)	285.950	295.9(3)
$\delta_{K}$	kHz	60.346(23)	56.242	59.95(22)	58.785(22)	54.566	58.26(20)
$\Phi_{J}$	mHz	−2.428	−2.428		−2.322	−2.322	
$\Phi_{JK}$	Hz	0.333	0.333	0.50(2)	0.346	0.346	0.64(10)
$\Phi_{KJ}$	Hz	2.649	2.649		2.717	2.717	
$\Phi_{K}$	Hz	31.603	31.603		31.501	31.501	
$\phi_{J}$	mHz	0.161	0.161		0.153	0.153	
$\phi_{JK}$	Hz	267.288	267.288		253.686	253.686	
$\phi_{K}$	Hz	23.853	23.853		24.136	24.136	
$\chi_{aa}$	MHz	−74.469(14)	−72.20	−74.52 ^3^	−58.711(21)	−56.90	−58.69 ^3^
$\chi_{bb}$	MHz	37.325(23)	36.01	37.260 ^3^	29.52(19)	28.38	29.345 ^3^

^1^ Numbers in parentheses are one standard deviation and apply to the last significant digits. Parameters without error are fixed at the corresponding theoretical value. ^2^ Equilibrium rotational constants from the SE equilibrium structure of ref. [61] augmented by fc-CCSD(T)/VQZ-V(Q+*d*)Z vibrational corrections; quartic centrifugal distortion constants at the fc-CCSD(T)/V5Z-aV(5+*d*)Z level; sextic centrifugal distortion constants at the fc-CCSD(T)/VQZ-V(Q+*d*)Z level; chlorine quadrupole coupling constants at the CCSD(T)/cc-pwCV5Z level (all electrons correlated) and augmented by fc-MP2/aug-cc-pVTZ vibrational corrections. See text. ^3^ Taken from ref. [66].

**Table 3:** Spectroscopic parameters determined for ^13^CH$D_{2}^{35}$Cl and ^13^CH$D_{2}^{37}$Cl.

Parameter	Unit	Experiment ^1^	Theory ^2^	Experiment ^1^	Theory ^2^
		^13^CH$D_{2}^{35}$Cl		^13^CH$D_{2}^{37}$Cl	
*A*	MHz	95358.5(50)	95355.295	95359.1(56)	95354.365
*B*	MHz	11330.5476(80)	11330.523	11134.1477(85)	11134.107
*C*	MHz	11039.9541(80)	11039.920	10853.4201(85)	10853.371
$\Delta_{J}$	kHz	12.0282(34)	11.835	11.6429(35)	11.454
$\Delta_{JK}$	kHz	122.738(19)	121.916	118.943(20)	118.162
$\Delta_{K}$	kHz	928.936	928.936	932.871	932.871
$\delta_{J}$	Hz	282.7(27)	274.503	272.2(30)	261.408
$\delta_{K}$	kHz	60.7(39)	53.918	57.7(42)	52.240
$\Phi_{J}$	mHz	−2.656	−2.656	−2.532	−2.532
$\Phi_{JK}$	Hz	0.306	0.306	0.287	0.287
$\Phi_{KJ}$	Hz	2.655	2.655	2.544	2.544
$\Phi_{K}$	Hz	32.0	32.0	32.0	32.0
$\phi_{J}$	mHz	0.128	0.128	0.122	0.122
$\phi_{JK}$	Hz	248.	248.	235.	235.
$\phi_{K}$	Hz	23.0	23.0	22.0	22.0
$\chi_{aa}$	MHz	−74.461(32)	−72.21	−58.751(53)	−56.91
$\chi_{bb}$	MHz	37.307(63)	36.02	29.71(19)	28.39

^1^ Numbers in parentheses are one standard deviation and apply to the last significant digits. Parameters without error are fixed at the theoretical value. ^2^ Equilibrium rotational constants from the SE equilibrium structure of ref. [61] augmented by fc-CCSD(T)/VQZ-V(Q+*d*)Z vibrational corrections; quartic centrifugal distortion constants at the fc-CCSD(T)/V5Z-aV(5+*d*)Z level; sextic centrifugal distortion constants at the fc-CCSD(T)/VQZ-V(Q+*d*)Z level; chlorine quadrupole coupling constants at the CCSD(T)/cc-pwCV5Z level (all electrons correlated) and augmented by fc-MP2/aug-cc-pVTZ vibrational corrections. See text.

**Table 4:** Vibrational and electronic contributions (MHz) to vibrational ground-state rotational constants for the different isotopologues of CH_3_Cl ^1^.

Isotopologue	$\Delta A_{\mathrm{vib}}^{\mathrm{CC}}$	$\Delta B_{\mathrm{vib}}^{\mathrm{CC}}$	$\Delta C_{\mathrm{vib}}^{\mathrm{CC}}$	$\Delta A_{\mathrm{vib}}^{\mathrm{rDSD}}$	$\Delta B_{\mathrm{vib}}^{\mathrm{rDSD}}$	$\Delta C_{\mathrm{vib}}^{\mathrm{rDSD}}$	$\Delta A_{\mathrm{ele}}^{\mathrm{PW}6}$	$\Delta B_{\mathrm{ele}}^{\mathrm{PW}6}$	$\Delta C_{\mathrm{ele}}^{\mathrm{PW}6}$
^12^CH_3_^35^Cl	−2044.154	−109.423	-	−1974.703	−106.636	-	21.628	−0.139	-
^12^CH_3_^37^Cl	−2044.542	−107.197	-	−1974.823	−104.508	-	21.628	−0.131	-
^13^CH_3_^35^Cl	−2033.338	−102.999	-	−1964.000	−100.400	-	21.628	−0.132	-
^13^CH_3_^37^Cl	−2033.459	−100.845	-	−1964.090	−98.302	-	21.628	−0.125	-
^12^CD_3_^35^Cl	−1000.000	−80.205	-	−744.565	−77.556	-	5.416	−0.102	-
^12^CD_3_^37^Cl	−1000.000	−78.472	-	−744.654	−75.877	-	5.416	−0.096	-
^12^CH_2_D^35^Cl	−1376.206	−94.668	−99.698	−1328.560	−92.306	−97.133	12.686	−0.123	−0.124
^12^CH_2_D^37^Cl	−1376.326	−92.744	−97.642	−1328.680	−90.417	−95.124	12.686	−0.116	−0.117
^13^CH_2_D^35^Cl	−1363.618	−89.632	−94.448	−1316.359	−87.390	−92.036	12.658	−0.118	−0.119
^13^CH_2_D^37^Cl	−1363.738	−87.726	−92.410	−1316.419	−85.501	−90.028	12.657	−0.111	−0.112
^12^CHD_2_^35^Cl	−1000.610	−85.817	−89.513	−966.231	−83.792	−87.180	8.032	−0.112	−0.112
^12^CHD_2_^37^Cl	−1000.731	−84.128	−87.514	−966.351	−82.023	−85.321	8.032	−0.106	−0.105
^13^CHD_2_^35^Cl	−991.185	−81.612	−85.161	−957.087	−79.655	−82.923	8.021	−0.107	−0.107
^13^CHD_2_^37^Cl	−991.305	−79.827	−83.283	−957.207	−77.916	−81.124	8.021	−0.101	−0.101

^1^ CCSD(T)/VQZ-V(Q+*d*)Z (CC) and rev-DSDPBEP86-D3/jun-cc-pV(T+*d*)Z (rDSD) vibrational corrections; electronic contributions at the PW6B95-D3/aug-cc-pV(T+*d*)Z (PW6) level.

**Table 5:** Semi-experimental and theoretical equilibrium structure of CH_3_Cl ^1^.

	$r_{e}^{\mathrm{SE}}$ ^2^	$r_{e}^{\mathrm{SE}}$ ^3^	$r_{e}^{\mathrm{SE}}$ ^4^	$r_{e}^{\mathrm{th}.}$ ^5^	$r_{e}^{\mathrm{th}.}$ ^6^	$r_{e}^{\mathrm{th}.}$ ^7^
*r*(C-Cl)	1.777716(10)	1.777863(50)	1.777725(11)	1.77686	1.77792	1.7777
*r*(C-H)	1.083484(17)	1.083699(65)	1.083450(17)	1.08338	1.08339	1.0834
α(Cl-C-H)	108.3723(18)	108.3739(81)	108.3740(19)	108.412	108.384	108.38

^1^ Bond lengths in Å, angle in °. ^2^ SE equilibrium structure from present work obtained using CCSD(T)/VQZ-V(Q+*d*)Z vibrational corrections. Figures in parentheses are 95% confidence intervals on the last significant digits. ^3^ SE equilibrium structure from present work obtained using rev-DSDPBEP86-D3/jun-cc-pV(T+*d*)Z vibrational corrections. Figures in parentheses are 95% confidence intervals on the last significant digits. ^4^ SE equilibrium structure from ref. [61]. Figures in parentheses are 95% confidence intervals on the last significant digits. ^5^ Equilibrium structure using the MEDIUM-like composite recipe (see text). ^6^ Equilibrium structure from CCSD(T)/CBS + CV + fT + fQ + DBOC + SR composite scheme. ^7^ Equilibrium structure from ref. [55] obtained from explicitly correlated CC calculations with extrapolation to the CBS limit and contributions due to CV correlation effects, higher-order coupled cluster excitations, scalar relativistic effects, and DBOC.

**Table 6:** Computed vibrational frequencies (Wvn, in cm^−1^ and anharmonic intensities (I, in km mol^−1^) of fundamental vibrations of different isotopologues of CHD_2_Cl ^1^.

	^12^CHD_2_^35^Cl		^12^CHD_2_^37^Cl		^13^CHD_2_^35^Cl		^13^CHD_2_^37^Cl	
	Wvn	I	Wvn	I	Wvn	I	Wvn	I
$\nu_{1}$	3010.4	9.56	3010.3	9.71	3001.2	10.40	3001.1	10.42
$\nu_{2}$	2193.9	11.07	2193.8	11.08	2185.2	10.80	2185.2	10.80
$\nu_{3}$	1251.4	5.57	1251.3	5.61	1247.9	4.86	1247.7	4.90
$\nu_{4}$	1051.9	5.55	1051.9	5.56	1045.8	5.23	1045.8	5.25
$\nu_{5}$	869.1	5.99	868.5	5.85	859.0	5.09	858.5	5.00
$\nu_{6}$	706.0	18.13	700.0	17.89	693.2	18.40	687.0	18.11
$\nu_{7}$	2274.5	2.15	2274.5	2.15	2259.9	2.30	2259.9	2.30.
$\nu_{8}$	1299.6	5.92	1299.4	5.92	1296.1	5.94	1296.0	5.94.
$\nu_{9}$	771.2	0.22	770.7	0.23	767.2	0.25	766.6	0.26.

^1^ Obtained from the hybrid force field combining harmonic data computed at the fc-CCSD(T)/V5Z-aV(5+*d*)Z level with anharmonic corrections calculated with the fc-CCSD(T)/VQZ-V(Q+*d*)Z level. See text for details.

**Table 7:** Vibrational assignments (in cm^−1^) of CHD_2_Cl and comparison with predicted values.

Band	Exp.	Wvn ^1^	I ^1^	Band	Exp.	Predicted ^1^	I ^1^
$\nu_{6}$	705.9(5)/700.0(5) ^2^	706	18.13	$2\nu_{8}$	2583.0(5)	2588	0.15
$\nu_{9}$	771.6(5)	771	0.22	$\nu_{1}$	3012.12(5) ^3^	3010	9.56
$\nu_{5}$	869.8(5)	869	5.99	$\nu_{4}+\nu_{7}$	3315.15(11) ^3^	3308	0.30
$\nu_{4}$	1052.25(4) ^3^	1052	5.55	$\nu_{2}+\nu_{3}$	3442.0(5)	3434	0.07
$\nu_{3}$	1250.3(5)	1251	5.57	$\nu_{2}+\nu_{8}$	3462.0(5)	3475	0.27
$\nu_{8}$	1300.50(6) ^3^	1300	5.92	$\nu_{7}+\nu_{8}$	3569.0(5)	3562	0.21
$2\nu_{6}$	1404.9(5)	1405	0.13	$\nu_{1}+\nu_{6}$	3733.0(5)	3719	0.05
$\nu_{4}+\nu_{5}$	1919.9(5)	1919	0.04	$\nu_{1}+\nu_{5}$	3880.0(5)	3877	0.30
$\nu_{3}+\nu_{6}$	1950.2(5)	1952	0.06	$\nu_{1}+\nu_{4}$	4060.0(5)	4061	0.07
$2\nu_{4}$	2089.0(5)	2091	0.80	$\nu_{1}+\nu_{3}$	4242.0(5)	4244	0.57
$\nu_{3}+\nu_{5}$	2114.0(5)	2112	0.61	$\nu_{1}+\nu_{8}$	4292.4(3)	4288	0.05
$\nu_{2}$	2192.0(5)	2194	11.07	$2\nu_{7}$	4526.8(3)	4510	0.20
$\nu_{7}$	2278.0(5)	2275	2.15	$\nu_{1}+\nu_{2}$	5202.0(5)	5192	0.04
$\nu_{3}+\nu_{4}$	2296.33(7) ^3^	2301	0.22	$\nu_{1}+\nu_{7}$	5290.0(5)	5286	0.05
$2\nu_{3}$	2491.0(5)	2492	0.15	$2\nu_{1}$	5897.0(5)	5898	0.52
$\nu_{3}+\nu_{8}$	2553.0(5)	2552	0.05				

^1^ Predicted anharmonic wavenumbers (Wvn, in cm^−1^) and intensities (I, in km mol^−1^) using the hybrid force field combining harmonic data computed at the fc-CCSD(T)/V5Z-aV(5+*d*)Z level with anharmonic corrections calculated with the fc-CCSD(T)/VQZ-V(Q+*d*)Z level. See text for details. ^2^^35/37^Cl isotopologue splitting. ^3^ Value obtained from the polynomial fit of partially resolved rotational structure. See text for details.

**Table 8:** Molecular parameters (cm^−1^) of the b- and c-type bands of CHD_2_^35^Cl ^1^.

Band	$\nu_{0}$	$\left(A^{\prime }-{\bar{B}}^{\prime }\right)$	$\left(\alpha^{A}-\alpha^{\bar{B}}\right)\times 10^{2}$	$D_{K}^{\prime }\times 10^{4}$	$\left(A^{\prime \prime }-{\bar{B}}^{\prime \prime }\right)$	Std. Dev. ^2^
$\nu_{4}$	1052.25(4)	2.786(4)	1.42(5)	0.32(14)	2.800(4)	0.122
$\nu_{8}$	1300.50(6)	2.821(4)	−2.49(25)	–	2.796(5)	0.116
$\nu_{3}+\nu_{4}$	2296.33(7)	2.724(9)	2.07(13)	0.76(56)	2.745(9)	0.207
$\nu_{1}$	3012.12(5)	2.788(4)	1.45(5)	0.48(17)	2.802(2)	0.117
$\nu_{4}+\nu_{7}$	3315.15(11)	2.781(7)	1.34(19)	–	2.788(7)	0.265

^1^ The uncertainties given in parentheses are one standard deviation of the last significant digit. The $\left(\alpha^{A}-\alpha^{\bar{B}}\right)$ data refer to the differences between the vibrational-rotational constants, α, for the A and $\bar{B}$ constants ($\bar{B}$ = (B + C)/2), respectively; these values are obtained as a by-product of the fit carried out by using Equation (9). ^2^ Standard deviation (cm^−1^).

**Table 9:** Radiative efficiency (10^−3^ W m^−2^ ppbv^−1^) of R40 isotopologues ^1^.

	^12^CH_3_^35^Cl	^12^CH_3_^37^Cl	^13^CH_3_^35^Cl	^13^CH_3_^37^Cl	^12^CD_3_^35^Cl	^12^CH_2_D^35^Cl	^12^CH_2_D^37^Cl	^12^CHD_2_^35^Cl
IRE ^2^	6.92	6.24	4.89	4.26	6.54	8.01	5.67	7.50
Weight. IRE ^3^	5.10	1.47	0.04	0.01	0.00	0.09	0.01	0.00

^1^ Only isotopologues providing a contribution to the total RE are reported with the exception of ^12^CD_3_^35^Cl and ^12^CHD_2_^35^Cl. ^2^ Instantaneous Radiative Efficiency (STA included) for the pure isotopologue. ^3^ Instantaneous Radiative Efficiency (STA included) weighted by the relative isotopic abundance.

## Data Availability

Data is available as Supporting Materials.

## Supplementary Materials

The following supporting information can be downloaded at: https://www.mdpi.com/article/10.3390/molecules30071604/s1, Table S1. Fit of the rotational transitions of ^12^CHD_2_^35^Cl in PIFORM format; Table S2. Fit of the rotational transitions of ^12^CHD_2_^37^Cl in PIFORM format; Table S3. Fit of the rotational transitions of ^13^CHD_2_^35^Cl in PIFORM format; Table S4. Fit of the rotational transitions of ^13^CHD_2_^37^Cl in PIFORM format; Table S5. Part of the CFOUR output file of the CCSD(T)/V5Z-aV(5+*d*)Z harmonic frequency calculation for ^12^CHD_2_^35^Cl; Table S6. Part of the CFOUR output file of the CCSD(T)/V5Z-aV(5+*d*)Z harmonic frequency calculation for ^12^CHD_2_^37^Cl; Table S7. Part of the CFOUR output file of the CCSD(T)/V5Z-aV(5+*d*)Z harmonic frequency calculation for ^13^CHD_2_^35^Cl; Table S8. Part of the CFOUR output file of the CCSD(T)/V5Z-aV(5+*d*)Z harmonic frequency calculation for ^13^CHD_2_^37^Cl; Table S9. Part of the CFOUR output file of the CCSD(T)/VQZ-aV(Q+*d*)Z anharmonic frequency calculation for ^12^CHD_2_^35^Cl; Table S10. Part of the CFOUR output file of the CCSD(T)/VQZ-aV(Q+*d*)Z anharmonic frequency calculation for ^12^CHD_2_^37^Cl; Table S11. Part of the CFOUR output file of the CCSD(T)/VQZ-aV(Q+*d*)Z anharmonic frequency calculation for ^13^CHD_2_^35^Cl; Table S12. Part of the CFOUR output file of the CCSD(T)/VQZ-aV(Q+*d*)Z anharmonic frequency calculation for ^13^CHD_2_^37^Cl.
