# Supplementary material for: Completing the Spectral Mosaic of Chloromethane by Adding the CHD2Cl Missing Piece Through the Interplay of Rotational/Vibrational Spectroscopy and Quantum Chemical Calculations
Source: Molecules. 2025 Apr 3;30(7):1604. doi: 10.3390/molecules30071604 (PMC11990711; doi:10.3390/molecules30071604)
Supplement: Supplementary file 1 [file molecules-30-01604-s001.zip › molecules-3531602-supplementary.pdf]

Supplementary Materials: Completing the spectral mosaic of chloromethane by adding the CHD_2Cl missing piece through the interplay of rotational/vibrational spectroscopy and quantum chemical calculations

Mattia Melosso [1], Paolo Stoppa [2], Daniela Alvarado-Jiménez [3,4], Filippo Tamassia [5], Carlotta Sapienza [1], Luca Bizzocchi [1], Luca Dore [1], Cristina Puzzarini [1,*], Andrea Pietropolli Charmet [2,*] and Nicola Tasinato [3,*]

List of supporting material available

Table S.1. Fit of the rotational transitions of $^{12}CHD_2{}^{35}Cl$ in PIFORM format.

Table S.2. Fit of the rotational transitions of $^{12}CHD_2{}^{37}Cl$ in PIFORM format.

Table S.3. Fit of the rotational transitions of $^{13}CHD_2{}^{35}Cl$ in PIFORM format.

Table S.4. Fit of the rotational transitions of $^{13}CHD_2{}^{37}Cl$ in PIFORM format.

Table S.5. Part of the CFOUR output file of the CCSD(T)/V5Z-aV(5+*d*)Z harmonic frequency calculation for $^{12}CHD_2{}^{35}Cl$.

Table S.6. Part of the CFOUR output file of the CCSD(T)/V5Z-aV(5+*d*)Z harmonic frequency calculation for $^{12}CHD_2{}^{37}Cl$.

Table S.7. Part of the CFOUR output file of the CCSD(T)/V5Z-aV(5+*d*)Z harmonic frequency calculation for $^{13}CHD_2{}^{35}Cl$.

Table S.8. Part of the CFOUR output file of the CCSD(T)/V5Z-aV(5+*d*)Z harmonic frequency calculation for $^{13}CHD_2{}^{37}Cl$.

Table S.9. Part of the CFOUR output file of the CCSD(T)/VQZ-aV(Q+*d*)Z anharmonic frequency calculation for $^{12}CHD_2{}^{35}Cl$.

Table S.10. Part of the CFOUR output file of the CCSD(T)/VQZ-aV(Q+*d*)Z anharmonic frequency calculation for $^{12}CHD_2{}^{37}Cl$.

Table S.11. Part of the CFOUR output file of the CCSD(T)/VQZ-aV(Q+*d*)Z anharmonic frequency calculation for $^{13}CHD_2{}^{35}Cl$.

Table S.12. Part of the CFOUR output file of the CCSD(T)/VQZ-aV(Q+*d*)Z anharmonic frequency calculation for $^{13}CHD_2{}^{37}Cl$.

Table S.1. Fit of the rotational transitions of $^{12}CHD_2{}^{35}Cl$ in PIFORM format

```
CHD2Cl -- 35Cl                                        Tue Mar 18 09:42:24 2025

---------------------------------------------------------------------------------------=========
                                              obs         o-c     error     blends     Notes
                                                                            o-c      wt
    / instead of : below denotes (o-c)>3*err
---------------------------------------------------------------------------------------=========
!Mallinson-TableII
   1:  5  0  5 10  7    4  1  3 10  6        29771.6170  -0.1104  0.020   0.0432 0.50
!Mallinson-TableII
   2:  5  0  5 10  4    4  1  3 10  3        29771.6170   0.1967  0.020   0.0432 0.50
!Mallinson-TableII
   3:  5  0  5 10  6    4  1  3 10  5        29768.8270  -0.1306  0.020   0.0221 0.50
!Mallinson-TableII
   4:  5  0  5 10  5    4  1  3 10  4        29768.8270   0.1748  0.020   0.0221 0.50
!Mallinson-TableII
   5:  8  2  6 10 10    9  1  8 10 11        37515.0420  -0.0778  0.035  -0.0280 0.50
!Mallinson-TableII
   6:  8  2  6 10  7    9  1  8 10  8        37515.0420   0.0218  0.035  -0.0280 0.50
!Mallinson-TableII
   7:  8  2  6 10  9    9  1  8 10 10        37517.5820  -0.0394  0.035   0.0109 0.50
!Mallinson-TableII
   8:  8  2  6 10  8    9  1  8 10  9        37517.5820   0.0611  0.035   0.0109 0.50
!Mallinson-TableII
   9:  9  2  8 10 11   10  1 10 10 12        29773.7720  -0.0105  0.010   0.0049 0.50
!Mallinson-TableII
  10:  9  2  8 10  8   10  1 10 10  9        29773.7720   0.0203  0.010   0.0049 0.50
!Mallinson-TableII
  11:  9  2  8 10 10   10  1 10 10 11        29775.7120  -0.0223  0.010  -0.0066 0.50
!Mallinson-TableII
  12:  9  2  8 10  9   10  1 10 10 10        29775.7120   0.0091  0.010  -0.0066 0.50
!Mallinson-TableII
  13: 12  1 11 10 14   11  2  9 10 13        36198.3260  -0.0222  0.042  -0.0135 0.50
!Mallinson-TableII
  14: 12  1 11 10 11   11  2  9 10 10        36198.3260  -0.0047  0.042  -0.0135 0.50
!Mallinson-TableII
  15: 12  1 11 10 13   11  2  9 10 12        36197.0360   0.0382  0.042   0.0468 0.50
!Mallinson-TableII
  16: 12  1 11 10 12   11  2  9 10 11        36197.0360   0.0554  0.042   0.0468 0.50
!Mallinson-TableII
  17: 13  1 13 10 15   12  2 11 10 14        33688.0350  -0.0380  0.062  -0.0218 0.50
!Mallinson-TableII
  18: 13  1 13 10 12   12  2 11 10 11        33688.0350  -0.0055  0.062  -0.0218 0.50
!Mallinson-TableII
  19: 13  1 13 10 14   12  2 11 10 13        33687.0250   0.0539  0.062   0.0701 0.50
!Mallinson-TableII
  20: 13  1 13 10 13   12  2 11 10 12        33687.0250   0.0862  0.062   0.0701 0.50
!Mallinson-TableII
  21: 12  2 10 10 14   13  0 13 10 15        37861.0280   0.0636  0.088   0.0650 0.50
!Mallinson-TableII
  22: 12  2 10 10 11   13  0 13 10 12        37861.0280   0.0663  0.088   0.0650 0.50
!Mallinson-TableII
  23: 12  2 10 10 13   13  0 13 10 14        37862.3280  -0.0548  0.088  -0.0534 0.50
!Mallinson-TableII
  24: 12  2 10 10 12   13  0 13 10 13        37862.3280  -0.0518  0.088  -0.0534 0.50
!Mallinson-TableII
  25: 16  3 14 10 18   17  2 16 10 19        28537.1450   0.0218  0.157   0.0257 0.50
!Mallinson-TableII
  26: 16  3 14 10 15   17  2 16 10 16        28537.1450   0.0295  0.157   0.0257 0.50
!Mallinson-TableII
  27: 16  3 14 10 17   17  2 16 10 18        28538.0360  -0.2016  0.157  -0.1977 0.50
!Mallinson-TableII
  28: 16  3 14 10 16   17  2 16 10 17        28538.0360  -0.1937  0.157  -0.1977 0.50
!Mallinson-TableII
  29: 20  2 19 10 22   19  3 17 10 21        39760.3040  -0.0491  0.091  -0.0452 0.50
!Mallinson-TableII
  30: 20  2 19 10 19   19  3 17 10 18        39760.3040  -0.0413  0.091  -0.0452 0.50
!Mallinson-TableII
  31: 20  2 19 10 21   19  3 17 10 20        39759.6440   0.0769  0.091   0.0808 0.50
!Mallinson-TableII
  32: 20  2 19 10 20   19  3 17 10 19        39759.6440   0.0846  0.091   0.0808 0.50
!Mallinson-TableII
  33:  1  1  0 10  3    2  0  2 10  4        37949.1890  -0.0118  0.050
!Mallinson-TableV
  34:  1  0  1  0  1    0  0  0  0  0        23049.8900   0.172 UNFITTD   23049.7176
```

```
!Mallinson-TableV
  35:  1  1  0  0  1    2  0  2  0  2        37956.3250  -0.0548  0.500
!Mallinson-TableV
  36:  2  0  2  0  2    1  0  1  0  1        46097.8000  -0.4767  0.200
!Mallinson-TableV
  37:  2  1  1  0  2    1  1  0  0  1        46406.2100  -1.7971  1.000
!Mallinson-TableV
  38:  2  1  2  0  2    1  1  1  0  1        45788.2100  -1.0281  1.000
!Mallinson-TableV
  39:  5  0  5  0  5    4  1  3  0  4        29770.3620   0.0323  0.020
!Mallinson-TableV
  40:  8  2  6  0  8    9  1  8  0  9        37516.3000  -0.0086  0.035
!Mallinson-TableV
  41:  9  2  8  0  9   10  1 10  0 10        29774.7310  -0.0004  0.010
!Mallinson-TableV
  42: 12  1 11  0 12   11  2  9  0 11        36197.6890   0.0162  0.042
!Mallinson-TableV
  43: 12  2 10  0 12   13  0 13  0 13        37861.6720   0.0058  0.088
!Mallinson-TableV
  44: 13  1 13  0 13   12  2 11  0 12        33687.5380   0.0247  0.062
!Mallinson-TableV
  45: 16  0 16  0 16   15  2 13  0 15        28822.1500   0.172 UNFITTD   28821.9776
!Mallinson-TableV
  46: 16  3 14  0 16   17  2 16  0 17        28537.5880  -0.0860  0.157
!Mallinson-TableV
  47: 20  2 19  0 20   19  3 17  0 19        39759.9760   0.0179  0.091
!Mallinson-TableV
  48: 23  4 19  0 23   24  3 21  0 24        33108.9480   0.0421  0.020
!Mallinson-TableV
  49: 23  4 20  0 23   24  3 22  0 24        33534.5580  -0.0227  0.020
!Mallinson-TableV
  50: 27  3 24  0 27   26  4 22  0 26        36580.5870  -0.0491  0.020
!Mallinson-TableV
  51: 27  3 25  0 27   26  4 23  0 26        35733.9270   0.0118  0.020
!Mallinson-TableV
  52: 30  5 25  0 30   31  4 27  0 31        39700.0210   0.100 UNFITTD   39699.9206
!Mallinson-TableV
  53: 30  5 26  0 30   31  4 28  0 31        39747.7760  -0.0168  0.020
!Mallinson-TableV
  54: 34  4 30  0 34   33  5 28  0 33        29642.2000   0.0117  0.020
!Mallinson-TableV
  55: 34  4 31  0 34   33  5 29  0 33        29543.4250  -0.0033  0.020
!Mallinson-TableV
  56: 13  1 12  0 13   13  1 13  0 13        28122.5740  -0.0063  0.020
!Mallinson-TableV
  57: 14  1 13  0 14   14  1 14  0 14        32440.8800  -0.0087  0.020
!Mallinson-TableV
  58: 15  1 14  0 15   15  1 15  0 15        37064.3570  -0.0074  0.020
!Mallinson-TableV
  59: 28  1 28  0 28   28  0 28  0 28        37983.7950  -0.0529  0.020
!Mallinson-TableV
  60: 29  1 29  0 29   29  0 29  0 29        35849.2880  -0.0167  0.020
!Mallinson-TableV
  61: 30  1 30  0 30   30  0 30  0 30        33770.1490  -0.0115  0.020
!Mallinson-TableV
  62: 30  2 28  0 30   30  2 29  0 30        28396.7500   0.0058  0.020
!Mallinson-TableV
  63: 31  1 31  0 31   31  0 31  0 31        31752.4170   0.0085  0.020
!Mallinson-TableV
  64: 31  2 29  0 31   31  2 30  0 31        32015.0000  -0.0008  0.020
!Mallinson-TableV
  65: 32  1 32  0 32   32  0 32  0 32        29801.2100   0.0202  0.020
!Mallinson-TableV
  66: 33  1 33  0 33   33  0 33  0 33        27920.8150   0.0330  0.020
  67:  4  1  4 10  5    3  1  3 10  5        91559.6990  -0.0078  0.010
  68:  4  1  4 10  4    3  1  3 10  3        91572.6800  -0.0008  0.010
  69:  4  1  4 10  5    3  1  3 10  4        91573.6470   0.0017  0.010
  70:  4  1  4 10  3    3  1  3 10  2        91574.5380  -0.0037  0.010
  71:  4  1  4 10  6    3  1  3 10  5        91575.5120   0.0042  0.010
  72:  4  1  4 10  4    3  1  3 10  4        91579.1870  -0.0009  0.010
  73:  4  1  4 10  3    3  1  3 10  3        91588.4770  -0.0061  0.020
  74:  4  0  4 10  5    3  0  3 10  5        92169.2350  -0.0014  0.010
  75:  4  3  1 10  4    3  3  0 10  4        92174.7190  -0.0117  0.010  -0.0074 0.50
  76:  4  3  2 10  4    3  3  1 10  4        92174.7190  -0.0029  0.010  -0.0074 0.50
  77:  4  3  1 10  5    3  3  0 10  4        92177.0190  -0.0000  0.010   0.0044 0.50
  78:  4  3  2 10  5    3  3  1 10  4        92177.0190   0.0087  0.010   0.0044 0.50
  79:  4  3  1 10  3    3  3  0 10  3        92179.0780  -0.0051  0.010  -0.0007 0.50
  80:  4  3  2 10  3    3  3  1 10  3        92179.0780   0.0037  0.010  -0.0007 0.50
```

81:	4	3	2	10	4	3	3	1	10	3	92185.6580	0.0733	0.010	-0.0023	0.15
82:	4	3	1	10	4	3	3	0	10	3	92185.6580	0.0644	0.010	-0.0023	0.15
83:	4	0	4	10	4	3	0	3	10	3	92185.6580	-0.0336	0.010	-0.0023	0.40
84:	4	0	4	10	3	3	0	3	10	2	92185.6580	-0.0342	0.010	-0.0023	0.30
85:	4	2	3	10	5	3	2	2	10	5	92186.3580	-0.0131	0.020		
86:	4	2	3	10	5	3	2	2	10	4	92186.3800	0.0131	0.020		
87:	4	0	4	10	6	3	0	3	10	5	92187.8610	0.0084	0.010	0.0084	0.50
88:	4	0	4	10	5	3	0	3	10	4	92187.8610	0.0082	0.010	0.0084	0.50
89:	4	2	3	10	4	3	2	2	10	4	92188.9660	-0.0110	0.020		
90:	4	2	3	10	4	3	2	2	10	3	92188.9860	0.0060	0.020		
91:	4	2	3	10	6	3	2	2	10	5	92193.7940	-0.0212	0.010	0.0101	0.33
92:	4	3	1	10	6	3	3	0	10	5	92193.7940	0.0213	0.010	0.0101	0.33
93:	4	3	2	10	6	3	3	1	10	5	92193.7940	0.0301	0.010	0.0101	0.33
94:	4	0	4	10	4	3	0	3	10	4	92194.3970	0.0136	0.080		
95:	4	2	2	10	5	3	2	1	10	5	92194.8990	-0.0137	0.010		
96:	4	2	2	10	5	3	2	1	10	4	92194.9190	0.0100	0.010		
97:	4	2	3	10	3	3	2	2	10	3	92196.4270	-0.0031	0.010		
98:	4	2	3	10	3	3	2	2	10	2	92196.4290	0.0051	0.010		
99:	4	2	2	10	4	3	2	1	10	4	92197.5080	-0.0113	0.010		
100:	4	2	2	10	4	3	2	1	10	3	92197.5250	0.0029	0.010		
101:	4	3	1	10	5	3	3	0	10	5	92200.2740	-0.0183	0.010	-0.0139	0.50
102:	4	3	2	10	5	3	3	1	10	5	92200.2740	-0.0095	0.010	-0.0139	0.50
103:	4	3	2	10	3	3	3	1	10	2	92202.3480	0.0057	0.010		
104:	4	2	2	10	6	3	2	1	10	5	92202.3520	-0.0056	0.010		
105:	4	3	1	10	3	3	3	0	10	2	92202.3570	0.0059	0.010		
106:	4	0	4	10	3	3	0	3	10	3	92204.2860	-0.0212	0.010		
107:	4	2	2	10	3	3	2	1	10	2	92204.9620	-0.0043	0.010		
108:	4	2	2	10	3	3	2	1	10	3	92204.9690	-0.0040	0.010		
109:	4	1	3	10	5	3	1	2	10	5	92797.0700	-0.0030	0.010		
110:	4	1	3	10	4	3	1	2	10	3	92810.0870	0.0001	0.010		
111:	4	1	3	10	5	3	1	2	10	4	92811.0600	0.0035	0.010		
112:	4	1	3	10	3	3	1	2	10	2	92811.9500	0.0022	0.010		
113:	4	1	3	10	6	3	1	2	10	5	92812.9280	0.0090	0.010		
114:	4	1	3	10	4	3	1	2	10	4	92816.6140	-0.0008	0.020		
115:	4	1	3	10	3	3	1	2	10	3	92825.9310	-0.0032	0.020		
116:	11	8	3	10	12	10	8	2	10	11	253302.6470	-0.0136	0.025	-0.0137	0.50
117:	11	8	4	10	12	10	8	3	10	11	253302.6470	-0.0136	0.025	-0.0137	0.50
118:	11	8	3	10	11	10	8	2	10	10	253303.5400	-0.0094	0.025	-0.0094	0.50
119:	11	8	4	10	11	10	8	3	10	10	253303.5400	-0.0094	0.025	-0.0094	0.50
120:	11	8	3	10	13	10	8	2	10	12	253308.0800	0.0033	0.025	0.0033	0.50
121:	11	8	4	10	13	10	8	3	10	12	253308.0800	0.0033	0.025	0.0033	0.50
122:	11	8	3	10	10	10	8	2	10	9	253308.9700	0.0047	0.025	0.0048	0.50
123:	11	8	4	10	10	10	8	3	10	9	253308.9700	0.0047	0.025	0.0048	0.50
124:	11	7	4	10	12	10	7	3	10	11	253346.2690	-0.0129	0.025	-0.0130	0.50
125:	11	7	5	10	12	10	7	4	10	11	253346.2690	-0.0129	0.025	-0.0130	0.50
126:	11	7	4	10	11	10	7	3	10	10	253346.9320	0.0249	0.025	0.0250	0.50
127:	11	7	5	10	11	10	7	4	10	10	253346.9320	0.0249	0.025	0.0250	0.50
128:	11	7	4	10	13	10	7	3	10	12	253350.4300	0.0011	0.025	0.0012	0.50
129:	11	7	5	10	13	10	7	4	10	12	253350.4300	0.0011	0.025	0.0012	0.50
130:	11	7	4	10	10	10	7	3	10	9	253351.0910	0.0376	0.025	0.0377	0.50
131:	11	7	5	10	10	10	7	4	10	9	253351.0910	0.0376	0.025	0.0377	0.50
132:	11	6	5	10	12	10	6	4	10	11	253384.7820	0.0183	0.025	0.0183	0.50
133:	11	6	6	10	12	10	6	5	10	11	253384.7820	0.0183	0.025	0.0183	0.50
134:	11	6	5	10	11	10	6	4	10	10	253385.1410	-0.0191	0.025	-0.0191	0.50
135:	11	6	6	10	11	10	6	5	10	10	253385.1410	-0.0191	0.025	-0.0191	0.50
136:	11	6	5	10	13	10	6	4	10	12	253387.8280	0.0175	0.025	0.0175	0.50
137:	11	6	6	10	13	10	6	5	10	12	253387.8280	0.0175	0.025	0.0175	0.50
138:	11	6	5	10	10	10	6	4	10	9	253388.1810	-0.0252	0.025	-0.0253	0.50
139:	11	6	6	10	10	10	6	5	10	9	253388.1810	-0.0252	0.025	-0.0253	0.50
140:	11	5	6	10	11	10	5	5	10	10	253418.6790	-0.1084	0.025	-0.0022	0.24
141:	11	5	6	10	12	10	5	5	10	11	253418.6790	0.0945	0.025	-0.0022	0.26
142:	11	5	7	10	11	10	5	6	10	10	253418.6790	-0.1083	0.025	-0.0022	0.24
143:	11	5	7	10	12	10	5	6	10	11	253418.6790	0.0945	0.025	-0.0022	0.26
144:	11	5	6	10	13	10	5	5	10	12	253420.7910	0.0905	0.025	0.0034	0.28
145:	11	5	6	10	10	10	5	5	10	9	253420.7910	-0.1117	0.025	0.0034	0.22
146:	11	5	7	10	13	10	5	6	10	12	253420.7910	0.0906	0.025	0.0034	0.28
147:	11	5	7	10	10	10	5	6	10	9	253420.7910	-0.1117	0.025	0.0034	0.22
148:	11	2	10	10	11	10	2	9	10	10	253439.9800	0.2598	0.025	-0.0011	0.24
149:	11	2	10	10	12	10	2	9	10	11	253439.9800	0.0930	0.025	-0.0011	0.26
150:	11	2	10	10	13	10	2	9	10	12	253439.9800	-0.2453	0.025	-0.0011	0.29
151:	11	2	10	10	10	10	2	9	10	9	253439.9800	-0.0784	0.025	-0.0011	0.22
152:	11	4	7	10	11	10	4	6	10	10	253448.9790	-0.0462	0.025	-0.0194	0.24
153:	11	4	7	10	12	10	4	6	10	11	253448.9790	-0.0017	0.025	-0.0194	0.26
154:	11	4	8	10	11	10	4	7	10	10	253448.9790	-0.0391	0.025	-0.0194	0.24
155:	11	4	8	10	12	10	4	7	10	11	253448.9790	0.0054	0.025	-0.0194	0.26
156:	11	4	7	10	13	10	4	6	10	12	253450.3360	0.0009	0.025	-0.0144	0.28
157:	11	4	7	10	10	10	4	6	10	9	253450.3360	-0.0430	0.025	-0.0144	0.22
158:	11	4	8	10	13	10	4	7	10	12	253450.3360	0.0081	0.025	-0.0144	0.28

159:	11	4	8	10	10	10	4	7	10	9	253450.3360	-0.0359	0.025	-0.0144 0.22
160:	11	3	9	10	11	10	3	8	10	10	253479.0100	0.0316	0.025	-0.0096 0.48
161:	11	3	9	10	12	10	3	8	10	11	253479.0100	-0.0471	0.025	-0.0096 0.52
162:	11	3	9	10	13	10	3	8	10	12	253479.7880	-0.0309	0.025	0.0031 0.57
163:	11	3	9	10	10	10	3	8	10	9	253479.7880	0.0480	0.025	0.0031 0.43
164:	11	3	8	10	11	10	3	7	10	10	253480.8840	0.0168	0.025	-0.0243 0.48
165:	11	3	8	10	12	10	3	7	10	11	253480.8840	-0.0618	0.025	-0.0243 0.52
166:	11	3	8	10	13	10	3	7	10	12	253481.6650	-0.0427	0.025	-0.0086 0.57
167:	11	3	8	10	10	10	3	7	10	9	253481.6650	0.0362	0.025	-0.0086 0.43
168:	11	2	9	10	11	10	2	8	10	10	253627.2360	0.2886	0.025	0.0272 0.24
169:	11	2	9	10	12	10	2	8	10	11	253627.2360	0.1218	0.025	0.0272 0.26
170:	11	2	9	10	13	10	2	8	10	12	253627.2360	-0.2174	0.025	0.0272 0.29
171:	11	2	9	10	10	10	2	8	10	9	253627.2360	-0.0506	0.025	0.0272 0.22
172:	11	1	10	10	11	10	1	9	10	10	255130.7090	0.1471	0.025	-0.0153 0.24
173:	11	1	10	10	12	10	1	9	10	11	255130.7090	-0.0727	0.025	-0.0153 0.26
174:	11	1	10	10	13	10	1	9	10	12	255130.7090	-0.1571	0.025	-0.0153 0.29
175:	11	1	10	10	10	10	1	9	10	9	255130.7090	0.0628	0.025	-0.0153 0.22
176:	11	2	9	10	13	11	1	11	10	13	262506.4530	-0.0467	0.025	0.0239 0.57
177:	11	2	9	10	10	11	1	11	10	10	262506.4530	0.1161	0.025	0.0239 0.43
178:	11	2	9	10	11	11	1	11	10	11	262507.6890	0.1117	0.025	0.0264 0.48
179:	11	2	9	10	12	11	1	11	10	12	262507.6890	-0.0515	0.025	0.0264 0.52
180:	16	0	16	10	15	15	1	14	10	14	264087.4500	-0.0399	0.025	0.0121 0.23
181:	16	0	16	10	16	15	1	14	10	15	264087.4500	0.1611	0.025	0.0121 0.24
182:	16	0	16	10	17	15	1	14	10	16	264087.4500	0.0702	0.025	0.0121 0.26
183:	16	0	16	10	18	15	1	14	10	17	264087.4500	-0.1308	0.025	0.0121 0.27
184:	12	1	12	10	11	11	1	11	10	10	274594.4200	0.0886	0.025	0.0202 0.22
185:	12	1	12	10	12	11	1	11	10	11	274594.4200	0.1535	0.025	0.0202 0.24
186:	12	1	12	10	13	11	1	11	10	12	274594.4200	-0.0323	0.025	0.0202 0.26
187:	12	1	12	10	14	11	1	11	10	13	274594.4200	-0.0972	0.025	0.0202 0.28
188:	12	11	1	10	13	11	11	0	10	12	276140.3770	-0.0169	0.025	-0.0170 0.50
189:	12	11	2	10	13	11	11	1	10	12	276140.3770	-0.0169	0.025	-0.0170 0.50
190:	12	11	1	10	12	11	11	0	10	11	276141.6790	-0.0127	0.025	-0.0127 0.50
191:	12	11	2	10	12	11	11	1	10	11	276141.6790	-0.0127	0.025	-0.0127 0.50
192:	12	11	1	10	14	11	11	0	10	13	276148.2630	-0.0068	0.025	-0.0069 0.50
193:	12	11	2	10	14	11	11	1	10	13	276148.2630	-0.0068	0.025	-0.0069 0.50
194:	12	11	1	10	11	11	11	0	10	10	276149.5670	-0.0020	0.025	-0.0021 0.50
195:	12	11	2	10	11	11	11	1	10	10	276149.5670	-0.0020	0.025	-0.0021 0.50
196:	12	10	2	10	13	11	10	1	10	12	276205.5100	0.0136	0.025	0.0136 0.50
197:	12	10	3	10	13	11	10	2	10	12	276205.5100	0.0136	0.025	0.0136 0.50
198:	12	10	2	10	12	11	10	1	10	11	276206.5520	0.0171	0.025	0.0171 0.50
199:	12	10	3	10	12	11	10	2	10	11	276206.5520	0.0171	0.025	0.0171 0.50
200:	12	10	2	10	14	11	10	1	10	13	276212.0010	-0.0047	0.025	-0.0047 0.50
201:	12	10	3	10	14	11	10	2	10	13	276212.0010	-0.0047	0.025	-0.0047 0.50
202:	12	10	2	10	11	11	10	1	10	10	276213.0450	0.0003	0.025	0.0003 0.50
203:	12	10	3	10	11	11	10	2	10	10	276213.0450	0.0003	0.025	0.0003 0.50
204:	12	0	12	10	13	11	0	11	10	13	276248.0600	-0.0055	0.025	
205:	12	9	3	10	13	11	9	2	10	12	276264.6220	-0.0286	0.025	-0.0287 0.50
206:	12	9	4	10	13	11	9	3	10	12	276264.6220	-0.0286	0.025	-0.0287 0.50
207:	12	9	3	10	12	11	9	2	10	11	276265.4430	-0.0113	0.025	-0.0114 0.50
208:	12	9	4	10	12	11	9	3	10	11	276265.4430	-0.0113	0.025	-0.0114 0.50
209:	12	0	12	10	11	11	0	11	10	10	276266.5900	0.1126	0.025	0.0045 0.22
210:	12	0	12	10	12	11	0	11	10	11	276266.5900	0.1115	0.025	0.0045 0.24
211:	12	0	12	10	13	11	0	11	10	12	276266.5900	-0.0869	0.025	0.0045 0.26
212:	12	0	12	10	14	11	0	11	10	13	276266.5900	-0.0859	0.025	0.0045 0.28
213:	12	9	3	10	14	11	9	2	10	13	276269.9120	-0.0114	0.025	-0.0114 0.50
214:	12	9	4	10	14	11	9	3	10	13	276269.9120	-0.0114	0.025	-0.0114 0.50
215:	12	9	3	10	11	11	9	2	10	10	276270.7480	0.0209	0.025	0.0209 0.50
216:	12	9	4	10	11	11	9	3	10	10	276270.7480	0.0209	0.025	0.0209 0.50
217:	12	0	12	10	11	11	0	11	10	11	276285.0790	-0.0097	0.025	
218:	12	8	4	10	13	11	8	3	10	12	276317.9050	-0.0196	0.025	-0.0197 0.50
219:	12	8	5	10	13	11	8	4	10	12	276317.9050	-0.0196	0.025	-0.0197 0.50
220:	12	8	4	10	12	11	8	3	10	11	276318.5610	0.0427	0.025	0.0427 0.50
221:	12	8	5	10	12	11	8	4	10	11	276318.5610	0.0427	0.025	0.0427 0.50
222:	12	8	4	10	14	11	8	3	10	13	276322.0740	-0.0169	0.025	-0.0170 0.50
223:	12	8	5	10	14	11	8	4	10	13	276322.0740	-0.0169	0.025	-0.0170 0.50
224:	12	8	4	10	11	11	8	3	10	10	276322.7280	0.0438	0.025	0.0439 0.50
225:	12	8	5	10	11	11	8	4	10	10	276322.7280	0.0438	0.025	0.0439 0.50
226:	12	7	5	10	12	11	7	4	10	11	276365.6260	-0.2367	0.025	-0.0241 0.24
227:	12	7	5	10	13	11	7	4	10	12	276365.6260	0.1713	0.025	-0.0241 0.26
228:	12	7	6	10	12	11	7	5	10	11	276365.6260	-0.2367	0.025	-0.0241 0.24
229:	12	7	6	10	13	11	7	5	10	12	276365.6260	0.1713	0.025	-0.0241 0.26
230:	12	7	5	10	11	11	7	4	10	10	276368.7660	-0.2861	0.025	-0.0567 0.22
231:	12	7	5	10	14	11	7	4	10	13	276368.7660	0.1214	0.025	-0.0567 0.28
232:	12	7	6	10	11	11	7	5	10	10	276368.7660	-0.2861	0.025	-0.0567 0.22
233:	12	7	6	10	14	11	7	5	10	13	276368.7660	0.1214	0.025	-0.0567 0.28
234:	12	6	6	10	12	11	6	5	10	11	276407.6280	-0.1396	0.025	-0.0108 0.24
235:	12	6	6	10	13	11	6	5	10	12	276407.6280	0.1075	0.025	-0.0108 0.26
236:	12	6	7	10	12	11	6	6	10	11	276407.6280	-0.1396	0.025	-0.0108 0.24

237:	12	6	7	10	13	11	6	6	10	12	276407.6280	0.1075	0.025	-0.0108	0.26
238:	12	6	6	10	11	11	6	5	10	10	276409.9560	-0.1548	0.025	-0.0159	0.22
239:	12	6	6	10	14	11	6	5	10	13	276409.9560	0.0918	0.025	-0.0159	0.28
240:	12	6	7	10	11	11	6	6	10	10	276409.9560	-0.1548	0.025	-0.0159	0.22
241:	12	6	7	10	14	11	6	6	10	13	276409.9560	0.0918	0.025	-0.0159	0.28
242:	12	5	7	10	12	11	5	6	10	11	276444.7920	-0.0645	0.025	-0.0067	0.24
243:	12	5	7	10	13	11	5	6	10	12	276444.7920	0.0465	0.025	-0.0067	0.26
244:	12	5	8	10	12	11	5	7	10	11	276444.7920	-0.0645	0.025	-0.0067	0.24
245:	12	5	8	10	13	11	5	7	10	12	276444.7920	0.0465	0.025	-0.0067	0.26
246:	12	5	7	10	11	11	5	6	10	10	276446.4140	-0.0697	0.025	-0.0075	0.22
247:	12	5	7	10	14	11	5	6	10	13	276446.4140	0.0408	0.025	-0.0075	0.28
248:	12	5	8	10	11	11	5	7	10	10	276446.4140	-0.0697	0.025	-0.0075	0.22
249:	12	5	8	10	14	11	5	7	10	13	276446.4140	0.0409	0.025	-0.0075	0.28
250:	12	2	11	10	11	11	2	10	10	10	276459.5570	-0.0392	0.025	0.0097	0.22
251:	12	2	11	10	12	11	2	10	10	11	276459.5570	0.2209	0.025	0.0097	0.24
252:	12	2	11	10	13	11	2	10	10	12	276459.5570	0.0720	0.025	0.0097	0.26
253:	12	2	11	10	14	11	2	10	10	13	276459.5570	-0.1882	0.025	0.0097	0.28
254:	12	4	8	10	12	11	4	7	10	11	276478.7300	-0.0083	0.025	-0.0018	0.24
255:	12	4	8	10	13	11	4	7	10	12	276478.7300	-0.0086	0.025	-0.0018	0.26
256:	12	4	9	10	12	11	4	8	10	11	276478.7300	0.0050	0.025	-0.0018	0.24
257:	12	4	9	10	13	11	4	8	10	12	276478.7300	0.0047	0.025	-0.0018	0.26
258:	12	4	8	10	11	11	4	7	10	10	276479.7770	-0.0027	0.025	0.0036	0.22
259:	12	4	8	10	14	11	4	7	10	13	276479.7770	-0.0034	0.025	0.0036	0.28
260:	12	4	9	10	11	11	4	8	10	10	276479.7770	0.0106	0.025	0.0036	0.22
261:	12	4	9	10	14	11	4	8	10	13	276479.7770	0.0099	0.025	0.0036	0.28
262:	12	3	10	10	12	11	3	9	10	11	276513.2230	0.0251	0.025	-0.0202	0.48
263:	12	3	10	10	13	11	3	9	10	12	276513.2230	-0.0618	0.025	-0.0202	0.52
264:	12	3	10	10	11	11	3	9	10	10	276513.8800	0.0962	0.025	0.0472	0.44
265:	12	3	10	10	14	11	3	9	10	13	276513.8800	0.0090	0.025	0.0472	0.56
266:	12	3	9	10	12	11	3	8	10	11	276516.1470	0.0124	0.025	-0.0329	0.48
267:	12	3	9	10	13	11	3	8	10	12	276516.1470	-0.0745	0.025	-0.0329	0.52
268:	12	3	9	10	11	11	3	8	10	10	276516.8050	0.0845	0.025	0.0354	0.44
269:	12	3	9	10	14	11	3	8	10	13	276516.8050	-0.0026	0.025	0.0354	0.56
270:	12	2	10	10	11	11	2	9	10	10	276702.6250	-0.0347	0.025	0.0147	0.22
271:	12	2	10	10	12	11	2	9	10	11	276702.6250	0.2264	0.025	0.0147	0.24
272:	12	2	10	10	13	11	2	9	10	12	276702.6250	0.0776	0.025	0.0147	0.26
273:	12	2	10	10	14	11	2	9	10	13	276702.6250	-0.1836	0.025	0.0147	0.28
274:	12	1	11	10	11	11	1	10	10	10	278299.9320	0.0954	0.025	0.0267	0.22
275:	12	1	11	10	12	11	1	10	10	11	278299.9320	0.1602	0.025	0.0267	0.24
276:	12	1	11	10	13	11	1	10	10	12	278299.9320	-0.0261	0.025	0.0267	0.26
277:	12	1	11	10	14	11	1	10	10	13	278299.9320	-0.0909	0.025	0.0267	0.28
278:	13	11	2	10	14	12	11	1	10	13	299137.4950	-0.0090	0.025	-0.0091	0.50
279:	13	11	3	10	14	12	11	2	10	13	299137.4950	-0.0090	0.025	-0.0091	0.50
280:	13	11	2	10	13	12	11	1	10	12	299138.4460	0.0271	0.025	0.0271	0.50
281:	13	11	3	10	13	12	11	2	10	12	299138.4460	0.0271	0.025	0.0271	0.50
282:	13	11	2	10	15	12	11	1	10	14	299143.6910	-0.0015	0.025	-0.0016	0.50
283:	13	11	3	10	15	12	11	2	10	14	299143.6910	-0.0015	0.025	-0.0016	0.50
284:	13	11	2	10	12	12	11	1	10	11	299144.6460	0.0381	0.025	0.0382	0.50
285:	13	11	3	10	12	12	11	2	10	11	299144.6460	0.0381	0.025	0.0382	0.50
286:	13	10	3	10	14	12	10	2	10	13	299207.8280	-0.0352	0.025	-0.0353	0.50
287:	13	10	4	10	14	12	10	3	10	13	299207.8280	-0.0352	0.025	-0.0353	0.50
288:	13	10	3	10	13	12	10	2	10	12	299208.6110	0.0207	0.025	0.0207	0.50
289:	13	10	4	10	13	12	10	3	10	12	299208.6110	0.0207	0.025	0.0207	0.50
290:	13	10	3	10	15	12	10	2	10	14	299212.9520	-0.0259	0.025	-0.0259	0.50
291:	13	10	4	10	15	12	10	3	10	14	299212.9520	-0.0259	0.025	-0.0259	0.50
292:	13	10	3	10	12	12	10	2	10	11	299213.7410	0.0360	0.025	0.0361	0.50
293:	13	10	4	10	12	12	10	3	10	11	299213.7410	0.0360	0.025	0.0361	0.50
294:	13	0	13	10	12	12	0	12	10	11	299226.8370	0.1076	0.025	0.0163	0.22
295:	13	0	13	10	13	12	0	12	10	12	299226.8370	0.1065	0.025	0.0163	0.24
296:	13	0	13	10	14	12	0	12	10	13	299226.8370	-0.0620	0.025	0.0163	0.26
297:	13	0	13	10	15	12	0	12	10	14	299226.8370	-0.0609	0.025	0.0163	0.28
298:	13	9	4	10	14	12	9	3	10	13	299271.8090	-0.0226	0.025	-0.0227	0.50
299:	13	9	5	10	14	12	9	4	10	13	299271.8090	-0.0226	0.025	-0.0227	0.50
300:	13	9	4	10	13	12	9	3	10	12	299272.4110	0.0223	0.025	0.0223	0.50
301:	13	9	5	10	13	12	9	4	10	12	299272.4110	0.0223	0.025	0.0223	0.50
302:	13	9	4	10	15	12	9	3	10	14	299275.9520	-0.0226	0.025	-0.0227	0.50
303:	13	9	5	10	15	12	9	4	10	14	299275.9520	-0.0226	0.025	-0.0227	0.50
304:	13	9	4	10	12	12	9	3	10	11	299276.5670	0.0356	0.025	0.0356	0.50
305:	13	9	5	10	12	12	9	4	10	11	299276.5670	0.0356	0.025	0.0356	0.50
306:	13	8	5	10	13	12	8	4	10	12	299329.6790	-0.2245	0.025	-0.0142	0.24
307:	13	8	5	10	14	12	8	4	10	13	299329.6790	0.1803	0.025	-0.0142	0.26
308:	13	8	6	10	13	12	8	5	10	12	299329.6790	-0.2245	0.025	-0.0142	0.24
309:	13	8	6	10	14	12	8	5	10	13	299329.6790	0.1803	0.025	-0.0142	0.26
310:	13	8	5	10	12	12	8	4	10	11	299332.9010	-0.2756	0.025	-0.0500	0.22
311:	13	8	5	10	15	12	8	4	10	14	299332.9010	0.1287	0.025	-0.0500	0.28
312:	13	8	6	10	12	12	8	5	10	11	299332.9010	-0.2756	0.025	-0.0500	0.22
313:	13	8	6	10	15	12	8	5	10	14	299332.9010	0.1287	0.025	-0.0500	0.28
314:	13	7	6	10	13	12	7	5	10	12	299381.1720	-0.1386	0.025	0.0019	0.24

315:	13	7	6	10	14	12	7	5	10	13	299381.1720	0.1319	0.025	0.0019	0.26
316:	13	7	7	10	13	12	7	6	10	12	299381.1720	-0.1386	0.025	0.0019	0.24
317:	13	7	7	10	14	12	7	6	10	13	299381.1720	0.1319	0.025	0.0019	0.26
318:	13	7	6	10	12	12	7	5	10	11	299383.6600	-0.1565	0.025	-0.0059	0.22
319:	13	7	6	10	15	12	7	5	10	14	299383.6600	0.1134	0.025	-0.0059	0.28
320:	13	7	7	10	12	12	7	6	10	11	299383.6600	-0.1565	0.025	-0.0059	0.22
321:	13	7	7	10	15	12	7	6	10	14	299383.6600	0.1134	0.025	-0.0059	0.28
322:	13	6	7	10	13	12	6	6	10	12	299426.8860	-0.0823	0.025	-0.0023	0.24
323:	13	6	7	10	14	12	6	6	10	13	299426.8860	0.0717	0.025	-0.0023	0.26
324:	13	6	8	10	13	12	6	7	10	12	299426.8860	-0.0823	0.025	-0.0023	0.24
325:	13	6	8	10	14	12	6	7	10	13	299426.8860	0.0717	0.025	-0.0023	0.26
326:	13	6	7	10	12	12	6	6	10	11	299428.7200	-0.0893	0.025	-0.0036	0.22
327:	13	6	7	10	15	12	6	6	10	14	299428.7200	0.0642	0.025	-0.0036	0.28
328:	13	6	8	10	12	12	6	7	10	11	299428.7200	-0.0893	0.025	-0.0036	0.22
329:	13	6	8	10	15	12	6	7	10	14	299428.7200	0.0642	0.025	-0.0036	0.28
330:	13	5	8	10	13	12	5	7	10	12	299467.6370	-0.0342	0.025	-0.0053	0.24
331:	13	5	8	10	14	12	5	7	10	13	299467.6370	0.0213	0.025	-0.0053	0.26
332:	13	5	9	10	13	12	5	8	10	12	299467.6370	-0.0341	0.025	-0.0053	0.24
333:	13	5	9	10	14	12	5	8	10	13	299467.6370	0.0213	0.025	-0.0053	0.26
334:	13	5	8	10	12	12	5	7	10	11	299468.9160	-0.0337	0.025	-0.0029	0.22
335:	13	5	8	10	15	12	5	7	10	14	299468.9160	0.0214	0.025	-0.0029	0.28
336:	13	5	9	10	12	12	5	8	10	11	299468.9160	-0.0336	0.025	-0.0029	0.22
337:	13	5	9	10	15	12	5	8	10	14	299468.9160	0.0215	0.025	-0.0029	0.28
338:	13	2	12	10	12	12	2	11	10	11	299473.7910	-0.0261	0.025	0.0042	0.22
339:	13	2	12	10	13	12	2	11	10	12	299473.7910	0.1782	0.025	0.0042	0.24
340:	13	2	12	10	14	12	2	11	10	13	299473.7910	0.0455	0.025	0.0042	0.26
341:	13	2	12	10	15	12	2	11	10	14	299473.7910	-0.1589	0.025	0.0042	0.28
342:	13	4	9	10	13	12	4	8	10	12	299505.4460	-0.0238	0.025	-0.0250	0.24
343:	13	4	9	10	14	12	4	8	10	13	299505.4460	-0.0489	0.025	-0.0250	0.26
344:	13	4	10	10	13	12	4	9	10	12	299505.4460	-0.0000	0.025	-0.0250	0.24
345:	13	4	10	10	14	12	4	9	10	13	299505.4460	-0.0251	0.025	-0.0250	0.26
346:	13	4	9	10	12	12	4	8	10	11	299506.3090	0.0208	0.025	0.0186	0.22
347:	13	4	9	10	15	12	4	8	10	14	299506.3090	-0.0045	0.025	0.0186	0.28
348:	13	4	10	10	12	12	4	9	10	11	299506.3090	0.0446	0.025	0.0186	0.22
349:	13	4	10	10	15	12	4	9	10	14	299506.3090	0.0193	0.025	0.0186	0.28
350:	13	3	11	10	13	12	3	10	10	12	299544.9270	0.0917	0.025	0.0461	0.48
351:	13	3	11	10	14	12	3	10	10	13	299544.9270	0.0038	0.025	0.0461	0.52
352:	13	3	11	10	12	12	3	10	10	11	299545.2900	-0.0056	0.025	-0.0548	0.44
353:	13	3	11	10	15	12	3	10	10	14	299545.2900	-0.0936	0.025	-0.0548	0.56
354:	13	3	10	10	13	12	3	9	10	12	299549.3460	0.1079	0.025	0.0623	0.48
355:	13	3	10	10	14	12	3	9	10	13	299549.3460	0.0201	0.025	0.0623	0.52
356:	13	3	10	10	12	12	3	9	10	11	299549.7090	0.0105	0.025	-0.0386	0.44
357:	13	3	10	10	15	12	3	9	10	14	299549.7090	-0.0774	0.025	-0.0386	0.56
358:	13	2	11	10	12	12	2	10	10	11	299782.6220	-0.0204	0.025	0.0105	0.22
359:	13	2	11	10	13	12	2	10	10	12	299782.6220	0.1850	0.025	0.0105	0.24
360:	13	2	11	10	14	12	2	10	10	13	299782.6220	0.0524	0.025	0.0105	0.26
361:	13	2	11	10	15	12	2	10	10	14	299782.6220	-0.1531	0.025	0.0105	0.28
362:	13	1	12	10	12	12	1	11	10	11	301462.6070	0.0712	0.025	0.0105	0.22
363:	13	1	12	10	13	12	1	11	10	12	301462.6070	0.1221	0.025	0.0105	0.24
364:	13	1	12	10	14	12	1	11	10	13	301462.6070	-0.0376	0.025	0.0105	0.26
365:	13	1	12	10	15	12	1	11	10	14	301462.6070	-0.0885	0.025	0.0105	0.28
366:	14	1	14	10	13	13	1	13	10	12	320299.9150	0.0582	0.025	0.0045	0.22
367:	14	1	14	10	14	13	1	13	10	13	320299.9150	0.0989	0.025	0.0045	0.24
368:	14	1	14	10	15	13	1	13	10	14	320299.9150	-0.0392	0.025	0.0045	0.26
369:	14	1	14	10	16	13	1	13	10	15	320299.9150	-0.0798	0.025	0.0045	0.28
370:	14	13	1	10	15	13	13	0	10	14	321958.4440	0.0054	0.025	0.0054	0.50
371:	14	13	2	10	15	13	13	1	10	14	321958.4440	0.0054	0.025	0.0054	0.50
372:	14	13	1	10	14	13	13	0	10	13	321959.4520	0.0365	0.025	0.0365	0.50
373:	14	13	2	10	14	13	13	1	10	13	321959.4520	0.0365	0.025	0.0365	0.50
374:	14	13	1	10	16	13	13	0	10	15	321965.3610	0.0079	0.025	0.0080	0.50
375:	14	13	2	10	16	13	13	1	10	15	321965.3610	0.0079	0.025	0.0080	0.50
376:	14	13	1	10	13	13	13	0	10	12	321966.3700	0.0389	0.025	0.0389	0.50
377:	14	13	2	10	13	13	13	1	10	12	321966.3700	0.0389	0.025	0.0389	0.50
378:	14	12	2	10	15	13	12	1	10	14	322047.8950	-0.0325	0.025	-0.0326	0.50
379:	14	12	3	10	15	13	12	2	10	14	322047.8950	-0.0325	0.025	-0.0326	0.50
380:	14	12	2	10	14	13	12	1	10	13	322048.7520	0.0132	0.025	0.0132	0.50
381:	14	12	3	10	14	13	12	2	10	13	322048.7520	0.0132	0.025	0.0132	0.50
382:	14	12	2	10	16	13	12	1	10	15	322053.7960	-0.0233	0.025	-0.0234	0.50
383:	14	12	3	10	16	13	12	2	10	15	322053.7960	-0.0233	0.025	-0.0234	0.50
384:	14	12	2	10	13	13	12	1	10	12	322054.6640	0.0328	0.025	0.0329	0.50
385:	14	12	3	10	13	13	12	2	10	12	322054.6640	0.0328	0.025	0.0329	0.50
386:	14	11	3	10	15	13	11	2	10	14	322130.4600	-0.0399	0.025	-0.0400	0.50
387:	14	11	4	10	15	13	11	3	10	14	322130.4600	-0.0399	0.025	-0.0400	0.50
388:	14	11	3	10	14	13	11	2	10	13	322131.1900	0.0313	0.025	0.0313	0.50
389:	14	11	4	10	14	13	11	3	10	13	322131.1900	0.0313	0.025	0.0313	0.50
390:	14	11	3	10	16	13	11	2	10	15	322135.4220	-0.0289	0.025	-0.0289	0.50
391:	14	11	4	10	16	13	11	3	10	15	322135.4220	-0.0289	0.025	-0.0289	0.50
392:	14	11	3	10	13	13	11	2	10	12	322136.1530	0.0433	0.025	0.0433	0.50

393:	14	11	4	10	13	13	11	3	10	12	322136.1530	0.0433	0.025	0.0433	0.50
394:	14	0	14	10	13	13	0	13	10	12	322172.4680	0.0835	0.025	0.0052	0.22
395:	14	0	14	10	14	13	0	13	10	13	322172.4680	0.0823	0.025	0.0052	0.24
396:	14	0	14	10	15	13	0	13	10	14	322172.4680	-0.0626	0.025	0.0052	0.26
397:	14	0	14	10	16	13	0	13	10	15	322172.4680	-0.0614	0.025	0.0052	0.28
398:	14	10	4	10	15	13	10	3	10	14	322206.1750	-0.0078	0.025	-0.0078	0.50
399:	14	10	5	10	15	13	10	4	10	14	322206.1750	-0.0078	0.025	-0.0078	0.50
400:	14	10	4	10	14	13	10	3	10	13	322206.6710	-0.0312	0.025	-0.0312	0.50
401:	14	10	5	10	14	13	10	4	10	13	322206.6710	-0.0312	0.025	-0.0312	0.50
402:	14	10	4	10	16	13	10	3	10	15	322210.2700	-0.0046	0.025	-0.0046	0.50
403:	14	10	5	10	16	13	10	4	10	15	322210.2700	-0.0046	0.025	-0.0046	0.50
404:	14	10	4	10	13	13	10	3	10	12	322210.7720	-0.0218	0.025	-0.0218	0.50
405:	14	10	5	10	13	13	10	4	10	12	322210.7720	-0.0218	0.025	-0.0218	0.50
406:	14	9	5	10	14	13	9	4	10	13	322275.2020	-0.2259	0.025	-0.0223	0.24
407:	14	9	5	10	15	13	9	4	10	14	322275.2020	0.1672	0.025	-0.0223	0.26
408:	14	9	6	10	14	13	9	5	10	13	322275.2020	-0.2259	0.025	-0.0223	0.24
409:	14	9	6	10	15	13	9	5	10	14	322275.2020	0.1672	0.025	-0.0223	0.26
410:	14	9	5	10	13	13	9	4	10	12	322278.4670	-0.2750	0.025	-0.0575	0.22
411:	14	9	5	10	16	13	9	4	10	15	322278.4670	0.1178	0.025	-0.0575	0.28
412:	14	9	6	10	13	13	9	5	10	12	322278.4670	-0.2750	0.025	-0.0575	0.22
413:	14	9	6	10	16	13	9	5	10	15	322278.4670	0.1178	0.025	-0.0575	0.28
414:	14	8	6	10	14	13	8	5	10	13	322337.3170	-0.1337	0.025	0.0115	0.24
415:	14	8	6	10	15	13	8	5	10	14	322337.3170	0.1466	0.025	0.0115	0.26
416:	14	8	7	10	14	13	8	6	10	13	322337.3170	-0.1337	0.025	0.0115	0.24
417:	14	8	7	10	15	13	8	6	10	14	322337.3170	0.1466	0.025	0.0115	0.26
418:	14	8	6	10	13	13	8	5	10	12	322339.9270	-0.1422	0.025	0.0129	0.22
419:	14	8	6	10	16	13	8	5	10	15	322339.9270	0.1377	0.025	0.0129	0.28
420:	14	8	7	10	13	13	8	6	10	12	322339.9270	-0.1422	0.025	0.0129	0.22
421:	14	8	7	10	16	13	8	6	10	15	322339.9270	0.1377	0.025	0.0129	0.28
422:	14	7	7	10	14	13	7	6	10	13	322392.8880	-0.1047	0.025	-0.0111	0.24
423:	14	7	7	10	15	13	7	6	10	14	322392.8880	0.0760	0.025	-0.0111	0.26
424:	14	7	8	10	14	13	7	7	10	13	322392.8880	-0.1047	0.025	-0.0111	0.24
425:	14	7	8	10	15	13	7	7	10	14	322392.8880	0.0760	0.025	-0.0111	0.26
426:	14	7	7	10	13	13	7	6	10	12	322394.8810	-0.1164	0.025	-0.0166	0.22
427:	14	7	7	10	16	13	7	6	10	15	322394.8810	0.0638	0.025	-0.0166	0.28
428:	14	7	8	10	13	13	7	7	10	12	322394.8810	-0.1164	0.025	-0.0166	0.22
429:	14	7	8	10	16	13	7	7	10	15	322394.8810	0.0638	0.025	-0.0166	0.28
430:	14	6	8	10	14	13	6	7	10	13	322442.4420	-0.0618	0.025	-0.0129	0.24
431:	14	6	8	10	15	13	6	7	10	14	322442.4420	0.0325	0.025	-0.0129	0.26
432:	14	6	9	10	14	13	6	8	10	13	322442.4420	-0.0618	0.025	-0.0129	0.24
433:	14	6	9	10	15	13	6	8	10	14	322442.4420	0.0325	0.025	-0.0129	0.26
434:	14	6	8	10	13	13	6	7	10	12	322443.9150	-0.0616	0.025	-0.0096	0.22
435:	14	6	8	10	16	13	6	7	10	15	322443.9150	0.0323	0.025	-0.0096	0.28
436:	14	6	9	10	13	13	6	8	10	12	322443.9150	-0.0616	0.025	-0.0096	0.22
437:	14	6	9	10	16	13	6	8	10	15	322443.9150	0.0323	0.025	-0.0096	0.28
438:	14	2	13	10	13	13	2	12	10	12	322482.2510	-0.0249	0.025	-0.0068	0.22
439:	14	2	13	10	14	13	2	12	10	13	322482.2510	0.1385	0.025	-0.0068	0.24
440:	14	2	13	10	15	13	2	12	10	14	322482.2510	0.0202	0.025	-0.0068	0.26
441:	14	2	13	10	16	13	2	12	10	15	322482.2510	-0.1432	0.025	-0.0068	0.28
442:	14	5	9	10	14	13	5	8	10	13	322486.9610	-0.0173	0.025	-0.0063	0.24
443:	14	5	9	10	15	13	5	8	10	14	322486.9610	0.0039	0.025	-0.0063	0.26
444:	14	5	10	10	14	13	5	9	10	13	322486.9610	-0.0172	0.025	-0.0063	0.24
445:	14	5	10	10	15	13	5	9	10	14	322486.9610	0.0040	0.025	-0.0063	0.26
446:	14	5	9	10	13	13	5	8	10	12	322488.0030	0.0017	0.025	0.0135	0.22
447:	14	5	9	10	16	13	5	8	10	15	322488.0030	0.0227	0.025	0.0135	0.28
448:	14	5	10	10	13	13	5	9	10	12	322488.0030	0.0019	0.025	0.0135	0.22
449:	14	5	10	10	16	13	5	9	10	15	322488.0030	0.0229	0.025	0.0135	0.28
450:	14	4	10	10	14	13	4	9	10	13	322528.9620	-0.0223	0.025	-0.0220	0.24
451:	14	4	10	10	15	13	4	9	10	14	322528.9620	-0.0608	0.025	-0.0220	0.26
452:	14	4	11	10	14	13	4	10	10	13	322528.9620	0.0181	0.025	-0.0220	0.24
453:	14	4	11	10	15	13	4	10	10	14	322528.9620	-0.0203	0.025	-0.0220	0.26
454:	14	4	10	10	13	13	4	9	10	12	322529.6920	0.0530	0.025	0.0518	0.22
455:	14	4	10	10	16	13	4	9	10	15	322529.6920	0.0142	0.025	0.0518	0.28
456:	14	4	11	10	13	13	4	10	10	12	322529.6920	0.0935	0.025	0.0518	0.22
457:	14	4	11	10	16	13	4	10	10	15	322529.6920	0.0547	0.025	0.0518	0.28
458:	14	3	12	10	13	13	3	11	10	12	322573.9230	-0.0897	0.025	0.0483	0.22
459:	14	3	12	10	14	13	3	11	10	13	322573.9230	0.2786	0.025	0.0483	0.24
460:	14	3	12	10	15	13	3	11	10	14	322573.9230	0.1935	0.025	0.0483	0.26
461:	14	3	12	10	16	13	3	11	10	15	322573.9230	-0.1749	0.025	0.0483	0.28
462:	14	3	11	10	13	13	3	10	10	12	322580.3010	-0.1120	0.025	0.0261	0.22
463:	14	3	11	10	14	13	3	10	10	13	322580.3010	0.2563	0.025	0.0261	0.24
464:	14	3	11	10	15	13	3	10	10	14	322580.3010	0.1712	0.025	0.0261	0.26
465:	14	3	11	10	16	13	3	10	10	15	322580.3010	-0.1972	0.025	0.0261	0.28
466:	14	2	12	10	13	13	2	11	10	12	322867.4800	-0.0170	0.025	0.0016	0.22
467:	14	2	12	10	14	13	2	11	10	13	322867.4800	0.1475	0.025	0.0016	0.24
468:	14	2	12	10	15	13	2	11	10	14	322867.4800	0.0292	0.025	0.0016	0.26
469:	14	2	12	10	16	13	2	11	10	15	322867.4800	-0.1354	0.025	0.0016	0.28
470:	14	1	13	10	13	13	1	12	10	12	324618.2210	0.0561	0.025	0.0021	0.22

```
 471: 14  1 13 10 14   13  1 12 10 13       324618.2210   0.0967  0.025   0.0021 0.24
 472: 14  1 13 10 15   13  1 12 10 14       324618.2210  -0.0417  0.025   0.0021 0.26
 473: 14  1 13 10 16   13  1 12 10 15       324618.2210  -0.0823  0.025   0.0021 0.28
-----------------------------------------------------------------------------------------=========
    3 Lines rejected from fit

PARAMETERS IN FIT (values truncated and Nlines statistics):

        1000000            A  /MHz        95426.0587(24)                     1
       -1001010            .  /MHz        95426.0587(24)         =  1.00000 *  1
        2000000            B  /MHz        11679.70195(18)                    2
       -2001010            .  /MHz        11679.70195(18)        =  1.00000 *  2
        3000000            C  /MHz        11370.06627(18)                    3
       -3001010            .  /MHz        11370.06627(18)        =  1.00000 *  3
          20000      Delta_J  /kHz           12.66567(43)                    4
         -21010            .  /kHz           12.66567(43)        =  1.00000 *  4
         110000     Delta_JK  /kHz          127.1323(17)                     5
        -111010            .  /kHz          127.1323(17)         =  1.00000 *  5
         200000      Delta_K  /kHz          959.74(19)                       6
        -201010            .  /kHz          959.74(19)           =  1.00000 *  6
        4010000      delta_J  /kHz            0.310662(41)                   7
       -4011010            .  /kHz            0.310662(41)       =  1.00000 *  7
        4100000      delta_K  /kHz           60.346(24)                      8
       -4101010            .  /kHz           60.346(24)          =  1.00000 *  8
          30000        Phi_J  /Hz           [-0.002427853123]                9
         -31010            .  /Hz           [-0.002427853123]    =  1.00000 *  9
         120000       Phi_JK  /Hz           [ 0.332733965]                  10
        -121010            .  /Hz           [ 0.332733965]       =  1.00000 * 10
         210000       Phi_KJ  /Hz           [ 2.649427203]                  11
        -211010            .  /Hz           [ 2.649427203]       =  1.00000 * 11
         300000        Phi_K  /Hz           [31.602872]                     12
        -301010            .  /Hz           [31.602872]          =  1.00000 * 12
        4020000        phi_J  /Hz           [ 0.000160615029]               13
       -4021010            .  /Hz           [ 0.000160615029]    =  1.00000 * 13
        4110000       phi_JK  /Hz           [ 0.267288418]                  14
       -4111010            .  /Hz           [ 0.267288418]       =  1.00000 * 14
        4200000        phi_K  /Hz           [23.8537]                       15
       -4201010            .  /Hz           [23.8537]            =  1.00000 * 15

    11001001010         X_aa  /MHz          -74.469(15)                     16
   -11003001010         X_cc  /MHz           74.469(15)          = -1.00000 * 16
    11002001010         X_bb  /MHz           37.324(24)                     17
   -11003001010         X_cc  /MHz          -37.324(24)          = -1.00000 * 17

 MICROWAVE AVG =       -0.016000 MHz, IR AVG =        0.00000
 MICROWAVE RMS =        0.148567 MHz, IR RMS =        0.00000
 END OF ITERATION  1 OLD, NEW RMS ERROR=        0.94172         0.94172

  distinct frequency lines in fit:   212
       distinct parameters of fit:    10
          lines rejected from fit:     3    (ERRTST =  3.00E+00)

MICROWAVE       lines fitted       lines    lines      RMS      RMS ERROR    J range   Ka range
freq. range
            total   dv=0 dv.ne.0   UNFITTD   e>900
v"= 0         30     30      0        2      0       0.388879     1.13912     1  34    0   5
27921    46406
v"=10        182    182      0        1      0       0.027943     0.90450     1  20    0  13
28537   324618
-----------------------------------------------------------------------------------------------
total:       212    212      0        3      0       0.148561     0.94126

NOTE: the RMS values above are for Nlines statistics, but the 'total' values may differ slightly
from
      those in the .FIT file since the o-c values for this evaluation are as rounded in the .FIT.


 PARAMETERS IN FIT WITH STANDARD ERRORS ON THOSE THAT ARE FITTED:
 (values rounded and degrees of freedom, Ndegf=Nlines-Nconst, statistics)

        1000000            A  /MHz      95426.0588(23)                       1
       -1001010            .  /MHz      95426.0588(23)           =  1.00000 *  1
        2000000            B  /MHz      11679.70196(17)                      2
       -2001010            .  /MHz      11679.70196(17)          =  1.00000 *  2
        3000000            C  /MHz      11370.06627(17)                      3
       -3001010            .  /MHz      11370.06627(17)          =  1.00000 *  3
          20000      Delta_J  /kHz         12.66567(41)                      4
         -21010            .  /kHz         12.66567(41)          =  1.00000 *  4
```

```
         110000     Delta_JK  /kHz        127.1323(16)                        5
        -111010            .  /kHz        127.1323(16)         =  1.00000 *  5
         200000      Delta_K  /kHz        959.74(19)                          6
        -201010            .  /kHz        959.74(19)           =  1.00000 *  6
        4010000      delta_J  /kHz          0.310662(40)                      7
       -4011010            .  /kHz          0.310662(40)       =  1.00000 *  7
        4100000      delta_K  /kHz         60.346(23)                         8
       -4101010            .  /kHz         60.346(23)          =  1.00000 *  8
          30000        Phi_J  /Hz           [-0.002427853123]                 9
         -31010            .  /Hz           [-0.002427853123]  =  1.00000 *  9
         120000       Phi_JK  /Hz           [ 0.332733965]                   10
        -121010            .  /Hz           [ 0.332733965]     =  1.00000 * 10
         210000       Phi_KJ  /Hz           [ 2.649427203]                   11
        -211010            .  /Hz           [ 2.649427203]     =  1.00000 * 11
         300000        Phi_K  /Hz           [31.602872]                      12
        -301010            .  /Hz           [31.602872]        =  1.00000 * 12
        4020000        phi_J  /Hz           [ 0.000160615029]                13
       -4021010            .  /Hz           [ 0.000160615029]  =  1.00000 * 13
        4110000       phi_JK  /Hz           [ 0.267288418]                   14
       -4111010            .  /Hz           [ 0.267288418]     =  1.00000 * 14
        4200000        phi_K  /Hz           [23.8537]                        15
       -4201010            .  /Hz           [23.8537]          =  1.00000 * 15

    11001001010         X_aa  /MHz        -74.469(14)                        16
   -11003001010         X_cc  /MHz         74.469(14)          = -1.00000 * 16
    11002001010         X_bb  /MHz         37.325(23)                        17
   -11003001010         X_cc  /MHz        -37.325(23)          = -1.00000 * 17

 CORRELATION COEFFICIENTS, C.ij:

           A        B        C        -Delta_J -Delta_J -Delta_K -delta_J -delta_K

A           1.0000
B           0.4479   1.0000
C           0.6306   0.6733   1.0000
-Delta_J   -0.4934  -0.6598  -0.6383   1.0000
-Delta_JK  -0.1529  -0.3326  -0.3791  -0.1437   1.0000
-Delta_K   -0.6293  -0.4689  -0.5327   0.9245  -0.3364   1.0000
-delta_J    0.3683  -0.1360   0.2348  -0.1271   0.1160  -0.2144   1.0000
-delta_K   -0.1129  -0.2590   0.1818   0.1289  -0.1445   0.1012  -0.4324   1.0000
X_aa       -0.0069  -0.0123  -0.0113   0.0074   0.0090   0.0046   0.0014  -0.0001
X_bb        0.0040   0.0245   0.0157  -0.0134  -0.0114  -0.0067  -0.0123   0.0016

           X_aa     X_bb

X_aa        1.0000
X_bb       -0.3569   1.0000

 Mean value of |C.ij|, i.ne.j =   0.2333
 Mean value of  C.ij,  i.ne.j =  -0.0611


 No correlations with absolute value greater than 0.9950


 Worst fitted lines (obs-calc/error):

    59:   -2.6        354:    2.5         50:   -2.5         36:   -2.4
   410:   -2.3        230:   -2.3        352:   -2.2          1:    2.2
   106:   -2.1         48:    2.1        454:    2.1        310:   -2.0
   458:    1.9        264:    1.9        350:    1.8         37:   -1.8
   224:    1.8        392:    1.7        220:    1.7         66:    1.7
    39:    1.6        386:   -1.6        376:    1.6        356:   -1.5
   284:    1.5        130:    1.5        372:    1.5        292:    1.4
   304:    1.4        268:    1.4        286:   -1.4        101:   -1.4
    95:   -1.4        266:   -1.3        384:    1.3        378:   -1.3
    27:   -1.3        388:    1.3        400:   -1.2        390:   -1.2
   205:   -1.1         49:   -1.1         19:    1.1         99:   -1.1
    15:    1.1          3:    1.1        168:    1.1        280:    1.1
   274:    1.1        178:    1.1

    59: 28  1 28  0 28   28  0 28  0 28       37983.7950  -0.0529  0.020
   354: 13  3 10 10 13   12  3  9 10 12      299549.3460   0.1079  0.025    0.0623 0.48
    50: 27  3 24  0 27   26  4 22  0 26       36580.5870  -0.0491  0.020
    36:  2  0  2  0  2    1  0  1  0  1       46097.8000  -0.4767  0.200
   410: 14  9  5 10 13   13  9  4 10 12      322278.4670  -0.2750  0.025   -0.0575 0.22
   230: 12  7  5 10 11   11  7  4 10 10      276368.7660  -0.2861  0.025   -0.0567 0.22
   352: 13  3 11 10 12   12  3 10 10 11      299545.2900  -0.0056  0.025   -0.0548 0.44
```

```
     1:  5  0  5 10  7    4  1  3 10  6        29771.6170  -0.1104  0.020   0.0432 0.50
   106:  4  0  4 10  3    3  0  3 10  3        92204.2860  -0.0212  0.010
    48: 23  4 19  0 23   24  3 21  0 24        33108.9480   0.0421  0.020
                                            _____________________________________
__________________________________________/ SPFIT output reformatted with PIFORM
```

Table S.2. Fit of the rotational transitions of $^{12}CHD_2{}^{37}Cl$ in PIFORM format

```
chd2cl -- 37cl                                        Tue Mar 18 09:41:13 2025

-------------------------------------------------------------------------------=========
                                             obs         o-c     error     blends     Notes
                                                                          o-c      wt
    / instead of : below denotes (o-c)>3*err
-------------------------------------------------------------------------------=========
!Mallinson-TableII
   1:  1  0  1 10  3    0  0  0 10  2        22673.8000   0.2004  0.090
!Mallinson-TableII
   2:  1  0  1 10  2    0  0  0 10  2        22659.2900   0.367 UNFITTD   22658.9229
!Mallinson-TableII
   3:  1  0  1 10  1    0  0  0 10  2        22685.6000   0.2563  0.090
!Mallinson-TableII
   4:  5  0  5 10  6    4  1  3 10  5        27737.9070   0.1022  0.161   0.2271 0.50
!Mallinson-TableII
   5:  5  0  5 10  5    4  1  3 10  4        27737.9070   0.3519  0.161   0.2271 0.50
!Mallinson-TableII
   6:  5  0  5 10  7    4  1  3 10  6        27739.7370  -0.2263  0.161  -0.1009 0.50
!Mallinson-TableII
   7:  5  0  5 10  4    4  1  3 10  3        27739.7370   0.0244  0.161  -0.1009 0.50
!Mallinson-TableII
   8:  9  2  8 10  8   10  1 10 10  9        33848.3030  -0.0142  0.110  -0.0247 0.50
!Mallinson-TableII
   9:  9  2  8 10 11   10  1 10 10 12        33848.3030  -0.0351  0.110  -0.0247 0.50
!Mallinson-TableII
  10:  9  2  8 10  9   10  1 10 10 10        33849.6880  -0.1428  0.110  -0.1535 0.50
!Mallinson-TableII
  11:  9  2  8 10 10   10  1 10 10 11        33849.6880  -0.1641  0.110  -0.1535 0.50
!Mallinson-TableV
  12:  1  0  1  0  1    0  0  0  0  0        22670.9400   0.277 UNFITTD   22670.6627
!Mallinson-TableV
  13:  5  0  5  0  5    4  1  3  0  4        27738.9920   0.1215  0.161
!Mallinson-TableV
  14:  9  2  8  0  9   10  1 10  0 10        33848.9870  -0.0884  0.110
!Mallinson-TableV
  15: 12  1 11  0 12   11  2  9  0 11        30739.6280   0.0268  0.050
!Mallinson-TableV
  16: 13  1 13  0 13   12  2 11  0 12        28664.1200  -0.1252  0.050
!Mallinson-TableV
  17: 16  3 13  0 16   17  2 15  0 17        32840.6690   0.0661  0.050
!Mallinson-TableV
  18: 16  3 14  0 16   17  2 16  0 17        35866.0620   0.231 UNFITTD   35865.8302
!Mallinson-TableV
  19: 20  2 18  0 20   19  3 16  0 19        36980.9920   0.0529  0.050
!Mallinson-TableV
  20: 20  2 19  0 20   19  3 17  0 19        31346.8190  -0.0075  0.050
!Mallinson-TableV
  21: 31  5 27  0 31   32  4 29  0 32        30534.0040  -0.260 UNFITTD   30534.2644
!Mallinson-TableV
  22: 31  5 26  0 31   32  4 28  0 32        30480.7990  -0.0034  0.050
!Mallinson-TableV
  23: 35  4 31  0 35   34  5 29  0 34        37719.5020  -0.0311  0.050
!Mallinson-TableV
  24: 35  4 32  0 35   34  5 30  0 34        37611.6570   0.0326  0.050
!Mallinson-TableV
  25: 13  1 12  0 13   13  1 13  0 13        27206.1010  -0.0081  0.020
!Mallinson-TableV
  26: 14  1 13  0 14   14  1 14  0 14        31384.0340  -0.0111  0.020
!Mallinson-TableV
  27: 15  1 14  0 15   15  1 15  0 15        35857.3680  -0.0214  0.020
!Mallinson-TableV
  28: 28  1 28  0 28   28  0 28  0 28        39159.6070  -0.069 UNFITTD   39159.6761
!Mallinson-TableV
  29: 29  1 29  0 29   29  0 29  0 29        37032.7810  -0.0433  0.020
!Mallinson-TableV
  30: 30  1 30  0 30   30  0 30  0 30        34956.2310  -0.0166  0.020
!Mallinson-TableV
  31: 30  2 28  0 30   30  2 29  0 30        26635.4680   0.0324  0.020
!Mallinson-TableV
  32: 31  1 31  0 31   31  0 31  0 31        32936.0400  -0.0158  0.020
!Mallinson-TableV
  33: 31  2 29  0 31   31  2 30  0 31        30044.1950   0.0147  0.020
!Mallinson-TableV
  34: 32  1 32  0 32   32  0 32  0 32        30977.5940   0.0175  0.020
```

```
!Mallinson-TableV
  35: 32  2 30  0 32   32  2 31  0 32        33731.3860  -0.0099  0.020
!Mallinson-TableV
  36: 33  1 33  0 33   33  0 33  0 33        29085.3650   0.0303  0.020
!Mallinson-TableV
  37: 33  2 31  0 33   33  2 32  0 33        37704.0680  -0.0254  0.020
  38:  4  1  4 10  4    3  1  3 10  3        90077.2440  -0.0064  0.010
  39:  4  1  4 10  5    3  1  3 10  4        90078.0080   0.0000  0.010
  40:  4  1  4 10  3    3  1  3 10  2        90078.7100  -0.0076  0.010
  41:  4  1  4 10  6    3  1  3 10  5        90079.4830   0.0067  0.010
  42:  4  0  4 10  5    3  0  3 10  5        90657.4950  -0.0021  0.010
  43:  4  3  1 10  4    3  3  0 10  4        90661.6450  -0.0078  0.010  -0.0039 0.50
  44:  4  3  2 10  4    3  3  1 10  4        90661.6450   0.0000  0.010  -0.0039 0.50
  45:  4  3  1 10  5    3  3  0 10  4        90663.4580   0.0016  0.010   0.0056 0.50
  46:  4  3  2 10  5    3  3  1 10  4        90663.4580   0.0095  0.010   0.0056 0.50
  47:  4  3  1 10  3    3  3  0 10  3        90665.0790  -0.0040  0.010  -0.0000 0.50
  48:  4  3  2 10  3    3  3  1 10  3        90665.0790   0.0039  0.010  -0.0000 0.50
  49:  4  3  1 10  4    3  3  0 10  3        90670.2220   0.0054  0.010   0.0094 0.50
  50:  4  3  2 10  4    3  3  1 10  3        90670.2220   0.0133  0.010   0.0094 0.50
  51:  4  0  4 10  4    3  0  3 10  3        90670.4580  -0.0124  0.010  -0.0125 0.50
  52:  4  0  4 10  3    3  0  3 10  2        90670.4580  -0.0125  0.010  -0.0125 0.50
  53:  4  2  3 10  5    3  2  2 10  4        90671.4130   0.0043  0.010   0.0030 0.50
  54:  4  2  3 10  5    3  2  2 10  5        90671.4130   0.0016  0.010   0.0030 0.50
  55:  4  0  4 10  6    3  0  3 10  5        90672.1740   0.0002  0.010   0.0001 0.50
  56:  4  0  4 10  5    3  0  3 10  4        90672.1740  -0.0000  0.010   0.0001 0.50
  57:  4  2  3 10  4    3  2  2 10  3        90673.4720   0.0035  0.010   0.0045 0.50
  58:  4  2  3 10  4    3  2  2 10  4        90673.4720   0.0053  0.010   0.0045 0.50
  59:  4  3  1 10  6    3  3  0 10  5        90676.6600  -0.0051  0.010  -0.0012 0.50
  60:  4  3  2 10  6    3  3  1 10  5        90676.6600   0.0027  0.010  -0.0012 0.50
  61:  4  2  3 10  6    3  2  2 10  5        90677.2970   0.0163  0.010  -0.0044 0.50
  62:  4  0  4 10  4    3  0  3 10  4        90677.2970  -0.0252  0.010  -0.0044 0.50
  63:  4  2  2 10  5    3  2  1 10  4        90679.3690  -0.0153  0.010   0.0065 0.25
  64:  4  2  3 10  3    3  2  2 10  2        90679.3690   0.0313  0.010   0.0065 0.25
  65:  4  2  2 10  5    3  2  1 10  5        90679.3690  -0.0174  0.010   0.0065 0.25
  66:  4  2  3 10  3    3  2  2 10  3        90679.3690   0.0273  0.010   0.0065 0.25
  67:  4  2  2 10  4    3  2  1 10  3        90681.4370  -0.0072  0.010  -0.0065 0.50
  68:  4  2  2 10  4    3  2  1 10  4        90681.4370  -0.0056  0.010  -0.0065 0.50
  69:  4  3  1 10  3    3  3  0 10  2        90683.4220  -0.0059  0.010  -0.0019 0.50
  70:  4  3  2 10  3    3  3  1 10  2        90683.4220   0.0020  0.010  -0.0019 0.50
  71:  4  0  4 10  3    3  0  3 10  3        90685.2450   0.0983  0.010  -0.0021 0.09
  72:  4  2  2 10  6    3  2  1 10  5        90685.2450  -0.0117  0.010  -0.0021 0.91
  73:  4  2  2 10  3    3  2  1 10  2        90687.3080  -0.0059  0.010  -0.0082 0.50
  74:  4  2  2 10  3    3  2  1 10  3        90687.3080  -0.0104  0.010  -0.0082 0.50
  75:  4  1  3 10  4    3  1  2 10  3        91274.2850   0.0011  0.010
  76:  4  1  3 10  5    3  1  2 10  4        91275.0590   0.0078  0.010
  77:  4  1  3 10  3    3  1  2 10  2        91275.7490  -0.0020  0.010
  78:  4  1  3 10  6    3  1  2 10  5        91276.5200   0.0006  0.010
  79: 11  1 11 10 11   10  1 10 10 10       247623.4850   0.1132  0.025  -0.0143 0.24
  80: 11  1 11 10 12   10  1 10 10 11       247623.4850  -0.0593  0.025  -0.0143 0.26
  81: 11  1 11 10 13   10  1 10 10 12       247623.4850  -0.1258  0.025  -0.0143 0.29
  82: 11  1 11 10 10   10  1 10 10  9       247623.4850   0.0468  0.025  -0.0143 0.22
  83: 11 10  1 10 13   10 10  0 10 12       249047.5800   0.0403  0.025   0.0404 0.50
  84: 11 10  2 10 13   10 10  1 10 12       249047.5800   0.0403  0.025   0.0404 0.50
  85: 11 10  1 10 10   10 10  0 10  9       249048.7660   0.0260  0.025   0.0261 0.50
  86: 11 10  2 10 10   10 10  1 10  9       249048.7660   0.0260  0.025   0.0261 0.50
  87: 11  9  2 10 12   10  9  1 10 11       249093.4950   0.0170  0.025   0.0171 0.50
  88: 11  9  3 10 12   10  9  2 10 11       249093.4950   0.0170  0.025   0.0171 0.50
  89: 11  9  2 10 11   10  9  1 10 10       249094.4310   0.0169  0.025   0.0169 0.50
  90: 11  9  3 10 11   10  9  2 10 10       249094.4310   0.0169  0.025   0.0169 0.50
  91: 11  9  2 10 13   10  9  1 10 12       249098.8850   0.0031  0.025   0.0031 0.50
  92: 11  9  3 10 13   10  9  2 10 12       249098.8850   0.0031  0.025   0.0031 0.50
  93: 11  9  2 10 10   10  9  1 10  9       249099.8220   0.0036  0.025   0.0036 0.50
  94: 11  9  3 10 10   10  9  2 10  9       249099.8220   0.0036  0.025   0.0036 0.50
  95: 11  0 11 10 11   10  0 10 10 10       249137.6080   0.0939  0.025  -0.0076 0.24
  96: 11  0 11 10 12   10  0 10 10 11       249137.6080  -0.0931  0.025  -0.0076 0.26
  97: 11  0 11 10 13   10  0 10 10 12       249137.6080  -0.0919  0.025  -0.0076 0.29
  98: 11  0 11 10 10   10  0 10 10  9       249137.6080   0.0951  0.025  -0.0076 0.22
  99: 11  8  3 10 12   10  8  2 10 11       249140.7960  -0.0123  0.025  -0.0124 0.50
 100: 11  8  4 10 12   10  8  3 10 11       249140.7960  -0.0123  0.025  -0.0124 0.50
 101: 11  8  3 10 11   10  8  2 10 10       249141.5210   0.0119  0.025   0.0120 0.50
 102: 11  8  4 10 11   10  8  3 10 10       249141.5210   0.0119  0.025   0.0120 0.50
 103: 11  8  3 10 13   10  8  2 10 12       249145.0630  -0.0153  0.025  -0.0154 0.50
 104: 11  8  4 10 13   10  8  3 10 12       249145.0630  -0.0153  0.025  -0.0154 0.50
 105: 11  8  3 10 10   10  8  2 10  9       249145.7920   0.0131  0.025   0.0131 0.50
 106: 11  8  4 10 10   10  8  3 10  9       249145.7920   0.0131  0.025   0.0131 0.50
 107: 11  7  4 10 12   10  7  3 10 11       249182.9300  -0.0245  0.025  -0.0246 0.50
 108: 11  7  5 10 12   10  7  4 10 11       249182.9300  -0.0245  0.025  -0.0246 0.50
 109: 11  7  4 10 11   10  7  3 10 10       249183.4730   0.0256  0.025   0.0257 0.50
```

110:	11	7	5	10	11	10	7	4	10	10	249183.4730	0.0256	0.025	0.0257 0.50
111:	11	7	4	10	13	10	7	3	10	12	249186.2180	-0.0058	0.025	-0.0059 0.50
112:	11	7	5	10	13	10	7	4	10	12	249186.2180	-0.0058	0.025	-0.0059 0.50
113:	11	7	4	10	10	10	7	3	10	9	249186.7550	0.0387	0.025	0.0387 0.50
114:	11	7	5	10	10	10	7	4	10	9	249186.7550	0.0387	0.025	0.0387 0.50
115:	11	6	5	10	11	10	6	4	10	10	249220.2510	-0.1765	0.025	-0.0131 0.24
116:	11	6	5	10	12	10	6	4	10	11	249220.2510	0.1359	0.025	-0.0131 0.26
117:	11	6	6	10	11	10	6	5	10	10	249220.2510	-0.1765	0.025	-0.0131 0.24
118:	11	6	6	10	12	10	6	5	10	11	249220.2510	0.1359	0.025	-0.0131 0.26
119:	11	6	5	10	13	10	6	4	10	12	249222.6210	0.1039	0.025	-0.0307 0.28
120:	11	6	5	10	10	10	6	4	10	9	249222.6210	-0.2081	0.025	-0.0307 0.22
121:	11	6	6	10	13	10	6	5	10	12	249222.6210	0.1039	0.025	-0.0307 0.28
122:	11	6	6	10	10	10	6	5	10	9	249222.6210	-0.2081	0.025	-0.0307 0.22
123:	11	5	6	10	11	10	5	5	10	10	249252.8010	-0.0947	0.025	-0.0111 0.24
124:	11	5	6	10	12	10	5	5	10	11	249252.8010	0.0651	0.025	-0.0111 0.26
125:	11	5	7	10	11	10	5	6	10	10	249252.8010	-0.0947	0.025	-0.0111 0.24
126:	11	5	7	10	12	10	5	6	10	11	249252.8010	0.0652	0.025	-0.0111 0.26
127:	11	5	6	10	13	10	5	5	10	12	249254.4570	0.0530	0.025	-0.0158 0.28
128:	11	5	6	10	10	10	5	5	10	9	249254.4570	-0.1065	0.025	-0.0158 0.22
129:	11	5	7	10	13	10	5	6	10	12	249254.4570	0.0530	0.025	-0.0158 0.28
130:	11	5	7	10	10	10	5	6	10	9	249254.4570	-0.1065	0.025	-0.0158 0.22
131:	11	2	10	10	11	10	2	9	10	10	249274.7130	0.2252	0.025	0.0196 0.24
132:	11	2	10	10	12	10	2	9	10	11	249274.7130	0.0937	0.025	0.0196 0.26
133:	11	2	10	10	13	10	2	9	10	12	249274.7130	-0.1729	0.025	0.0196 0.29
134:	11	2	10	10	10	10	2	9	10	9	249274.7130	-0.0414	0.025	0.0196 0.22
135:	11	4	7	10	11	10	4	6	10	10	249281.9880	-0.0174	0.025	0.0040 0.24
136:	11	4	7	10	12	10	4	6	10	11	249281.9880	0.0175	0.025	0.0040 0.26
137:	11	4	8	10	11	10	4	7	10	10	249281.9880	-0.0112	0.025	0.0040 0.24
138:	11	4	8	10	12	10	4	7	10	11	249281.9880	0.0238	0.025	0.0040 0.26
139:	11	4	7	10	13	10	4	6	10	12	249283.0540	0.0158	0.025	0.0040 0.28
140:	11	4	7	10	10	10	4	6	10	9	249283.0540	-0.0188	0.025	0.0040 0.22
141:	11	4	8	10	13	10	4	7	10	12	249283.0540	0.0221	0.025	0.0040 0.28
142:	11	4	8	10	10	10	4	7	10	9	249283.0540	-0.0126	0.025	0.0040 0.22
143:	11	3	9	10	11	10	3	8	10	10	249310.7180	0.0318	0.025	-0.0006 0.48
144:	11	3	9	10	12	10	3	8	10	11	249310.7180	-0.0302	0.025	-0.0006 0.52
145:	11	3	9	10	13	10	3	8	10	12	249311.3590	0.0101	0.025	0.0370 0.57
146:	11	3	9	10	10	10	3	8	10	9	249311.3590	0.0723	0.025	0.0370 0.43
147:	11	3	8	10	11	10	3	7	10	10	249312.4080	0.0197	0.025	-0.0128 0.48
148:	11	3	8	10	12	10	3	7	10	11	249312.4080	-0.0423	0.025	-0.0128 0.52
149:	11	3	8	10	13	10	3	7	10	12	249313.0540	0.0029	0.025	0.0298 0.57
150:	11	3	8	10	10	10	3	7	10	9	249313.0540	0.0651	0.025	0.0298 0.43
151:	11	2	9	10	11	10	2	8	10	10	249449.5300	0.1926	0.025	-0.0136 0.24
152:	11	2	9	10	12	10	2	8	10	11	249449.5300	0.0612	0.025	-0.0136 0.26
153:	11	2	9	10	13	10	2	8	10	12	249449.5300	-0.2067	0.025	-0.0136 0.29
154:	11	2	9	10	10	10	2	8	10	9	249449.5300	-0.0752	0.025	-0.0136 0.22
155:	11	1	10	10	11	10	1	9	10	10	250910.9680	0.1498	0.025	0.0217 0.24
156:	11	1	10	10	12	10	1	9	10	11	250910.9680	-0.0238	0.025	0.0217 0.26
157:	11	1	10	10	13	10	1	9	10	12	250910.9680	-0.0902	0.025	0.0217 0.29
158:	11	1	10	10	10	10	1	9	10	9	250910.9680	0.0834	0.025	0.0217 0.22
159:	12	1	12	10	11	11	1	11	10	10	270112.7690	0.0360	0.025	-0.0179 0.22
160:	12	1	12	10	12	11	1	11	10	11	270112.7690	0.0870	0.025	-0.0179 0.24
161:	12	1	12	10	13	11	1	11	10	12	270112.7690	-0.0592	0.025	-0.0179 0.26
162:	12	1	12	10	14	11	1	11	10	13	270112.7690	-0.1102	0.025	-0.0179 0.28
163:	12	10	2	10	13	11	10	1	10	12	271669.1440	0.0103	0.025	0.0103 0.50
164:	12	10	3	10	13	11	10	2	10	12	271669.1440	0.0103	0.025	0.0103 0.50
165:	12	10	2	10	12	11	10	1	10	11	271669.9610	0.0085	0.025	0.0086 0.50
166:	12	10	3	10	12	11	10	2	10	11	271669.9610	0.0085	0.025	0.0086 0.50
167:	12	10	2	10	14	11	10	1	10	13	271674.2690	0.0033	0.025	0.0034 0.50
168:	12	10	3	10	14	11	10	2	10	13	271674.2690	0.0033	0.025	0.0034 0.50
169:	12	10	2	10	11	11	10	1	10	10	271675.0940	0.0092	0.025	0.0093 0.50
170:	12	10	3	10	11	11	10	2	10	10	271675.0940	0.0092	0.025	0.0093 0.50
171:	12	9	3	10	13	11	9	2	10	12	271726.3550	-0.0061	0.025	-0.0062 0.50
172:	12	9	4	10	13	11	9	3	10	12	271726.3550	-0.0061	0.025	-0.0062 0.50
173:	12	9	3	10	12	11	9	2	10	11	271727.0260	0.0312	0.025	0.0312 0.50
174:	12	9	4	10	12	11	9	3	10	11	271727.0260	0.0312	0.025	0.0312 0.50
175:	12	9	3	10	14	11	9	2	10	13	271730.5200	0.0018	0.025	0.0018 0.50
176:	12	9	4	10	14	11	9	3	10	13	271730.5200	0.0018	0.025	0.0018 0.50
177:	12	9	3	10	11	11	9	2	10	10	271731.1970	0.0452	0.025	0.0452 0.50
178:	12	9	4	10	11	11	9	3	10	10	271731.1970	0.0452	0.025	0.0452 0.50
179:	12	0	12	10	11	11	0	11	10	10	271736.6760	0.0922	0.025	0.0067 0.22
180:	12	0	12	10	12	11	0	11	10	11	271736.6760	0.0909	0.025	0.0067 0.24
181:	12	0	12	10	13	11	0	11	10	12	271736.6760	-0.0655	0.025	0.0067 0.26
182:	12	0	12	10	14	11	0	11	10	13	271736.6760	-0.0642	0.025	0.0067 0.28
183:	12	8	4	10	13	11	8	3	10	12	271777.8930	0.0010	0.025	0.0010 0.50
184:	12	8	5	10	13	11	8	4	10	12	271777.8930	0.0010	0.025	0.0010 0.50
185:	12	8	4	10	12	11	8	3	10	11	271778.3900	0.0301	0.025	0.0301 0.50
186:	12	8	5	10	12	11	8	4	10	11	271778.3900	0.0301	0.025	0.0301 0.50
187:	12	8	4	10	14	11	8	3	10	13	271781.1780	0.0013	0.025	0.0014 0.50

188:	12	8	5	10	14	11	8	4	10	13	271781.1780	0.0013	0.025	0.0014	0.50
189:	12	8	4	10	11	11	8	3	10	10	271781.6700	0.0256	0.025	0.0257	0.50
190:	12	8	5	10	11	11	8	4	10	10	271781.6700	0.0256	0.025	0.0257	0.50
191:	12	7	5	10	12	11	7	4	10	11	271823.9890	-0.1846	0.025	-0.0170	0.24
192:	12	7	5	10	13	11	7	4	10	12	271823.9890	0.1370	0.025	-0.0170	0.26
193:	12	7	6	10	12	11	7	5	10	11	271823.9890	-0.1846	0.025	-0.0170	0.24
194:	12	7	6	10	13	11	7	5	10	12	271823.9890	0.1370	0.025	-0.0170	0.26
195:	12	7	5	10	11	11	7	4	10	10	271826.4750	-0.2132	0.025	-0.0323	0.22
196:	12	7	5	10	14	11	7	4	10	13	271826.4750	0.1081	0.025	-0.0323	0.28
197:	12	7	6	10	11	11	7	5	10	10	271826.4750	-0.2132	0.025	-0.0323	0.22
198:	12	7	6	10	14	11	7	5	10	13	271826.4750	0.1081	0.025	-0.0323	0.28
199:	12	6	6	10	12	11	6	5	10	11	271864.5820	-0.1143	0.025	-0.0128	0.24
200:	12	6	6	10	13	11	6	5	10	12	271864.5820	0.0805	0.025	-0.0128	0.26
201:	12	6	7	10	12	11	6	6	10	11	271864.5820	-0.1143	0.025	-0.0128	0.24
202:	12	6	7	10	13	11	6	6	10	12	271864.5820	0.0805	0.025	-0.0128	0.26
203:	12	6	6	10	11	11	6	5	10	10	271866.4150	-0.1287	0.025	-0.0192	0.22
204:	12	6	6	10	14	11	6	5	10	13	271866.4150	0.0657	0.025	-0.0192	0.28
205:	12	6	7	10	11	11	6	6	10	10	271866.4150	-0.1287	0.025	-0.0192	0.22
206:	12	6	7	10	14	11	6	6	10	13	271866.4150	0.0657	0.025	-0.0192	0.28
207:	12	5	7	10	12	11	5	6	10	11	271900.4630	-0.0461	0.025	-0.0006	0.24
208:	12	5	7	10	13	11	5	6	10	12	271900.4630	0.0413	0.025	-0.0006	0.26
209:	12	5	8	10	12	11	5	7	10	11	271900.4630	-0.0461	0.025	-0.0006	0.24
210:	12	5	8	10	13	11	5	7	10	12	271900.4630	0.0414	0.025	-0.0006	0.26
211:	12	5	7	10	11	11	5	6	10	10	271901.7390	-0.0531	0.025	-0.0040	0.22
212:	12	5	7	10	14	11	5	6	10	13	271901.7390	0.0341	0.025	-0.0040	0.28
213:	12	5	8	10	11	11	5	7	10	10	271901.7390	-0.0530	0.025	-0.0040	0.22
214:	12	5	8	10	14	11	5	7	10	13	271901.7390	0.0341	0.025	-0.0040	0.28
215:	12	2	11	10	11	11	2	10	10	10	271916.4490	-0.0465	0.025	-0.0081	0.22
216:	12	2	11	10	12	11	2	10	10	11	271916.4490	0.1584	0.025	-0.0081	0.24
217:	12	2	11	10	13	11	2	10	10	12	271916.4490	0.0410	0.025	-0.0081	0.26
218:	12	2	11	10	14	11	2	10	10	13	271916.4490	-0.1640	0.025	-0.0081	0.28
219:	12	4	8	10	12	11	4	7	10	11	271933.1090	-0.0041	0.025	0.0015	0.24
220:	12	4	8	10	13	11	4	7	10	12	271933.1090	-0.0044	0.025	0.0015	0.26
221:	12	4	9	10	12	11	4	8	10	11	271933.1090	0.0074	0.025	0.0015	0.24
222:	12	4	9	10	13	11	4	8	10	12	271933.1090	0.0072	0.025	0.0015	0.26
223:	12	4	8	10	11	11	4	7	10	10	271933.9430	0.0087	0.025	0.0143	0.22
224:	12	4	8	10	14	11	4	7	10	13	271933.9430	0.0082	0.025	0.0143	0.28
225:	12	4	9	10	11	11	4	8	10	10	271933.9430	0.0203	0.025	0.0143	0.22
226:	12	4	9	10	14	11	4	8	10	13	271933.9430	0.0198	0.025	0.0143	0.28
227:	12	3	10	10	12	11	3	9	10	11	271966.1240	0.0338	0.025	-0.0019	0.48
228:	12	3	10	10	13	11	3	9	10	12	271966.1240	-0.0347	0.025	-0.0019	0.52
229:	12	3	10	10	11	11	3	9	10	10	271966.6150	0.0628	0.025	0.0242	0.44
230:	12	3	10	10	14	11	3	9	10	13	271966.6150	-0.0058	0.025	0.0242	0.56
231:	12	3	9	10	12	11	3	8	10	11	271968.7610	0.0241	0.025	-0.0116	0.48
232:	12	3	9	10	13	11	3	8	10	12	271968.7610	-0.0444	0.025	-0.0116	0.52
233:	12	3	9	10	11	11	3	8	10	10	271969.2560	0.0571	0.025	0.0185	0.44
234:	12	3	9	10	14	11	3	8	10	13	271969.2560	-0.0115	0.025	0.0185	0.56
235:	12	2	10	10	11	11	2	9	10	10	272143.4630	-0.0456	0.025	-0.0065	0.22
236:	12	2	10	10	12	11	2	9	10	11	272143.4630	0.1606	0.025	-0.0065	0.24
237:	12	2	10	10	13	11	2	9	10	12	272143.4630	0.0433	0.025	-0.0065	0.26
238:	12	2	10	10	14	11	2	9	10	13	272143.4630	-0.1630	0.025	-0.0065	0.28
239:	12	1	11	10	11	11	1	10	10	10	273697.6270	0.0318	0.025	-0.0225	0.22
240:	12	1	11	10	12	11	1	10	10	11	273697.6270	0.0828	0.025	-0.0225	0.24
241:	12	1	11	10	13	11	1	10	10	12	273697.6270	-0.0643	0.025	-0.0225	0.26
242:	12	1	11	10	14	11	1	10	10	13	273697.6270	-0.1153	0.025	-0.0225	0.28
243:	13	1	13	10	12	12	1	12	10	11	292596.4190	0.0284	0.025	-0.0192	0.22
244:	13	1	13	10	13	12	1	12	10	12	292596.4190	0.0684	0.025	-0.0192	0.24
245:	13	1	13	10	14	12	1	12	10	13	292596.4190	-0.0570	0.025	-0.0192	0.26
246:	13	1	13	10	15	12	1	12	10	14	292596.4190	-0.0970	0.025	-0.0192	0.28
247:	13	12	1	10	14	12	12	0	10	13	294151.2070	-0.0409	0.025	-0.0409	0.50
248:	13	12	2	10	14	12	12	1	10	13	294151.2070	-0.0409	0.025	-0.0409	0.50
249:	13	12	1	10	13	12	12	0	10	12	294152.1320	0.0007	0.025	0.0007	0.50
250:	13	12	2	10	13	12	12	1	10	12	294152.1320	0.0007	0.025	0.0007	0.50
251:	13	12	1	10	15	12	12	0	10	14	294156.9840	-0.0701	0.025	-0.0702	0.50
252:	13	12	2	10	15	12	12	1	10	14	294156.9840	-0.0701	0.025	-0.0702	0.50
253:	13	12	1	10	12	12	12	0	10	11	294157.8990	-0.0393	0.025	-0.0393	0.50
254:	13	12	2	10	12	12	12	1	10	11	294157.8990	-0.0393	0.025	-0.0393	0.50
255:	13	11	2	10	14	12	11	1	10	13	294225.5820	-0.0104	0.025	-0.0104	0.50
256:	13	11	3	10	14	12	11	2	10	13	294225.5820	-0.0104	0.025	-0.0104	0.50
257:	13	11	2	10	13	12	11	1	10	12	294226.3620	0.0483	0.025	0.0484	0.50
258:	13	11	3	10	13	12	11	2	10	12	294226.3620	0.0483	0.025	0.0484	0.50
259:	13	11	2	10	15	12	11	1	10	14	294230.4410	-0.0304	0.025	-0.0304	0.50
260:	13	11	3	10	15	12	11	2	10	14	294230.4410	-0.0304	0.025	-0.0304	0.50
261:	13	11	2	10	12	12	11	1	10	11	294231.2170	0.0240	0.025	0.0240	0.50
262:	13	11	3	10	12	12	11	2	10	11	294231.2170	0.0240	0.025	0.0240	0.50
263:	13	10	3	10	14	12	10	2	10	13	294293.6790	-0.0257	0.025	-0.0258	0.50
264:	13	10	4	10	14	12	10	3	10	13	294293.6790	-0.0257	0.025	-0.0258	0.50
265:	13	10	3	10	13	12	10	2	10	12	294294.3150	0.0371	0.025	0.0371	0.50

266:	13	10	4	10	13	12	10	3	10	12	294294.3150	0.0371	0.025	0.0371	0.50
267:	13	10	3	10	15	12	10	2	10	14	294297.7310	-0.0061	0.025	-0.0061	0.50
268:	13	10	4	10	15	12	10	3	10	14	294297.7310	-0.0061	0.025	-0.0061	0.50
269:	13	10	3	10	12	12	10	2	10	11	294298.3620	0.0517	0.025	0.0517	0.50
270:	13	10	4	10	12	12	10	3	10	11	294298.3620	0.0517	0.025	0.0517	0.50
271:	13	0	13	10	12	12	0	12	10	11	294322.8750	0.0503	0.025	-0.0220	0.22
272:	13	0	13	10	13	12	0	12	10	12	294322.8750	0.0489	0.025	-0.0220	0.24
273:	13	0	13	10	14	12	0	12	10	13	294322.8750	-0.0840	0.025	-0.0220	0.26
274:	13	0	13	10	15	12	0	12	10	14	294322.8750	-0.0826	0.025	-0.0220	0.28
275:	13	9	4	10	14	12	9	3	10	13	294355.6680	0.0433	0.025	0.0433	0.50
276:	13	9	5	10	14	12	9	4	10	13	294355.6680	0.0433	0.025	0.0433	0.50
277:	13	9	4	10	13	12	9	3	10	12	294356.0180	-0.0458	0.025	-0.0458	0.50
278:	13	9	5	10	13	12	9	4	10	12	294356.0180	-0.0458	0.025	-0.0458	0.50
279:	13	9	4	10	15	12	9	3	10	14	294358.9240	0.0329	0.025	0.0330	0.50
280:	13	9	5	10	15	12	9	4	10	14	294358.9240	0.0329	0.025	0.0330	0.50
281:	13	9	4	10	12	12	9	3	10	11	294359.2820	-0.0479	0.025	-0.0479	0.50
282:	13	9	5	10	12	12	9	4	10	11	294359.2820	-0.0479	0.025	-0.0479	0.50
283:	13	8	5	10	13	12	8	4	10	12	294411.6050	-0.1483	0.025	0.0174	0.24
284:	13	8	5	10	14	12	8	4	10	13	294411.6050	0.1707	0.025	0.0174	0.26
285:	13	8	6	10	13	12	8	5	10	12	294411.6050	-0.1483	0.025	0.0174	0.24
286:	13	8	6	10	14	12	8	5	10	13	294411.6050	0.1707	0.025	0.0174	0.26
287:	13	8	5	10	12	12	8	4	10	11	294414.1710	-0.1629	0.025	0.0150	0.22
288:	13	8	5	10	15	12	8	4	10	14	294414.1710	0.1558	0.025	0.0150	0.28
289:	13	8	6	10	12	12	8	5	10	11	294414.1710	-0.1629	0.025	0.0150	0.22
290:	13	8	6	10	15	12	8	5	10	14	294414.1710	0.1558	0.025	0.0150	0.28
291:	13	7	6	10	13	12	7	5	10	12	294461.4130	-0.0965	0.025	0.0143	0.24
292:	13	7	6	10	14	12	7	5	10	13	294461.4130	0.1167	0.025	0.0143	0.26
293:	13	7	7	10	13	12	7	6	10	12	294461.4130	-0.0965	0.025	0.0143	0.24
294:	13	7	7	10	14	12	7	6	10	13	294461.4130	0.1167	0.025	0.0143	0.26
295:	13	7	6	10	12	12	7	5	10	11	294463.3710	-0.1142	0.025	0.0046	0.22
296:	13	7	6	10	15	12	7	5	10	14	294463.3710	0.0987	0.025	0.0046	0.28
297:	13	7	7	10	12	12	7	6	10	11	294463.3710	-0.1142	0.025	0.0046	0.22
298:	13	7	7	10	15	12	7	6	10	14	294463.3710	0.0987	0.025	0.0046	0.28
299:	13	6	7	10	13	12	6	6	10	12	294505.5950	-0.0705	0.025	-0.0074	0.24
300:	13	6	7	10	14	12	6	6	10	13	294505.5950	0.0509	0.025	-0.0074	0.26
301:	13	6	8	10	13	12	6	7	10	12	294505.5950	-0.0705	0.025	-0.0074	0.24
302:	13	6	8	10	14	12	6	7	10	13	294505.5950	0.0509	0.025	-0.0074	0.26
303:	13	6	7	10	12	12	6	6	10	11	294507.0400	-0.0770	0.025	-0.0094	0.22
304:	13	6	7	10	15	12	6	6	10	14	294507.0400	0.0441	0.025	-0.0094	0.28
305:	13	6	8	10	12	12	6	7	10	11	294507.0400	-0.0770	0.025	-0.0094	0.22
306:	13	6	8	10	15	12	6	7	10	14	294507.0400	0.0441	0.025	-0.0094	0.28
307:	13	5	8	10	13	12	5	7	10	12	294544.9230	-0.0393	0.025	-0.0166	0.24
308:	13	5	8	10	14	12	5	7	10	13	294544.9230	0.0044	0.025	-0.0166	0.26
309:	13	5	9	10	13	12	5	8	10	12	294544.9230	-0.0392	0.025	-0.0166	0.24
310:	13	5	9	10	14	12	5	8	10	13	294544.9230	0.0044	0.025	-0.0166	0.26
311:	13	5	8	10	12	12	5	7	10	11	294545.9370	-0.0333	0.025	-0.0090	0.22
312:	13	5	8	10	15	12	5	7	10	14	294545.9370	0.0101	0.025	-0.0090	0.28
313:	13	5	9	10	12	12	5	8	10	11	294545.9370	-0.0333	0.025	-0.0090	0.22
314:	13	5	9	10	15	12	5	8	10	14	294545.9370	0.0102	0.025	-0.0090	0.28
315:	13	2	12	10	12	12	2	11	10	11	294553.1030	-0.0389	0.025	-0.0150	0.22
316:	13	2	12	10	13	12	2	11	10	12	294553.1030	0.1220	0.025	-0.0150	0.24
317:	13	2	12	10	14	12	2	11	10	13	294553.1030	0.0174	0.025	-0.0150	0.26
318:	13	2	12	10	15	12	2	11	10	14	294553.1030	-0.1436	0.025	-0.0150	0.28
319:	13	4	9	10	13	12	4	8	10	12	294581.2720	-0.0409	0.025	-0.0408	0.24
320:	13	4	9	10	14	12	4	8	10	13	294581.2720	-0.0607	0.025	-0.0408	0.26
321:	13	4	10	10	13	12	4	9	10	12	294581.2720	-0.0202	0.025	-0.0408	0.24
322:	13	4	10	10	14	12	4	9	10	13	294581.2720	-0.0400	0.025	-0.0408	0.26
323:	13	4	9	10	12	12	4	8	10	11	294581.9810	0.0228	0.025	0.0221	0.22
324:	13	4	9	10	15	12	4	8	10	14	294581.9810	0.0029	0.025	0.0221	0.28
325:	13	4	10	10	12	12	4	9	10	11	294581.9810	0.0436	0.025	0.0221	0.22
326:	13	4	10	10	15	12	4	9	10	14	294581.9810	0.0236	0.025	0.0221	0.28
327:	13	3	11	10	12	12	3	10	10	11	294619.1750	-0.1522	0.025	-0.0087	0.22
328:	13	3	11	10	13	12	3	10	10	12	294619.1750	0.2107	0.025	-0.0087	0.24
329:	13	3	11	10	14	12	3	10	10	13	294619.1750	0.1415	0.025	-0.0087	0.26
330:	13	3	11	10	15	12	3	10	10	14	294619.1750	-0.2216	0.025	-0.0087	0.28
331:	13	3	10	10	12	12	3	9	10	11	294623.1320	-0.1633	0.025	-0.0197	0.22
332:	13	3	10	10	13	12	3	9	10	12	294623.1320	0.1997	0.025	-0.0197	0.24
333:	13	3	10	10	14	12	3	9	10	13	294623.1320	0.1305	0.025	-0.0197	0.26
334:	13	3	10	10	15	12	3	9	10	14	294623.1320	-0.2326	0.025	-0.0197	0.28
335:	13	2	11	10	12	12	2	10	10	11	294841.5810	-0.0226	0.025	0.0020	0.22
336:	13	2	11	10	13	12	2	10	10	12	294841.5810	0.1398	0.025	0.0020	0.24
337:	13	2	11	10	14	12	2	10	10	13	294841.5810	0.0352	0.025	0.0020	0.26
338:	13	2	11	10	15	12	2	10	10	14	294841.5810	-0.1272	0.025	0.0020	0.28
339:	13	1	12	10	12	12	1	11	10	11	296478.1750	0.0537	0.025	0.0056	0.22
340:	13	1	12	10	13	12	1	11	10	12	296478.1750	0.0936	0.025	0.0056	0.24
341:	13	1	12	10	14	12	1	11	10	13	296478.1750	-0.0325	0.025	0.0056	0.26
342:	13	1	12	10	15	12	1	11	10	14	296478.1750	-0.0724	0.025	0.0056	0.28
343:	14	1	14	10	13	13	1	13	10	12	315074.0060	0.0281	0.025	-0.0142	0.22

344:	14	1	14	10	14	13	1	13	10	13	315074.0060	0.0600	0.025	-0.0142	0.24
345:	14	1	14	10	15	13	1	13	10	14	315074.0060	-0.0486	0.025	-0.0142	0.26
346:	14	1	14	10	16	13	1	13	10	15	315074.0060	-0.0805	0.025	-0.0142	0.28
347:	14	11	3	10	15	13	11	2	10	14	316841.1010	0.0030	0.025	0.0030	0.50
348:	14	11	4	10	15	13	11	3	10	14	316841.1010	0.0030	0.025	0.0030	0.50
349:	14	11	3	10	14	13	11	2	10	13	316841.6140	-0.0032	0.025	-0.0033	0.50
350:	14	11	4	10	14	13	11	3	10	13	316841.6140	-0.0032	0.025	-0.0033	0.50
351:	14	11	3	10	16	13	11	2	10	15	316845.0070	0.0057	0.025	0.0057	0.50
352:	14	11	4	10	16	13	11	3	10	15	316845.0070	0.0057	0.025	0.0057	0.50
353:	14	11	3	10	13	13	11	2	10	12	316845.5110	-0.0096	0.025	-0.0096	0.50
354:	14	11	4	10	13	13	11	3	10	12	316845.5110	-0.0096	0.025	-0.0096	0.50
355:	14	0	14	10	13	13	0	13	10	12	316895.3230	0.0516	0.025	-0.0104	0.22
356:	14	0	14	10	14	13	0	13	10	13	316895.3230	0.0501	0.025	-0.0104	0.24
357:	14	0	14	10	15	13	0	13	10	14	316895.3230	-0.0642	0.025	-0.0104	0.26
358:	14	0	14	10	16	13	0	13	10	15	316895.3230	-0.0627	0.025	-0.0104	0.28
359:	14	10	4	10	14	13	10	3	10	13	316914.5840	-0.2170	0.025	-0.0049	0.24
360:	14	10	4	10	15	13	10	3	10	14	316914.5840	0.1924	0.025	-0.0049	0.26
361:	14	10	5	10	14	13	10	4	10	13	316914.5840	-0.2170	0.025	-0.0049	0.24
362:	14	10	5	10	15	13	10	4	10	14	316914.5840	0.1924	0.025	-0.0049	0.26
363:	14	10	4	10	13	13	10	3	10	12	316917.7810	-0.2458	0.025	-0.0192	0.22
364:	14	10	4	10	16	13	10	3	10	15	316917.7810	0.1634	0.025	-0.0192	0.28
365:	14	10	5	10	13	13	10	4	10	12	316917.7810	-0.2458	0.025	-0.0192	0.22
366:	14	10	5	10	16	13	10	4	10	15	316917.7810	0.1634	0.025	-0.0192	0.28
367:	14	9	5	10	14	13	9	4	10	13	316981.1940	-0.1781	0.025	-0.0176	0.24
368:	14	9	5	10	15	13	9	4	10	14	316981.1940	0.1318	0.025	-0.0176	0.26
369:	14	9	6	10	14	13	9	5	10	13	316981.1940	-0.1781	0.025	-0.0176	0.24
370:	14	9	6	10	15	13	9	5	10	14	316981.1940	0.1318	0.025	-0.0176	0.26
371:	14	9	5	10	13	13	9	4	10	12	316983.7890	-0.1960	0.025	-0.0245	0.22
372:	14	9	5	10	16	13	9	4	10	15	316983.7890	0.1137	0.025	-0.0245	0.28
373:	14	9	6	10	13	13	9	5	10	12	316983.7890	-0.1960	0.025	-0.0245	0.22
374:	14	9	6	10	16	13	9	5	10	15	316983.7890	0.1137	0.025	-0.0245	0.28
375:	14	8	6	10	14	13	8	5	10	13	317041.3360	-0.1002	0.025	0.0143	0.24
376:	14	8	6	10	15	13	8	5	10	14	317041.3360	0.1207	0.025	0.0143	0.26
377:	14	8	7	10	14	13	8	6	10	13	317041.3360	-0.1002	0.025	0.0143	0.24
378:	14	8	7	10	15	13	8	6	10	14	317041.3360	0.1207	0.025	0.0143	0.26
379:	14	8	6	10	13	13	8	5	10	12	317043.3870	-0.1137	0.025	0.0086	0.22
380:	14	8	6	10	16	13	8	5	10	15	317043.3870	0.1070	0.025	0.0086	0.28
381:	14	8	7	10	13	13	8	6	10	12	317043.3870	-0.1137	0.025	0.0086	0.22
382:	14	8	7	10	16	13	8	6	10	15	317043.3870	0.1070	0.025	0.0086	0.28
383:	14	7	7	10	14	13	7	6	10	13	317095.1130	-0.0863	0.025	-0.0125	0.24
384:	14	7	7	10	15	13	7	6	10	14	317095.1130	0.0561	0.025	-0.0125	0.26
385:	14	7	8	10	14	13	7	7	10	13	317095.1130	-0.0863	0.025	-0.0125	0.24
386:	14	7	8	10	15	13	7	7	10	14	317095.1130	0.0561	0.025	-0.0125	0.26
387:	14	7	7	10	13	13	7	6	10	12	317096.6890	-0.0909	0.025	-0.0121	0.22
388:	14	7	7	10	16	13	7	6	10	15	317096.6890	0.0512	0.025	-0.0121	0.28
389:	14	7	8	10	13	13	7	7	10	12	317096.6890	-0.0909	0.025	-0.0121	0.22
390:	14	7	8	10	16	13	7	7	10	15	317096.6890	0.0512	0.025	-0.0121	0.28
391:	14	6	8	10	14	13	6	7	10	13	317143.0490	-0.0311	0.025	0.0074	0.24
392:	14	6	8	10	15	13	6	7	10	14	317143.0490	0.0432	0.025	0.0074	0.26
393:	14	6	9	10	14	13	6	8	10	13	317143.0490	-0.0311	0.025	0.0074	0.24
394:	14	6	9	10	15	13	6	8	10	14	317143.0490	0.0432	0.025	0.0074	0.26
395:	14	6	8	10	13	13	6	7	10	12	317144.2170	-0.0244	0.025	0.0167	0.22
396:	14	6	8	10	16	13	6	7	10	15	317144.2170	0.0497	0.025	0.0167	0.28
397:	14	6	9	10	13	13	6	8	10	12	317144.2170	-0.0244	0.025	0.0167	0.22
398:	14	6	9	10	16	13	6	8	10	15	317144.2170	0.0497	0.025	0.0167	0.28
399:	14	2	13	10	13	13	2	12	10	12	317184.2640	-0.0034	0.025	0.0108	0.22
400:	14	2	13	10	14	13	2	12	10	13	317184.2640	0.1253	0.025	0.0108	0.24
401:	14	2	13	10	15	13	2	12	10	14	317184.2640	0.0320	0.025	0.0108	0.26
402:	14	2	13	10	16	13	2	12	10	15	317184.2640	-0.0967	0.025	0.0108	0.28
403:	14	5	9	10	14	13	5	8	10	13	317185.9930	-0.0131	0.025	-0.0044	0.24
404:	14	5	9	10	15	13	5	8	10	14	317185.9930	0.0036	0.025	-0.0044	0.26
405:	14	5	10	10	14	13	5	9	10	13	317185.9930	-0.0130	0.025	-0.0044	0.24
406:	14	5	10	10	15	13	5	9	10	14	317185.9930	0.0037	0.025	-0.0044	0.26
407:	14	5	9	10	13	13	5	8	10	12	317186.8480	0.0354	0.025	0.0447	0.22
408:	14	5	9	10	16	13	5	8	10	15	317186.8480	0.0519	0.025	0.0447	0.28
409:	14	5	10	10	13	13	5	9	10	12	317186.8480	0.0355	0.025	0.0447	0.22
410:	14	5	10	10	16	13	5	9	10	15	317186.8480	0.0521	0.025	0.0447	0.28
411:	14	4	10	10	14	13	4	9	10	13	317226.3850	0.0123	0.025	0.0142	0.24
412:	14	4	10	10	15	13	4	9	10	14	317226.3850	-0.0180	0.025	0.0142	0.26
413:	14	4	11	10	14	13	4	10	10	13	317226.3850	0.0475	0.025	0.0142	0.24
414:	14	4	11	10	15	13	4	10	10	14	317226.3850	0.0171	0.025	0.0142	0.26
415:	14	4	10	10	13	13	4	9	10	12	317226.9100	0.0211	0.025	0.0219	0.22
416:	14	4	10	10	16	13	4	9	10	15	317226.9100	-0.0093	0.025	0.0219	0.28
417:	14	4	11	10	13	13	4	10	10	12	317226.9100	0.0564	0.025	0.0219	0.22
418:	14	4	11	10	16	13	4	10	10	15	317226.9100	0.0258	0.025	0.0219	0.28
419:	14	3	12	10	13	13	3	11	10	12	317269.2640	-0.0949	0.025	0.0139	0.22
420:	14	3	12	10	14	13	3	11	10	13	317269.2640	0.1955	0.025	0.0139	0.24
421:	14	3	12	10	15	13	3	11	10	14	317269.2640	0.1284	0.025	0.0139	0.26

```
 422: 14  3 12 10 16   13  3 11 10 15       317269.2640  -0.1621  0.025   0.0139 0.28
 423: 14  3 11 10 13   13  3 10 10 12       317275.0120  -0.1153  0.025  -0.0064 0.22
 424: 14  3 11 10 14   13  3 10 10 13       317275.0120   0.1751  0.025  -0.0064 0.24
 425: 14  3 11 10 15   13  3 10 10 14       317275.0120   0.1081  0.025  -0.0064 0.26
 426: 14  3 11 10 16   13  3 10 10 15       317275.0120  -0.1824  0.025  -0.0064 0.28
 427: 14  2 12 10 13   13  2 11 10 12       317544.1170  -0.0161  0.025  -0.0012 0.22
 428: 14  2 12 10 14   13  2 11 10 13       317544.1170   0.1141  0.025  -0.0012 0.24
 429: 14  2 12 10 15   13  2 11 10 14       317544.1170   0.0209  0.025  -0.0012 0.26
 430: 14  2 12 10 16   13  2 11 10 15       317544.1170  -0.1094  0.025  -0.0012 0.28
 431: 14  1 13 10 13   13  1 12 10 12       319251.9550   0.0415  0.025  -0.0012 0.22
 432: 14  1 13 10 14   13  1 12 10 13       319251.9550   0.0733  0.025  -0.0012 0.24
 433: 14  1 13 10 15   13  1 12 10 14       319251.9550  -0.0359  0.025  -0.0012 0.26
 434: 14  1 13 10 16   13  1 12 10 15       319251.9550  -0.0678  0.025  -0.0012 0.28
---------------------------------------------------------------------------------=========
    5 Lines rejected from fit

PARAMETERS IN FIT (values truncated and Nlines statistics):

        1000000            A  /MHz        95425.1307(71)                     1
       -1001010            .  /MHz        95425.1307(71)         =  1.00000 *  1
        2000000            B  /MHz        11485.12341(25)                    2
       -2001010            .  /MHz        11485.12341(25)        =  1.00000 *  2
        3000000            C  /MHz        11185.58835(24)                    3
       -3001010            .  /MHz        11185.58835(24)        =  1.00000 *  3
          20000      Delta_J  /kHz           12.27402(76)                    4
         -21010            .  /kHz           12.27402(76)        =  1.00000 *  4
         110000     Delta_JK  /kHz          123.3619(21)                     5
        -111010            .  /kHz          123.3619(21)         =  1.00000 *  5
         200000      Delta_K  /kHz          963.28(39)                       6
        -201010            .  /kHz          963.28(39)           =  1.00000 *  6
        4010000      delta_J  /kHz            0.296208(43)                   7
       -4011010            .  /kHz            0.296208(43)       =  1.00000 *  7
        4100000      delta_K  /kHz           58.785(24)                      8
       -4101010            .  /kHz           58.785(24)          =  1.00000 *  8
          30000        Phi_J  /Hz           [-0.002322]                      9
         -31010            .  /Hz           [-0.002322]          =  1.00000 *  9
         120000       Phi_JK  /Hz           [ 0.346]                        10
        -121010            .  /Hz           [ 0.346]             =  1.00000 * 10
         210000       Phi_KJ  /Hz           [ 2.717]                        11
        -211010            .  /Hz           [ 2.717]             =  1.00000 * 11
         300000        Phi_K  /Hz           [32.]                           12
        -301010            .  /Hz           [32.]                =  1.00000 * 12
        4020000        phi_J  /Hz           [ 0.000153]                     13
       -4021010            .  /Hz           [ 0.000153]          =  1.00000 * 13
        4110000       phi_JK  /Hz           [ 0.254]                        14
       -4111010            .  /Hz           [ 0.254]             =  1.00000 * 14
        4200000        phi_K  /Hz           [24.]                           15
       -4201010            .  /Hz           [24.]                =  1.00000 * 15

    11001001010         X_aa  /MHz          -58.711(22)                     16
   -11003001010         X_cc  /MHz           58.711(22)          = -1.00000 * 16
    11002001010         X_bb  /MHz           29.52(20)                      17
   -11003001010         X_cc  /MHz          -29.52(20)           = -1.00000 * 17

 MICROWAVE AVG =        0.002619 MHz, IR AVG =        0.00000
 MICROWAVE RMS =        0.042450 MHz, IR RMS =        0.00000
 END OF ITERATION  1 OLD, NEW RMS ERROR=        0.91556         0.91556

  distinct frequency lines in fit:   168
       distinct parameters of fit:    10
          lines rejected from fit:     5    (ERRTST =  3.00E+00)

MICROWAVE       lines fitted       lines    lines      RMS      RMS ERROR    J range  Ka range
freq. range
            total   dv=0 dv.ne.0   UNFITTD  e>900
v"= 0         22     22      0        3      0       0.049779     1.11150     4  35    0   5
26635    37720
v"=10        146    146      0        2      0       0.041222     0.88188     0  14    0  12
22674   319252
-------------------------------------------------------------------------------------------
total:       168    168      0        5      0       0.042441     0.91523

NOTE: the RMS values above are for Nlines statistics, but the 'total' values may differ slightly
from
      those in the .FIT file since the o-c values for this evaluation are as rounded in the .FIT.


 PARAMETERS IN FIT WITH STANDARD ERRORS ON THOSE THAT ARE FITTED:
```

```
 (values rounded and degrees of freedom, Ndegf=Nlines-Nconst, statistics)

       1000000            A  /MHz      95425.1307(67)                       1
      -1001010            .  /MHz      95425.1307(67)          =  1.00000 *  1
       2000000            B  /MHz      11485.12342(23)                      2
      -2001010            .  /MHz      11485.12342(23)         =  1.00000 *  2
       3000000            C  /MHz      11185.58835(23)                      3
      -3001010            .  /MHz      11185.58835(23)         =  1.00000 *  3
         20000      Delta_J  /kHz         12.27402(72)                      4
        -21010            .  /kHz         12.27402(72)         =  1.00000 *  4
        110000     Delta_JK  /kHz        123.3620(20)                       5
       -111010            .  /kHz        123.3620(20)          =  1.00000 *  5
        200000      Delta_K  /kHz        963.28(37)                         6
       -201010            .  /kHz        963.28(37)            =  1.00000 *  6
       4010000      delta_J  /kHz          0.296208(41)                     7
      -4011010            .  /kHz          0.296208(41)        =  1.00000 *  7
       4100000      delta_K  /kHz         58.785(22)                        8
      -4101010            .  /kHz         58.785(22)           =  1.00000 *  8
         30000        Phi_J  /Hz           [-0.002322]                      9
        -31010            .  /Hz           [-0.002322]         =  1.00000 *  9
        120000       Phi_JK  /Hz           [ 0.346]                        10
       -121010            .  /Hz           [ 0.346]            =  1.00000 * 10
        210000       Phi_KJ  /Hz           [ 2.717]                        11
       -211010            .  /Hz           [ 2.717]            =  1.00000 * 11
        300000        Phi_K  /Hz           [32.]                           12
       -301010            .  /Hz           [32.]               =  1.00000 * 12
       4020000        phi_J  /Hz           [ 0.000153]                     13
      -4021010            .  /Hz           [ 0.000153]         =  1.00000 * 13
       4110000       phi_JK  /Hz           [ 0.254]                        14
      -4111010            .  /Hz           [ 0.254]            =  1.00000 * 14
       4200000        phi_K  /Hz           [24.]                           15
      -4201010            .  /Hz           [24.]               =  1.00000 * 15

   11001001010         X_aa  /MHz        -58.711(21)                       16
  -11003001010         X_cc  /MHz         58.711(21)           = -1.00000 * 16
   11002001010         X_bb  /MHz         29.52(19)                        17
  -11003001010         X_cc  /MHz        -29.52(19)            = -1.00000 * 17

 CORRELATION COEFFICIENTS, C.ij:

           A        B        C        -Delta_J -Delta_J -Delta_K -delta_J -delta_K

A           1.0000
B           0.3133   1.0000
C           0.2139   0.8121   1.0000
-Delta_J   -0.2379  -0.8032  -0.7817   1.0000
-Delta_JK  -0.0249  -0.1174  -0.1618  -0.2913   1.0000
-Delta_K   -0.6166  -0.6660  -0.6053   0.8659  -0.3754   1.0000
-delta_J    0.3084  -0.0596   0.1831  -0.1084   0.1205  -0.2182   1.0000
-delta_K   -0.3644  -0.2220   0.0627   0.1462  -0.1572   0.2656  -0.5953   1.0000
X_aa       -0.0447  -0.1534  -0.1533   0.1282   0.0381   0.1008  -0.0085   0.0120
X_bb       -0.0042   0.0037  -0.0101   0.0033  -0.0014   0.0042  -0.0250   0.0056

           X_aa     X_bb

X_aa        1.0000
X_bb       -0.0815   1.0000

 Mean value of |C.ij|, i.ne.j =   0.2328
 Mean value of  C.ij,  i.ne.j =  -0.0734


 No correlations with absolute value greater than 0.9950


 Worst fitted lines (obs-calc/error):

     3:    2.8        251:   -2.8         16:   -2.5          1:    2.2
    29:   -2.2        269:    2.1        257:    1.9        281:   -1.9
   277:   -1.8        177:    1.8        407:    1.8        275:    1.7
   247:   -1.6        319:   -1.6         31:    1.6         83:    1.6
   253:   -1.6        113:    1.5         36:    1.5        265:    1.5
   145:    1.5          4:    1.4         10:   -1.4         17:    1.3
   279:    1.3        195:   -1.3         37:   -1.3         51:   -1.2
   173:    1.2        119:   -1.2        259:   -1.2        185:    1.2
   149:    1.2         27:   -1.1         19:    1.1         85:    1.0
   263:   -1.0        189:    1.0        109:    1.0        107:   -1.0
   371:   -1.0        229:    1.0        261:    1.0         49:    0.9
```

```
   239:   -0.9        323:    0.9        271:   -0.9         34:    0.9
   415:    0.9        155:    0.9

     3:  1  0  1 10  1    0  0  0 10  2        22685.6000   0.2563  0.090
   251: 13 12  1 10 15   12 12  0 10 14       294156.9840  -0.0701  0.025  -0.0702 0.50
    16: 13  1 13  0 13   12  2 11  0 12        28664.1200  -0.1252  0.050
     1:  1  0  1 10  3    0  0  0 10  2        22673.8000   0.2004  0.090
    29: 29  1 29  0 29   29  0 29  0 29        37032.7810  -0.0433  0.020
   269: 13 10  3 10 12   12 10  2 10 11       294298.3620   0.0517  0.025   0.0517 0.50
   257: 13 11  2 10 13   12 11  1 10 12       294226.3620   0.0483  0.025   0.0484 0.50
   281: 13  9  4 10 12   12  9  3 10 11       294359.2820  -0.0479  0.025  -0.0479 0.50
   277: 13  9  4 10 13   12  9  3 10 12       294356.0180  -0.0458  0.025  -0.0458 0.50
   177: 12  9  3 10 11   11  9  2 10 10       271731.1970   0.0452  0.025   0.0452 0.50
                                             _____________________________________
_________________________________________/ SPFIT output reformatted with PIFORM
```

Table S.3. Fit of the rotational transitions of $^{13}CHD_2{}^{35}Cl$ in PIFORM format

```
13CHD2Cl -- 35Cl                                          Tue Mar 18 09:44:02 2025

---------------------------------------------------------------------------------=========
                                             obs         o-c     error     blends     Notes
                                                                         o-c      wt
    / instead of : below denotes (o-c)>3*err
---------------------------------------------------------------------------------=========
    1:  4  1  4  5    3  1  3  5            88881.1581   0.0325  0.020
    2:  4  1  4  4    3  1  3  3            88894.0946  -0.0063  0.020
    3:  4  1  4  5    3  1  3  4            88895.0652  -0.0005  0.020
    4:  4  1  4  3    3  1  3  2            88895.9632   0.0015  0.020
    5:  4  1  4  6    3  1  3  5            88896.9389   0.0108  0.020
    6:  4  1  4  4    3  1  3  4            88900.5830  -0.0259  0.020
    7:  4  1  4  3    3  1  3  3            88909.9442   0.0392  0.020
    8:  4  0  4  5    3  0  3  5            89453.3750  -0.0047  0.020
    9:  4  3  1  4    3  3  0  4            89457.9513   0.0148  0.020   0.0185 0.50
   10:  4  3  2  4    3  3  1  4            89457.9513   0.0221  0.020   0.0185 0.50
   11:  4  3  1  5    3  3  0  4            89460.2320   0.0074  0.020   0.0110 0.50
   12:  4  3  2  5    3  3  1  4            89460.2320   0.0146  0.020   0.0110 0.50
   13:  4  3  1  3    3  3  0  3            89462.3082   0.0196  0.020   0.0233 0.50
   14:  4  3  2  3    3  3  1  3            89462.3082   0.0269  0.020   0.0233 0.50
   15:  4  3  1  4    3  3  0  3            89468.7855  -0.0125  0.020  -0.0090 0.50
   16:  4  3  2  4    3  3  1  3            89468.7855  -0.0053  0.020  -0.0090 0.50
   17:  4  0  4  3    3  0  3  2            89469.7996  -0.0342  0.020   0.0208 0.27
   18:  4  0  4  4    3  0  3  3            89469.7996  -0.0336  0.020   0.0208 0.36
   19:  4  2  3  5    3  2  2  4            89469.7996   0.1137  0.020   0.0208 0.34
   20:  4  2  3  5    3  2  2  5            89469.7996   0.1093  0.020   0.0208 0.03
   21:  4  0  4  5    3  0  3  4            89472.0167   0.0226  0.020  -0.0416 0.34
   22:  4  0  4  6    3  0  3  5            89472.0167   0.0227  0.020  -0.0416 0.45
   23:  4  2  3  4    3  2  2  3            89472.0167  -0.2819  0.020  -0.0416 0.18
   24:  4  2  3  4    3  2  2  4            89472.0167  -0.2789  0.020  -0.0416 0.03
   25:  4  2  2  5    3  2  1  4            89477.0636  -0.1200  0.020  -0.0243 0.26
   26:  4  2  2  5    3  2  1  5            89477.0636  -0.1240  0.020  -0.0243 0.02
   27:  4  2  3  6    3  2  2  5            89477.0636  -0.0698  0.020  -0.0243 0.34
   28:  4  3  1  6    3  3  0  5            89477.0636   0.0873  0.020  -0.0243 0.19
   29:  4  3  2  6    3  3  1  5            89477.0636   0.0945  0.020  -0.0243 0.19
   30:  4  2  2  4    3  2  1  3            89479.7847  -0.0118  0.020   0.0115 0.49
   31:  4  2  2  4    3  2  1  4            89479.7847  -0.0089  0.020   0.0115 0.08
   32:  4  2  3  3    3  2  2  2            89479.7847   0.0429  0.020   0.0115 0.37
   33:  4  2  3  3    3  2  2  3            89479.7847   0.0365  0.020   0.0115 0.06
   34:  4  3  1  5    3  3  0  5            89483.5196   0.0241  0.020   0.0278 0.50
   35:  4  3  2  5    3  3  1  5            89483.5196   0.0314  0.020   0.0278 0.50
   36:  4  2  2  6    3  2  1  5            89484.6339   0.0023  0.020
   37:  4  3  1  3    3  3  0  2            89485.5797   0.0257  0.020   0.0294 0.50
   38:  4  3  2  3    3  3  1  2            89485.5797   0.0330  0.020   0.0294 0.50
   39:  4  2  2  3    3  2  1  2            89487.2541   0.0142  0.020   0.0133 0.86
   40:  4  2  2  3    3  2  1  3            89487.2541   0.0074  0.020   0.0133 0.14
   41:  4  0  4  3    3  0  3  3            89488.4169  -0.0299  0.020
   42:  4  1  3  4    3  1  2  3            90055.3541   0.0057  0.020
   43:  4  1  3  5    3  1  2  4            90056.3265   0.0089  0.020
   44:  4  1  3  3    3  1  2  2            90057.2233   0.0141  0.020
   45:  4  1  3  6    3  1  2  5            90058.1940   0.0141  0.020
   46:  4  1  3  4    3  1  2  4            90061.8633  -0.0109  0.020
   47:  8  1  8  7    7  1  7  6           177761.7880   0.0982  0.040  -0.0146 0.20
   48:  8  1  8  8    7  1  7  7           177761.7880   0.3196  0.040  -0.0146 0.23
   49:  8  1  8  9    7  1  7  8           177761.7880  -0.0750  0.040  -0.0146 0.26
   50:  8  1  8 10    7  1  7  9           177761.7880  -0.2965  0.040  -0.0146 0.30
   51:  8  7  1  9    7  7  0  8           178838.9450   0.0251  0.040   0.0252 0.50
   52:  8  7  2  9    7  7  1  8           178838.9450   0.0251  0.040   0.0252 0.50
   53:  8  7  1  8    7  7  0  7           178841.6330   0.0247  0.040   0.0247 0.50
   54:  8  7  2  8    7  7  1  7           178841.6330   0.0247  0.040   0.0247 0.50
   55:  8  7  1 10    7  7  0  9           178849.7650  -0.0126  0.040  -0.0126 0.50
   56:  8  7  2 10    7  7  1  9           178849.7650  -0.0126  0.040  -0.0126 0.50
   57:  8  7  1  7    7  7  0  6           178852.4100  -0.0585  0.040  -0.0586 0.50
   58:  8  7  2  7    7  7  1  6           178852.4100  -0.0585  0.040  -0.0586 0.50
   59:  8  6  2  9    7  6  1  8           178866.7670   0.0292  0.040   0.0293 0.50
   60:  8  6  3  9    7  6  2  8           178866.7670   0.0292  0.040   0.0293 0.50
   61:  8  6  2  8    7  6  1  7           178868.5330  -0.0590  0.040  -0.0590 0.50
   62:  8  6  3  8    7  6  2  7           178868.5330  -0.0590  0.040  -0.0590 0.50
   63:  8  6  2 10    7  6  1  9           178874.7420   0.0263  0.040   0.0263 0.50
   64:  8  6  3 10    7  6  2  9           178874.7420   0.0263  0.040   0.0263 0.50
   65:  8  0  8  7    7  0  7  6           178876.5560   0.3778  0.040   0.1053 0.18
   66:  8  0  8  8    7  0  7  7           178876.5560   0.3773  0.040   0.1053 0.21
   67:  8  6  2  7    7  6  1  6           178876.5560  -0.0139  0.040   0.1053 0.05
   68:  8  6  3  7    7  6  2  6           178876.5560  -0.0139  0.040   0.1053 0.05
```

69:	8	0	8	10	7	0	7	9	178876.5560	-0.0816	0.040	0.1053	0.27
70:	8	0	8	9	7	0	7	8	178876.5560	-0.0822	0.040	0.1053	0.24
71:	8	5	3	9	7	5	2	8	178890.7220	0.0134	0.040	0.0135	0.50
72:	8	5	4	9	7	5	3	8	178890.7220	0.0134	0.040	0.0135	0.50
73:	8	5	3	8	7	5	2	7	178891.8620	0.0055	0.040	0.0056	0.50
74:	8	5	4	8	7	5	3	7	178891.8620	0.0055	0.040	0.0056	0.50
75:	8	5	3	10	7	5	2	9	178896.2300	-0.0192	0.040	-0.0193	0.50
76:	8	5	4	10	7	5	3	9	178896.2300	-0.0192	0.040	-0.0193	0.50
77:	8	5	3	7	7	5	2	6	178897.3860	-0.0100	0.040	-0.0100	0.50
78:	8	5	4	7	7	5	3	6	178897.3860	-0.0100	0.040	-0.0100	0.50
79:	8	4	4	9	7	4	3	8	178911.3010	0.0555	0.040	0.0558	0.50
80:	8	4	5	9	7	4	4	8	178911.3010	0.0560	0.040	0.0558	0.50
81:	8	4	4	8	7	4	3	7	178911.8810	0.0659	0.040	0.0663	0.50
82:	8	4	5	8	7	4	4	7	178911.8810	0.0665	0.040	0.0663	0.50
83:	8	4	4	10	7	4	3	9	178914.8110	0.0191	0.040	0.0194	0.50
84:	8	4	5	10	7	4	4	9	178914.8110	0.0197	0.040	0.0194	0.50
85:	8	4	4	7	7	4	3	6	178915.3840	0.0239	0.040	0.0242	0.50
86:	8	4	5	7	7	4	4	6	178915.3840	0.0244	0.040	0.0242	0.50
87:	8	2	7	8	7	2	6	7	178921.9890	0.0816	0.040	-0.0260	0.47
88:	8	2	7	9	7	2	6	8	178921.9890	-0.1205	0.040	-0.0260	0.53
89:	8	2	7	7	7	2	6	6	178922.9420	0.1485	0.040	0.0279	0.40
90:	8	2	7	10	7	2	6	9	178922.9420	-0.0541	0.040	0.0279	0.60
91:	8	3	5	8	7	3	4	7	178929.7700	-0.2168	0.040	-0.0013	0.23
92:	8	3	5	9	7	3	4	8	178929.7700	-0.0973	0.040	-0.0013	0.27
93:	8	3	6	8	7	3	5	7	178929.7700	0.0870	0.040	-0.0013	0.23
94:	8	3	6	9	7	3	5	8	178929.7700	0.2065	0.040	-0.0013	0.27
95:	8	3	5	7	7	3	4	6	178931.7510	-0.2298	0.040	-0.0074	0.20
96:	8	3	5	10	7	3	4	9	178931.7510	-0.1113	0.040	-0.0074	0.30
97:	8	3	6	7	7	3	5	6	178931.7510	0.0740	0.040	-0.0074	0.20
98:	8	3	6	10	7	3	5	9	178931.7510	0.1925	0.040	-0.0074	0.30
99:	8	2	6	8	7	2	5	7	178984.9130	0.0919	0.040	-0.0156	0.47
100:	8	2	6	9	7	2	5	8	178984.9130	-0.1101	0.040	-0.0156	0.53
101:	8	2	6	7	7	2	5	6	178985.8680	0.1602	0.040	0.0397	0.40
102:	8	2	6	10	7	2	5	9	178985.8680	-0.0423	0.040	0.0397	0.60
103:	8	1	7	7	7	1	6	6	180083.1990	0.1655	0.040	0.0522	0.20
104:	8	1	7	8	7	1	6	7	180083.1990	0.3869	0.040	0.0522	0.23
105:	8	1	7	9	7	1	6	8	180083.1990	-0.0086	0.040	0.0522	0.26
106:	8	1	7	10	7	1	6	9	180083.1990	-0.2301	0.040	0.0522	0.30
107:	9	1	9	11	8	1	8	10	199970.2160	-0.2081	0.040	0.0103	0.29
108:	9	1	9	8	8	1	8	7	199970.2160	0.1108	0.040	0.0103	0.21
109:	9	1	9	9	8	1	8	8	199970.2160	0.2658	0.040	0.0103	0.23
110:	9	1	9	10	8	1	8	9	199970.2160	-0.0531	0.040	0.0103	0.26
111:	9	8	1	11	8	8	0	10	201164.7500	0.0558	0.040	0.0558	0.50
112:	9	8	2	11	8	8	1	10	201164.7500	0.0558	0.040	0.0558	0.50
113:	9	8	1	8	8	8	0	7	201166.9370	0.0581	0.040	0.0582	0.50
114:	9	8	2	8	8	8	1	7	201166.9370	0.0581	0.040	0.0582	0.50
115:	9	7	2	10	8	7	1	9	201189.6930	-0.0641	0.040	-0.0641	0.50
116:	9	7	3	10	8	7	2	9	201189.6930	-0.0641	0.040	-0.0641	0.50
117:	9	7	2	9	8	7	1	8	201191.2550	-0.0896	0.040	-0.0897	0.50
118:	9	7	3	9	8	7	2	8	201191.2550	-0.0896	0.040	-0.0897	0.50
119:	9	7	2	11	8	7	1	10	201197.3270	-0.0312	0.040	-0.0312	0.50
120:	9	7	3	11	8	7	2	10	201197.3270	-0.0312	0.040	-0.0312	0.50
121:	9	7	2	8	8	7	1	7	201198.8980	-0.0480	0.040	-0.0480	0.50
122:	9	7	3	8	8	7	2	7	201198.8980	-0.0480	0.040	-0.0480	0.50
123:	9	0	9	11	8	0	8	10	201209.6200	-0.1327	0.040	0.0262	0.29
124:	9	0	9	8	8	0	8	7	201209.6200	0.2263	0.040	0.0262	0.21
125:	9	0	9	9	8	0	8	8	201209.6200	0.2257	0.040	0.0262	0.23
126:	9	0	9	10	8	0	8	9	201209.6200	-0.1334	0.040	0.0262	0.26
127:	9	6	3	10	8	6	2	9	201220.3580	-0.0576	0.040	-0.0576	0.50
128:	9	6	4	10	8	6	3	9	201220.3580	-0.0576	0.040	-0.0576	0.50
129:	9	6	3	9	8	6	2	8	201221.4630	-0.0242	0.040	-0.0242	0.50
130:	9	6	4	9	8	6	3	8	201221.4630	-0.0242	0.040	-0.0242	0.50
131:	9	6	3	11	8	6	2	10	201225.9350	-0.0655	0.040	-0.0655	0.50
132:	9	6	4	11	8	6	3	10	201225.9350	-0.0655	0.040	-0.0655	0.50
133:	9	6	3	8	8	6	2	7	201227.0400	-0.0313	0.040	-0.0314	0.50
134:	9	6	4	8	8	6	3	7	201227.0400	-0.0313	0.040	-0.0314	0.50
135:	9	5	4	10	8	5	3	9	201246.9940	0.0248	0.040	0.0248	0.50
136:	9	5	5	10	8	5	4	9	201246.9940	0.0248	0.040	0.0248	0.50
137:	9	5	4	9	8	5	3	8	201247.6010	-0.0029	0.040	-0.0029	0.50
138:	9	5	5	9	8	5	4	8	201247.6010	-0.0029	0.040	-0.0029	0.50
139:	9	5	4	11	8	5	3	10	201250.8440	-0.0038	0.040	-0.0038	0.50
140:	9	5	5	11	8	5	4	10	201250.8440	-0.0038	0.040	-0.0038	0.50
141:	9	5	4	8	8	5	3	7	201251.4960	0.0144	0.040	0.0145	0.50
142:	9	5	5	8	8	5	4	7	201251.4960	0.0144	0.040	0.0145	0.50
143:	9	4	5	9	8	4	4	8	201270.0980	-0.1874	0.040	-0.0404	0.24
144:	9	4	5	10	8	4	4	9	201270.0980	0.0896	0.040	-0.0404	0.26
145:	9	4	6	9	8	4	5	8	201270.0980	-0.1861	0.040	-0.0404	0.24
146:	9	4	6	10	8	4	5	9	201270.0980	0.0909	0.040	-0.0404	0.26

147:	9	4	5	11	8	4	4	10	201272.5680	0.0771	0.040	-0.0369	0.29
148:	9	4	5	8	8	4	4	7	201272.5680	-0.1989	0.040	-0.0369	0.21
149:	9	4	6	11	8	4	5	10	201272.5680	0.0784	0.040	-0.0369	0.29
150:	9	4	6	8	8	4	5	7	201272.5680	-0.1977	0.040	-0.0369	0.21
151:	9	2	8	9	8	2	7	8	201277.0020	0.1442	0.040	0.0386	0.47
152:	9	2	8	10	8	2	7	9	201277.0020	-0.0557	0.040	0.0386	0.53
153:	9	2	8	11	8	2	7	10	201277.5510	-0.1273	0.040	-0.0440	0.58
154:	9	2	8	8	8	2	7	7	201277.5510	0.0730	0.040	-0.0440	0.42
155:	9	3	6	9	8	3	5	8	201291.3880	-0.3694	0.040	-0.0916	0.24
156:	9	3	6	10	8	3	5	9	201291.3880	-0.3706	0.040	-0.0916	0.26
157:	9	3	7	9	8	3	6	8	201291.3880	0.1874	0.040	-0.0916	0.24
158:	9	3	7	10	8	3	6	9	201291.3880	0.1862	0.040	-0.0916	0.26
159:	9	3	6	11	8	3	5	10	201292.9090	-0.2461	0.040	0.0331	0.29
160:	9	3	6	8	8	3	5	7	201292.9090	-0.2443	0.040	0.0331	0.21
161:	9	3	7	11	8	3	6	10	201292.9090	0.3107	0.040	0.0331	0.29
162:	9	3	7	8	8	3	6	7	201292.9090	0.3125	0.040	0.0331	0.21
163:	9	2	7	9	8	2	6	8	201366.8450	0.1616	0.040	0.0560	0.47
164:	9	2	7	10	8	2	6	9	201366.8450	-0.0383	0.040	0.0560	0.53
165:	9	2	7	11	8	2	6	10	201367.4010	-0.1035	0.040	-0.0203	0.58
166:	9	2	7	8	8	2	6	7	201367.4010	0.0967	0.040	-0.0203	0.42
167:	9	1	8	11	8	1	7	10	202581.1740	-0.2019	0.040	0.0169	0.29
168:	9	1	8	8	8	1	7	7	202581.1740	0.1178	0.040	0.0169	0.21
169:	9	1	8	9	8	1	7	8	202581.1740	0.2727	0.040	0.0169	0.23
170:	9	1	8	10	8	1	7	9	202581.1740	-0.0469	0.040	0.0169	0.26
171:	10	1	10	11	9	1	9	10	222174.4480	-0.0792	0.040	-0.0179	0.26
172:	10	1	10	12	9	1	9	11	222174.4480	-0.1918	0.040	-0.0179	0.29
173:	10	1	10	9	9	1	9	8	222174.4480	0.0706	0.040	-0.0179	0.21
174:	10	1	10	10	9	1	9	9	222174.4480	0.1832	0.040	-0.0179	0.24
175:	10	9	1	11	9	9	0	10	223455.6890	0.0029	0.040	0.0030	0.50
176:	10	9	2	11	9	9	1	10	223455.6890	0.0029	0.040	0.0030	0.50
177:	10	9	1	10	9	9	0	9	223457.4490	-0.0441	0.040	-0.0441	0.50
178:	10	9	2	10	9	9	1	9	223457.4490	-0.0441	0.040	-0.0441	0.50
179:	10	9	1	12	9	9	0	11	223464.8150	-0.0085	0.040	-0.0086	0.50
180:	10	9	2	12	9	9	1	11	223464.8150	-0.0085	0.040	-0.0086	0.50
181:	10	9	1	9	9	9	0	8	223466.6700	0.0374	0.040	0.0375	0.50
182:	10	9	2	9	9	9	1	8	223466.6700	0.0374	0.040	0.0375	0.50
183:	10	8	2	11	9	8	1	10	223498.9170	-0.0126	0.040	-0.0127	0.50
184:	10	8	3	11	9	8	2	10	223498.9170	-0.0126	0.040	-0.0127	0.50
185:	10	8	2	10	9	8	1	9	223500.2760	-0.0215	0.040	-0.0215	0.50
186:	10	8	3	10	9	8	2	9	223500.2760	-0.0215	0.040	-0.0215	0.50
187:	10	8	2	12	9	8	1	11	223506.1430	-0.0069	0.040	-0.0070	0.50
188:	10	8	3	12	9	8	2	11	223506.1430	-0.0069	0.040	-0.0070	0.50
189:	10	8	2	9	9	8	1	8	223507.5400	0.0218	0.040	0.0218	0.50
190:	10	8	3	9	9	8	2	8	223507.5400	0.0218	0.040	0.0218	0.50
191:	10	0	10	11	9	0	9	10	223533.4350	-0.1627	0.040	-0.0327	0.26
192:	10	0	10	12	9	0	9	11	223533.4350	-0.1620	0.040	-0.0327	0.29
193:	10	0	10	9	9	0	9	8	223533.4350	0.1266	0.040	-0.0327	0.21
194:	10	0	10	10	9	0	9	9	223533.4350	0.1259	0.040	-0.0327	0.24
195:	10	7	3	11	9	7	2	10	223537.3490	-0.0142	0.040	-0.0143	0.50
196:	10	7	4	11	9	7	3	10	223537.3490	-0.0142	0.040	-0.0143	0.50
197:	10	7	3	10	9	7	2	9	223538.3670	0.0237	0.040	0.0237	0.50
198:	10	7	4	10	9	7	3	9	223538.3670	0.0237	0.040	0.0237	0.50
199:	10	7	3	12	9	7	2	11	223542.8580	-0.0336	0.040	-0.0336	0.50
200:	10	7	4	12	9	7	3	11	223542.8580	-0.0336	0.040	-0.0336	0.50
201:	10	7	3	9	9	7	2	8	223543.9360	0.0648	0.040	0.0648	0.50
202:	10	7	4	9	9	7	3	8	223543.9360	0.0648	0.040	0.0648	0.50
203:	10	6	4	11	9	6	3	10	223571.1270	0.0022	0.040	0.0022	0.50
204:	10	6	5	11	9	6	4	10	223571.1270	0.0022	0.040	0.0022	0.50
205:	10	6	4	10	9	6	3	9	223571.7260	-0.0424	0.040	-0.0424	0.50
206:	10	6	5	10	9	6	4	9	223571.7260	-0.0424	0.040	-0.0424	0.50
207:	10	6	4	12	9	6	3	11	223575.1790	-0.0076	0.040	-0.0076	0.50
208:	10	6	5	12	9	6	4	11	223575.1790	-0.0076	0.040	-0.0076	0.50
209:	10	6	4	9	9	6	3	8	223575.7340	-0.0955	0.040	-0.0955	0.50
210:	10	6	5	9	9	6	4	8	223575.7340	-0.0955	0.040	-0.0955	0.50
211:	10	5	5	11	9	5	4	10	223600.6880	0.1606	0.040	-0.0097	0.26
212:	10	5	5	10	9	5	4	9	223600.6880	-0.1982	0.040	-0.0097	0.24
213:	10	5	6	11	9	5	5	10	223600.6880	0.1606	0.040	-0.0097	0.26
214:	10	5	6	10	9	5	5	9	223600.6880	-0.1982	0.040	-0.0097	0.24
215:	10	5	5	12	9	5	4	11	223603.4650	0.1167	0.040	-0.0351	0.29
216:	10	5	5	9	9	5	4	8	223603.4650	-0.2413	0.040	-0.0351	0.21
217:	10	5	6	12	9	5	5	11	223603.4650	0.1167	0.040	-0.0351	0.29
218:	10	5	6	9	9	5	5	8	223603.4650	-0.2413	0.040	-0.0351	0.21
219:	10	4	6	11	9	4	5	10	223626.4300	0.0466	0.040	-0.0117	0.26
220:	10	4	6	10	9	4	5	9	223626.4300	-0.0792	0.040	-0.0117	0.24
221:	10	4	7	11	9	4	6	10	223626.4300	0.0493	0.040	-0.0117	0.26
222:	10	4	7	10	9	4	6	9	223626.4300	-0.0765	0.040	-0.0117	0.24
223:	10	2	9	11	9	2	8	10	223628.2970	0.0525	0.040	-0.0279	0.15
224:	10	2	9	12	9	2	8	11	223628.2970	-0.3986	0.040	-0.0279	0.17

225:	10	2	9	9	9	2	8	8	223628.2970	-0.2134	0.040	-0.0279	0.12
226:	10	2	9	10	9	2	8	9	223628.2970	0.2375	0.040	-0.0279	0.14
227:	10	4	6	12	9	4	5	11	223628.2970	0.1081	0.040	-0.0279	0.12
228:	10	4	6	9	9	4	5	8	223628.2970	-0.0170	0.040	-0.0279	0.09
229:	10	4	7	12	9	4	6	11	223628.2970	0.1109	0.040	-0.0279	0.12
230:	10	4	7	9	9	4	6	8	223628.2970	-0.0143	0.040	-0.0279	0.09
231:	10	3	8	11	9	3	7	10	223650.8020	-0.0847	0.040	-0.0584	0.53
232:	10	3	8	10	9	3	7	9	223650.8020	-0.0293	0.040	-0.0584	0.47
233:	10	3	7	11	9	3	6	10	223651.8250	-0.0161	0.040	-0.0219	0.26
234:	10	3	7	10	9	3	6	9	223651.8250	0.0392	0.040	-0.0219	0.24
235:	10	3	8	12	9	3	7	11	223651.8250	-0.0774	0.040	-0.0219	0.29
236:	10	3	8	9	9	3	7	8	223651.8250	-0.0215	0.040	-0.0219	0.21
237:	10	3	7	12	9	3	6	11	223652.8620	0.0051	0.040	0.0289	0.58
238:	10	3	7	9	9	3	6	8	223652.8620	0.0610	0.040	0.0289	0.42
239:	10	2	8	11	9	2	7	10	223751.7870	0.1246	0.040	-0.0193	0.26
240:	10	2	8	12	9	2	7	11	223751.7870	-0.3273	0.040	-0.0193	0.29
241:	10	2	8	9	9	2	7	8	223751.7870	-0.1421	0.040	-0.0193	0.21
242:	10	2	8	10	9	2	7	9	223751.7870	0.3096	0.040	-0.0193	0.24
243:	10	1	9	11	9	1	8	10	225074.7250	-0.0947	0.040	-0.0332	0.26
244:	10	1	9	12	9	1	8	11	225074.7250	-0.2074	0.040	-0.0332	0.29
245:	10	1	9	9	9	1	8	8	225074.7250	0.0556	0.040	-0.0332	0.21
246:	10	1	9	10	9	1	8	9	225074.7250	0.1682	0.040	-0.0332	0.24
247:	11	1	11	11	10	1	10	10	244374.1580	0.1762	0.040	0.0141	0.24
248:	11	1	11	12	10	1	10	11	244374.1580	-0.0431	0.040	0.0141	0.26
249:	11	1	11	13	10	1	10	12	244374.1580	-0.1275	0.040	0.0141	0.29
250:	11	1	11	10	10	1	10	9	244374.1580	0.0918	0.040	0.0141	0.22
251:	11	10	1	12	10	10	0	11	245739.9630	-0.0074	0.040	-0.0075	0.50
252:	11	10	2	12	10	10	1	11	245739.9630	-0.0074	0.040	-0.0075	0.50
253:	11	10	1	11	10	10	0	10	245741.4890	-0.0023	0.040	-0.0023	0.50
254:	11	10	2	11	10	10	1	10	245741.4890	-0.0023	0.040	-0.0023	0.50
255:	11	10	1	13	10	10	0	12	245748.4420	0.0108	0.040	0.0108	0.50
256:	11	10	2	13	10	10	1	12	245748.4420	0.0108	0.040	0.0108	0.50
257:	11	10	1	10	10	10	0	9	245749.9700	0.0163	0.040	0.0163	0.50
258:	11	10	2	10	10	10	1	9	245749.9700	0.0163	0.040	0.0163	0.50
259:	11	9	2	12	10	9	1	11	245792.5040	0.0000	0.040	0.0001	0.50
260:	11	9	3	12	10	9	2	11	245792.5040	0.0000	0.040	0.0001	0.50
261:	11	9	2	11	10	9	1	10	245793.7030	0.0117	0.040	0.0118	0.50
262:	11	9	3	11	10	9	2	10	245793.7030	0.0117	0.040	0.0118	0.50
263:	11	9	2	13	10	9	1	12	245799.3530	-0.0045	0.040	-0.0045	0.50
264:	11	9	3	13	10	9	2	12	245799.3530	-0.0045	0.040	-0.0045	0.50
265:	11	9	2	10	10	9	1	9	245800.5540	0.0087	0.040	0.0087	0.50
266:	11	9	3	10	10	9	2	9	245800.5540	0.0087	0.040	0.0087	0.50
267:	11	8	3	12	10	8	2	11	245839.7160	-0.0365	0.040	-0.0365	0.50
268:	11	8	4	12	10	8	3	11	245839.7160	-0.0365	0.040	-0.0365	0.50
269:	11	8	3	11	10	8	2	10	245840.6560	0.0148	0.040	0.0148	0.50
270:	11	8	4	11	10	8	3	10	245840.6560	0.0148	0.040	0.0148	0.50
271:	11	8	3	13	10	8	2	12	245845.1240	-0.0439	0.040	-0.0440	0.50
272:	11	8	4	13	10	8	3	12	245845.1240	-0.0439	0.040	-0.0440	0.50
273:	11	8	3	10	10	8	2	9	245845.9510	-0.1054	0.040	-0.1054	0.50
274:	11	8	4	10	10	8	3	9	245845.9510	-0.1054	0.040	-0.1054	0.50
275:	11	0	11	11	10	0	10	10	245847.0510	0.1171	0.040	-0.0120	0.24
276:	11	0	11	12	10	0	10	11	245847.0510	-0.1201	0.040	-0.0120	0.26
277:	11	0	11	13	10	0	10	12	245847.0510	-0.1193	0.040	-0.0120	0.29
278:	11	0	11	10	10	0	10	9	245847.0510	0.1179	0.040	-0.0120	0.22
279:	11	7	4	12	10	7	3	11	245881.7730	-0.0318	0.040	-0.0318	0.50
280:	11	7	5	12	10	7	4	11	245881.7730	-0.0318	0.040	-0.0318	0.50
281:	11	7	4	11	10	7	3	10	245882.4380	0.0081	0.040	0.0082	0.50
282:	11	7	5	11	10	7	4	10	245882.4380	0.0081	0.040	0.0082	0.50
283:	11	7	4	13	10	7	3	12	245885.9190	-0.0322	0.040	-0.0322	0.50
284:	11	7	5	13	10	7	4	12	245885.9190	-0.0322	0.040	-0.0322	0.50
285:	11	7	4	10	10	7	3	9	245886.5990	0.0233	0.040	0.0234	0.50
286:	11	7	5	10	10	7	4	9	245886.5990	0.0233	0.040	0.0234	0.50
287:	11	6	5	11	10	6	4	10	245919.0120	-0.2313	0.040	-0.0240	0.24
288:	11	6	5	12	10	6	4	11	245919.0120	0.1650	0.040	-0.0240	0.26
289:	11	6	6	11	10	6	5	10	245919.0120	-0.2313	0.040	-0.0240	0.24
290:	11	6	6	12	10	6	5	11	245919.0120	0.1650	0.040	-0.0240	0.26
291:	11	6	5	13	10	6	4	12	245922.0160	0.1225	0.040	-0.0481	0.28
292:	11	6	5	10	10	6	4	9	245922.0160	-0.2731	0.040	-0.0481	0.22
293:	11	6	6	13	10	6	5	12	245922.0160	0.1225	0.040	-0.0481	0.28
294:	11	6	6	10	10	6	5	9	245922.0160	-0.2731	0.040	-0.0481	0.22
295:	11	5	6	11	10	5	5	10	245951.3950	-0.1058	0.040	0.0003	0.24
296:	11	5	6	12	10	5	5	11	245951.3950	0.0970	0.040	0.0003	0.26
297:	11	5	7	11	10	5	6	10	245951.3950	-0.1058	0.040	0.0003	0.24
298:	11	5	7	12	10	5	6	11	245951.3950	0.0970	0.040	0.0003	0.26
299:	11	5	6	13	10	5	5	12	245953.4970	0.0833	0.040	-0.0039	0.28
300:	11	5	6	10	10	5	5	9	245953.4970	-0.1189	0.040	-0.0039	0.22
301:	11	5	7	13	10	5	6	12	245953.4970	0.0833	0.040	-0.0039	0.28
302:	11	5	7	10	10	5	6	9	245953.4970	-0.1189	0.040	-0.0039	0.22

303:	11	2	10	11	10	2	9	10	245975.3970	0.2799	0.040	0.0190	0.24
304:	11	2	10	12	10	2	9	11	245975.3970	0.1131	0.040	0.0190	0.26
305:	11	2	10	13	10	2	9	12	245975.3970	-0.2252	0.040	0.0190	0.29
306:	11	2	10	10	10	2	9	9	245975.3970	-0.0583	0.040	0.0190	0.22
307:	11	4	7	11	10	4	6	10	245980.2720	-0.0142	0.040	0.0118	0.24
308:	11	4	7	12	10	4	6	11	245980.2720	0.0302	0.040	0.0118	0.26
309:	11	4	8	11	10	4	7	10	245980.2720	-0.0087	0.040	0.0118	0.24
310:	11	4	8	12	10	4	7	11	245980.2720	0.0357	0.040	0.0118	0.26
311:	11	4	7	13	10	4	6	12	245981.6360	0.0401	0.040	0.0239	0.28
312:	11	4	7	10	10	4	6	9	245981.6360	-0.0038	0.040	0.0239	0.22
313:	11	4	8	13	10	4	7	12	245981.6360	0.0456	0.040	0.0239	0.28
314:	11	4	8	10	10	4	7	9	245981.6360	0.0016	0.040	0.0239	0.22
315:	11	3	9	11	10	3	8	10	246008.4020	0.0232	0.040	-0.0179	0.48
316:	11	3	9	12	10	3	8	11	246008.4020	-0.0554	0.040	-0.0179	0.52
317:	11	3	9	13	10	3	8	12	246009.1910	-0.0282	0.040	0.0059	0.57
318:	11	3	9	10	10	3	8	9	246009.1910	0.0508	0.040	0.0059	0.43
319:	11	3	8	11	10	3	7	10	246009.9790	0.0499	0.040	0.0088	0.48
320:	11	3	8	12	10	3	7	11	246009.9790	-0.0287	0.040	0.0088	0.52
321:	11	3	8	13	10	3	7	12	246010.7840	0.0144	0.040	0.0485	0.57
322:	11	3	8	10	10	3	7	9	246010.7840	0.0934	0.040	0.0485	0.43
323:	11	2	9	11	10	2	8	10	246139.7870	0.2711	0.040	0.0099	0.24
324:	11	2	9	12	10	2	8	11	246139.7870	0.1044	0.040	0.0099	0.26
325:	11	2	9	13	10	2	8	12	246139.7870	-0.2347	0.040	0.0099	0.29
326:	11	2	9	10	10	2	8	9	246139.7870	-0.0678	0.040	0.0099	0.22
327:	11	1	10	11	10	1	9	10	247563.4430	0.1624	0.040	0.0000	0.24
328:	11	1	10	12	10	1	9	11	247563.4430	-0.0573	0.040	0.0000	0.26
329:	11	1	10	13	10	1	9	12	247563.4430	-0.1418	0.040	0.0000	0.29
330:	11	1	10	10	10	1	9	9	247563.4430	0.0780	0.040	0.0000	0.22
331:	12	1	12	11	11	1	11	10	266568.8130	0.0755	0.040	0.0071	0.22
332:	12	1	12	12	11	1	11	11	266568.8130	0.1403	0.040	0.0071	0.24
333:	12	1	12	13	11	1	11	12	266568.8130	-0.0454	0.040	0.0071	0.26
334:	12	1	12	14	11	1	11	13	266568.8130	-0.1103	0.040	0.0071	0.28
335:	12	11	1	13	11	11	0	12	268005.9010	-0.0132	0.040	-0.0133	0.50
336:	12	11	2	13	11	11	1	12	268005.9010	-0.0132	0.040	-0.0133	0.50
337:	12	11	1	12	11	11	0	11	268007.2240	0.0120	0.040	0.0121	0.50
338:	12	11	2	12	11	11	1	11	268007.2240	0.0120	0.040	0.0121	0.50
339:	12	11	1	14	11	11	0	13	268013.7810	-0.0083	0.040	-0.0083	0.50
340:	12	11	2	14	11	11	1	13	268013.7810	-0.0083	0.040	-0.0083	0.50
341:	12	10	2	13	11	10	1	12	268068.7090	-0.0348	0.040	-0.0349	0.50
342:	12	10	3	13	11	10	2	12	268068.7090	-0.0348	0.040	-0.0349	0.50
343:	12	10	2	12	11	10	1	11	268069.8280	0.0457	0.040	0.0458	0.50
344:	12	10	3	12	11	10	2	11	268069.8280	0.0457	0.040	0.0458	0.50
345:	12	10	2	14	11	10	1	13	268075.2240	-0.0284	0.040	-0.0285	0.50
346:	12	10	3	14	11	10	2	13	268075.2240	-0.0284	0.040	-0.0285	0.50
347:	12	10	2	11	11	10	1	10	268076.3550	0.0636	0.040	0.0637	0.50
348:	12	10	3	11	11	10	2	10	268076.3550	0.0636	0.040	0.0637	0.50
349:	12	9	3	13	11	9	2	12	268125.7600	-0.0575	0.040	-0.0575	0.50
350:	12	9	4	13	11	9	3	12	268125.7600	-0.0575	0.040	-0.0575	0.50
351:	12	9	3	12	11	9	2	11	268126.6810	0.0598	0.040	0.0598	0.50
352:	12	9	4	12	11	9	3	11	268126.6810	0.0598	0.040	0.0598	0.50
353:	12	9	3	14	11	9	2	13	268131.0410	-0.0487	0.040	-0.0487	0.50
354:	12	9	4	14	11	9	3	13	268131.0410	-0.0487	0.040	-0.0487	0.50
355:	12	9	3	11	11	9	2	10	268131.9840	0.0907	0.040	0.0907	0.50
356:	12	9	4	11	11	9	3	10	268131.9840	0.0907	0.040	0.0907	0.50
357:	12	0	12	11	11	0	11	10	268149.4130	0.1199	0.040	0.0119	0.22
358:	12	0	12	12	11	0	11	11	268149.4130	0.1190	0.040	0.0119	0.24
359:	12	0	12	13	11	0	11	12	268149.4130	-0.0794	0.040	0.0119	0.26
360:	12	0	12	14	11	0	11	13	268149.4130	-0.0785	0.040	0.0119	0.28
361:	12	8	4	12	11	8	3	11	268177.4920	-0.2944	0.040	0.0149	0.24
362:	12	8	4	13	11	8	3	12	268177.4920	0.2990	0.040	0.0149	0.26
363:	12	8	5	12	11	8	4	11	268177.4920	-0.2944	0.040	0.0149	0.24
364:	12	8	5	13	11	8	4	12	268177.4920	0.2990	0.040	0.0149	0.26
365:	12	8	4	11	11	8	3	10	268181.5870	-0.3648	0.040	-0.0309	0.22
366:	12	8	4	14	11	8	3	13	268181.5870	0.2282	0.040	-0.0309	0.28
367:	12	8	5	11	11	8	4	10	268181.5870	-0.3648	0.040	-0.0309	0.22
368:	12	8	5	14	11	8	4	13	268181.5870	0.2282	0.040	-0.0309	0.28
369:	12	7	5	12	11	7	4	11	268223.1860	-0.2098	0.040	0.0027	0.24
370:	12	7	5	13	11	7	4	12	268223.1860	0.1981	0.040	0.0027	0.26
371:	12	7	6	12	11	7	5	11	268223.1860	-0.2098	0.040	0.0027	0.24
372:	12	7	6	13	11	7	5	12	268223.1860	0.1981	0.040	0.0027	0.26
373:	12	7	5	11	11	7	4	10	268226.3430	-0.2419	0.040	-0.0125	0.22
374:	12	7	5	14	11	7	4	13	268226.3430	0.1655	0.040	-0.0125	0.28
375:	12	7	6	11	11	7	5	10	268226.3430	-0.2419	0.040	-0.0125	0.22
376:	12	7	6	14	11	7	5	13	268226.3430	0.1655	0.040	-0.0125	0.28
377:	12	6	6	12	11	6	5	11	268263.5630	-0.1306	0.040	-0.0018	0.24
378:	12	6	6	13	11	6	5	12	268263.5630	0.1165	0.040	-0.0018	0.26
379:	12	6	7	12	11	6	6	11	268263.5630	-0.1306	0.040	-0.0018	0.24
380:	12	6	7	13	11	6	6	12	268263.5630	0.1165	0.040	-0.0018	0.26

381:	12	6	6	11	11	6	5	10	268265.8930	-0.1435	0.040	-0.0047	0.22
382:	12	6	6	14	11	6	5	13	268265.8930	0.1031	0.040	-0.0047	0.28
383:	12	6	7	11	11	6	6	10	268265.8930	-0.1435	0.040	-0.0047	0.22
384:	12	6	7	14	11	6	6	13	268265.8930	0.1031	0.040	-0.0047	0.28
385:	12	5	7	12	11	5	6	11	268299.1650	-0.0607	0.040	-0.0029	0.24
386:	12	5	7	13	11	5	6	12	268299.1650	0.0503	0.040	-0.0029	0.26
387:	12	5	8	12	11	5	7	11	268299.1650	-0.0607	0.040	-0.0029	0.24
388:	12	5	8	13	11	5	7	12	268299.1650	0.0503	0.040	-0.0029	0.26
389:	12	5	7	11	11	5	6	10	268300.7940	-0.0587	0.040	0.0035	0.22
390:	12	5	7	14	11	5	6	13	268300.7940	0.0518	0.040	0.0035	0.28
391:	12	5	8	11	11	5	7	10	268300.7940	-0.0587	0.040	0.0035	0.22
392:	12	5	8	14	11	5	7	13	268300.7940	0.0519	0.040	0.0035	0.28
393:	12	2	11	11	11	2	10	10	268317.8360	-0.0537	0.040	-0.0048	0.22
394:	12	2	11	12	11	2	10	11	268317.8360	0.2064	0.040	-0.0048	0.24
395:	12	2	11	13	11	2	10	12	268317.8360	0.0575	0.040	-0.0048	0.26
396:	12	2	11	14	11	2	10	13	268317.8360	-0.2027	0.040	-0.0048	0.28
397:	12	4	8	12	11	4	7	11	268331.3640	-0.0386	0.040	-0.0337	0.24
398:	12	4	8	13	11	4	7	12	268331.3640	-0.0389	0.040	-0.0337	0.26
399:	12	4	9	12	11	4	8	11	268331.3640	-0.0283	0.040	-0.0337	0.24
400:	12	4	9	13	11	4	8	12	268331.3640	-0.0286	0.040	-0.0337	0.26
401:	12	4	8	11	11	4	7	10	268332.4980	0.0540	0.040	0.0588	0.22
402:	12	4	8	14	11	4	7	13	268332.4980	0.0533	0.040	0.0588	0.28
403:	12	4	9	11	11	4	8	10	268332.4980	0.0643	0.040	0.0588	0.22
404:	12	4	9	14	11	4	8	13	268332.4980	0.0636	0.040	0.0588	0.28
405:	12	3	10	12	11	3	9	11	268363.7290	0.0978	0.040	0.0525	0.48
406:	12	3	10	13	11	3	9	12	268363.7290	0.0109	0.040	0.0525	0.52
407:	12	3	10	11	11	3	9	10	268364.2580	0.0410	0.040	-0.0080	0.44
408:	12	3	10	14	11	3	9	13	268364.2580	-0.0461	0.040	-0.0080	0.56
409:	12	3	9	12	11	3	8	11	268366.1340	0.0922	0.040	0.0469	0.48
410:	12	3	9	13	11	3	8	12	268366.1340	0.0052	0.040	0.0469	0.52
411:	12	3	9	11	11	3	8	10	268366.6650	0.0374	0.040	-0.0117	0.44
412:	12	3	9	14	11	3	8	13	268366.6650	-0.0497	0.040	-0.0117	0.56
413:	12	2	10	11	11	2	9	10	268531.2940	-0.0543	0.040	-0.0050	0.22
414:	12	2	10	12	11	2	9	11	268531.2940	0.2066	0.040	-0.0050	0.24
415:	12	2	10	13	11	2	9	12	268531.2940	0.0578	0.040	-0.0050	0.26
416:	12	2	10	14	11	2	9	13	268531.2940	-0.2033	0.040	-0.0050	0.28
417:	12	1	11	11	11	1	10	10	270046.7170	0.0873	0.040	0.0187	0.22
418:	12	1	11	12	11	1	10	11	270046.7170	0.1522	0.040	0.0187	0.24
419:	12	1	11	13	11	1	10	12	270046.7170	-0.0340	0.040	0.0187	0.26
420:	12	1	11	14	11	1	10	13	270046.7170	-0.0989	0.040	0.0187	0.28
421:	13	1	13	12	12	1	12	11	288758.0400	0.0758	0.040	0.0153	0.22
422:	13	1	13	13	12	1	12	12	288758.0400	0.1267	0.040	0.0153	0.24
423:	13	1	13	14	12	1	12	13	288758.0400	-0.0326	0.040	0.0153	0.26
424:	13	1	13	15	12	1	12	14	288758.0400	-0.0836	0.040	0.0153	0.28
425:	13	12	1	14	12	12	0	13	290251.7830	-0.0317	0.040	-0.0318	0.50
426:	13	12	2	14	12	12	1	13	290251.7830	-0.0317	0.040	-0.0318	0.50
427:	13	12	1	13	12	12	0	12	290252.9590	0.0240	0.040	0.0241	0.50
428:	13	12	2	13	12	12	1	12	290252.9590	0.0240	0.040	0.0241	0.50
429:	13	12	1	15	12	12	0	14	290259.1730	-0.0054	0.040	-0.0054	0.50
430:	13	12	2	15	12	12	1	14	290259.1730	-0.0054	0.040	-0.0054	0.50
431:	13	12	1	12	12	12	0	11	290260.3480	0.0480	0.040	0.0481	0.50
432:	13	12	2	12	12	12	1	11	290260.3480	0.0480	0.040	0.0481	0.50
433:	13	11	2	14	12	11	1	13	290325.8860	-0.0391	0.040	-0.0391	0.50
434:	13	11	3	14	12	11	2	13	290325.8860	-0.0391	0.040	-0.0391	0.50
435:	13	11	2	13	12	11	1	12	290326.8830	0.0432	0.040	0.0432	0.50
436:	13	11	3	13	12	11	2	12	290326.8830	0.0432	0.040	0.0432	0.50
437:	13	11	2	15	12	11	1	14	290332.0770	-0.0359	0.040	-0.0359	0.50
438:	13	11	3	15	12	11	2	14	290332.0770	-0.0359	0.040	-0.0359	0.50
439:	13	11	2	12	12	11	1	11	290333.0870	0.0588	0.040	0.0589	0.50
440:	13	11	3	12	12	11	2	11	290333.0870	0.0588	0.040	0.0589	0.50
441:	13	10	3	14	12	10	2	13	290393.7880	-0.0247	0.040	-0.0247	0.50
442:	13	10	4	14	12	10	3	13	290393.7880	-0.0247	0.040	-0.0247	0.50
443:	13	10	3	13	12	10	2	12	290394.5810	0.0413	0.040	0.0414	0.50
444:	13	10	4	13	12	10	3	12	290394.5810	0.0413	0.040	0.0414	0.50
445:	13	10	3	15	12	10	2	14	290398.9100	-0.0168	0.040	-0.0168	0.50
446:	13	10	4	15	12	10	3	14	290398.9100	-0.0168	0.040	-0.0168	0.50
447:	13	10	3	12	12	10	2	11	290399.7170	0.0632	0.040	0.0633	0.50
448:	13	10	4	12	12	10	3	11	290399.7170	0.0632	0.040	0.0633	0.50
449:	13	0	13	12	12	0	12	11	290439.5390	0.1037	0.040	0.0124	0.22
450:	13	0	13	13	12	0	12	12	290439.5390	0.1027	0.040	0.0124	0.24
451:	13	0	13	14	12	0	12	13	290439.5390	-0.0657	0.040	0.0124	0.26
452:	13	0	13	15	12	0	12	14	290439.5390	-0.0648	0.040	0.0124	0.28
453:	13	9	4	13	12	9	3	12	290455.7500	-0.3210	0.040	-0.0318	0.24
454:	13	9	4	14	12	9	3	13	290455.7500	0.2358	0.040	-0.0318	0.26
455:	13	9	5	13	12	9	4	12	290455.7500	-0.3210	0.040	-0.0318	0.24
456:	13	9	5	14	12	9	4	13	290455.7500	0.2358	0.040	-0.0318	0.26
457:	13	9	4	12	12	9	3	11	290459.8400	-0.3733	0.040	-0.0627	0.22
458:	13	9	4	15	12	9	3	14	290459.8400	0.1833	0.040	-0.0627	0.28

459:	13	9	5	12	12	9	4	11	290459.8400	-0.3733	0.040	-0.0627	0.22
460:	13	9	5	15	12	9	4	14	290459.8400	0.1833	0.040	-0.0627	0.28
461:	13	8	5	13	12	8	4	12	290511.3030	-0.2075	0.040	0.0027	0.24
462:	13	8	5	14	12	8	4	13	290511.3030	0.1972	0.040	0.0027	0.26
463:	13	8	6	13	12	8	5	12	290511.3030	-0.2075	0.040	0.0027	0.24
464:	13	8	6	14	12	8	5	13	290511.3030	0.1972	0.040	0.0027	0.26
465:	13	8	5	12	12	8	4	11	290514.5470	-0.2363	0.040	-0.0107	0.22
466:	13	8	5	15	12	8	4	14	290514.5470	0.1680	0.040	-0.0107	0.28
467:	13	8	6	12	12	8	5	11	290514.5470	-0.2363	0.040	-0.0107	0.22
468:	13	8	6	15	12	8	5	14	290514.5470	0.1680	0.040	-0.0107	0.28
469:	13	7	6	13	12	7	5	12	290560.8550	-0.1555	0.040	-0.0150	0.24
470:	13	7	6	14	12	7	5	13	290560.8550	0.1149	0.040	-0.0150	0.26
471:	13	7	7	13	12	7	6	12	290560.8550	-0.1555	0.040	-0.0150	0.24
472:	13	7	7	14	12	7	6	13	290560.8550	0.1149	0.040	-0.0150	0.26
473:	13	7	6	12	12	7	5	11	290563.3300	-0.1861	0.040	-0.0355	0.22
474:	13	7	6	15	12	7	5	14	290563.3300	0.0838	0.040	-0.0355	0.28
475:	13	7	7	12	12	7	6	11	290563.3300	-0.1861	0.040	-0.0355	0.22
476:	13	7	7	15	12	7	6	14	290563.3300	0.0838	0.040	-0.0355	0.28
477:	13	6	7	13	12	6	6	12	290604.7910	-0.0928	0.040	-0.0128	0.24
478:	13	6	7	14	12	6	6	13	290604.7910	0.0612	0.040	-0.0128	0.26
479:	13	6	8	13	12	6	7	12	290604.7910	-0.0928	0.040	-0.0128	0.24
480:	13	6	8	14	12	6	7	13	290604.7910	0.0612	0.040	-0.0128	0.26
481:	13	6	7	12	12	6	6	11	290606.6350	-0.0896	0.040	-0.0040	0.22
482:	13	6	7	15	12	6	6	14	290606.6350	0.0639	0.040	-0.0040	0.28
483:	13	6	8	12	12	6	7	11	290606.6350	-0.0896	0.040	-0.0040	0.22
484:	13	6	8	15	12	6	7	14	290606.6350	0.0639	0.040	-0.0040	0.28
485:	13	5	8	13	12	5	7	12	290643.7740	-0.0526	0.040	-0.0237	0.24
486:	13	5	8	14	12	5	7	13	290643.7740	0.0029	0.040	-0.0237	0.26
487:	13	5	9	13	12	5	8	12	290643.7740	-0.0525	0.040	-0.0237	0.24
488:	13	5	9	14	12	5	8	13	290643.7740	0.0029	0.040	-0.0237	0.26
489:	13	5	8	12	12	5	7	11	290645.0900	-0.0149	0.040	0.0158	0.22
490:	13	5	8	15	12	5	7	14	290645.0900	0.0402	0.040	0.0158	0.28
491:	13	5	9	12	12	5	8	11	290645.0900	-0.0149	0.040	0.0158	0.22
492:	13	5	9	15	12	5	8	14	290645.0900	0.0402	0.040	0.0158	0.28
493:	13	2	12	12	12	2	11	11	290655.3730	-0.0239	0.040	0.0064	0.22
494:	13	2	12	13	12	2	11	12	290655.3730	0.1804	0.040	0.0064	0.24
495:	13	2	12	14	12	2	11	13	290655.3730	0.0477	0.040	0.0064	0.26
496:	13	2	12	15	12	2	11	14	290655.3730	-0.1567	0.040	0.0064	0.28
497:	13	4	9	13	12	4	8	12	290679.5900	-0.0463	0.040	-0.0502	0.24
498:	13	4	9	14	12	4	8	13	290679.5900	-0.0714	0.040	-0.0502	0.26
499:	13	4	10	13	12	4	9	12	290679.5900	-0.0280	0.040	-0.0502	0.24
500:	13	4	10	14	12	4	9	13	290679.5900	-0.0531	0.040	-0.0502	0.26
501:	13	4	9	12	12	4	8	11	290680.5020	0.0475	0.040	0.0425	0.22
502:	13	4	9	15	12	4	8	14	290680.5020	0.0221	0.040	0.0425	0.28
503:	13	4	10	12	12	4	9	11	290680.5020	0.0657	0.040	0.0425	0.22
504:	13	4	10	15	12	4	9	14	290680.5020	0.0404	0.040	0.0425	0.28
505:	13	3	11	12	12	3	10	11	290716.6280	-0.1971	0.040	-0.0151	0.22
506:	13	3	11	13	12	3	10	12	290716.6280	0.2631	0.040	-0.0151	0.24
507:	13	3	11	14	12	3	10	13	290716.6280	0.1753	0.040	-0.0151	0.26
508:	13	3	11	15	12	3	10	14	290716.6280	-0.2851	0.040	-0.0151	0.28
509:	13	3	10	12	12	3	9	11	290720.2550	-0.1844	0.040	-0.0023	0.22
510:	13	3	10	13	12	3	9	12	290720.2550	0.2759	0.040	-0.0023	0.24
511:	13	3	10	14	12	3	9	13	290720.2550	0.1880	0.040	-0.0023	0.26
512:	13	3	10	15	12	3	9	14	290720.2550	-0.2724	0.040	-0.0023	0.28
513:	13	2	11	12	12	2	10	11	290926.6500	-0.0087	0.040	0.0221	0.22
514:	13	2	11	13	12	2	10	12	290926.6500	0.1965	0.040	0.0221	0.24
515:	13	2	11	14	12	2	10	13	290926.6500	0.0639	0.040	0.0221	0.26
516:	13	2	11	15	12	2	10	14	290926.6500	-0.1414	0.040	0.0221	0.28
517:	13	1	12	12	12	1	11	11	292524.0200	0.0762	0.040	0.0156	0.22
518:	13	1	12	13	12	1	11	12	292524.0200	0.1271	0.040	0.0156	0.24
519:	13	1	12	14	12	1	11	13	292524.0200	-0.0325	0.040	0.0156	0.26
520:	13	1	12	15	12	1	11	14	292524.0200	-0.0834	0.040	0.0156	0.28
521:	14	1	14	13	13	1	13	12	310941.3940	0.0678	0.040	0.0141	0.22
522:	14	1	14	14	13	1	13	13	310941.3940	0.1085	0.040	0.0141	0.24
523:	14	1	14	15	13	1	13	14	310941.3940	-0.0295	0.040	0.0141	0.26
524:	14	1	14	16	13	1	13	15	310941.3940	-0.0702	0.040	0.0141	0.28
525:	14	12	2	15	13	12	1	14	312562.2980	-0.0355	0.040	-0.0355	0.50
526:	14	12	3	15	13	12	2	14	312562.2980	-0.0355	0.040	-0.0355	0.50
527:	14	12	2	14	13	12	1	13	312563.1980	0.0533	0.040	0.0533	0.50
528:	14	12	3	14	13	12	2	13	312563.1980	0.0533	0.040	0.0533	0.50
529:	14	12	2	16	13	12	1	15	312568.1970	-0.0277	0.040	-0.0277	0.50
530:	14	12	3	16	13	12	2	15	312568.1970	-0.0277	0.040	-0.0277	0.50
531:	14	12	2	13	13	12	1	12	312569.1120	0.0756	0.040	0.0756	0.50
532:	14	12	3	13	13	12	2	12	312569.1120	0.0756	0.040	0.0756	0.50
533:	14	11	3	15	13	11	2	14	312642.0280	0.0204	0.040	0.0204	0.50
534:	14	11	4	15	13	11	3	14	312642.0280	0.0204	0.040	0.0204	0.50
535:	14	11	3	14	13	11	2	13	312642.6450	-0.0212	0.040	-0.0212	0.50
536:	14	11	4	14	13	11	3	13	312642.6450	-0.0212	0.040	-0.0212	0.50

537:	14	11	3	16	13	11	2	15	312646.9870	0.0290	0.040	0.0290	0.50
538:	14	11	4	16	13	11	3	15	312646.9870	0.0290	0.040	0.0290	0.50
539:	14	11	3	13	13	11	2	12	312647.6190	0.0023	0.040	0.0023	0.50
540:	14	11	4	13	13	11	3	12	312647.6190	0.0023	0.040	0.0023	0.50
541:	14	0	14	13	13	0	13	12	312716.5200	0.0847	0.040	0.0065	0.22
542:	14	0	14	14	13	0	13	13	312716.5200	0.0837	0.040	0.0065	0.24
543:	14	0	14	15	13	0	13	14	312716.5200	-0.0612	0.040	0.0065	0.26
544:	14	0	14	16	13	0	13	15	312716.5200	-0.0602	0.040	0.0065	0.28
545:	14	9	5	14	13	9	4	13	312781.5910	-0.2155	0.040	-0.0118	0.24
546:	14	9	5	15	13	9	4	14	312781.5910	0.1777	0.040	-0.0118	0.26
547:	14	9	6	14	13	9	5	13	312781.5910	-0.2155	0.040	-0.0118	0.24
548:	14	9	6	15	13	9	5	14	312781.5910	0.1777	0.040	-0.0118	0.26
549:	14	9	5	13	13	9	4	12	312784.8900	-0.2302	0.040	-0.0127	0.22
550:	14	9	5	16	13	9	4	15	312784.8900	0.1626	0.040	-0.0127	0.28
551:	14	9	6	13	13	9	5	12	312784.8900	-0.2302	0.040	-0.0127	0.22
552:	14	9	6	16	13	9	5	15	312784.8900	0.1626	0.040	-0.0127	0.28
553:	14	8	6	14	13	8	5	13	312841.4280	-0.1446	0.040	0.0006	0.24
554:	14	8	6	15	13	8	5	14	312841.4280	0.1356	0.040	0.0006	0.26
555:	14	8	7	14	13	8	6	13	312841.4280	-0.1446	0.040	0.0006	0.24
556:	14	8	7	15	13	8	6	14	312841.4280	0.1356	0.040	0.0006	0.26
557:	14	8	6	13	13	8	5	12	312844.0360	-0.1548	0.040	0.0001	0.22
558:	14	8	6	16	13	8	5	15	312844.0360	0.1250	0.040	0.0001	0.28
559:	14	8	7	13	13	8	6	12	312844.0360	-0.1548	0.040	0.0001	0.22
560:	14	8	7	16	13	8	6	15	312844.0360	0.1250	0.040	0.0001	0.28
561:	14	7	7	14	13	7	6	13	312894.9310	-0.0978	0.040	-0.0042	0.24
562:	14	7	7	15	13	7	6	14	312894.9310	0.0829	0.040	-0.0042	0.26
563:	14	7	8	14	13	7	7	13	312894.9310	-0.0978	0.040	-0.0042	0.24
564:	14	7	8	15	13	7	7	14	312894.9310	0.0829	0.040	-0.0042	0.26
565:	14	7	7	13	13	7	6	12	312896.9350	-0.0983	0.040	0.0015	0.22
566:	14	7	7	16	13	7	6	15	312896.9350	0.0819	0.040	0.0015	0.28
567:	14	7	8	13	13	7	7	12	312896.9350	-0.0983	0.040	0.0015	0.22
568:	14	7	8	16	13	7	7	15	312896.9350	0.0819	0.040	0.0015	0.28
569:	14	6	8	14	13	6	7	13	312942.5110	-0.0569	0.040	-0.0080	0.24
570:	14	6	8	15	13	6	7	14	312942.5110	0.0374	0.040	-0.0080	0.26
571:	14	6	9	14	13	6	8	13	312942.5110	-0.0569	0.040	-0.0080	0.24
572:	14	6	9	15	13	6	8	14	312942.5110	0.0374	0.040	-0.0080	0.26
573:	14	6	8	13	13	6	7	12	312944.0050	-0.0356	0.040	0.0164	0.22
574:	14	6	8	16	13	6	7	15	312944.0050	0.0583	0.040	0.0164	0.28
575:	14	6	9	13	13	6	8	12	312944.0050	-0.0356	0.040	0.0164	0.22
576:	14	6	9	16	13	6	8	15	312944.0050	0.0583	0.040	0.0164	0.28
577:	14	5	9	14	13	5	8	13	312985.0160	-0.0454	0.040	-0.0344	0.24
578:	14	5	9	15	13	5	8	14	312985.0160	-0.0241	0.040	-0.0344	0.26
579:	14	5	10	14	13	5	9	13	312985.0160	-0.0453	0.040	-0.0344	0.24
580:	14	5	10	15	13	5	9	14	312985.0160	-0.0240	0.040	-0.0344	0.26
581:	14	5	9	13	13	5	8	12	312986.0970	0.0128	0.040	0.0245	0.22
582:	14	5	9	16	13	5	8	15	312986.0970	0.0338	0.040	0.0245	0.28
583:	14	5	10	13	13	5	9	12	312986.0970	0.0129	0.040	0.0245	0.22
584:	14	5	10	16	13	5	9	15	312986.0970	0.0339	0.040	0.0245	0.28
585:	14	2	13	13	13	2	12	12	312987.5480	-0.0158	0.040	0.0022	0.22
586:	14	2	13	14	13	2	12	13	312987.5480	0.1476	0.040	0.0022	0.24
587:	14	2	13	15	13	2	12	14	312987.5480	0.0293	0.040	0.0022	0.26
588:	14	2	13	16	13	2	12	15	312987.5480	-0.1342	0.040	0.0022	0.28
589:	14	4	10	14	13	4	9	13	313024.8040	0.0441	0.040	0.0397	0.24
590:	14	4	10	15	13	4	9	14	313024.8040	0.0055	0.040	0.0397	0.26
591:	14	4	11	14	13	4	10	13	313024.8040	0.0751	0.040	0.0397	0.24
592:	14	4	11	15	13	4	10	14	313024.8040	0.0366	0.040	0.0397	0.26
593:	14	4	10	13	13	4	9	12	313025.3640	-0.0504	0.040	-0.0564	0.22
594:	14	4	10	16	13	4	9	15	313025.3640	-0.0892	0.040	-0.0564	0.28
595:	14	4	11	13	13	4	10	12	313025.3640	-0.0193	0.040	-0.0564	0.22
596:	14	4	11	16	13	4	10	15	313025.3640	-0.0581	0.040	-0.0564	0.28
597:	14	3	12	13	13	3	11	12	313066.5820	-0.1321	0.040	0.0059	0.22
598:	14	3	12	14	13	3	11	13	313066.5820	0.2361	0.040	0.0059	0.24
599:	14	3	12	15	13	3	11	14	313066.5820	0.1510	0.040	0.0059	0.26
600:	14	3	12	16	13	3	11	15	313066.5820	-0.2173	0.040	0.0059	0.28
601:	14	3	11	13	13	3	10	12	313071.8230	-0.1453	0.040	-0.0073	0.22
602:	14	3	11	14	13	3	10	13	313071.8230	0.2230	0.040	-0.0073	0.24
603:	14	3	11	15	13	3	10	14	313071.8230	0.1379	0.040	-0.0073	0.26
604:	14	3	11	16	13	3	10	15	313071.8230	-0.2304	0.040	-0.0073	0.28
605:	14	2	12	13	13	2	11	12	313326.0200	0.0114	0.040	0.0300	0.22
606:	14	2	12	14	13	2	11	13	313326.0200	0.1758	0.040	0.0300	0.24
607:	14	2	12	15	13	2	11	14	313326.0200	0.0576	0.040	0.0300	0.26
608:	14	2	12	16	13	2	11	15	313326.0200	-0.1069	0.040	0.0300	0.28
609:	14	1	13	13	13	1	12	12	314994.8400	0.0595	0.040	0.0056	0.22
610:	14	1	13	14	13	1	12	13	314994.8400	0.1001	0.040	0.0056	0.24
611:	14	1	13	15	13	1	12	14	314994.8400	-0.0382	0.040	0.0056	0.26
612:	14	1	13	16	13	1	12	15	314994.8400	-0.0789	0.040	0.0056	0.28

```
PARAMETERS IN FIT (values truncated and Nlines statistics):

       10000            A  /MHz        95358.5(55)                          1
       20000            B  /MHz        11330.5476(88)                       2
       30000            C  /MHz        11039.9541(88)                       3
         200      Delta_J  /kHz           12.0282(37)                       4
        1100     Delta_JK  /kHz          122.738(21)                        5
        2000      Delta_K  /kHz         [928.936]                           6
       40100      delta_J  /kHz            0.2827(30)                       7
       41000      delta_K  /kHz           60.7(43)                          8
         300        Phi_J  /Hz           [-0.002656]                        9
        1200       Phi_JK  /Hz           [ 0.306]                          10
        2100       Phi_KJ  /Hz           [ 2.655]                          11
        3000        Phi_K  /Hz           [32.]                             12
       40200        phi_J  /Hz           [ 0.000128]                       13
       41100       phi_JK  /Hz           [ 0.248]                          14
       42000        phi_K  /Hz           [23.]                             15
   110010000         X_aa  /MHz          -74.461(35)                       16
  -110030000         X_cc  /MHz           74.461(35)          = -1.00000 * 16
   110020000         X_bb  /MHz           37.307(70)                       17
  -110030000         X_cc  /MHz          -37.307(70)          = -1.00000 * 17

 MICROWAVE AVG =       -0.000531 MHz, IR AVG =        0.00000
 MICROWAVE RMS =        0.033418 MHz, IR RMS =        0.00000
 END OF ITERATION  1 OLD, NEW RMS ERROR=        0.88846         0.88846

  distinct frequency lines in fit:   221
       distinct parameters of fit:     9

                                  upper state  lower state         overall
      limits of quantum number  1:      4   14       3   13           3   14
      limits of quantum number  2:      0   12       0   12           0   12
      limits of quantum number  3:      1   14       0   13           0   14
      limits of quantum number  4:      3   16       2   15           2   16

                 frequency range:       88881       314994


 PARAMETERS IN FIT WITH STANDARD ERRORS ON THOSE THAT ARE FITTED:
 (values rounded and degrees of freedom, Ndegf=Nlines-Nconst, statistics)

       10000            A  /MHz        95358.5(50)                          1
       20000            B  /MHz        11330.5476(80)                       2
       30000            C  /MHz        11039.9541(80)                       3
         200      Delta_J  /kHz           12.0282(34)                       4
        1100     Delta_JK  /kHz          122.738(19)                        5
        2000      Delta_K  /kHz         [928.936]                           6
       40100      delta_J  /kHz            0.2827(27)                       7
       41000      delta_K  /kHz           60.7(39)                          8
         300        Phi_J  /Hz           [-0.002656]                        9
        1200       Phi_JK  /Hz           [ 0.306]                          10
        2100       Phi_KJ  /Hz           [ 2.655]                          11
        3000        Phi_K  /Hz           [32.]                             12
       40200        phi_J  /Hz           [ 0.000128]                       13
       41100       phi_JK  /Hz           [ 0.248]                          14
       42000        phi_K  /Hz           [23.]                             15
   110010000         X_aa  /MHz          -74.461(32)                       16
  -110030000         X_cc  /MHz           74.461(32)          = -1.00000 * 16
   110020000         X_bb  /MHz           37.307(63)                       17
  -110030000         X_cc  /MHz          -37.307(63)          = -1.00000 * 17

 CORRELATION COEFFICIENTS, C.ij:

           A        B        C        -Delta_J -Delta_J -delta_J -delta_K X_aa

A           1.0000
B          -0.5199   1.0000
C           0.5219  -0.9968   1.0000
-Delta_J    0.4904  -0.9366   0.9132   1.0000
-Delta_JK  -0.5235   0.9747  -0.9757  -0.9454   1.0000
-delta_J    0.0559  -0.1745   0.1708   0.0469  -0.0337   1.0000
-delta_K    0.5385  -0.9849   0.9857   0.9355  -0.9898   0.0346   1.0000
X_aa        0.0023   0.0048  -0.0033  -0.0020  -0.0034  -0.0356   0.0034   1.0000
X_bb       -0.0062  -0.0148   0.0133   0.0039   0.0020   0.0760  -0.0019  -0.6103

           X_bb

X_bb        1.0000
```

```
Mean value of |C.ij|, i.ne.j =   0.3759
Mean value of  C.ij,  i.ne.j =  -0.0551


Worst correlations, with absolute value greater than 0.9950:

      30000  C         <->       20000  B            -0.996809


Worst fitted lines (obs-calc/error):

  273:   -2.6         65:    2.6        209:   -2.4        155:   -2.3
  355:    2.3        117:   -2.2         21:   -2.1          7:    2.0
  531:    1.9         81:    1.7        131:   -1.6          1:    1.6
  201:    1.6        115:   -1.6        347:    1.6        447:    1.6
  457:   -1.6        351:    1.5         41:   -1.5         61:   -1.5
  439:    1.5         37:    1.5        401:    1.5         57:   -1.5
  231:   -1.5        113:    1.5        127:   -1.4        349:   -1.4
  593:   -1.4        163:    1.4        111:    1.4         79:    1.4
   34:    1.4        527:    1.3        405:    1.3        103:    1.3
    6:   -1.3        497:   -1.3        353:   -1.2         25:   -1.2
  321:    1.2        431:    1.2        291:   -1.2        121:   -1.2
  409:    1.2         13:    1.2        343:    1.1        177:   -1.1
  153:   -1.1        271:   -1.1

  273: 11  8  3 10   10  8  2  9             245845.9510  -0.1054  0.040  -0.1054 0.50
   65:  8  0  8  7    7  0  7  6             178876.5560   0.3778  0.040   0.1053 0.18
  209: 10  6  4  9    9  6  3  8             223575.7340  -0.0955  0.040  -0.0955 0.50
  155:  9  3  6  9    8  3  5  8             201291.3880  -0.3694  0.040  -0.0916 0.24
  355: 12  9  3 11   11  9  2 10             268131.9840   0.0907  0.040   0.0907 0.50
  117:  9  7  2  9    8  7  1  8             201191.2550  -0.0896  0.040  -0.0897 0.50
   21:  4  0  4  5    3  0  3  4              89472.0167   0.0226  0.020  -0.0416 0.34
    7:  4  1  4  3    3  1  3  3              88909.9442   0.0392  0.020
  531: 14 12  2 13   13 12  1 12             312569.1120   0.0756  0.040   0.0756 0.50
   81:  8  4  4  8    7  4  3  7             178911.8810   0.0659  0.040   0.0663 0.50
                                          _____________________________________
_________________________________________/ SPFIT output reformatted with PIFORM
```

Table S.4. Fit of the rotational transitions of $^{13}CHD_2{}^{37}Cl$ in PIFORM format

13CHD2Cl -- 37Cl   Tue Mar 18 09:44:55 2025

/ instead of : below denotes (o-c)>3*err

									obs	o-c	error	blends o-c	blends wt	Notes
1:	4	1	4	4	3	1	3	3	87382.6597	-0.0125	0.020			
2:	4	1	4	5	3	1	3	4	87383.4362	0.0106	0.020			
3:	4	1	4	3	3	1	3	2	87384.1273	-0.0131	0.020			
4:	4	1	4	6	3	1	3	5	87384.9109	0.0162	0.020			
5:	4	1	4	4	3	1	3	4	87387.7713	-0.0035	0.020			
6:	4	3	1	5	3	3	0	4	87931.1715	0.0164	0.020	0.0197	0.50	
7:	4	3	2	5	3	3	1	4	87931.1715	0.0229	0.020	0.0197	0.50	
8:	4	3	1	4	3	3	0	3	87937.9193	-0.0005	0.020	0.0027	0.50	
9:	4	3	2	4	3	3	1	3	87937.9193	0.0059	0.020	0.0027	0.50	
10:	4	0	4	3	3	0	3	2	87939.1044	0.0549	0.020	-0.0014	0.28	
11:	4	0	4	4	3	0	3	3	87939.1044	0.0548	0.020	-0.0014	0.37	
12:	4	2	3	5	3	2	2	4	87939.1044	-0.1055	0.020	-0.0014	0.35	
13:	4	0	4	5	3	0	3	4	87940.7590	0.0046	0.020	0.0050	0.43	
14:	4	0	4	6	3	0	3	5	87940.7590	0.0051	0.020	0.0050	0.57	
15:	4	3	1	6	3	3	0	5	87944.3759	0.0029	0.020	0.0062	0.50	
16:	4	3	2	6	3	3	1	5	87944.3759	0.0094	0.020	0.0062	0.50	
17:	4	2	3	6	3	2	2	5	87945.1067	0.0207	0.020			
18:	4	2	2	5	3	2	1	4	87946.1664	-0.0250	0.020			
19:	4	2	3	3	3	2	2	2	87947.1648	0.0204	0.020			
20:	4	2	2	4	3	2	1	3	87948.2734	0.0205	0.020			
21:	4	3	1	3	3	3	0	2	87951.1208	-0.0195	0.020	-0.0163	0.50	
22:	4	3	2	3	3	3	1	2	87951.1208	-0.0130	0.020	-0.0163	0.50	
23:	4	2	2	6	3	2	1	5	87952.0796	0.0114	0.020			
24:	4	2	2	3	3	2	1	2	87954.1153	-0.0113	0.020			
25:	4	1	3	4	3	1	2	3	88504.4866	-0.0123	0.020			
26:	4	1	3	5	3	1	2	4	88505.2688	-0.0028	0.020			
27:	4	1	3	3	3	1	2	2	88505.9614	-0.0056	0.020			
28:	4	1	3	6	3	1	2	5	88506.7435	0.0027	0.020			
29:	8	1	8	7	7	1	7	6	174739.5820	0.0791	0.040	-0.0090	0.20	
30:	8	1	8	8	7	1	7	7	174739.5820	0.2536	0.040	-0.0090	0.23	
31:	8	1	8	9	7	1	7	8	174739.5820	-0.0560	0.040	-0.0090	0.26	
32:	8	1	8	10	7	1	7	9	174739.5820	-0.2306	0.040	-0.0090	0.30	
33:	8	7	1	9	7	7	0	8	175780.4500	0.0068	0.040	0.0069	0.50	
34:	8	7	2	9	7	7	1	8	175780.4500	0.0068	0.040	0.0069	0.50	
35:	8	7	1	8	7	7	0	7	175782.6150	0.0503	0.040	0.0504	0.50	
36:	8	7	2	8	7	7	1	7	175782.6150	0.0503	0.040	0.0504	0.50	
37:	8	7	1	10	7	7	0	9	175789.0190	0.0085	0.040	0.0086	0.50	
38:	8	7	2	10	7	7	1	9	175789.0190	0.0085	0.040	0.0086	0.50	
39:	8	7	1	7	7	7	0	6	175791.0820	-0.0514	0.040	-0.0514	0.50	
40:	8	7	2	7	7	7	1	6	175791.0820	-0.0514	0.040	-0.0514	0.50	
41:	8	6	2	9	7	6	1	8	175807.0630	0.0051	0.040	0.0051	0.50	
42:	8	6	3	9	7	6	2	8	175807.0630	0.0051	0.040	0.0051	0.50	
43:	8	6	2	8	7	6	1	7	175808.5040	-0.0169	0.040	-0.0169	0.50	
44:	8	6	3	8	7	6	2	7	175808.5040	-0.0169	0.040	-0.0169	0.50	
45:	8	6	2	10	7	6	1	9	175813.3370	-0.0157	0.040	-0.0157	0.50	
46:	8	6	3	10	7	6	2	9	175813.3370	-0.0157	0.040	-0.0157	0.50	
47:	8	6	2	7	7	6	1	6	175814.8070	-0.0087	0.040	-0.0088	0.50	
48:	8	6	3	7	7	6	2	6	175814.8070	-0.0087	0.040	-0.0088	0.50	
49:	8	0	8	7	7	0	7	6	175818.1070	0.2233	0.040	0.0181	0.20	
50:	8	0	8	8	7	0	7	7	175818.1070	0.2220	0.040	0.0181	0.23	
51:	8	0	8	9	7	0	7	8	175818.1070	-0.1406	0.040	0.0181	0.26	
52:	8	0	8	10	7	0	7	9	175818.1070	-0.1393	0.040	0.0181	0.30	
53:	8	5	3	9	7	5	2	8	175830.0010	0.0199	0.040	0.0200	0.50	
54:	8	5	4	9	7	5	3	8	175830.0010	0.0199	0.040	0.0200	0.50	
55:	8	5	3	8	7	5	2	7	175830.8940	0.0073	0.040	0.0073	0.50	
56:	8	5	4	8	7	5	3	7	175830.8940	0.0073	0.040	0.0073	0.50	
57:	8	5	3	10	7	5	2	9	175834.3300	-0.0227	0.040	-0.0228	0.50	
58:	8	5	4	10	7	5	3	9	175834.3300	-0.0227	0.040	-0.0228	0.50	
59:	8	5	3	7	7	5	2	6	175835.2800	0.0223	0.040	0.0223	0.50	
60:	8	5	4	7	7	5	3	6	175835.2800	0.0223	0.040	0.0223	0.50	
61:	8	4	4	8	7	4	3	7	175849.7930	-0.2535	0.040	-0.0141	0.23	
62:	8	4	4	9	7	4	3	8	175849.7930	0.1957	0.040	-0.0141	0.27	
63:	8	4	5	8	7	4	4	7	175849.7930	-0.2530	0.040	-0.0141	0.23	
64:	8	4	5	9	7	4	4	8	175849.7930	0.1962	0.040	-0.0141	0.27	
65:	8	4	4	7	7	4	3	6	175852.5380	-0.3058	0.040	-0.0387	0.20	
66:	8	4	4	10	7	4	3	9	175852.5380	0.1426	0.040	-0.0387	0.30	
67:	8	4	5	7	7	4	4	6	175852.5380	-0.3053	0.040	-0.0387	0.20	
68:	8	4	5	10	7	4	4	9	175852.5380	0.1430	0.040	-0.0387	0.30	

69:	8	2	7	8	7	2	6	7	175860.2370	0.0260	0.040	-0.0589	0.47
70:	8	2	7	9	7	2	6	8	175860.2370	-0.1335	0.040	-0.0589	0.53
71:	8	2	7	7	7	2	6	6	175861.0340	0.1239	0.040	0.0288	0.40
72:	8	2	7	10	7	2	6	9	175861.0340	-0.0358	0.040	0.0288	0.60
73:	8	3	5	8	7	3	4	7	175867.2140	-0.1957	0.040	-0.0093	0.23
74:	8	3	5	9	7	3	4	8	175867.2140	-0.1015	0.040	-0.0093	0.27
75:	8	3	6	8	7	3	5	7	175867.2140	0.0769	0.040	-0.0093	0.23
76:	8	3	6	9	7	3	5	8	175867.2140	0.1711	0.040	-0.0093	0.27
77:	8	3	5	7	7	3	4	6	175868.8000	-0.1833	0.040	0.0088	0.20
78:	8	3	5	10	7	3	4	9	175868.8000	-0.0897	0.040	0.0088	0.30
79:	8	3	6	7	7	3	5	6	175868.8000	0.0894	0.040	0.0088	0.20
80:	8	3	6	10	7	3	5	9	175868.8000	0.1830	0.040	0.0088	0.30
81:	8	2	6	8	7	2	5	7	175918.8610	0.0640	0.040	-0.0208	0.47
82:	8	2	6	9	7	2	5	8	175918.8610	-0.0953	0.040	-0.0208	0.53
83:	8	2	6	7	7	2	5	6	175919.6270	0.1296	0.040	0.0345	0.40
84:	8	2	6	10	7	2	5	9	175919.6270	-0.0300	0.040	0.0345	0.60
85:	8	1	7	7	7	1	6	6	176982.2270	0.1397	0.040	0.0493	0.20
86:	8	1	7	8	7	1	6	7	176982.2270	0.3142	0.040	0.0493	0.23
87:	8	1	7	9	7	1	6	8	176982.2270	0.0003	0.040	0.0493	0.26
88:	8	1	7	10	7	1	6	9	176982.2270	-0.1741	0.040	0.0493	0.30
89:	9	1	9	11	8	1	8	10	196570.7650	-0.1671	0.040	0.0045	0.29
90:	9	1	9	8	8	1	8	7	196570.7650	0.0832	0.040	0.0045	0.21
91:	9	1	9	9	8	1	8	8	196570.7650	0.2052	0.040	0.0045	0.23
92:	9	1	9	10	8	1	8	9	196570.7650	-0.0451	0.040	0.0045	0.26
93:	9	8	1	11	8	8	0	10	197722.8420	0.0772	0.040	0.0773	0.50
94:	9	8	2	11	8	8	1	10	197722.8420	0.0772	0.040	0.0773	0.50
95:	9	8	1	8	8	8	0	7	197724.5460	0.0577	0.040	0.0577	0.50
96:	9	8	2	8	8	8	1	7	197724.5460	0.0577	0.040	0.0577	0.50
97:	9	7	2	10	8	7	1	9	197748.6090	0.0439	0.040	0.0440	0.50
98:	9	7	3	10	8	7	2	9	197748.6090	0.0439	0.040	0.0440	0.50
99:	9	7	2	9	8	7	1	8	197749.8810	0.0633	0.040	0.0634	0.50
100:	9	7	3	9	8	7	2	8	197749.8810	0.0633	0.040	0.0634	0.50
101:	9	7	2	11	8	7	1	10	197754.5260	-0.0365	0.040	-0.0365	0.50
102:	9	7	3	11	8	7	2	10	197754.5260	-0.0365	0.040	-0.0365	0.50
103:	9	7	2	8	8	7	1	7	197755.8190	0.0036	0.040	0.0037	0.50
104:	9	7	3	8	8	7	2	7	197755.8190	0.0036	0.040	0.0037	0.50
105:	9	0	9	11	8	0	8	10	197770.5200	-0.0977	0.040	0.0272	0.29
106:	9	0	9	8	8	0	8	7	197770.5200	0.1857	0.040	0.0272	0.21
107:	9	0	9	9	8	0	8	8	197770.5200	0.1842	0.040	0.0272	0.23
108:	9	0	9	10	8	0	8	9	197770.5200	-0.0992	0.040	0.0272	0.26
109:	9	6	3	10	8	6	2	9	197777.9940	-0.0309	0.040	-0.0310	0.50
110:	9	6	4	10	8	6	3	9	197777.9940	-0.0309	0.040	-0.0310	0.50
111:	9	6	3	9	8	6	2	8	197778.9160	0.0455	0.040	0.0456	0.50
112:	9	6	4	9	8	6	3	8	197778.9160	0.0455	0.040	0.0456	0.50
113:	9	6	3	11	8	6	2	10	197782.3750	-0.0565	0.040	-0.0566	0.50
114:	9	6	4	11	8	6	3	10	197782.3750	-0.0565	0.040	-0.0566	0.50
115:	9	6	3	8	8	6	2	7	197783.3080	0.0314	0.040	0.0314	0.50
116:	9	6	4	8	8	6	3	7	197783.3080	0.0314	0.040	0.0314	0.50
117:	9	5	4	9	8	5	3	8	197803.7680	-0.2552	0.040	0.0094	0.24
118:	9	5	4	10	8	5	3	9	197803.7680	0.2454	0.040	0.0094	0.26
119:	9	5	5	9	8	5	4	8	197803.7680	-0.2552	0.040	0.0094	0.24
120:	9	5	5	10	8	5	4	9	197803.7680	0.2454	0.040	0.0094	0.26
121:	9	5	4	11	8	5	3	10	197806.7470	0.1641	0.040	-0.0437	0.29
122:	9	5	4	8	8	5	3	7	197806.7470	-0.3359	0.040	-0.0437	0.21
123:	9	5	5	11	8	5	4	10	197806.7470	0.1641	0.040	-0.0437	0.29
124:	9	5	5	8	8	5	4	7	197806.7470	-0.3359	0.040	-0.0437	0.21
125:	9	4	5	9	8	4	4	8	197825.6840	-0.1418	0.040	-0.0258	0.24
126:	9	4	5	10	8	4	4	9	197825.6840	0.0766	0.040	-0.0258	0.26
127:	9	4	6	9	8	4	5	8	197825.6840	-0.1407	0.040	-0.0258	0.24
128:	9	4	6	10	8	4	5	9	197825.6840	0.0777	0.040	-0.0258	0.26
129:	9	4	5	11	8	4	4	10	197827.6330	0.0669	0.040	-0.0231	0.29
130:	9	4	5	8	8	4	4	7	197827.6330	-0.1510	0.040	-0.0231	0.21
131:	9	4	6	11	8	4	5	10	197827.6330	0.0680	0.040	-0.0231	0.29
132:	9	4	6	8	8	4	5	7	197827.6330	-0.1499	0.040	-0.0231	0.21
133:	9	2	8	11	8	2	7	10	197833.1780	-0.3247	0.040	-0.0115	0.29
134:	9	2	8	8	8	2	7	7	197833.1780	-0.1667	0.040	-0.0115	0.21
135:	9	2	8	9	8	2	7	8	197833.1780	0.3225	0.040	-0.0115	0.23
136:	9	2	8	10	8	2	7	9	197833.1780	0.1646	0.040	-0.0115	0.26
137:	9	3	6	9	8	3	5	8	197846.0250	-0.3163	0.040	-0.0670	0.24
138:	9	3	6	10	8	3	5	9	197846.0250	-0.3173	0.040	-0.0670	0.26
139:	9	3	7	9	8	3	6	8	197846.0250	0.1834	0.040	-0.0670	0.24
140:	9	3	7	10	8	3	6	9	197846.0250	0.1825	0.040	-0.0670	0.26
141:	9	3	6	11	8	3	5	10	197847.2850	-0.1593	0.040	0.0912	0.29
142:	9	3	6	8	8	3	5	7	197847.2850	-0.1579	0.040	0.0912	0.21
143:	9	3	7	11	8	3	6	10	197847.2850	0.3405	0.040	0.0912	0.29
144:	9	3	7	8	8	3	6	7	197847.2850	0.3419	0.040	0.0912	0.21
145:	9	2	7	11	8	2	6	10	197916.8930	-0.2603	0.040	0.0536	0.29
146:	9	2	7	8	8	2	6	7	197916.8930	-0.1024	0.040	0.0536	0.21

147:	9	2	7	9	8	2	6	8	197916.8930	0.3883	0.040	0.0536	0.23
148:	9	2	7	10	8	2	6	9	197916.8930	0.2305	0.040	0.0536	0.26
149:	9	1	8	11	8	1	7	10	199093.1460	-0.1719	0.040	0.0011	0.29
150:	9	1	8	8	8	1	7	7	199093.1460	0.0817	0.040	0.0011	0.21
151:	9	1	8	9	8	1	7	8	199093.1460	0.2037	0.040	0.0011	0.23
152:	9	1	8	10	8	1	7	9	199093.1460	-0.0499	0.040	0.0011	0.26
153:	10	1	10	11	9	1	9	10	218397.9580	-0.0665	0.040	-0.0185	0.26
154:	10	1	10	12	9	1	9	11	218397.9580	-0.1551	0.040	-0.0185	0.29
155:	10	1	10	9	9	1	9	8	218397.9580	0.0509	0.040	-0.0185	0.21
156:	10	1	10	10	9	1	9	9	218397.9580	0.1395	0.040	-0.0185	0.24
157:	10	8	2	11	9	8	1	10	219676.6190	-0.0390	0.040	-0.0391	0.50
158:	10	8	3	11	9	8	2	10	219676.6190	-0.0390	0.040	-0.0391	0.50
159:	10	8	2	10	9	8	1	9	219677.7410	0.0036	0.040	0.0036	0.50
160:	10	8	3	10	9	8	2	9	219677.7410	0.0036	0.040	0.0036	0.50
161:	10	8	2	12	9	8	1	11	219682.2990	-0.0561	0.040	-0.0561	0.50
162:	10	8	3	12	9	8	2	11	219682.2990	-0.0561	0.040	-0.0561	0.50
163:	10	8	2	9	9	8	1	8	219683.4690	0.0343	0.040	0.0344	0.50
164:	10	8	3	9	9	8	2	8	219683.4690	0.0343	0.040	0.0344	0.50
165:	10	0	10	11	9	0	9	10	219714.1890	-0.0688	0.040	0.0343	0.26
166:	10	0	10	12	9	0	9	11	219714.1890	-0.0672	0.040	0.0343	0.29
167:	10	0	10	9	9	0	9	8	219714.1890	0.1606	0.040	0.0343	0.21
168:	10	0	10	10	9	0	9	9	219714.1890	0.1589	0.040	0.0343	0.24
169:	10	7	3	12	9	7	2	11	219718.0340	-0.0221	0.040	-0.0221	0.50
170:	10	7	4	12	9	7	3	11	219718.0340	-0.0221	0.040	-0.0221	0.50
171:	10	7	3	9	9	7	2	8	219718.8840	0.0549	0.040	0.0550	0.50
172:	10	7	4	9	9	7	3	8	219718.8840	0.0549	0.040	0.0550	0.50
173:	10	6	4	11	9	6	3	10	219746.3960	0.1823	0.040	-0.0586	0.26
174:	10	6	4	10	9	6	3	9	219746.3960	-0.3254	0.040	-0.0586	0.24
175:	10	6	5	11	9	6	4	10	219746.3960	0.1823	0.040	-0.0586	0.26
176:	10	6	5	10	9	6	4	9	219746.3960	-0.3254	0.040	-0.0586	0.24
177:	10	6	4	12	9	6	3	11	219749.5780	0.1594	0.040	-0.0558	0.29
178:	10	6	4	9	9	6	3	8	219749.5780	-0.3478	0.040	-0.0558	0.21
179:	10	6	5	12	9	6	4	11	219749.5780	0.1594	0.040	-0.0558	0.29
180:	10	6	5	9	9	6	4	8	219749.5780	-0.3478	0.040	-0.0558	0.21
181:	10	5	5	11	9	5	4	10	219774.6230	0.1148	0.040	-0.0196	0.26
182:	10	5	5	10	9	5	4	9	219774.6230	-0.1683	0.040	-0.0196	0.24
183:	10	5	6	11	9	5	5	10	219774.6230	0.1148	0.040	-0.0196	0.26
184:	10	5	6	10	9	5	5	9	219774.6230	-0.1683	0.040	-0.0196	0.24
185:	10	5	5	12	9	5	4	11	219776.8520	0.1180	0.040	-0.0018	0.29
186:	10	5	5	9	9	5	4	8	219776.8520	-0.1645	0.040	-0.0018	0.21
187:	10	5	6	12	9	5	5	11	219776.8520	0.1180	0.040	-0.0018	0.29
188:	10	5	6	9	9	5	5	8	219776.8520	-0.1645	0.040	-0.0018	0.21
189:	10	4	6	11	9	4	5	10	219799.3530	0.0191	0.040	-0.0268	0.26
190:	10	4	6	10	9	4	5	9	219799.3530	-0.0801	0.040	-0.0268	0.24
191:	10	4	7	11	9	4	6	10	219799.3530	0.0215	0.040	-0.0268	0.26
192:	10	4	7	10	9	4	6	9	219799.3530	-0.0777	0.040	-0.0268	0.24
193:	10	4	6	12	9	4	5	11	219800.7800	0.0215	0.040	-0.0192	0.29
194:	10	4	6	9	9	4	5	8	219800.7800	-0.0773	0.040	-0.0192	0.21
195:	10	4	7	12	9	4	6	11	219800.7800	0.0239	0.040	-0.0192	0.29
196:	10	4	7	9	9	4	6	8	219800.7800	-0.0749	0.040	-0.0192	0.21
197:	10	2	9	11	9	2	8	10	219802.1880	0.1223	0.040	0.0091	0.26
198:	10	2	9	12	9	2	8	11	219802.1880	-0.2335	0.040	0.0091	0.29
199:	10	2	9	9	9	2	8	8	219802.1880	-0.0874	0.040	0.0091	0.21
200:	10	2	9	10	9	2	8	9	219802.1880	0.2683	0.040	0.0091	0.24
201:	10	3	8	11	9	3	7	10	219822.6580	-0.0915	0.040	-0.0707	0.53
202:	10	3	8	10	9	3	7	9	219822.6580	-0.0477	0.040	-0.0707	0.47
203:	10	3	7	11	9	3	6	10	219823.5400	-0.0661	0.040	-0.0187	0.26
204:	10	3	7	10	9	3	6	9	219823.5400	-0.0224	0.040	-0.0187	0.24
205:	10	3	8	12	9	3	7	11	219823.5400	-0.0109	0.040	-0.0187	0.29
206:	10	3	8	9	9	3	7	8	219823.5400	0.0330	0.040	-0.0187	0.21
207:	10	3	7	12	9	3	6	11	219824.4370	0.0293	0.040	0.0480	0.58
208:	10	3	7	9	9	3	6	8	219824.4370	0.0733	0.040	0.0480	0.42
209:	10	2	8	11	9	2	7	10	219917.1230	0.1210	0.040	0.0070	0.26
210:	10	2	8	12	9	2	7	11	219917.1230	-0.2364	0.040	0.0070	0.29
211:	10	2	8	9	9	2	7	8	219917.1230	-0.0905	0.040	0.0070	0.21
212:	10	2	8	10	9	2	7	9	219917.1230	0.2669	0.040	0.0070	0.24
213:	10	1	9	11	9	1	8	10	221199.9070	-0.0534	0.040	-0.0043	0.26
214:	10	1	9	12	9	1	8	11	221199.9070	-0.1421	0.040	-0.0043	0.29
215:	10	1	9	9	9	1	8	8	221199.9070	0.0665	0.040	-0.0043	0.21
216:	10	1	9	10	9	1	8	9	221199.9070	0.1551	0.040	-0.0043	0.24
217:	11	1	11	11	10	1	10	10	240220.8230	0.1319	0.040	0.0046	0.24
218:	11	1	11	12	10	1	10	11	240220.8230	-0.0402	0.040	0.0046	0.26
219:	11	1	11	13	10	1	10	12	240220.8230	-0.1065	0.040	0.0046	0.29
220:	11	1	11	10	10	1	10	9	240220.8230	0.0656	0.040	0.0046	0.22
221:	11	9	2	12	10	9	1	11	241589.5820	-0.0593	0.040	-0.0594	0.50
222:	11	9	3	12	10	9	2	11	241589.5820	-0.0593	0.040	-0.0594	0.50
223:	11	9	2	11	10	9	1	10	241590.6340	0.0558	0.040	0.0558	0.50
224:	11	9	3	11	10	9	2	10	241590.6340	0.0558	0.040	0.0558	0.50

225:	11	9	2	13	10	9	1	12	241595.0090	-0.0400	0.040	-0.0401	0.50
226:	11	9	3	13	10	9	2	12	241595.0090	-0.0400	0.040	-0.0401	0.50
227:	11	9	2	10	10	9	1	9	241596.0770	0.0907	0.040	0.0908	0.50
228:	11	9	3	10	10	9	2	9	241596.0770	0.0907	0.040	0.0908	0.50
229:	11	8	3	12	10	8	2	11	241635.2150	-0.0364	0.040	-0.0364	0.50
230:	11	8	4	12	10	8	3	11	241635.2150	-0.0364	0.040	-0.0364	0.50
231:	11	8	3	11	10	8	2	10	241635.9540	0.0014	0.040	0.0014	0.50
232:	11	8	4	11	10	8	3	10	241635.9540	0.0014	0.040	0.0014	0.50
233:	11	8	3	13	10	8	2	12	241639.5310	0.0066	0.040	0.0066	0.50
234:	11	8	4	13	10	8	3	12	241639.5310	0.0066	0.040	0.0066	0.50
235:	11	8	3	10	10	8	2	9	241640.2460	0.0206	0.040	0.0206	0.50
236:	11	8	4	10	10	8	3	9	241640.2460	0.0206	0.040	0.0206	0.50
237:	11	0	11	11	10	0	10	10	241648.1540	0.1214	0.040	0.0201	0.24
238:	11	0	11	12	10	0	10	11	241648.1540	-0.0658	0.040	0.0201	0.26
239:	11	0	11	13	10	0	10	12	241648.1540	-0.0640	0.040	0.0201	0.29
240:	11	0	11	10	10	0	10	9	241648.1540	0.1232	0.040	0.0201	0.22
241:	11	7	4	11	10	7	3	10	241676.0550	-0.2717	0.040	-0.0138	0.24
242:	11	7	4	12	10	7	3	11	241676.0550	0.2213	0.040	-0.0138	0.26
243:	11	7	5	11	10	7	4	10	241676.0550	-0.2717	0.040	-0.0138	0.24
244:	11	7	5	12	10	7	4	11	241676.0550	0.2213	0.040	-0.0138	0.26
245:	11	7	4	13	10	7	3	12	241679.2760	0.1707	0.040	-0.0418	0.28
246:	11	7	4	10	10	7	3	9	241679.2760	-0.3220	0.040	-0.0418	0.22
247:	11	7	5	13	10	7	4	12	241679.2760	0.1707	0.040	-0.0418	0.28
248:	11	7	5	10	10	7	4	9	241679.2760	-0.3220	0.040	-0.0418	0.22
249:	11	6	5	11	10	6	4	10	241711.6820	-0.1919	0.040	-0.0284	0.24
250:	11	6	5	12	10	6	4	11	241711.6820	0.1207	0.040	-0.0284	0.26
251:	11	6	6	11	10	6	5	10	241711.6820	-0.1919	0.040	-0.0284	0.24
252:	11	6	6	12	10	6	5	11	241711.6820	0.1207	0.040	-0.0284	0.26
253:	11	6	5	13	10	6	4	12	241714.0920	0.1270	0.040	-0.0076	0.28
254:	11	6	5	10	10	6	4	9	241714.0920	-0.1852	0.040	-0.0076	0.22
255:	11	6	6	13	10	6	5	12	241714.0920	0.1270	0.040	-0.0076	0.28
256:	11	6	6	10	10	6	5	9	241714.0920	-0.1852	0.040	-0.0076	0.22
257:	11	5	6	11	10	5	5	10	241742.8880	-0.0959	0.040	-0.0123	0.24
258:	11	5	6	12	10	5	5	11	241742.8880	0.0640	0.040	-0.0123	0.26
259:	11	5	7	11	10	5	6	10	241742.8880	-0.0959	0.040	-0.0123	0.24
260:	11	5	7	12	10	5	6	11	241742.8880	0.0640	0.040	-0.0123	0.26
261:	11	5	6	13	10	5	5	12	241744.5590	0.0657	0.040	-0.0031	0.28
262:	11	5	6	10	10	5	5	9	241744.5590	-0.0939	0.040	-0.0031	0.22
263:	11	5	7	13	10	5	6	12	241744.5590	0.0657	0.040	-0.0031	0.28
264:	11	5	7	10	10	5	6	9	241744.5590	-0.0939	0.040	-0.0031	0.22
265:	11	2	10	11	10	2	9	10	241767.2270	0.2044	0.040	-0.0014	0.24
266:	11	2	10	12	10	2	9	11	241767.2270	0.0728	0.040	-0.0014	0.26
267:	11	2	10	13	10	2	9	12	241767.2270	-0.1939	0.040	-0.0014	0.29
268:	11	2	10	10	10	2	9	9	241767.2270	-0.0622	0.040	-0.0014	0.22
269:	11	4	7	11	10	4	6	10	241770.6050	-0.0608	0.040	-0.0401	0.24
270:	11	4	7	12	10	4	6	11	241770.6050	-0.0257	0.040	-0.0401	0.26
271:	11	4	8	11	10	4	7	10	241770.6050	-0.0560	0.040	-0.0401	0.24
272:	11	4	8	12	10	4	7	11	241770.6050	-0.0209	0.040	-0.0401	0.26
273:	11	4	7	13	10	4	6	12	241771.7530	0.0538	0.040	0.0412	0.28
274:	11	4	7	10	10	4	6	9	241771.7530	0.0190	0.040	0.0412	0.22
275:	11	4	8	13	10	4	7	12	241771.7530	0.0585	0.040	0.0412	0.28
276:	11	4	8	10	10	4	7	9	241771.7530	0.0237	0.040	0.0412	0.22
277:	11	3	9	11	10	3	8	10	241797.7650	0.2315	0.040	-0.1042	0.24
278:	11	3	9	12	10	3	8	11	241797.7650	0.1694	0.040	-0.1042	0.26
279:	11	3	9	13	10	3	8	12	241797.7650	-0.4317	0.040	-0.1042	0.29
280:	11	3	9	10	10	3	8	9	241797.7650	-0.3694	0.040	-0.1042	0.22
281:	11	3	8	11	10	3	7	10	241799.3780	0.4529	0.040	0.1172	0.24
282:	11	3	8	12	10	3	7	11	241799.3780	0.3908	0.040	0.1172	0.26
283:	11	3	8	13	10	3	7	12	241799.3780	-0.2103	0.040	0.1172	0.29
284:	11	3	8	10	10	3	7	9	241799.3780	-0.1481	0.040	0.1172	0.22
285:	11	2	9	11	10	2	8	10	241920.3540	0.2222	0.040	0.0156	0.24
286:	11	2	9	12	10	2	8	11	241920.3540	0.0907	0.040	0.0156	0.26
287:	11	2	9	13	10	2	8	12	241920.3540	-0.1778	0.040	0.0156	0.29
288:	11	2	9	10	10	2	8	9	241920.3540	-0.0463	0.040	0.0156	0.22
289:	11	1	10	11	10	1	9	10	243301.9830	0.1165	0.040	-0.0120	0.24
290:	11	1	10	12	10	1	9	11	243301.9830	-0.0578	0.040	-0.0120	0.26
291:	11	1	10	13	10	1	9	12	243301.9830	-0.1241	0.040	-0.0120	0.29
292:	11	1	10	10	10	1	9	9	243301.9830	0.0502	0.040	-0.0120	0.22
293:	12	1	12	11	11	1	11	10	262038.8770	0.0600	0.040	0.0063	0.22
294:	12	1	12	12	11	1	11	11	262038.8770	0.1109	0.040	0.0063	0.24
295:	12	1	12	13	11	1	11	12	262038.8770	-0.0349	0.040	0.0063	0.26
296:	12	1	12	14	11	1	11	13	262038.8770	-0.0858	0.040	0.0063	0.28
297:	12	11	1	13	11	11	0	12	263425.0530	-0.0536	0.040	-0.0536	0.50
298:	12	11	2	13	11	11	1	12	263425.0530	-0.0536	0.040	-0.0536	0.50
299:	12	11	1	12	11	11	0	11	263426.1650	0.0344	0.040	0.0344	0.50
300:	12	11	2	12	11	11	1	11	263426.1650	0.0344	0.040	0.0344	0.50
301:	12	11	1	14	11	11	0	13	263431.2350	-0.0853	0.040	-0.0853	0.50
302:	12	11	2	14	11	11	1	13	263431.2350	-0.0853	0.040	-0.0853	0.50

303:	12	11	1	11	11	11	0	10	263432.3500	0.0047	0.040	0.0048	0.50
304:	12	11	2	11	11	11	1	10	263432.3500	0.0047	0.040	0.0048	0.50
305:	12	10	2	13	11	10	1	12	263485.7780	-0.0549	0.040	-0.0549	0.50
306:	12	10	3	13	11	10	2	12	263485.7780	-0.0549	0.040	-0.0549	0.50
307:	12	10	2	12	11	10	1	11	263486.7250	0.0727	0.040	0.0727	0.50
308:	12	10	3	12	11	10	2	11	263486.7250	0.0727	0.040	0.0727	0.50
309:	12	10	2	14	11	10	1	13	263490.9180	-0.0504	0.040	-0.0505	0.50
310:	12	10	3	14	11	10	2	13	263490.9180	-0.0504	0.040	-0.0505	0.50
311:	12	10	2	11	11	10	1	10	263491.8700	0.0819	0.040	0.0819	0.50
312:	12	10	3	11	11	10	2	10	263491.8700	0.0819	0.040	0.0819	0.50
313:	12	0	12	11	11	0	11	10	263571.5050	0.0857	0.040	-0.0001	0.22
314:	12	0	12	12	11	0	11	11	263571.5050	0.0837	0.040	-0.0001	0.24
315:	12	0	12	13	11	0	11	12	263571.5050	-0.0729	0.040	-0.0001	0.26
316:	12	0	12	14	11	0	11	13	263571.5050	-0.0708	0.040	-0.0001	0.28
317:	12	8	4	12	11	8	3	11	263590.8400	-0.2567	0.040	-0.0127	0.24
318:	12	8	4	13	11	8	3	12	263590.8400	0.2115	0.040	-0.0127	0.26
319:	12	8	5	12	11	8	4	11	263590.8400	-0.2567	0.040	-0.0127	0.24
320:	12	8	5	13	11	8	4	12	263590.8400	0.2115	0.040	-0.0127	0.26
321:	12	8	4	11	11	8	3	10	263594.0850	-0.2984	0.040	-0.0350	0.22
322:	12	8	4	14	11	8	3	13	263594.0850	0.1695	0.040	-0.0350	0.28
323:	12	8	5	11	11	8	4	10	263594.0850	-0.2984	0.040	-0.0350	0.22
324:	12	8	5	14	11	8	4	13	263594.0850	0.1695	0.040	-0.0350	0.28
325:	12	7	5	12	11	7	4	11	263635.0010	-0.1813	0.040	-0.0136	0.24
326:	12	7	5	13	11	7	4	12	263635.0010	0.1405	0.040	-0.0136	0.26
327:	12	7	6	12	11	7	5	11	263635.0010	-0.1813	0.040	-0.0136	0.24
328:	12	7	6	13	11	7	5	12	263635.0010	0.1405	0.040	-0.0136	0.26
329:	12	7	5	11	11	7	4	10	263637.5040	-0.1946	0.040	-0.0136	0.22
330:	12	7	5	14	11	7	4	13	263637.5040	0.1269	0.040	-0.0136	0.28
331:	12	7	6	11	11	7	5	10	263637.5040	-0.1946	0.040	-0.0136	0.22
332:	12	7	6	14	11	7	5	13	263637.5040	0.1269	0.040	-0.0136	0.28
333:	12	6	6	12	11	6	5	11	263673.9950	-0.1120	0.040	-0.0104	0.24
334:	12	6	6	13	11	6	5	12	263673.9950	0.0830	0.040	-0.0104	0.26
335:	12	6	7	12	11	6	6	11	263673.9950	-0.1120	0.040	-0.0104	0.24
336:	12	6	7	13	11	6	6	12	263673.9950	0.0830	0.040	-0.0104	0.26
337:	12	6	6	11	11	6	5	10	263675.8410	-0.1146	0.040	-0.0051	0.22
338:	12	6	6	14	11	6	5	13	263675.8410	0.0799	0.040	-0.0051	0.28
339:	12	6	7	11	11	6	6	10	263675.8410	-0.1146	0.040	-0.0051	0.22
340:	12	6	7	14	11	6	6	13	263675.8410	0.0799	0.040	-0.0051	0.28
341:	12	5	7	12	11	5	6	11	263708.2980	-0.0809	0.040	-0.0352	0.24
342:	12	5	7	13	11	5	6	12	263708.2980	0.0067	0.040	-0.0352	0.26
343:	12	5	8	12	11	5	7	11	263708.2980	-0.0809	0.040	-0.0352	0.24
344:	12	5	8	13	11	5	7	12	263708.2980	0.0067	0.040	-0.0352	0.26
345:	12	5	7	11	11	5	6	10	263709.6190	-0.0437	0.040	0.0054	0.22
346:	12	5	7	14	11	5	6	13	263709.6190	0.0435	0.040	0.0054	0.28
347:	12	5	8	11	11	5	7	10	263709.6190	-0.0437	0.040	0.0054	0.22
348:	12	5	8	14	11	5	7	13	263709.6190	0.0435	0.040	0.0054	0.28
349:	12	2	11	11	11	2	10	10	263727.9500	-0.0337	0.040	0.0048	0.22
350:	12	2	11	12	11	2	10	11	263727.9500	0.1713	0.040	0.0048	0.24
351:	12	2	11	13	11	2	10	12	263727.9500	0.0538	0.040	0.0048	0.26
352:	12	2	11	14	11	2	10	13	263727.9500	-0.1512	0.040	0.0048	0.28
353:	12	4	8	12	11	4	7	11	263739.2430	-0.0678	0.040	-0.0635	0.24
354:	12	4	8	13	11	4	7	12	263739.2430	-0.0681	0.040	-0.0635	0.26
355:	12	4	9	12	11	4	8	11	263739.2430	-0.0589	0.040	-0.0635	0.24
356:	12	4	9	13	11	4	8	12	263739.2430	-0.0592	0.040	-0.0635	0.26
357:	12	4	8	11	11	4	7	10	263740.1820	0.0494	0.040	0.0536	0.22
358:	12	4	8	14	11	4	7	13	263740.1820	0.0489	0.040	0.0536	0.28
359:	12	4	9	11	11	4	8	10	263740.1820	0.0583	0.040	0.0536	0.22
360:	12	4	9	14	11	4	8	13	263740.1820	0.0578	0.040	0.0536	0.28
361:	12	3	10	11	11	3	9	10	263770.3930	-0.1852	0.040	0.0079	0.22
362:	12	3	10	12	11	3	9	11	263770.3930	0.2771	0.040	0.0079	0.24
363:	12	3	10	13	11	3	9	12	263770.3930	0.2084	0.040	0.0079	0.26
364:	12	3	10	14	11	3	9	13	263770.3930	-0.2540	0.040	0.0079	0.28
365:	12	3	9	11	11	3	8	10	263772.5540	-0.1881	0.040	0.0050	0.22
366:	12	3	9	12	11	3	8	11	263772.5540	0.2743	0.040	0.0050	0.24
367:	12	3	9	13	11	3	8	12	263772.5540	0.2056	0.040	0.0050	0.26
368:	12	3	9	14	11	3	8	13	263772.5540	-0.2568	0.040	0.0050	0.28
369:	12	2	10	11	11	2	9	10	263926.7660	-0.0329	0.040	0.0066	0.22
370:	12	2	10	12	11	2	9	11	263926.7660	0.1741	0.040	0.0066	0.24
371:	12	2	10	13	11	2	9	12	263926.7660	0.0567	0.040	0.0066	0.26
372:	12	2	10	14	11	2	9	13	263926.7660	-0.1504	0.040	0.0066	0.28
373:	12	1	11	11	11	1	10	10	265398.9040	0.0504	0.040	-0.0043	0.22
374:	12	1	11	12	11	1	10	11	265398.9040	0.1013	0.040	-0.0043	0.24
375:	12	1	11	13	11	1	10	12	265398.9040	-0.0464	0.040	-0.0043	0.26
376:	12	1	11	14	11	1	10	13	265398.9040	-0.0972	0.040	-0.0043	0.28
377:	13	1	13	12	12	1	12	11	283851.7400	0.0637	0.040	0.0162	0.22
378:	13	1	13	13	12	1	12	12	283851.7400	0.1035	0.040	0.0162	0.24
379:	13	1	13	14	12	1	12	13	283851.7400	-0.0215	0.040	0.0162	0.26
380:	13	1	13	15	12	1	12	14	283851.7400	-0.0614	0.040	0.0162	0.28

381:	13	12	1	14	12	12	0	13	285291.8500	-0.0304	0.040	-0.0304	0.50
382:	13	12	2	14	12	12	1	13	285291.8500	-0.0304	0.040	-0.0304	0.50
383:	13	12	1	13	12	12	0	12	285292.8320	0.0676	0.040	0.0677	0.50
384:	13	12	2	13	12	12	1	12	285292.8320	0.0676	0.040	0.0677	0.50
385:	13	12	1	15	12	12	0	14	285297.6860	-0.0046	0.040	-0.0046	0.50
386:	13	12	2	15	12	12	1	14	285297.6860	-0.0046	0.040	-0.0046	0.50
387:	13	12	1	12	12	12	0	11	285298.6530	0.0775	0.040	0.0776	0.50
388:	13	12	2	12	12	12	1	11	285298.6530	0.0775	0.040	0.0776	0.50
389:	13	11	2	14	12	11	1	13	285363.5570	-0.0044	0.040	-0.0045	0.50
390:	13	11	3	14	12	11	2	13	285363.5570	-0.0044	0.040	-0.0045	0.50
391:	13	11	2	13	12	11	1	12	285364.3450	0.0618	0.040	0.0618	0.50
392:	13	11	3	13	12	11	2	12	285364.3450	0.0618	0.040	0.0618	0.50
393:	13	11	2	15	12	11	1	14	285368.4260	-0.0178	0.040	-0.0179	0.50
394:	13	11	3	15	12	11	2	14	285368.4260	-0.0178	0.040	-0.0179	0.50
395:	13	11	2	12	12	11	1	11	285369.2040	0.0380	0.040	0.0381	0.50
396:	13	11	3	12	12	11	2	11	285369.2040	0.0380	0.040	0.0381	0.50
397:	13	10	3	13	12	10	2	12	285429.4620	-0.3288	0.040	-0.0310	0.24
398:	13	10	3	14	12	10	2	13	285429.4620	0.2446	0.040	-0.0310	0.26
399:	13	10	4	13	12	10	3	12	285429.4620	-0.3288	0.040	-0.0310	0.24
400:	13	10	4	14	12	10	3	13	285429.4620	0.2446	0.040	-0.0310	0.26
401:	13	10	3	12	12	10	2	11	285433.4450	-0.3810	0.040	-0.0610	0.22
402:	13	10	3	15	12	10	2	14	285433.4450	0.1925	0.040	-0.0610	0.28
403:	13	10	4	12	12	10	3	11	285433.4450	-0.3810	0.040	-0.0610	0.22
404:	13	10	4	15	12	10	3	14	285433.4450	0.1925	0.040	-0.0610	0.28
405:	13	0	13	12	12	0	12	11	285483.3570	0.0665	0.040	-0.0063	0.22
406:	13	0	13	13	12	0	12	12	285483.3570	0.0643	0.040	-0.0063	0.24
407:	13	0	13	14	12	0	12	13	285483.3570	-0.0687	0.040	-0.0063	0.26
408:	13	0	13	15	12	0	12	14	285483.3570	-0.0665	0.040	-0.0063	0.28
409:	13	9	4	13	12	9	3	12	285489.0920	-0.2289	0.040	-0.0007	0.24
410:	13	9	4	14	12	9	3	13	285489.0920	0.2104	0.040	-0.0007	0.26
411:	13	9	5	13	12	9	4	12	285489.0920	-0.2289	0.040	-0.0007	0.24
412:	13	9	5	14	12	9	4	13	285489.0920	0.2104	0.040	-0.0007	0.26
413:	13	9	4	12	12	9	3	11	285492.3250	-0.2643	0.040	-0.0193	0.22
414:	13	9	4	15	12	9	3	14	285492.3250	0.1748	0.040	-0.0193	0.28
415:	13	9	5	12	12	9	4	11	285492.3250	-0.2643	0.040	-0.0193	0.22
416:	13	9	5	15	12	9	4	14	285492.3250	0.1748	0.040	-0.0193	0.28
417:	13	8	5	13	12	8	4	12	285542.7710	-0.1732	0.040	-0.0074	0.24
418:	13	8	5	14	12	8	4	13	285542.7710	0.1460	0.040	-0.0074	0.26
419:	13	8	6	13	12	8	5	12	285542.7710	-0.1732	0.040	-0.0074	0.24
420:	13	8	6	14	12	8	5	13	285542.7710	0.1460	0.040	-0.0074	0.26
421:	13	8	5	12	12	8	4	11	285545.3330	-0.1936	0.040	-0.0156	0.22
422:	13	8	5	15	12	8	4	14	285545.3330	0.1254	0.040	-0.0156	0.28
423:	13	8	6	12	12	8	5	11	285545.3330	-0.1936	0.040	-0.0156	0.22
424:	13	8	6	15	12	8	5	14	285545.3330	0.1254	0.040	-0.0156	0.28
425:	13	7	6	13	12	7	5	12	285590.6800	-0.1224	0.040	-0.0116	0.24
426:	13	7	6	14	12	7	5	13	285590.6800	0.0909	0.040	-0.0116	0.26
427:	13	7	7	13	12	7	6	12	285590.6800	-0.1224	0.040	-0.0116	0.24
428:	13	7	7	14	12	7	6	13	285590.6800	0.0909	0.040	-0.0116	0.26
429:	13	7	6	12	12	7	5	11	285592.6530	-0.1265	0.040	-0.0076	0.22
430:	13	7	6	15	12	7	5	14	285592.6530	0.0865	0.040	-0.0076	0.28
431:	13	7	7	12	12	7	6	11	285592.6530	-0.1265	0.040	-0.0076	0.22
432:	13	7	7	15	12	7	6	14	285592.6530	0.0865	0.040	-0.0076	0.28
433:	13	6	7	13	12	6	6	12	285633.1070	-0.0794	0.040	-0.0163	0.24
434:	13	6	7	14	12	6	6	13	285633.1070	0.0421	0.040	-0.0163	0.26
435:	13	6	8	13	12	6	7	12	285633.1070	-0.0794	0.040	-0.0163	0.24
436:	13	6	8	14	12	6	7	13	285633.1070	0.0421	0.040	-0.0163	0.26
437:	13	6	7	12	12	6	6	11	285634.5810	-0.0580	0.040	0.0097	0.22
438:	13	6	7	15	12	6	6	14	285634.5810	0.0632	0.040	0.0097	0.28
439:	13	6	8	12	12	6	7	11	285634.5810	-0.0580	0.040	0.0097	0.22
440:	13	6	8	15	12	6	7	14	285634.5810	0.0632	0.040	0.0097	0.28
441:	13	5	8	13	12	5	7	12	285670.6760	-0.0682	0.040	-0.0454	0.24
442:	13	5	8	14	12	5	7	13	285670.6760	-0.0244	0.040	-0.0454	0.26
443:	13	5	9	13	12	5	8	12	285670.6760	-0.0681	0.040	-0.0454	0.24
444:	13	5	9	14	12	5	8	13	285670.6760	-0.0243	0.040	-0.0454	0.26
445:	13	5	8	12	12	5	7	11	285671.7620	0.0090	0.040	0.0334	0.22
446:	13	5	8	15	12	5	7	14	285671.7620	0.0526	0.040	0.0334	0.28
447:	13	5	9	12	12	5	8	11	285671.7620	0.0090	0.040	0.0334	0.22
448:	13	5	9	15	12	5	8	14	285671.7620	0.0526	0.040	0.0334	0.28
449:	13	2	12	12	12	2	11	11	285683.9390	-0.0219	0.040	0.0019	0.22
450:	13	2	12	13	12	2	11	12	285683.9390	0.1390	0.040	0.0019	0.24
451:	13	2	12	14	12	2	11	13	285683.9390	0.0343	0.040	0.0019	0.26
452:	13	2	12	15	12	2	11	14	285683.9390	-0.1266	0.040	0.0019	0.28
453:	13	4	9	13	12	4	8	12	285705.2290	0.0804	0.040	0.0781	0.24
454:	13	4	9	14	12	4	8	13	285705.2290	0.0606	0.040	0.0781	0.26
455:	13	4	10	13	12	4	9	12	285705.2290	0.0962	0.040	0.0781	0.24
456:	13	4	10	14	12	4	9	13	285705.2290	0.0764	0.040	0.0781	0.26
457:	13	4	9	12	12	4	8	11	285705.7370	-0.0572	0.040	-0.0605	0.22
458:	13	4	9	15	12	4	8	14	285705.7370	-0.0772	0.040	-0.0605	0.28

459:	13	4	10	12	12	4	9	11	285705.7370	-0.0414	0.040	-0.0605	0.22
460:	13	4	10	15	12	4	9	14	285705.7370	-0.0613	0.040	-0.0605	0.28
461:	13	3	11	12	12	3	10	11	285740.4540	-0.1432	0.040	0.0005	0.22
462:	13	3	11	13	12	3	10	12	285740.4540	0.2201	0.040	0.0005	0.24
463:	13	3	11	14	12	3	10	13	285740.4540	0.1508	0.040	0.0005	0.26
464:	13	3	11	15	12	3	10	14	285740.4540	-0.2126	0.040	0.0005	0.28
465:	13	3	10	12	12	3	9	11	285743.6930	-0.1485	0.040	-0.0047	0.22
466:	13	3	10	13	12	3	9	12	285743.6930	0.2148	0.040	-0.0047	0.24
467:	13	3	10	14	12	3	9	13	285743.6930	0.1455	0.040	-0.0047	0.26
468:	13	3	10	15	12	3	9	14	285743.6930	-0.2179	0.040	-0.0047	0.28
469:	13	2	11	12	12	2	10	11	285936.6220	-0.0145	0.040	0.0104	0.22
470:	13	2	11	13	12	2	10	12	285936.6220	0.1486	0.040	0.0104	0.24
471:	13	2	11	14	12	2	10	13	285936.6220	0.0440	0.040	0.0104	0.26
472:	13	2	11	15	12	2	10	14	285936.6220	-0.1191	0.040	0.0104	0.28
473:	13	1	12	12	12	1	11	11	287490.1680	0.0587	0.040	0.0104	0.22
474:	13	1	12	13	12	1	11	12	287490.1680	0.0985	0.040	0.0104	0.24
475:	13	1	12	14	12	1	11	13	287490.1680	-0.0281	0.040	0.0104	0.26
476:	13	1	12	15	12	1	11	14	287490.1680	-0.0678	0.040	0.0104	0.28
477:	14	1	14	13	13	1	13	12	305658.9760	0.0437	0.040	0.0014	0.22
478:	14	1	14	14	13	1	13	13	305658.9760	0.0754	0.040	0.0014	0.24
479:	14	1	14	15	13	1	13	14	305658.9760	-0.0329	0.040	0.0014	0.26
480:	14	1	14	16	13	1	13	15	305658.9760	-0.0647	0.040	0.0014	0.28
481:	14	10	4	14	13	10	3	13	307369.0960	-0.2121	0.040	0.0001	0.24
482:	14	10	4	15	13	10	3	14	307369.0960	0.1976	0.040	0.0001	0.26
483:	14	10	5	14	13	10	4	13	307369.0960	-0.2121	0.040	0.0001	0.24
484:	14	10	5	15	13	10	4	14	307369.0960	0.1976	0.040	0.0001	0.26
485:	14	10	4	13	13	10	3	12	307372.2840	-0.2521	0.040	-0.0254	0.22
486:	14	10	4	16	13	10	3	15	307372.2840	0.1574	0.040	-0.0254	0.28
487:	14	10	5	13	13	10	4	12	307372.2840	-0.2521	0.040	-0.0254	0.22
488:	14	10	5	16	13	10	4	15	307372.2840	0.1574	0.040	-0.0254	0.28
489:	14	0	14	13	13	0	13	12	307382.8280	0.0615	0.040	-0.0009	0.22
490:	14	0	14	14	13	0	13	13	307382.8280	0.0592	0.040	-0.0009	0.24
491:	14	0	14	15	13	0	13	14	307382.8280	-0.0552	0.040	-0.0009	0.26
492:	14	0	14	16	13	0	13	15	307382.8280	-0.0528	0.040	-0.0009	0.28
493:	14	9	5	14	13	9	4	13	307433.2750	-0.1613	0.040	-0.0006	0.24
494:	14	9	5	15	13	9	4	14	307433.2750	0.1488	0.040	-0.0006	0.26
495:	14	9	6	14	13	9	5	13	307433.2750	-0.1613	0.040	-0.0006	0.24
496:	14	9	6	15	13	9	5	14	307433.2750	0.1488	0.040	-0.0006	0.26
497:	14	9	5	13	13	9	4	12	307435.8790	-0.1720	0.040	-0.0003	0.22
498:	14	9	5	16	13	9	4	15	307435.8790	0.1379	0.040	-0.0003	0.28
499:	14	9	6	13	13	9	5	12	307435.8790	-0.1720	0.040	-0.0003	0.22
500:	14	9	6	16	13	9	5	15	307435.8790	0.1379	0.040	-0.0003	0.28
501:	14	7	7	14	13	7	6	13	307542.8680	-0.0765	0.040	-0.0027	0.24
502:	14	7	7	15	13	7	6	14	307542.8680	0.0660	0.040	-0.0027	0.26
503:	14	7	8	14	13	7	7	13	307542.8680	-0.0765	0.040	-0.0027	0.24
504:	14	7	8	15	13	7	7	14	307542.8680	0.0660	0.040	-0.0027	0.26
505:	14	7	7	13	13	7	6	12	307544.4630	-0.0632	0.040	0.0156	0.22
506:	14	7	7	16	13	7	6	15	307544.4630	0.0790	0.040	0.0156	0.28
507:	14	7	8	13	13	7	7	12	307544.4630	-0.0632	0.040	0.0156	0.22
508:	14	7	8	16	13	7	7	15	307544.4630	0.0790	0.040	0.0156	0.28
509:	14	6	8	14	13	6	7	13	307588.8090	-0.0606	0.040	-0.0221	0.24
510:	14	6	8	15	13	6	7	14	307588.8090	0.0137	0.040	-0.0221	0.26
511:	14	6	9	14	13	6	8	13	307588.8090	-0.0606	0.040	-0.0221	0.24
512:	14	6	9	15	13	6	8	14	307588.8090	0.0137	0.040	-0.0221	0.26
513:	14	6	8	13	13	6	7	12	307590.0260	-0.0057	0.040	0.0354	0.22
514:	14	6	8	16	13	6	7	15	307590.0260	0.0684	0.040	0.0354	0.28
515:	14	6	9	13	13	6	8	12	307590.0260	-0.0057	0.040	0.0354	0.22
516:	14	6	9	16	13	6	8	15	307590.0260	0.0684	0.040	0.0354	0.28
517:	14	5	9	14	13	5	8	13	307629.8060	-0.0357	0.040	-0.0270	0.24
518:	14	5	9	15	13	5	8	14	307629.8060	-0.0189	0.040	-0.0270	0.26
519:	14	5	10	14	13	5	9	13	307629.8060	-0.0356	0.040	-0.0270	0.24
520:	14	5	10	15	13	5	9	14	307629.8060	-0.0188	0.040	-0.0270	0.26
521:	14	5	9	13	13	5	8	12	307630.6850	0.0362	0.040	0.0455	0.22
522:	14	5	9	16	13	5	8	15	307630.6850	0.0528	0.040	0.0455	0.28
523:	14	5	10	13	13	5	9	12	307630.6850	0.0363	0.040	0.0455	0.22
524:	14	5	10	16	13	5	9	15	307630.6850	0.0529	0.040	0.0455	0.28
525:	14	2	13	13	13	2	12	12	307634.8170	-0.0089	0.040	0.0052	0.22
526:	14	2	13	14	13	2	12	13	307634.8170	0.1197	0.040	0.0052	0.24
527:	14	2	13	15	13	2	12	14	307634.8170	0.0263	0.040	0.0052	0.26
528:	14	2	13	16	13	2	12	15	307634.8170	-0.1024	0.040	0.0052	0.28
529:	14	4	10	13	13	4	9	12	307668.2420	-0.2296	0.040	0.0251	0.11
530:	14	4	10	14	13	4	9	13	307668.2420	0.2869	0.040	0.0251	0.12
531:	14	4	10	15	13	4	9	14	307668.2420	0.2565	0.040	0.0251	0.13
532:	14	4	10	16	13	4	9	15	307668.2420	-0.2602	0.040	0.0251	0.14
533:	14	4	11	13	13	4	10	12	307668.2420	-0.2027	0.040	0.0251	0.11
534:	14	4	11	14	13	4	10	13	307668.2420	0.3138	0.040	0.0251	0.12
535:	14	4	11	15	13	4	10	14	307668.2420	0.2834	0.040	0.0251	0.13
536:	14	4	11	16	13	4	10	15	307668.2420	-0.2333	0.040	0.0251	0.14

```
 537: 14  3 12 13   13  3 11 12           307707.8570  -0.0932  0.040   0.0158 0.22
 538: 14  3 12 14   13  3 11 13           307707.8570   0.1975  0.040   0.0158 0.24
 539: 14  3 12 15   13  3 11 14           307707.8570   0.1304  0.040   0.0158 0.26
 540: 14  3 12 16   13  3 11 15           307707.8570  -0.1604  0.040   0.0158 0.28
 541: 14  3 11 13   13  3 10 12           307712.5670  -0.0996  0.040   0.0094 0.22
 542: 14  3 11 14   13  3 10 13           307712.5670   0.1912  0.040   0.0094 0.24
 543: 14  3 11 15   13  3 10 14           307712.5670   0.1240  0.040   0.0094 0.26
 544: 14  3 11 16   13  3 10 15           307712.5670  -0.1668  0.040   0.0094 0.28
 545: 14  2 12 13   13  2 11 12           307950.1040  -0.0126  0.040   0.0027 0.22
 546: 14  2 12 14   13  2 11 13           307950.1040   0.1184  0.040   0.0027 0.24
 547: 14  2 12 15   13  2 11 14           307950.1040   0.0251  0.040   0.0027 0.26
 548: 14  2 12 16   13  2 11 15           307950.1040  -0.1059  0.040   0.0027 0.28
 549: 14  1 13 13   13  1 12 12           309575.2490   0.0488  0.040   0.0058 0.22
 550: 14  1 13 14   13  1 12 13           309575.2490   0.0805  0.040   0.0058 0.24
 551: 14  1 13 15   13  1 12 14           309575.2490  -0.0292  0.040   0.0058 0.26
 552: 14  1 13 16   13  1 12 15           309575.2490  -0.0609  0.040   0.0058 0.28
--------------------------------------------------------------------------------

PARAMETERS IN FIT (values truncated and Nlines statistics):

        10000            A  /MHz        95359.1(60)                            1
        20000            B  /MHz        11134.1477(92)                         2
        30000            C  /MHz        10853.4201(92)                         3
          200      Delta_J  /kHz           11.6429(38)                         4
         1100     Delta_JK  /kHz          118.942(22)                          5
         2000      Delta_K  /kHz         [932.871]                             6
        40100      delta_J  /kHz            0.2722(32)                         7
        41000      delta_K  /kHz           57.7(45)                            8
          300        Phi_J  /Hz           [-0.002532]                          9
         1200       Phi_JK  /Hz           [ 0.287]                            10
         2100       Phi_KJ  /Hz           [ 2.544]                            11
         3000        Phi_K  /Hz           [32.]                               12
        40200        phi_J  /Hz           [ 0.000122]                         13
        41100       phi_JK  /Hz           [ 0.235]                            14
        42000        phi_K  /Hz           [22.]                               15
    110010000         X_aa  /MHz          -58.751(57)                         16
   -110030000         X_cc  /MHz           58.751(57)           = -1.00000 * 16
    110020000         X_bb  /MHz           29.71(20)                          17
   -110030000         X_cc  /MHz          -29.71(20)            = -1.00000 * 17

 MICROWAVE AVG =       -0.000094 MHz, IR AVG =        0.00000
 MICROWAVE RMS =        0.035300 MHz, IR RMS =        0.00000
 END OF ITERATION  1 OLD, NEW RMS ERROR=        0.90468         0.90468

  distinct frequency lines in fit:   180
       distinct parameters of fit:     9

                                   upper state  lower state         overall
      limits of quantum number  1:      4   14       3   13           3   14
      limits of quantum number  2:      0   12       0   12           0   12
      limits of quantum number  3:      1   14       0   13           0   14
      limits of quantum number  4:      3   16       2   15           2   16

                  frequency range:       87382       309575


 PARAMETERS IN FIT WITH STANDARD ERRORS ON THOSE THAT ARE FITTED:
 (values rounded and degrees of freedom, Ndegf=Nlines-Nconst, statistics)

        10000            A  /MHz        95359.1(56)                            1
        20000            B  /MHz        11134.1477(85)                         2
        30000            C  /MHz        10853.4201(85)                         3
          200      Delta_J  /kHz           11.6429(35)                         4
         1100     Delta_JK  /kHz          118.943(20)                          5
         2000      Delta_K  /kHz         [932.871]                             6
        40100      delta_J  /kHz            0.2722(30)                         7
        41000      delta_K  /kHz           57.7(42)                            8
          300        Phi_J  /Hz           [-0.002532]                          9
         1200       Phi_JK  /Hz           [ 0.287]                            10
         2100       Phi_KJ  /Hz           [ 2.544]                            11
         3000        Phi_K  /Hz           [32.]                               12
        40200        phi_J  /Hz           [ 0.000122]                         13
        41100       phi_JK  /Hz           [ 0.235]                            14
        42000        phi_K  /Hz           [22.]                               15
    110010000         X_aa  /MHz          -58.751(53)                         16
   -110030000         X_cc  /MHz           58.751(53)           = -1.00000 * 16
    110020000         X_bb  /MHz           29.71(19)                          17
   -110030000         X_cc  /MHz          -29.71(19)            = -1.00000 * 17
```

```
 CORRELATION COEFFICIENTS, C.ij:

           A         B         C         -Delta_J -Delta_J -delta_J -delta_K X_aa

A           1.0000
B          -0.5172   1.0000
C           0.5184  -0.9963   1.0000
-Delta_J    0.4810  -0.9248   0.8967   1.0000
-Delta_JK  -0.5164   0.9690  -0.9703  -0.9313   1.0000
-delta_J    0.0409  -0.1840   0.1826   0.0348  -0.0305   1.0000
-delta_K    0.5377  -0.9831   0.9834   0.9250  -0.9863   0.0318   1.0000
X_aa       -0.0050   0.0012   0.0058  -0.0315   0.0064   0.0267  -0.0036   1.0000
X_bb       -0.0004  -0.0158   0.0239  -0.0264  -0.0065   0.0770   0.0037  -0.2773

           X_bb

X_bb        1.0000

 Mean value of |C.ij|, i.ne.j =   0.3654
 Mean value of  C.ij,  i.ne.j =  -0.0461


 Worst correlations, with absolute value greater than 0.9950:

       30000  C         <->      20000  B           -0.996328


 Worst fitted lines (obs-calc/error):

   281:    2.9        277:   -2.6        141:    2.3        227:    2.3
   301:   -2.1        311:    2.0        453:    2.0        387:    1.9
    93:    1.9        307:    1.8        201:   -1.8        383:    1.7
   137:   -1.7        353:   -1.6         99:    1.6        391:    1.5
   401:   -1.5        457:   -1.5        221:   -1.5         69:   -1.5
   173:   -1.5         95:    1.4        113:   -1.4        161:   -1.4
   177:   -1.4        223:    1.4        171:    1.4        305:   -1.4
   297:   -1.3        357:    1.3        145:    1.3         39:   -1.3
   309:   -1.3         35:    1.3         18:   -1.2         85:    1.2
   207:    1.2        111:    1.1        521:    1.1        441:   -1.1
    97:    1.1        121:   -1.1        245:   -1.0         17:    1.0
   273:    1.0         20:    1.0         19:    1.0        269:   -1.0
   225:   -1.0          6:    1.0

   281: 11  3  8 11   10  3  7 10            241799.3780   0.4529  0.040   0.1172 0.24
   277: 11  3  9 11   10  3  8 10            241797.7650   0.2315  0.040  -0.1042 0.24
   141:  9  3  6 11    8  3  5 10            197847.2850  -0.1593  0.040   0.0912 0.29
   227: 11  9  2 10   10  9  1  9            241596.0770   0.0907  0.040   0.0908 0.50
   301: 12 11  1 14   11 11  0 13            263431.2350  -0.0853  0.040  -0.0853 0.50
   311: 12 10  2 11   11 10  1 10            263491.8700   0.0819  0.040   0.0819 0.50
   453: 13  4  9 13   12  4  8 12            285705.2290   0.0804  0.040   0.0781 0.24
   387: 13 12  1 12   12 12  0 11            285298.6530   0.0775  0.040   0.0776 0.50
    93:  9  8  1 11    8  8  0 10            197722.8420   0.0772  0.040   0.0773 0.50
   307: 12 10  2 12   11 10  1 11            263486.7250   0.0727  0.040   0.0727 0.50
                                          ______________________________________
__________________________________________/ SPFIT output reformatted with PIFORM
```

Table S.5. Part of the CFOUR output file of the CCSD(T)/V5Z-aV(5+*d*)Z harmonic frequency calculation for $^{12}CHD_2{}^{35}Cl$

```
   **************************************************************************
         <<<     CCCCCC     CCCCCC   |||     CCCCCC     CCCCCC   >>>
       <<<      CCC        CCC       |||    CCC        CCC         >>>
      <<<      CCC        CCC        |||   CCC        CCC            >>>
    <<<        CCC        CCC        |||   CCC        CCC              >>>
      <<<      CCC        CCC        |||   CCC        CCC            >>>
       <<<      CCC        CCC       |||    CCC        CCC         >>>
         <<<     CCCCCC     CCCCCC   |||     CCCCCC     CCCCCC   >>>
   **************************************************************************

     ****************************************************************
     * CFOUR Coupled-Cluster techniques for Computational Chemistry *
     ****************************************************************


   Department of Chemistry                Institut fuer Physikalische Chemie
   University of Texas at Austin          Universitaet Mainz
   Austin, TX  78712, USA                 D-55099 Mainz, Germany

                       Version 1.0


                                   Normal Coordinates

               A'                         A''                          A'
             719.17                     782.13                       883.84
            VIBRATION                  VIBRATION                    VIBRATION
 C       0.062 0.6618  0.0000    0.0000  0.0000  0.3694    0.4286 -0.3572  0.0000
 CL     -0.019-0.5637  0.0000    0.0000  0.0000 -0.1636   -0.1272  0.0910  0.0000
 H      -0.030 0.3407  0.0108   -0.0287  0.6380 -0.0752   -0.2663  0.4025  0.0374
 H      -0.030 0.3407 -0.0108    0.0287 -0.6380 -0.0752   -0.2663  0.4025 -0.0374
 H      -0.014 0.0740  0.0000    0.0000  0.0000 -0.0985    0.0234 -0.4418  0.0000

               A'                          A'                         A''
             1073.53                    1279.36                     1332.59
            VIBRATION                  VIBRATION                    VIBRATION
 C       0.271 0.2890  0.0000    0.0520 -0.2828  0.0000    0.0000  0.0000  0.2598
 CL      0.002 0.0065  0.0000    0.0483 -0.0375  0.0000    0.0000  0.0000  0.0038
 H      -0.345-0.3346  0.4314   -0.2531  0.1315  0.0572    0.3252 -0.1278 -0.0321
 H      -0.345-0.3346 -0.4314   -0.2531  0.1315 -0.0572   -0.3252  0.1278 -0.0321
 H       0.022-0.0897  0.0000    0.2517  0.8252  0.0000    0.0000  0.0000 -0.8284

               A'                         A''                          A'
             2252.79                    2363.83                     3152.90
            VIBRATION                  VIBRATION                    VIBRATION
 C       0.231 0.1875  0.0000    0.0000  0.0000  0.4343    0.2745 -0.0620  0.0000
 CL     -0.001-0.0036  0.0000    0.0000  0.0000 -0.0029   -0.0008 -0.0001  0.0000
 H      -0.312-0.2121 -0.5552   -0.2979 -0.2011 -0.5258   -0.0058 -0.0118 -0.0288
 H      -0.312-0.2121  0.5552    0.2979  0.2011 -0.5258   -0.0058 -0.0118  0.0288
 H       0.093-0.0259  0.0000    0.0000  0.0000  0.0051   -0.9259  0.2481  0.0000
            Gradient vector in normal coordinate representation
         ----------------------------------------------------------
   i    W(I)     dE/dQ(i)     dE/dq      dE/dq     [dE/dQ(i)]/w(i)
                             (cm-1)      (eV)        (relative)
         ----------------------------------------------------------
    7   719.17  0.0000000000      0.00000      0.00000   0.0000000000
    8   782.13  0.0000000000      0.00000      0.00000   0.0000000000
    9   883.84  0.0000000000      0.00000      0.00000   0.0000000000
   10  1073.53  0.0000000000      0.00000      0.00000   0.0000000000
   11  1279.36  0.0000000000      0.00000      0.00000   0.0000000000
   12  1332.59  0.0000000000      0.00000      0.00000   0.0000000000
   13  2252.79  0.0000000000      0.00000      0.00000   0.0000000000
   14  2363.83  0.0000000000      0.00000      0.00000   0.0000000000
   15  3152.90  0.0000000000      0.00000      0.00000   0.0000000000
         ----------------------------------------------------------


                      Normal modes in internal coordinates
 ---------------------------------------------------------------------------
              0.072         0.007         0.075         1.296         1.354
 ---------------------------------------------------------------------------
 RCl        0.000000      0.000000      0.000000      0.000000      0.000000
 RH         0.000000      0.000000      0.000000      0.000000      0.000000
 TDA1       0.000000      0.000000      0.000000      0.000002      0.000003
 RH         0.000000      0.000000      0.000000      0.000000      0.000000
```

```
TDA1       0.000000      0.000000      0.000000      0.000002     -0.000003
D120       0.000000      0.000000      0.000000      0.000003      0.000000
RH         0.000000      0.000000      0.000000      0.000001      0.000000
TDA1       0.000000      0.000000      0.000000     -0.000003      0.000000
D120       0.000000      0.000000      0.000000     -0.000001     -0.000002
---------------------------------------------------------------------------
              5.313       719.170       782.129       883.840      1073.533
---------------------------------------------------------------------------
RCl        0.000000      0.949091      0.000000     -0.236890      0.137008
RH         0.000000      0.014356      0.023541      0.013656     -0.008861
TDA1       0.000001      0.100392      0.646416      0.428477     -0.250074
RH         0.000000      0.014356     -0.023541      0.013656     -0.008861
TDA1      -0.000001      0.100392     -0.646416      0.428477     -0.250074
D120       0.000000      0.143335      0.000000      0.573441      0.808943
RH         0.000000     -0.003300      0.000000      0.005932      0.009294
TDA1       0.000000     -0.230140      0.000000     -0.406506     -0.194742
D120      -0.000003     -0.071668     -0.403961     -0.286720     -0.404472
---------------------------------------------------------------------------
           1279.362      1332.587      2252.792      2363.827      3152.903
---------------------------------------------------------------------------
RCl       -0.144280      0.000000      0.069503      0.000000     -0.020159
RH         0.004047     -0.007614     -0.697790     -0.696753     -0.049796
TDA1       0.179063     -0.051775     -0.005018      0.067346      0.025617
RH         0.004047      0.007614     -0.697790      0.696753     -0.049796
TDA1       0.179063      0.051775     -0.005018     -0.067346      0.025617
D120       0.357509      0.000000      0.098114      0.000000      0.073571
RH         0.045566      0.000000     -0.062810      0.000000      0.992706
TDA1       0.867860      0.000000     -0.072940      0.000000     -0.033026
D120      -0.178754     -0.997258     -0.049057     -0.141420     -0.036786
---------------------------------------------------------------------------

  ----------------------------------------------------------------
                  Dipole Moment Function
                 (Normal Coordinate Basis)
  ----------------------------------------------------------------
    Mode     Symmetry    d(Mu(x))/dQ   d(Mu(y))/dQ   d(Mu(z))/dQ
  ----------------------------------------------------------------
     Q7          A'       -0.008002      0.132310      0.000000
     Q8         A''        0.000000      0.000000     -0.014836
     Q9          A'       -0.024521     -0.075551      0.000000
     Q10         A'       -0.047857      0.061641      0.000000
     Q11         A'       -0.007183     -0.079667      0.000000
     Q12        A''        0.000000      0.000000     -0.077461
     Q13         A'        0.019207      0.109512      0.000000
     Q14        A''        0.000000      0.000000      0.047297
     Q15         A'        0.053949     -0.077611      0.000000
  ----------------------------------------------------------------
---------------------------------------------------------------------------
------------------------------------------------------------
        Parameter               (MHz)                  (CM-1)
------------------------------------------------------------


           R6               -.141944E-04           -.473474E-09
           R5               -.123279E-01           -.411214E-06
           SI                            0.555878E+03

  A-reduced centrifugal distortion parameters

           DJ               0.124356E-01           0.414806E-06
           DK               0.925514E+00           0.308718E-04
           DJK              0.126376E+00           0.421546E-05
           DELJ             0.124640E-01           0.415753E-06
           DELK             0.925656E+00           0.308766E-04
           DELJK            0.126206E+00           0.420978E-05
           delJ             0.299622E-03           0.999430E-08
           delK             0.562172E-01           0.187520E-05

  S-reduced centrifugal distortion parameters

           DJ               0.124134E-01           0.414066E-06
           DK               0.925403E+00           0.308681E-04
           DJK              0.126509E+00           0.421990E-05
           D1               -.299622E-03           -.999430E-08
           D2               -.252830E-04           -.843352E-09
------------------------------------------------------------
   Vibrational frequencies after rotational projection  of
   Cartesian force constants:
            1               0.0000i
```

```
          2              0.0000i
          3              0.0000i
          4              0.0000
          5              0.0000
          6              0.0000
          7            719.1695
          8            782.1286
          9            883.8401
         10           1073.5334
         11           1279.3616
         12           1332.5867
         13           2252.7922
         14           2363.8272
         15           3152.9030
 Zero-point vibrational energy:   19.7855 kcal/mol =   82.7825 kJ/mol.
              0.50 seconds walltime passed
 --executable xjoda finished with status           0
```

Table S.6. Part of the CFOUR output file of the CCSD(T)/V5Z-aV(5+*d*)Z harmonic frequency calculation for $^{12}CHD_2{}^{37}Cl$

```
   **************************************************************************
         <<<     CCCCCC     CCCCCC   |||     CCCCCC     CCCCCC   >>>
       <<<      CCC        CCC       |||    CCC        CCC         >>>
      <<<      CCC        CCC        |||   CCC        CCC            >>>
    <<<        CCC        CCC        |||   CCC        CCC              >>>
      <<<      CCC        CCC        |||   CCC        CCC            >>>
       <<<      CCC        CCC       |||    CCC        CCC         >>>
         <<<     CCCCCC     CCCCCC   |||     CCCCCC     CCCCCC   >>>
   **************************************************************************

     ****************************************************************
     * CFOUR Coupled-Cluster techniques for Computational Chemistry *
     ****************************************************************


   Department of Chemistry                Institut fuer Physikalische Chemie
   University of Texas at Austin          Universitaet Mainz
   Austin, TX  78712, USA                 D-55099 Mainz, Germany

                       Version 1.0

                                   Normal Coordinates

              A'                         A''                         A'
            712.95                     781.56                      883.26
          VIBRATION                  VIBRATION                   VIBRATION
 C      0.060 0.6697  0.0000    0.0000  0.0000  0.3697    0.4291 -0.3539  0.0000
 CL    -0.018-0.5538  0.0000    0.0000  0.0000 -0.1592   -0.1240  0.0859  0.0000
 H     -0.029 0.3409  0.0105   -0.0287  0.6385 -0.0753   -0.2663  0.4043  0.0375
 H     -0.029 0.3409 -0.0105    0.0287 -0.6385 -0.0753   -0.2663  0.4043 -0.0375
 H     -0.014 0.0794  0.0000    0.0000  0.0000 -0.0986    0.0231 -0.4425  0.0000

              A'                          A'                        A''
            1073.53                    1279.23                     1332.59
          VIBRATION                  VIBRATION                   VIBRATION
 C      0.271 0.2891  0.0000    0.0521 -0.2834  0.0000    0.0000  0.0000  0.2598
 CL     0.002 0.0063  0.0000    0.0469 -0.0362  0.0000    0.0000  0.0000  0.0037
 H     -0.345-0.3345  0.4314   -0.2532  0.1316  0.0572    0.3252 -0.1279 -0.0321
 H     -0.345-0.3345 -0.4314   -0.2532  0.1316 -0.0572   -0.3252  0.1279 -0.0321
 H      0.022-0.0897  0.0000    0.2519  0.8250  0.0000    0.0000  0.0000 -0.8284

              A'                         A''                         A'
            2252.79                    2363.83                     3152.90
          VIBRATION                  VIBRATION                   VIBRATION
 C      0.231 0.1874  0.0000    0.0000  0.0000  0.4343    0.2745 -0.0620  0.0000
 CL    -0.001-0.0035  0.0000    0.0000  0.0000 -0.0028   -0.0008 -0.0001  0.0000
 H     -0.312-0.2121 -0.5552   -0.2979 -0.2011 -0.5258   -0.0058 -0.0118 -0.0288
 H     -0.312-0.2121  0.5552    0.2979  0.2011 -0.5258   -0.0058 -0.0118  0.0288
 H      0.093-0.0259  0.0000    0.0000  0.0000  0.0051   -0.9258  0.2482  0.0000
          Gradient vector in normal coordinate representation
        ------------------------------------------------------------
   i    W(I)     dE/dQ(i)     dE/dq      dE/dq     [dE/dQ(i)]/w(i)
                              (cm-1)      (eV)        (relative)
        ------------------------------------------------------------
    7   712.95  0.0000000000      0.00000      0.00000   0.0000000000
    8   781.56  0.0000000000      0.00000      0.00000   0.0000000000
    9   883.26  0.0000000000      0.00000      0.00000   0.0000000000
   10  1073.53  0.0000000000      0.00000      0.00000   0.0000000000
   11  1279.23  0.0000000000      0.00000      0.00000   0.0000000000
   12  1332.59  0.0000000000      0.00000      0.00000   0.0000000000
   13  2252.79  0.0000000000      0.00000      0.00000   0.0000000000
   14  2363.83  0.0000000000      0.00000      0.00000   0.0000000000
   15  3152.90  0.0000000000      0.00000      0.00000   0.0000000000
        ------------------------------------------------------------


                      Normal modes in internal coordinates
 ---------------------------------------------------------------------------
              0.053         0.020         0.061         1.291         1.309
 ---------------------------------------------------------------------------
 RCl        0.000000      0.000000      0.000000      0.000000      0.000000
 RH         0.000000      0.000000      0.000000      0.000000      0.000000
 TDA1       0.000000      0.000000      0.000000      0.000003      0.000001
 RH         0.000000      0.000000      0.000000      0.000000      0.000000
 TDA1       0.000000      0.000000      0.000000     -0.000003      0.000001
```

```
D120       0.000000      0.000000      0.000000      0.000000      0.000003
RH         0.000000      0.000000      0.000000      0.000000      0.000001
TDA1       0.000000      0.000000      0.000000      0.000000     -0.000003
D120       0.000000      0.000000      0.000000     -0.000002     -0.000001
---------------------------------------------------------------------------
              5.217       712.954       781.562       883.261      1073.531
---------------------------------------------------------------------------
RCl        0.000000      0.951721      0.000000     -0.232639      0.137190
RH         0.000000      0.014231      0.023537      0.013715     -0.008858
TDA1       0.000001      0.096945      0.646423      0.428726     -0.250062
RH         0.000000      0.014231     -0.023537      0.013715     -0.008858
TDA1      -0.000001      0.096945     -0.646423      0.428726     -0.250062
D120       0.000000      0.138479      0.000000      0.573786      0.808918
RH         0.000000     -0.003318      0.000000      0.005896      0.009292
TDA1       0.000000     -0.225931      0.000000     -0.407821     -0.194774
D120      -0.000004     -0.069239     -0.403939     -0.286893     -0.404459
---------------------------------------------------------------------------
           1279.234      1332.586      2252.791      2363.826      3152.903
---------------------------------------------------------------------------
RCl       -0.145329      0.000000      0.069456      0.000000     -0.020160
RH         0.004044     -0.007613     -0.697793     -0.696754     -0.049796
TDA1       0.179334     -0.051751     -0.005022      0.067337      0.025616
RH         0.004044      0.007613     -0.697793      0.696754     -0.049796
TDA1       0.179334      0.051751     -0.005022     -0.067337      0.025616
D120       0.357849      0.000000      0.098111      0.000000      0.073570
RH         0.045560      0.000000     -0.062809      0.000000      0.992706
TDA1       0.867398      0.000000     -0.072934      0.000000     -0.033023
D120      -0.178925     -0.997260     -0.049055     -0.141415     -0.036785
---------------------------------------------------------------------------

  ----------------------------------------------------------------
                  Dipole Moment Function
                 (Normal Coordinate Basis)
  ----------------------------------------------------------------
    Mode     Symmetry    d(Mu(x))/dQ   d(Mu(y))/dQ   d(Mu(z))/dQ
  ----------------------------------------------------------------
     Q7          A'       -0.007830      0.131492      0.000000
     Q8         A''        0.000000      0.000000     -0.015117
     Q9          A'       -0.024806     -0.074464      0.000000
     Q10         A'       -0.047845      0.061700      0.000000
     Q11         A'       -0.007118     -0.079937      0.000000
     Q12        A''        0.000000      0.000000     -0.077454
     Q13         A'        0.019220      0.109493      0.000000
     Q14        A''        0.000000      0.000000      0.047292
     Q15         A'        0.053937     -0.077620      0.000000
  ----------------------------------------------------------------
---------------------------------------------------------------------------
------------------------------------------------------------
        Parameter               (MHz)               (CM-1)
------------------------------------------------------------


           R6              -.132919E-04          -.443371E-09
           R5              -.119581E-01          -.398880E-06
           SI                           0.575974E+03

  A-reduced centrifugal distortion parameters

           DJ              0.120525E-01          0.402029E-06
           DK              0.929567E+00          0.310070E-04
           DJK             0.122603E+00          0.408959E-05
           DELJ            0.120791E-01          0.402916E-06
           DELK            0.929700E+00          0.310115E-04
           DELJK           0.122443E+00          0.408427E-05
           delJ            0.285896E-03          0.953646E-08
           delK            0.545395E-01          0.181924E-05

  S-reduced centrifugal distortion parameters

           DJ              0.120318E-01          0.401337E-06
           DK              0.929464E+00          0.310036E-04
           DJK             0.122728E+00          0.409375E-05
           D1              -.285896E-03          -.953646E-08
           D2              -.236727E-04          -.789637E-09
------------------------------------------------------------
   Vibrational frequencies after rotational projection  of
   Cartesian force constants:
            1              0.0000i
            2              0.0000i
```

```
           3              0.0000
           4              0.0000
           5              0.0000
           6              0.0000
           7            712.9536
           8            781.5625
           9            883.2610
          10           1073.5310
          11           1279.2338
          12           1332.5861
          13           2252.7908
          14           2363.8263
          15           3152.9027
  Zero-point vibrational energy:   19.7747 kcal/mol =   82.7376 kJ/mol.
              2.25 seconds walltime passed
 --executable xjoda finished with status            0
```

Table S.7. Part of the CFOUR output file of the CCSD(T)/V5Z-aV(5+*d*)Z harmonic frequency calculation for $^{13}CHD_2{}^{35}Cl$

```
 *************************************************************************
         <<<     CCCCCC     CCCCCC   |||     CCCCCC     CCCCCC   >>>
       <<<      CCC        CCC       |||    CCC        CCC         >>>
      <<<      CCC        CCC        |||   CCC        CCC            >>>
    <<<        CCC        CCC        |||   CCC        CCC              >>>
      <<<      CCC        CCC        |||   CCC        CCC            >>>
       <<<      CCC        CCC       |||    CCC        CCC         >>>
         <<<     CCCCCC     CCCCCC   |||     CCCCCC     CCCCCC   >>>
    *************************************************************************

      ****************************************************************
      * CFOUR Coupled-Cluster techniques for Computational Chemistry *
      ****************************************************************


    Department of Chemistry                Institut fuer Physikalische Chemie
    University of Texas at Austin          Universitaet Mainz
    Austin, TX  78712, USA                 D-55099 Mainz, Germany

                        Version 1.0



                                    Normal Coordinates

                A'                         A''                          A'
              706.18                      777.90                      873.26
             VIBRATION                   VIBRATION                   VIBRATION
  C       0.051 0.6801  0.0000    0.0000  0.0000  0.3656    0.4237 -0.3306  0.0000
  CL     -0.015-0.5770  0.0000    0.0000  0.0000 -0.1644   -0.1283  0.0734  0.0000
  H      -0.026 0.3114  0.0139   -0.0282  0.6372 -0.0835   -0.2766  0.4184  0.0390
  H      -0.026 0.3114 -0.0139    0.0282 -0.6372 -0.0835   -0.2766  0.4184 -0.0390
  H      -0.017 0.0754  0.0000    0.0000  0.0000 -0.1086    0.0162 -0.4276  0.0000

                A'                          A'                         A''
              1067.01                     1275.41                     1329.06
             VIBRATION                   VIBRATION                   VIBRATION
  C       0.263 0.2775  0.0000    0.0494 -0.2632  0.0000    0.0000  0.0000  0.2542
  CL      0.002 0.0145  0.0000    0.0493 -0.0420  0.0000    0.0000  0.0000  0.0045
  H      -0.346-0.3462  0.4261   -0.2543  0.1279  0.0648    0.3220 -0.1339 -0.0387
  H      -0.346-0.3462 -0.4261   -0.2543  0.1279 -0.0648   -0.3220  0.1339 -0.0387
  H       0.015-0.1034  0.0000    0.2509  0.8311  0.0000    0.0000  0.0000 -0.8301

                A'                         A''                          A'
              2245.06                     2346.63                     3143.30
             VIBRATION                   VIBRATION                   VIBRATION
  C       0.225 0.1780  0.0000    0.0000  0.0000  0.4186    0.2629 -0.0606  0.0000
  CL     -0.001-0.0031  0.0000    0.0000  0.0000 -0.0027   -0.0007 -0.0003  0.0000
  H      -0.313-0.2118 -0.5584   -0.3017 -0.2025 -0.5294   -0.0039 -0.0111 -0.0269
  H      -0.313-0.2118  0.5584    0.3017  0.2025 -0.5294   -0.0039 -0.0111  0.0269
  H       0.086-0.0225  0.0000    0.0000  0.0000  0.0088   -0.9288  0.2508  0.0000
             Gradient vector in normal coordinate representation
          ----------------------------------------------------------
    i    W(I)     dE/dQ(i)     dE/dq      dE/dq     [dE/dQ(i)]/w(i)
                              (cm-1)      (eV)        (relative)
          ----------------------------------------------------------
     7   706.18  0.0000000000      0.00000      0.00000   0.0000000000
     8   777.90  0.0000000000      0.00000      0.00000   0.0000000000
     9   873.26  0.0000000000      0.00000      0.00000   0.0000000000
    10  1067.01  0.0000000000      0.00000      0.00000   0.0000000000
    11  1275.41  0.0000000000      0.00000      0.00000   0.0000000000
    12  1329.06  0.0000000000      0.00000      0.00000   0.0000000000
    13  2245.06  0.0000000000      0.00000      0.00000   0.0000000000
    14  2346.63  0.0000000000      0.00000      0.00000   0.0000000000
    15  3143.30  0.0000000000      0.00000      0.00000   0.0000000000
          ----------------------------------------------------------

                          Normal modes in internal coordinates
 ---------------------------------------------------------------------------
               0.096          0.054          0.111          1.277          1.330
 ---------------------------------------------------------------------------
 RCl         0.000000       0.000000       0.000000       0.000000       0.000000
 RH          0.000000       0.000000       0.000000       0.000000       0.000000
 TDA1        0.000000       0.000000       0.000000       0.000003       0.000002
```

```
RH          0.000000      0.000000      0.000000      0.000000      0.000000
TDA1        0.000000      0.000000      0.000000     -0.000003      0.000002
D120        0.000000      0.000000      0.000000      0.000000      0.000003
RH          0.000000      0.000000      0.000000      0.000000      0.000001
TDA1        0.000000      0.000000      0.000000      0.000000     -0.000003
D120        0.000000      0.000000      0.000000     -0.000002     -0.000001
---------------------------------------------------------------------------
               5.216       706.184       777.897       873.259      1067.010
---------------------------------------------------------------------------
RCl         0.000000      0.960052      0.000000     -0.210285      0.125590
RH          0.000000      0.011099      0.021345      0.013384     -0.011678
TDA1        0.000001      0.067444      0.644826      0.430020     -0.256372
RH          0.000000      0.011099     -0.021345      0.013384     -0.011678
TDA1       -0.000001      0.067444     -0.644826      0.430020     -0.256372
D120        0.000000      0.126702      0.000000      0.580792      0.806028
RH          0.000000     -0.004520      0.000000      0.007991      0.009702
TDA1        0.000000     -0.221064      0.000000     -0.404814     -0.200761
D120       -0.000004     -0.063351     -0.409252     -0.290396     -0.403014
---------------------------------------------------------------------------
            1275.411      1329.056      2245.063      2346.629      3143.304
---------------------------------------------------------------------------
RCl        -0.126870      0.000000      0.063771      0.000000     -0.018805
RH          0.005196     -0.011596     -0.699269     -0.698342     -0.045772
TDA1        0.168208     -0.056603     -0.003436      0.063294      0.023612
RH          0.005196      0.011596     -0.699269      0.698342     -0.045772
TDA1        0.168208      0.056603     -0.003436     -0.063294      0.023612
D120        0.365048      0.000000      0.090579      0.000000      0.067017
RH          0.046774      0.000000     -0.057029      0.000000      0.993860
TDA1        0.870918      0.000000     -0.066684      0.000000     -0.031116
D120       -0.182524     -0.996656     -0.045289     -0.128933     -0.033509
---------------------------------------------------------------------------


  ----------------------------------------------------------------
                  Dipole Moment Function
                 (Normal Coordinate Basis)
  ----------------------------------------------------------------
    Mode     Symmetry    d(Mu(x))/dQ   d(Mu(y))/dQ   d(Mu(z))/dQ
  ----------------------------------------------------------------
     Q7          A'       -0.007645      0.133632      0.000000
     Q8         A''        0.000000      0.000000     -0.016081
     Q9          A'       -0.026087     -0.068718      0.000000
     Q10         A'       -0.047793      0.058854      0.000000
     Q11         A'       -0.007955     -0.074512      0.000000
     Q12        A''        0.000000      0.000000     -0.077647
     Q13         A'        0.019557      0.106321      0.000000
     Q14        A''        0.000000      0.000000      0.046905
     Q15         A'        0.053225     -0.077325      0.000000
  ----------------------------------------------------------------
---------------------------------------------------------------------------
------------------------------------------------------------
        Parameter              (MHz)                (CM-1)
------------------------------------------------------------

           R6               -.128349E-04          -.428126E-09
           R5               -.116901E-01          -.389940E-06
           SI                            0.594361E+03

  A-reduced centrifugal distortion parameters

           DJ               0.118072E-01          0.393845E-06
           DK               0.928660E+00          0.309768E-04
           DJK              0.122045E+00          0.407100E-05
           DELJ             0.118328E-01          0.394701E-06
           DELK             0.928789E+00          0.309811E-04
           DELJK            0.121891E+00          0.406586E-05
           delJ             0.274458E-03          0.915492E-08
           delK             0.538945E-01          0.179773E-05

  S-reduced centrifugal distortion parameters

           DJ               0.117875E-01          0.393189E-06
           DK               0.928562E+00          0.309735E-04
           DJK              0.122163E+00          0.407493E-05
           D1               -.274458E-03          -.915492E-08
           D2               -.226691E-04          -.756160E-09
------------------------------------------------------------
   Vibrational frequencies after rotational projection  of
   Cartesian force constants:
```

```
           1              0.0000i
           2              0.0000i
           3              0.0000i
           4              0.0000
           5              0.0000
           6              0.0000
           7            706.1843
           8            777.8969
           9            873.2590
          10           1067.0098
          11           1275.4113
          12           1329.0561
          13           2245.0634
          14           2346.6288
          15           3143.3041
 Zero-point vibrational energy:   19.6763 kcal/mol =   82.3259 kJ/mol.
              0.12 seconds walltime passed
 --executable xjoda finished with status           0
```

Table S.8. Part of the CFOUR output file of the CCSD(T)/V5Z-aV(5+*d*)Z harmonic frequency calculation for $^{13}CHD_2{}^{37}Cl$

```
--invoking executable xjoda


   ***********************************************************************
         <<<     CCCCCC     CCCCCC   |||     CCCCCC     CCCCCC   >>>
       <<<      CCC        CCC       |||    CCC        CCC         >>>
      <<<      CCC        CCC        |||   CCC        CCC            >>>
    <<<        CCC        CCC        |||   CCC        CCC              >>>
      <<<      CCC        CCC        |||   CCC        CCC            >>>
       <<<      CCC        CCC       |||    CCC        CCC         >>>
         <<<     CCCCCC     CCCCCC   |||     CCCCCC     CCCCCC   >>>
   ***********************************************************************

     ****************************************************************
     * CFOUR Coupled-Cluster techniques for Computational Chemistry *
     ****************************************************************


   Department of Chemistry                Institut fuer Physikalische Chemie
   University of Texas at Austin          Universitaet Mainz
   Austin, TX  78712, USA                 D-55099 Mainz, Germany

                       Version 1.0



                                   Normal Coordinates

              A'                        A''                        A'
             699.79                    777.33                     872.75
           VIBRATION                 VIBRATION                  VIBRATION
 C      0.048 0.6877  0.0000    0.0000  0.0000  0.3659    0.4241 -0.3279  0.0000
 CL    -0.014-0.5668  0.0000    0.0000  0.0000 -0.1601   -0.1250  0.0692  0.0000
 H     -0.024 0.3122  0.0135   -0.0282  0.6376 -0.0836   -0.2766  0.4198  0.0390
 H     -0.024 0.3122 -0.0135    0.0282 -0.6376 -0.0836   -0.2766  0.4198 -0.0390
 H     -0.017 0.0803  0.0000    0.0000  0.0000 -0.1086    0.0158 -0.4283  0.0000

              A'                         A'                        A''
            1067.00                   1275.27                    1329.06
           VIBRATION                 VIBRATION                  VIBRATION
 C      0.263 0.2778  0.0000    0.0495 -0.2637  0.0000    0.0000  0.0000  0.2542
 CL     0.002 0.0139  0.0000    0.0479 -0.0405  0.0000    0.0000  0.0000  0.0043
 H     -0.346-0.3461  0.4261   -0.2544  0.1280  0.0648    0.3220 -0.1340 -0.0387
 H     -0.346-0.3461 -0.4261   -0.2544  0.1280 -0.0648   -0.3220  0.1340 -0.0387
 H      0.015-0.1034  0.0000    0.2511  0.8309  0.0000    0.0000  0.0000 -0.8301

              A'                        A''                        A'
            2245.06                   2346.63                    3143.30
           VIBRATION                 VIBRATION                  VIBRATION
 C      0.225 0.1780  0.0000    0.0000  0.0000  0.4186    0.2629 -0.0607  0.0000
 CL    -0.001-0.0030  0.0000    0.0000  0.0000 -0.0026   -0.0007 -0.0003  0.0000
 H     -0.313-0.2118 -0.5584   -0.3018 -0.2024 -0.5294   -0.0039 -0.0111 -0.0269
 H     -0.313-0.2118  0.5584    0.3018  0.2024 -0.5294   -0.0039 -0.0111  0.0269
 H      0.086-0.0225  0.0000    0.0000  0.0000  0.0088   -0.9287  0.2509  0.0000
             Gradient vector in normal coordinate representation
          ----------------------------------------------------------
   i    W(I)     dE/dQ(i)     dE/dq      dE/dq     [dE/dQ(i)]/w(i)
                             (cm-1)      (eV)        (relative)
          ----------------------------------------------------------
    7   699.79  0.0000000000      0.00000      0.00000   0.0000000000
    8   777.33  0.0000000000      0.00000      0.00000   0.0000000000
    9   872.75  0.0000000000      0.00000      0.00000   0.0000000000
   10  1067.00  0.0000000000      0.00000      0.00000   0.0000000000
   11  1275.27  0.0000000000      0.00000      0.00000   0.0000000000
   12  1329.06  0.0000000000      0.00000      0.00000   0.0000000000
   13  2245.06  0.0000000000      0.00000      0.00000   0.0000000000
   14  2346.63  0.0000000000      0.00000      0.00000   0.0000000000
   15  3143.30  0.0000000000      0.00000      0.00000   0.0000000000
          ----------------------------------------------------------

                           Normal modes in internal coordinates
--------------------------------------------------------------------------
              0.100         0.044         0.109         1.288         1.322
--------------------------------------------------------------------------
```

```
RCl         0.000000      0.000000      0.000000      0.000000      0.000000
RH          0.000000      0.000000      0.000000      0.000000      0.000000
TDA1        0.000000      0.000000      0.000000      0.000003      0.000002
RH          0.000000      0.000000      0.000000      0.000000      0.000000
TDA1        0.000000      0.000000      0.000000     -0.000003      0.000002
D120        0.000000      0.000000      0.000000      0.000000      0.000003
RH          0.000000      0.000000      0.000000      0.000000      0.000001
TDA1        0.000000      0.000000      0.000000      0.000000     -0.000003
D120        0.000000      0.000000      0.000000     -0.000002     -0.000001
----------------------------------------------------------------------------
               5.235       699.794       777.329       872.746      1067.004
----------------------------------------------------------------------------
RCl         0.000000      0.961930      0.000000     -0.206836      0.126010
RH          0.000000      0.011006      0.021342      0.013418     -0.011674
TDA1        0.000001      0.064815      0.644835      0.430102     -0.256383
RH          0.000000      0.011006     -0.021342      0.013418     -0.011674
TDA1       -0.000001      0.064815     -0.644835      0.430102     -0.256383
D120        0.000000      0.122537      0.000000      0.581060      0.805954
RH          0.000000     -0.004540      0.000000      0.007955      0.009697
TDA1        0.000000     -0.217388      0.000000     -0.405934     -0.200845
D120       -0.000004     -0.061268     -0.409225     -0.290530     -0.402977
----------------------------------------------------------------------------
            1275.268      1329.056      2245.063      2346.629      3143.305
----------------------------------------------------------------------------
RCl        -0.128011      0.000000      0.063730      0.000000     -0.018807
RH          0.005193     -0.011595     -0.699272     -0.698344     -0.045772
TDA1        0.168468     -0.056575     -0.003439      0.063285      0.023611
RH          0.005193      0.011595     -0.699272      0.698344     -0.045772
TDA1        0.168468      0.056575     -0.003439     -0.063285      0.023611
D120        0.365424      0.000000      0.090575      0.000000      0.067016
RH          0.046770      0.000000     -0.057029      0.000000      0.993861
TDA1        0.870454      0.000000     -0.066678      0.000000     -0.031114
D120       -0.182712     -0.996659     -0.045288     -0.128928     -0.033508
----------------------------------------------------------------------------

  -----------------------------------------------------------------
                  Dipole Moment Function
                 (Normal Coordinate Basis)
  -----------------------------------------------------------------
    Mode     Symmetry    d(Mu(x))/dQ   d(Mu(y))/dQ   d(Mu(z))/dQ
  -----------------------------------------------------------------
     Q7          A'       -0.007476      0.132633      0.000000
     Q8         A''        0.000000      0.000000     -0.016364
     Q9          A'       -0.026364     -0.067828      0.000000
     Q10         A'       -0.047781      0.058979      0.000000
     Q11         A'       -0.007890     -0.074802      0.000000
     Q12        A''        0.000000      0.000000     -0.077640
     Q13         A'        0.019570      0.106304      0.000000
     Q14        A''        0.000000      0.000000      0.046901
     Q15         A'        0.053213     -0.077334      0.000000
  -----------------------------------------------------------------
----------------------------------------------------------------------------
--------------------------------------------------------------
        Parameter               (MHz)                 (CM-1)
--------------------------------------------------------------

           R6               -.119858E-04           -.399804E-09
           R5               -.113241E-01           -.377730E-06
           SI                            0.616702E+03

  A-reduced centrifugal distortion parameters

           DJ                0.114285E-01           0.381215E-06
           DK                0.932701E+00           0.311116E-04
           DJK               0.118277E+00           0.394529E-05
           DELJ              0.114525E-01           0.382015E-06
           DELK              0.932821E+00           0.311156E-04
           DELJK             0.118133E+00           0.394049E-05
           delJ              0.261359E-03           0.871800E-08
           delK              0.522149E-01           0.174170E-05

  S-reduced centrifugal distortion parameters

           DJ                0.114102E-01           0.380603E-06
           DK                0.932610E+00           0.311085E-04
           DJK               0.118387E+00           0.394896E-05
           D1               -.261359E-03           -.871800E-08
           D2               -.211670E-04           -.706054E-09
```

```
--------------------------------------------------------------
   Vibrational frequencies after rotational projection  of
   Cartesian force constants:
            1              0.0000i
            2              0.0000i
            3              0.0000i
            4              0.0000
            5              0.0000
            6              0.0000
            7            699.7944
            8            777.3288
            9            872.7463
           10           1067.0044
           11           1275.2679
           12           1329.0561
           13           2245.0630
           14           2346.6288
           15           3143.3049
  Zero-point vibrational energy:   19.6654 kcal/mol =   82.2803 kJ/mol.
              0.38 seconds walltime passed
 --executable xjoda finished with status           0
```

Table S.9. Part of the CFOUR output file of the CCSD(T)/VQZ-aV(Q+*d*)Z anharmonic frequency calculation for $^{12}CHD_2{}^{35}Cl$

```
   *************************************************************************
         <<<     CCCCCC     CCCCCC   |||     CCCCCC     CCCCCC   >>>
       <<<      CCC        CCC       |||    CCC        CCC         >>>
      <<<      CCC        CCC        |||   CCC        CCC            >>>
     <<<       CCC        CCC        |||   CCC        CCC              >>>
      <<<      CCC        CCC        |||   CCC        CCC            >>>
       <<<      CCC        CCC       |||    CCC        CCC         >>>
         <<<     CCCCCC     CCCCCC   |||     CCCCCC     CCCCCC   >>>
   *************************************************************************

     ****************************************************************
     * CFOUR Coupled-Cluster techniques for Computational Chemistry *
     ****************************************************************


   Department of Chemistry                Institut fuer Physikalische Chemie
   University of Texas at Austin          Universitaet Mainz
   Austin, TX  78712, USA                 D-55099 Mainz, Germany

                       Version 1.0



                                   Normal Coordinates

                A'                         A''                        A'
              718.57                     781.05                     881.33
             VIBRATION                  VIBRATION                  VIBRATION
 C       0.067 0.6600  0.0000    0.0000  0.0000  0.3718    0.4306 -0.3570  0.0000
 CL     -0.021-0.5628  0.0000    0.0000  0.0000 -0.1636   -0.1295  0.0967  0.0000
 H      -0.031 0.3432  0.0113   -0.0233  0.6371 -0.0775   -0.2631  0.3951  0.0387
 H      -0.031 0.3432 -0.0113    0.0233 -0.6371 -0.0775   -0.2631  0.3951 -0.0387
 H      -0.015 0.0669  0.0000    0.0000  0.0000 -0.1001    0.0208 -0.4548  0.0000

                A'                          A'                        A''
              1073.05                    1269.13                    1324.63
             VIBRATION                  VIBRATION                  VIBRATION
 C       0.268 0.2948  0.0000    0.0601 -0.2812  0.0000    0.0000  0.0000  0.2598
 CL      0.002 0.0072  0.0000    0.0483 -0.0382  0.0000    0.0000  0.0000  0.0042
 H      -0.338-0.3393  0.4306   -0.2635  0.1342  0.0651    0.3299 -0.1321 -0.0347
 H      -0.338-0.3393 -0.4306   -0.2635  0.1342 -0.0651   -0.3299  0.1321 -0.0347
 H       0.019-0.0999  0.0000    0.2528  0.8163  0.0000    0.0000  0.0000 -0.8231

                A'                         A''                        A'
              2251.67                    2361.61                    3202.70
             VIBRATION                  VIBRATION                  VIBRATION
 C       0.232 0.1873  0.0000    0.0000  0.0000  0.4338    0.2741 -0.0631  0.0000
 CL     -0.001-0.0037  0.0000    0.0000  0.0000 -0.0029   -0.0008 -0.0001  0.0000
 H      -0.312-0.2124 -0.5550   -0.2983 -0.2013 -0.5257   -0.0053 -0.0110 -0.0270
 H      -0.312-0.2124  0.5550    0.2983  0.2013 -0.5257   -0.0053 -0.0110  0.0270
 H       0.090-0.0243  0.0000    0.0000  0.0000  0.0068   -0.9257  0.2494  0.0000
             Gradient vector in normal coordinate representation
          ---------------------------------------------------------
    i    W(I)     dE/dQ(i)     dE/dq      dE/dq     [dE/dQ(i)]/w(i)
                              (cm-1)      (eV)        (relative)
          ---------------------------------------------------------
     7   718.57 -0.0000002244     -0.02016      0.00000   0.0000000000
     8   781.05  0.0000000000      0.00000      0.00000   0.0000000000
     9   881.33  0.0000006107      0.04954      0.00001   0.0000000000
    10  1073.05  0.0000005087      0.03740      0.00000   0.0000000000
    11  1269.13  0.0000022090      0.14933      0.00002   0.0000000000
    12  1324.63  0.0000000000      0.00000      0.00000   0.0000000000
    13  2251.67 -0.0000074473     -0.37796     -0.00005   0.0000000000
    14  2361.61  0.0000000000      0.00000      0.00000   0.0000000000
    15  3202.70 -0.0037340810   -158.89939     -0.01970   0.0000021356
          ---------------------------------------------------------

                         Normal modes in internal coordinates
 ---------------------------------------------------------------------------
             95.671        35.741          0.011          0.013          0.045
 ---------------------------------------------------------------------------
 RCl        0.000000     -0.000468       0.000000       0.000000       0.000000
 RH         0.000100      0.000015       0.000000       0.000000       0.000000
 TDA1      -0.001493     -0.000239       0.000000       0.000000       0.000000
```

```
RH        -0.000100      0.000015      0.000000      0.000000      0.000000
TDA1       0.001493     -0.000239      0.000000      0.000000      0.000000
D120       0.000000     -0.000808      0.000000      0.000000      0.000000
RH         0.000000      0.000089      0.000000      0.000000      0.000000
TDA1       0.000000      0.002633      0.000000      0.000000      0.000000
D120       0.006659      0.000404      0.000000      0.000000      0.000000
---------------------------------------------------------------------------
             10.402       718.574       781.047       881.330      1073.052
---------------------------------------------------------------------------
RCl        0.000000      0.943625      0.000000     -0.238483      0.140233
RH         0.000006      0.014511      0.023448      0.013727     -0.008865
TDA1       0.000149      0.104930      0.643991      0.423207     -0.255798
RH        -0.000006      0.014511     -0.023448      0.013727     -0.008865
TDA1      -0.000149      0.104930     -0.643991      0.423207     -0.255798
D120       0.000000      0.150919      0.000000      0.571906      0.803532
RH         0.000000     -0.003123      0.000000      0.004458      0.008122
TDA1       0.000000     -0.242178      0.000000     -0.419136     -0.205346
D120      -0.000177     -0.075459     -0.411645     -0.285953     -0.401766
---------------------------------------------------------------------------
           1269.134      1324.629      2251.671      2361.613      3202.695
---------------------------------------------------------------------------
RCl       -0.143013      0.000000      0.069421      0.000000     -0.020478
RH         0.005462     -0.009415     -0.697928     -0.696877     -0.048256
TDA1       0.180866     -0.054291     -0.004766      0.067453      0.025774
RH         0.005462      0.009415     -0.697928      0.696877     -0.048256
TDA1       0.180866      0.054291     -0.004766     -0.067453      0.025774
D120       0.379290      0.000000      0.098948      0.000000      0.073790
RH         0.044100      0.000000     -0.058648      0.000000      0.992837
TDA1       0.855740      0.000000     -0.072465      0.000000     -0.032600
D120      -0.189645     -0.996959     -0.049474     -0.140089     -0.036895
---------------------------------------------------------------------------

  ----------------------------------------------------------------
                  Dipole Moment Function
                 (Normal Coordinate Basis)
  ----------------------------------------------------------------
    Mode     Symmetry    d(Mu(x))/dQ   d(Mu(y))/dQ   d(Mu(z))/dQ
  ----------------------------------------------------------------
     Q7         A'       -0.008331      0.130930      0.000000
     Q8        A''        0.000000      0.000000     -0.016955
     Q9         A'       -0.026353     -0.075060      0.000000
     Q10        A'       -0.047419      0.064275      0.000000
     Q11        A'       -0.008076     -0.081295      0.000000
     Q12       A''        0.000000      0.000000     -0.076140
     Q13        A'        0.021483      0.109741      0.000000
     Q14       A''        0.000000      0.000000      0.052101
     Q15        A'        0.056871     -0.076301      0.000000
  ----------------------------------------------------------------
---------------------------------------------------------------------------
------------------------------------------------------------
        Parameter               (MHz)                 (CM-1)
------------------------------------------------------------

           R6                 -.139874E-04          -.466570E-09
           R5                 -.120508E-01          -.401970E-06
           SI                             0.553555E+03

  A-reduced centrifugal distortion parameters

           DJ                 0.123981E-01          0.413557E-06
           DK                 0.930803E+00          0.310483E-04
           DJK                0.128062E+00          0.427169E-05
           DELJ               0.124261E-01          0.414490E-06
           DELK               0.930943E+00          0.310529E-04
           DELJK              0.127894E+00          0.426609E-05
           delJ               0.300842E-03          0.100350E-07
           delK               0.550727E-01          0.183703E-05

  S-reduced centrifugal distortion parameters

           DJ                 0.123764E-01          0.412831E-06
           DK                 0.930694E+00          0.310446E-04
           DJK                0.128193E+00          0.427605E-05
           D1                 -.300842E-03          -.100350E-07
           D2                 -.248723E-04          -.829650E-09
------------------------------------------------------------
   Vibrational frequencies after rotational projection  of
   Cartesian force constants:
```

```
          1             0.0000i
          2             0.0000i
          3             0.0000i
          4             0.0000i
          5             0.0000
          6             0.0000
          7           718.5729
          8           781.0264
          9           881.3244
         10          1073.0511
         11          1269.1217
         12          1324.5462
         13          2251.6709
         14          2361.6132
         15          3202.6951
  Zero-point vibrational energy:   19.8190 kcal/mol =   82.9229 kJ/mol.
              0.00 seconds walltime passed
 --executable xjoda finished with status           0
 --invoking executable xcubic
  @GETMEM-I,   Allocated   13351 MB of main memory.
  back



********************************************************************************
        PARAMETERS RELEVANT TO MOLECULAR STRUCTURE
********************************************************************************

  Force Field from Numerical Differentiation of Analytic Second Derivatives
  ----------------------------------------------------------------------------
 Cubic force constants written to file cubic.

----------------------------------------------------------------------------
  Mean and mean-square displacements in dimensionless normal coordinates

             Geometrical properties evaluated at     0.00 K

         Coordinate             <q>            <q**2>
----------------------------------------------------------------------------
            7             0.1320164107     0.4999999889
            8             0.0000000000     0.4999999889
            9            -0.0334764131     0.4999999889
           10             0.0058884200     0.4999999889
           11             0.0118353056     0.4999999889
           12             0.0000000000     0.4999999889
           13            -0.1290163300     0.4999999889
           14             0.0000000000     0.4999999889
           15             0.0784897075     0.4999999889
----------------------------------------------------------------------------

  ------------------------------------------------------
   ATOM             INTERNUCLEAR DISTANCE / Angstrom
  -------------------------------------------------------
  I    J           Re           Rg           Ra
  ------------------------------------------------------
  2    1        1.7826656    1.7908931    1.7905717
  3    1        1.0850773    1.1004835    1.0929730
  3    2        2.3617095    2.3756770    2.3739115
  4    1        1.0850773    1.1004835    1.0929730
  4    2        2.3617095    2.3756770    2.3739115
  4    3        1.7831251    1.8022929    1.7960215
  5    1        1.0850773    1.1062110    1.0942246
  5    2        2.3617095    2.3798912    2.3763141
  5    3        1.7831251    1.8062534    1.7970700
  5    4        1.7831251    1.8062534    1.7970700
  ------------------------------------------------------
********************************************************************************
     PARAMETERS RELEVANT TO ROTATIONAL SPECTROSCOPY
********************************************************************************



  -----------------------------------------------------------------------------
                                   VIB-ROT CONSTANT / (cm-1)
                                ------------------------------
     AXIS        MODE       CORIOLIS      QUADRATIC     ANHARMONIC    TOTAL
  -----------------------------------------------------------------------------
       1          7       0.0006563     -0.0010991     0.0035170     0.0030742
       1          8       0.0003843     -0.0000001     0.0007751     0.0011593
```

1	9	0.0000070	-0.0002227	0.0009862	0.0007705
1	10	0.0001123	-0.0000297	0.0001810	0.0002636
1	11	0.0060005	-0.0000750	0.0007627	0.0066882
1	12	-0.0063108	-0.0000102	-0.0001027	-0.0064237
1	13	-0.0002683	-0.0000276	0.0007256	0.0004296
1	14	-0.0004188	-0.0000048	0.0005426	0.0001190
1	15	-0.0000191	-0.0000040	-0.0000971	-0.0001202
2	7	0.0009497	-0.0000578	0.0033571	0.0042490
2	8	0.0248495	-0.0047677	-0.0185359	0.0015459
2	9	-0.0096434	-0.0035625	-0.0146246	-0.0278305
2	10	0.0237549	-0.0038711	-0.0057027	0.0141810
2	11	0.0403636	-0.0024535	-0.0033257	0.0345843
2	12	-0.0158126	-0.0001123	-0.0054433	-0.0213681
2	13	-0.0028688	-0.0198078	0.0525677	0.0298912
2	14	-0.0342059	-0.0001572	0.0504218	0.0160587
2	15	-0.0199165	-0.0029719	0.0383398	0.0154513
3	7	0.0002197	-0.0011584	0.0036851	0.0027464
3	8	0.0002897	-0.0005801	0.0009985	0.0007081
3	9	0.0016180	-0.0000313	0.0009877	0.0025744
3	10	-0.0012216	-0.0000178	0.0001292	-0.0011103
3	11	0.0000295	-0.0000005	0.0007238	0.0007528
3	12	-0.0001655	-0.0000241	0.0000100	-0.0001797
3	13	-0.0001879	-0.0000050	0.0002813	0.0000885
3	14	-0.0000959	-0.0000241	0.0003470	0.0002271
3	15	-0.0003518	-0.0000070	0.0002832	-0.0000756

Be, B0 AND B-B0 SHIFTS FOR SINGLY EXCITED VIBRATIONAL STATES (CM-1)

VIBRATION	X AXIS	Y AXIS	Z AXIS
Be	0.38017121	3.20840528	0.39029061
B0	0.37719095	3.17502387	0.38742477
Be-B0	0.00298026	0.03338141	0.00286584
B'	0.37718747	3.17502850	0.38742594
Be-B'	0.00298374	0.03337677	0.00286467
B''	0.37719416	3.17502932	0.38742931
Be-B''	0.00297704	0.03337595	0.00286129
B^A	0.37718537	3.17502849	0.38742804
Be-B^A	0.00298583	0.03337678	0.00286256
B^S	0.37718911	3.17502850	0.38742430
Be-B^S	0.00298210	0.03337677	0.00286631
7	-0.00307416	-0.00424905	-0.00274643
8	-0.00115932	-0.00154585	-0.00070807
9	-0.00077054	0.02783052	-0.00257442
10	-0.00026364	-0.01418100	0.00111029
11	-0.00668816	-0.03458432	-0.00075278
12	0.00642372	0.02136810	0.00017966
13	-0.00042962	-0.02989115	-0.00008847
14	-0.00011896	-0.01605872	-0.00022706
15	0.00012017	-0.01545133	0.00007561

Be, B0 AND B-B0 SHIFTS FOR SINGLY EXCITED VIBRATIONAL STATES (MHz)

VIBRATION	X AXIS	Y AXIS	Z AXIS
Be	11397.24608989	96185.57041048	11700.61803626
B0	11307.90025843	95184.82089272	11614.70238667
Be-B0	89.34583145	1000.74951776	85.91564959
B'	11307.79583792	95184.95987365	11614.73750930
Be-B'	89.45025197	1000.61053683	85.88052696
B''	11307.99657534	95184.98447408	11614.83858772
Be-B''	89.24951455	1000.58593640	85.77944854
B^A	11307.73301344	95184.95964796	11614.80055948
Be-B^A	89.51307645	1000.61076252	85.81747678
B^S	11307.84508370	95184.95989936	11614.68828809
Be-B^S	89.40100618	1000.61051112	85.92974817
7	-92.16088932	-127.38330134	-82.33592820
8	-34.75554915	-46.34350914	-21.22743550
9	-23.10020403	834.33787236	-77.17915917
10	-7.90371380	-425.13554512	33.28576198
11	-200.50604902	-1036.81191401	-22.56788075
12	192.57822935	640.59938712	5.38614289
13	-12.87965860	-896.11421317	-2.65233496
14	-3.56647283	-481.42844331	-6.80714394
15	3.60264449	-463.21936889	2.26667848

```
  ----------------------------------------------------------------------------
                Vibrationally averaged dipole moment
------------------------------------------------------------------------
                 a.u.                         Debye
         x         y         z              x         y         z
------------------------------------------------------------------------
MU_e -0.03019   0.75412   0.00000       -0.07674   1.91665   0.00000
<MU> -0.02813   0.74862   0.00000       -0.07148   1.90268   0.00000

Equilibrium dipole moment:    0.75472 a.u. (   1.91818 D)
Equilibrium dipole moment:    0.74915 a.u. (   1.90402 D)
------------------------------------------------------------------------
********************************************************************************
    PARAMETERS RELEVANT TO VIBRATIONAL SPECTROSCOPY
********************************************************************************
  Quartic force constants written to file quartic
  ================================================================
  Performing F(IIJJ)/F(JJII) consistency check
  Differences greater than 1 cm-1 will be printed.
  ================================================================
  I    I    K    K       F(IIKK)       F(KKII)       Difference
  ----------------------------------------------------------------
  ----------------------------------------------------------------
  Largest absolute difference is    0.46814 cm-1.
  Largest relative difference is .32008D-01.
VPT2 vibrational analysis


 Thresholds for removing resonance denominators:
 Delta_omega = 50 cm-1; Phi(ijk) = 80 cm-1

-------------------------------
  ANHARMONICITY CONSTANTS X(ij)
                 (cm-1)
-------------------------------
  (*) Near-zero denominators were removed
-------------------------------
          I  J          X(IJ)
-------------------------------
           7  7       -3.4121
           7  8       -3.7196
           7  9       -4.0086
           7 10       -1.4486
           7 11       -5.5887
           7 12       -0.9215
           7 13       -0.1260
           7 14        0.7965
           7 15        2.3682
           8  8       -0.5109
           8  9       -1.3140
           8 10       -4.0775
           8 11       -0.5246
           8 12       -3.4518
           8 13       -4.0033
           8 14       -3.2081
           8 15        0.5008
           9  9       -2.1739
           9 10       -1.8309
           9 11       -8.7608
           9 12       -4.4351
           9 13        5.4717
           9 14       -2.9957
           9 15       -2.9799
          10 10       -5.5258
          10 11       -1.9862
          10 12        4.3094
          10 13        3.6909
          10 14      -18.7134
          10 15       -1.0806
          11 11       -5.2848
          11 12        1.1528
          11 13        1.3460
          11 14       -2.3987
          11 15      -17.9768
          12 12       -5.5215
          12 13       -6.0736
          12 14      -12.6586
          12 15      -21.9961
```

13	13	-14.9604
13	14	-62.3382
13	15	-0.0204
14	14	-19.6510
14	15	1.4084
15	15	-61.3604

HARMONIC AND FUNDAMENTAL FREQUENCIES (cm-1) AND INTENSITIES (km/mol)

Mode	Harmonic Frequency	Fundamental Frequency	Anharmonic Contribution	Harmonic Intensity	Fundamental Intensity	Anharm Contrib
7	719.1584	706.0100	-13.1484	16.8484	17.8539	1.0055
8	781.6742	770.7533	-10.9209	0.2645	0.2670	0.0025
9	883.4357	868.6612	-14.7744	6.2980	6.1323	-0.1658
10	1073.3561	1051.7360	-21.6201	6.0395	5.6502	-0.3893
11	1278.8086	1250.8706	-27.9380	6.4837	5.8193	-0.6644
12	1332.5687	1299.4886	-33.0802	5.7344	5.8024	0.0680
13	2253.3234	2192.3762	-60.9472	12.1905	11.2089	-0.9815
14	2363.8797	2274.5239	-89.3558	2.5978	2.5673	-0.0305
15	3153.1883	3010.5791	-142.6091	9.2611	10.1088	0.8476

ZERO-POINT VIBRATIONAL ENERGIES

	kcal/mol	kJ/mol	Hartree	cm-1
Harmonic contribution :	19.7844	82.7779	0.03152846	6919.697
VPT2-correction :	-0.2334	-0.9766	-0.00037199	-81.642
Harm+VPT2 :	19.5510	81.8012	0.03115647	6838.055

MAXLEVEL set to 3

All levels with up to three quanta

MODE I	MODE J	MODE K	MODE L	MODE M	NI	NJ	NK	NL	NM	Anharmonic Frequency	Anharm Intensity	Harmonic Transition
7	0	0	0	0	1	0	0	0	0	706.010	17.853576	719.158399
8	0	0	0	0	1	0	0	0	0	770.753	0.266969	781.674223
9	0	0	0	0	1	0	0	0	0	868.661	6.132139	883.435675
10	0	0	0	0	1	0	0	0	0	1051.736	5.650042	1073.356127
11	0	0	0	0	1	0	0	0	0	1250.870	5.819164	1278.808612
12	0	0	0	0	1	0	0	0	0	1299.488	5.802245	1332.568742
7	0	0	0	0	2	0	0	0	0	1405.195	0.129840	1438.316798
8	7	0	0	0	1	1	0	0	0	1473.043	0.012255	1500.832622
8	0	0	0	0	2	0	0	0	0	1540.484	0.001663	1563.348446
9	7	0	0	0	1	1	0	0	0	1570.662	0.002643	1602.594075
9	8	0	0	0	1	1	0	0	0	1638.100	0.020393	1665.109899
9	0	0	0	0	2	0	0	0	0	1732.974	0.050132	1766.871351
10	7	0	0	0	1	1	0	0	0	1756.297	0.043206	1792.514526
10	8	0	0	0	1	1	0	0	0	1818.411	0.065701	1855.030350
10	9	0	0	0	1	1	0	0	0	1918.566	0.043220	1956.791802
11	7	0	0	0	1	1	0	0	0	1951.291	0.064394	1997.967011
12	7	0	0	0	1	1	0	0	0	2004.577	0.002853	2051.727141
11	8	0	0	0	1	1	0	0	0	2021.099	0.004763	2060.482835
12	8	0	0	0	1	1	0	0	0	2066.790	0.083132	2114.242965
10	0	0	0	0	2	0	0	0	0	2092.420	0.795687	2146.712254
7	0	0	0	0	3	0	0	0	0	2097.557	0.000000	2157.475198
11	9	0	0	0	1	1	0	0	0	2110.771	0.613857	2162.244287
12	9	0	0	0	1	1	0	0	0	2163.714	0.000274	2216.004418
8	7	0	0	0	1	2	0	0	0	2168.509	0.000000	2219.991022
13	0	0	0	0	1	0	0	0	0	2192.376	11.208705	2253.323425
8	7	0	0	0	2	1	0	0	0	2239.055	0.000000	2282.506845
9	7	0	0	0	1	2	0	0	0	2265.839	0.000000	2321.752474
14	0	0	0	0	1	0	0	0	0	2274.523	2.567247	2363.879670
11	10	0	0	0	1	1	0	0	0	2300.620	0.218413	2352.164739
8	0	0	0	0	3	0	0	0	0	2309.194	0.000000	2345.022669
9	8	7	0	0	1	1	1	0	0	2336.382	0.000000	2384.268298
12	10	0	0	0	1	1	0	0	0	2355.533	0.813133	2405.924869
9	8	0	0	0	1	2	0	0	0	2406.518	0.000000	2446.784122
9	7	0	0	0	2	1	0	0	0	2430.967	0.000000	2486.029750
10	7	0	0	0	1	2	0	0	0	2454.034	0.000000	2511.672925
11	0	0	0	0	2	0	0	0	0	2491.171	0.154296	2557.617224
9	8	0	0	0	2	1	0	0	0	2501.099	0.000000	2548.545574
10	8	7	0	0	1	1	1	0	0	2519.253	0.000000	2574.188749

12	11	0	0	0	1	1	0	0	0	2551.512	0.047841	2611.377354
10	8	0	0	0	1	2	0	0	0	2584.065	0.000000	2636.704573
12	0	0	0	0	2	0	0	0	0	2587.934	0.151023	2665.137484
9	0	0	0	0	3	0	0	0	0	2592.940	0.000000	2650.307026
10	9	7	0	0	1	1	1	0	0	2619.119	0.000000	2675.950202
11	7	0	0	0	1	2	0	0	0	2644.889	0.000000	2717.125410
10	9	8	0	0	1	1	1	0	0	2683.928	0.000000	2738.466026
12	7	0	0	0	1	2	0	0	0	2702.841	0.000000	2770.885541
11	8	7	0	0	1	1	1	0	0	2717.801	0.000000	2779.641234
12	8	7	0	0	1	1	1	0	0	2768.159	0.000000	2833.401365
10	9	0	0	0	1	2	0	0	0	2781.048	0.000000	2840.227478
11	8	0	0	0	1	2	0	0	0	2790.306	0.000000	2842.157058
10	7	0	0	0	2	1	0	0	0	2795.533	0.000000	2865.870653
11	9	7	0	0	1	1	1	0	0	2807.183	0.000000	2881.402686
12	8	0	0	0	1	2	0	0	0	2833.069	0.000000	2895.917188
10	8	0	0	0	2	1	0	0	0	2855.018	0.000000	2928.386477
12	9	7	0	0	1	1	1	0	0	2864.794	0.000000	2935.162817
11	9	8	0	0	1	1	1	0	0	2879.685	0.000000	2943.918510
13	7	0	0	0	1	1	0	0	0	2898.260	0.092466	2972.481824
12	9	8	0	0	1	1	1	0	0	2929.702	0.000000	2997.678641
10	9	0	0	0	2	1	0	0	0	2957.419	0.000000	3030.147929
13	8	0	0	0	1	1	0	0	0	2959.126	0.002985	3034.997648
11	9	0	0	0	1	2	0	0	0	2966.323	0.000000	3045.679963
14	7	0	0	0	1	1	0	0	0	2981.330	0.016787	3083.038070
11	10	7	0	0	1	1	1	0	0	2999.593	0.000000	3071.323138
15	0	0	0	0	1	0	0	0	0	3010.579	10.108554	3153.188268
12	9	0	0	0	1	2	0	0	0	3023.593	0.000000	3099.440093
14	8	0	0	0	1	1	0	0	0	3042.069	0.538831	3145.553893
12	10	7	0	0	1	1	1	0	0	3059.173	0.000000	3125.083268
13	9	0	0	0	1	1	0	0	0	3066.509	0.737065	3136.759101
11	10	8	0	0	1	1	1	0	0	3066.771	0.000000	3133.838962
12	10	8	0	0	1	1	1	0	0	3118.757	0.000000	3187.599092
10	0	0	0	0	3	0	0	0	0	3122.052	0.000000	3220.068381
14	9	0	0	0	1	1	0	0	0	3140.189	0.011316	3247.315346
11	10	9	0	0	1	1	1	0	0	3158.690	0.000000	3235.600414
11	7	0	0	0	2	1	0	0	0	3186.004	0.000000	3276.775623
12	10	9	0	0	1	1	1	0	0	3217.929	0.000000	3289.360545
13	10	0	0	0	1	1	0	0	0	3247.803	0.057767	3326.679552
12	11	7	0	0	1	1	1	0	0	3251.011	0.000000	3330.535753
11	8	0	0	0	2	1	0	0	0	3260.875	0.000000	3339.291447
12	7	0	0	0	2	1	0	0	0	3292.101	0.000000	3384.295884
14	10	0	0	0	1	1	0	0	0	3307.546	0.303655	3437.235797
12	11	8	0	0	1	1	1	0	0	3318.288	0.000000	3393.051577
11	10	0	0	0	1	2	0	0	0	3339.318	0.000000	3425.520866
11	9	0	0	0	2	1	0	0	0	3342.311	0.000000	3441.052899
12	8	0	0	0	2	1	0	0	0	3351.783	0.000000	3446.811708
12	10	0	0	0	1	2	0	0	0	3400.527	0.000000	3479.280996
12	11	9	0	0	1	1	1	0	0	3406.977	0.000000	3494.813029
13	11	0	0	0	1	1	0	0	0	3444.592	0.072097	3532.132037
12	9	0	0	0	2	1	0	0	0	3447.725	0.000000	3548.573160
13	12	0	0	0	1	1	0	0	0	3485.791	0.268558	3585.892167
14	11	0	0	0	1	1	0	0	0	3522.995	0.018733	3642.688282
11	10	0	0	0	2	1	0	0	0	3538.935	0.000000	3630.973351
14	12	0	0	0	1	1	0	0	0	3561.353	0.208368	3696.448413
13	7	0	0	0	1	2	0	0	0	3597.319	0.000000	3691.640224
12	11	10	0	0	1	1	1	0	0	3605.571	0.000000	3684.733481
12	10	0	0	0	2	1	0	0	0	3648.288	0.000000	3738.493611
13	8	7	0	0	1	1	1	0	0	3661.290	0.000000	3754.156048
14	7	0	0	0	1	2	0	0	0	3681.312	0.000000	3802.196469
15	7	0	0	0	1	1	0	0	0	3718.957	0.049644	3872.346667
11	0	0	0	0	3	0	0	0	0	3720.903	0.000000	3836.425835
13	8	0	0	0	1	2	0	0	0	3724.854	0.000000	3816.671871
14	8	7	0	0	1	1	1	0	0	3745.156	0.000000	3864.712293
13	9	7	0	0	1	1	1	0	0	3768.384	0.000000	3855.917500
15	8	0	0	0	1	1	0	0	0	3781.833	0.003397	3934.862491
12	11	0	0	0	1	2	0	0	0	3792.965	0.000000	3890.185966
14	8	0	0	0	1	2	0	0	0	3808.592	0.000000	3927.228117
13	9	8	0	0	1	1	1	0	0	3831.945	0.000000	3918.433324
12	11	0	0	0	2	1	0	0	0	3841.110	0.000000	3943.946096
14	9	7	0	0	1	1	1	0	0	3842.987	0.000000	3966.473745
12	0	0	0	0	3	0	0	0	0	3865.336	0.000000	3997.706227
15	9	0	0	0	1	1	0	0	0	3876.260	0.304073	4036.623943
14	9	8	0	0	1	1	1	0	0	3906.420	0.000000	4028.989569
13	9	0	0	0	1	2	0	0	0	3936.294	0.000000	4020.194776
13	10	7	0	0	1	1	1	0	0	3952.238	0.000000	4045.837951
14	9	0	0	0	1	2	0	0	0	4001.507	0.000000	4130.751021
13	10	8	0	0	1	1	1	0	0	4010.475	0.000000	4108.353775
14	10	7	0	0	1	1	1	0	0	4012.904	0.000000	4156.394197

15	10	0	0	0	1	1	0	0	0	4061.234	0.067941	4226.544395
14	10	8	0	0	1	1	1	0	0	4071.014	0.000000	4218.910020
13	10	9	0	0	1	1	1	0	0	4120.105	0.000000	4210.115228
13	11	7	0	0	1	1	1	0	0	4144.888	0.000000	4251.290436
14	10	9	0	0	1	1	1	0	0	4171.381	0.000000	4320.671473
13	12	7	0	0	1	1	1	0	0	4190.753	0.000000	4305.050567
13	11	8	0	0	1	1	1	0	0	4210.818	0.000000	4313.806260
14	11	7	0	0	1	1	1	0	0	4224.213	0.000000	4361.846681
15	11	0	0	0	1	1	0	0	0	4243.472	0.571554	4431.996880
13	12	8	0	0	1	1	1	0	0	4249.089	0.000000	4367.566391
14	12	7	0	0	1	1	1	0	0	4267.238	0.000000	4415.606812
15	12	0	0	0	1	1	0	0	0	4288.071	0.054579	4485.757010
14	11	8	0	0	1	1	1	0	0	4290.016	0.000000	4424.362505
13	10	0	0	0	1	2	0	0	0	4292.178	0.000000	4400.035679
13	11	9	0	0	1	1	1	0	0	4309.964	0.000000	4415.567712
14	12	8	0	0	1	1	1	0	0	4325.447	0.000000	4478.122636
14	10	0	0	0	1	2	0	0	0	4329.517	0.000000	4510.591924
13	0	0	0	0	2	0	0	0	0	4354.831	0.032388	4506.646850
13	12	9	0	0	1	1	1	0	0	4355.489	0.000000	4469.327843
14	11	9	0	0	1	1	1	0	0	4379.900	0.000000	4526.123958
14	13	0	0	0	1	1	0	0	0	4404.561	0.274181	4617.203096
15	7	0	0	0	1	2	0	0	0	4420.511	0.000000	4591.505066
14	12	9	0	0	1	1	1	0	0	4422.584	0.000000	4579.884088
15	8	7	0	0	1	1	1	0	0	4486.491	0.000000	4654.020890
13	11	10	0	0	1	1	1	0	0	4498.033	0.000000	4605.488164
14	0	0	0	0	2	0	0	0	0	4509.745	0.195644	4727.759341
13	12	10	0	0	1	1	1	0	0	4545.527	0.000000	4659.248294
15	8	0	0	0	1	2	0	0	0	4552.065	0.000000	4716.536714
14	11	10	0	0	1	1	1	0	0	4554.032	0.000000	4716.044409
15	9	7	0	0	1	1	1	0	0	4580.630	0.000000	4755.782342
14	12	10	0	0	1	1	1	0	0	4598.685	0.000000	4769.804540
15	9	8	0	0	1	1	1	0	0	4646.200	0.000000	4818.298166
13	11	0	0	0	1	2	0	0	0	4686.239	0.000000	4810.940649
15	9	0	0	0	1	2	0	0	0	4737.594	0.000000	4920.059619
13	12	11	0	0	1	1	1	0	0	4739.160	0.000000	4864.700779
14	11	0	0	0	1	2	0	0	0	4760.898	0.000000	4921.496894
13	12	0	0	0	1	2	0	0	0	4768.163	0.000000	4918.460910
15	10	7	0	0	1	1	1	0	0	4768.164	0.000000	4945.702794
14	12	11	0	0	1	1	1	0	0	4810.978	0.000000	4975.257024
15	10	8	0	0	1	1	1	0	0	4828.411	0.000000	5008.218618
14	12	0	0	0	1	2	0	0	0	4837.140	0.000000	5029.017155
15	10	9	0	0	1	1	1	0	0	4925.085	0.000000	5109.980070
15	11	7	0	0	1	1	1	0	0	4946.262	0.000000	5151.155279
15	12	7	0	0	1	1	1	0	0	4995.528	0.000000	5204.915409
15	11	8	0	0	1	1	1	0	0	5014.202	0.000000	5213.671103
15	12	8	0	0	1	1	1	0	0	5055.873	0.000000	5267.431233
13	7	0	0	0	2	1	0	0	0	5060.589	0.000000	5225.805250
15	11	9	0	0	1	1	1	0	0	5100.393	0.000000	5315.432555
15	10	0	0	0	1	2	0	0	0	5100.838	0.000000	5299.900522
14	13	7	0	0	1	1	1	0	0	5111.242	0.000000	5336.361495
13	8	0	0	0	2	1	0	0	0	5117.578	0.000000	5288.321073
15	12	9	0	0	1	1	1	0	0	5149.317	0.000000	5369.192685
14	13	8	0	0	1	1	1	0	0	5168.104	0.000000	5398.877319
15	13	0	0	0	1	1	0	0	0	5202.934	0.041933	5406.511693
14	7	0	0	0	2	1	0	0	0	5217.348	0.000000	5446.917740
13	9	0	0	0	2	1	0	0	0	5234.436	0.000000	5390.082526
14	8	0	0	0	2	1	0	0	0	5274.083	0.000000	5509.433564
14	13	9	0	0	1	1	1	0	0	5275.699	0.000000	5500.638771
15	14	0	0	0	1	1	0	0	0	5286.511	0.047033	5517.067938
15	11	10	0	0	1	1	1	0	0	5292.142	0.000000	5505.353007
15	12	10	0	0	1	1	1	0	0	5343.036	0.000000	5559.113137
14	9	0	0	0	2	1	0	0	0	5372.415	0.000000	5611.195016
13	10	0	0	0	2	1	0	0	0	5413.949	0.000000	5580.002977
14	13	10	0	0	1	1	1	0	0	5441.275	0.000000	5690.559223
15	11	0	0	0	1	2	0	0	0	5465.797	0.000000	5710.805491
15	12	11	0	0	1	1	1	0	0	5522.118	0.000000	5764.565622
14	10	0	0	0	2	1	0	0	0	5524.055	0.000000	5801.115468
15	12	0	0	0	1	2	0	0	0	5554.521	0.000000	5818.325752
13	11	0	0	0	2	1	0	0	0	5608.394	0.000000	5785.455462
13	12	0	0	0	2	1	0	0	0	5642.173	0.000000	5839.215593
14	13	11	0	0	1	1	1	0	0	5654.379	0.000000	5896.011707
14	13	12	0	0	1	1	1	0	0	5685.318	0.000000	5949.771838
14	11	0	0	0	2	1	0	0	0	5755.819	0.000000	6006.567952
14	12	0	0	0	2	1	0	0	0	5783.917	0.000000	6060.328083
15	0	0	0	0	2	0	0	0	0	5898.437	0.515631	6306.376535
15	13	7	0	0	1	1	1	0	0	5911.187	0.000000	6125.670092
15	13	8	0	0	1	1	1	0	0	5970.185	0.000000	6188.185916
15	14	7	0	0	1	1	1	0	0	5995.686	0.000000	6236.226337

```
   15   14    8    0    0  1   1   1   0   0  6054.557    0.000000   6298.742161
   15   13    9    0    0  1   1   1   0   0  6074.088    0.000000   6289.947368
   15   14    9    0    0  1   1   1   0   0  6149.197    0.000000   6400.503614
   15   13   10    0    0  1   1   1   0   0  6257.281    0.000000   6479.867820
   15   14   10    0    0  1   1   1   0   0  6318.453    0.000000   6590.424065
   15   13   11    0    0  1   1   1   0   0  6437.174    0.000000   6685.320305
   15   13   12    0    0  1   1   1   0   0  6474.353    0.000000   6739.080435
   13    0    0    0    0  3   0   0   0   0  6487.366    0.000000   6759.970276
   14   13    0    0    0  1   2   0   0   0  6504.679    0.000000   6870.526521
   15   14   11    0    0  1   1   1   0   0  6517.006    0.000000   6795.876550
   15   14   12    0    0  1   1   1   0   0  6551.345    0.000000   6849.636680
   14   13    0    0    0  2   1   0   0   0  6577.445    0.000000   6981.082766
   15    7    0    0    0  2   1   0   0   0  6609.183    0.000000   7025.534935
   15    8    0    0    0  2   1   0   0   0  6670.192    0.000000   7088.050759
   14    0    0    0    0  3   0   0   0   0  6705.666    0.000000   7091.639011
   15    9    0    0    0  2   1   0   0   0  6761.138    0.000000   7189.812211
   15   10    0    0    0  2   1   0   0   0  6948.012    0.000000   7379.732662
   15   11    0    0    0  2   1   0   0   0  7113.354    0.000000   7585.185147
   15   12    0    0    0  2   1   0   0   0  7153.933    0.000000   7638.945278
   15   13    0    0    0  1   2   0   0   0  7365.370    0.000000   7659.835118
   15   14   13    0    0  1   1   1   0   0  7416.529    0.000000   7770.391363
   15   14    0    0    0  1   2   0   0   0  7523.141    0.000000   7880.947608
   15   13    0    0    0  2   1   0   0   0  8090.772    0.000000   8559.699961
   15   14    0    0    0  2   1   0   0   0  8175.778    0.000000   8670.256206
   15    0    0    0    0  3   0   0   0   0  8663.574    0.000000   9459.564803
 --------------------------------------------------------------------------------
  Dipole moment function written to files dipolex(y,z)
   @CHECKOUT-I, Total execution time :       0.0000 seconds.
  --executable xcubic finished with status           0
```

Table S.10. Part of the CFOUR output file of the CCSD(T)/VQZ-aV(Q+*d*)Z anharmonic frequency calculation for $^{12}CHD_2{}^{37}Cl$

```
   *************************************************************************
         <<<     CCCCCC     CCCCCC   |||     CCCCCC     CCCCCC   >>>
       <<<      CCC        CCC       |||    CCC        CCC         >>>
      <<<      CCC        CCC        |||   CCC        CCC            >>>
    <<<        CCC        CCC        |||   CCC        CCC              >>>
      <<<      CCC        CCC        |||   CCC        CCC            >>>
       <<<      CCC        CCC       |||    CCC        CCC         >>>
         <<<     CCCCCC     CCCCCC   |||     CCCCCC     CCCCCC   >>>
   *************************************************************************

     ****************************************************************
     * CFOUR Coupled-Cluster techniques for Computational Chemistry *
     ****************************************************************


   Department of Chemistry                Institut fuer Physikalische Chemie
   University of Texas at Austin          Universitaet Mainz
   Austin, TX  78712, USA                 D-55099 Mainz, Germany

                       Version 1.0


                                   Normal Coordinates

                A'                          A''                         A'
              712.38                      780.48                      880.71
             VIBRATION                   VIBRATION                   VIBRATION
 C       0.064 0.6681  0.0000    0.0000  0.0000  0.3721    0.4312 -0.3534  0.0000
 CL     -0.020-0.5529  0.0000    0.0000  0.0000 -0.1592   -0.1262  0.0912  0.0000
 H      -0.030 0.3433  0.0110   -0.0232  0.6376 -0.0776   -0.2631  0.3971  0.0388
 H      -0.030 0.3433 -0.0110    0.0232 -0.6376 -0.0776   -0.2631  0.3971 -0.0388
 H      -0.015 0.0726  0.0000    0.0000  0.0000 -0.1001    0.0204 -0.4556  0.0000

                A'                          A'                          A''
             1073.05                     1269.00                     1324.63
             VIBRATION                   VIBRATION                   VIBRATION
 C       0.268 0.2949  0.0000    0.0602 -0.2818  0.0000    0.0000  0.0000  0.2598
 CL      0.002 0.0069  0.0000    0.0470 -0.0369  0.0000    0.0000  0.0000  0.0041
 H      -0.338-0.3392  0.4306   -0.2636  0.1342  0.0652    0.3299 -0.1322 -0.0347
 H      -0.338-0.3392 -0.4306   -0.2636  0.1342 -0.0652   -0.3299  0.1322 -0.0347
 H       0.019-0.0999  0.0000    0.2530  0.8160  0.0000    0.0000  0.0000 -0.8231

                A'                          A''                         A'
             2251.67                     2361.61                     3202.70
             VIBRATION                   VIBRATION                   VIBRATION
 C       0.232 0.1873  0.0000    0.0000  0.0000  0.4338    0.2740 -0.0632  0.0000
 CL     -0.001-0.0035  0.0000    0.0000  0.0000 -0.0028   -0.0008 -0.0001  0.0000
 H      -0.312-0.2123 -0.5550   -0.2983 -0.2012 -0.5257   -0.0053 -0.0110 -0.0270
 H      -0.312-0.2123  0.5550    0.2983  0.2012 -0.5257   -0.0053 -0.0110  0.0270
 H       0.090-0.0243  0.0000    0.0000  0.0000  0.0068   -0.9257  0.2495  0.0000
             Gradient vector in normal coordinate representation
           ----------------------------------------------------------
   i    W(I)     dE/dQ(i)      dE/dq      dE/dq      [dE/dQ(i)]/w(i)
                              (cm-1)      (eV)         (relative)
           ----------------------------------------------------------
    7   712.38 -0.0000002243     -0.02024      0.00000   0.0000000000
    8   780.48  0.0000000000      0.00000      0.00000   0.0000000000
    9   880.71  0.0000006073      0.04928      0.00001   0.0000000000
   10  1073.05  0.0000005085      0.03738      0.00000   0.0000000000
   11  1269.00  0.0000022098      0.14939      0.00002   0.0000000000
   12  1324.63  0.0000000000      0.00000      0.00000   0.0000000000
   13  2251.67 -0.0000074472     -0.37795     -0.00005   0.0000000000
   14  2361.61  0.0000000000      0.00000      0.00000   0.0000000000
   15  3202.70 -0.0037340809   -158.89938     -0.01970   0.0000021628
           ----------------------------------------------------------

                      Normal modes in internal coordinates
--------------------------------------------------------------------------
            95.659        35.442          0.012          0.012         0.040
--------------------------------------------------------------------------
RCl        0.000000     -0.000464       0.000000       0.000000      0.000000
```

RH	0.000100	0.000014	0.000000	0.000000	0.000000
TDA1	-0.001496	-0.000239	0.000000	0.000000	0.000000
RH	-0.000100	0.000014	0.000000	0.000000	0.000000
TDA1	0.001496	-0.000239	0.000000	0.000000	0.000000
D120	0.000000	-0.000803	0.000000	0.000000	0.000000
RH	0.000000	0.000088	0.000000	0.000000	0.000000
TDA1	0.000000	0.002613	0.000000	0.000000	0.000000
D120	0.006660	0.000402	0.000000	0.000000	0.000000

	10.319	712.382	780.482	880.714	1073.050
RCl	0.000000	0.946571	0.000000	-0.233946	0.140440
RH	0.000006	0.014382	0.023445	0.013789	-0.008862
TDA1	0.000149	0.101322	0.643997	0.423453	-0.255796
RH	-0.000006	0.014382	-0.023445	0.013789	-0.008862
TDA1	-0.000149	0.101322	-0.643997	0.423453	-0.255796
D120	0.000000	0.145789	0.000000	0.572269	0.803497
RH	0.000000	-0.003131	0.000000	0.004422	0.008120
TDA1	0.000000	-0.237652	0.000000	-0.420571	-0.205382
D120	-0.000176	-0.072895	-0.411628	-0.286135	-0.401748

	1269.005	1324.628	2251.670	2361.613	3202.695
RCl	-0.144084	0.000000	0.069373	0.000000	-0.020478
RH	0.005459	-0.009414	-0.697931	-0.696878	-0.048256
TDA1	0.181138	-0.054266	-0.004770	0.067444	0.025773
RH	0.005459	0.009414	-0.697931	0.696878	-0.048256
TDA1	0.181138	0.054266	-0.004770	-0.067444	0.025773
D120	0.379643	0.000000	0.098945	0.000000	0.073789
RH	0.044090	0.000000	-0.058648	0.000000	0.992838
TDA1	0.855250	0.000000	-0.072459	0.000000	-0.032597
D120	-0.189821	-0.996962	-0.049472	-0.140084	-0.036894

Dipole Moment Function
(Normal Coordinate Basis)

Mode	Symmetry	d(Mu(x))/dQ	d(Mu(y))/dQ	d(Mu(z))/dQ
Q7	A'	-0.008141	0.130147	0.000000
Q8	A''	0.000000	0.000000	-0.017233
Q9	A'	-0.026646	-0.073911	0.000000
Q10	A'	-0.047407	0.064340	0.000000
Q11	A'	-0.008015	-0.081570	0.000000
Q12	A''	0.000000	0.000000	-0.076134
Q13	A'	0.021496	0.109721	0.000000
Q14	A''	0.000000	0.000000	0.052096
Q15	A'	0.056859	-0.076309	0.000000

Parameter	(MHz)	(CM-1)
R6	-.130982E-04	-.436909E-09
R5	-.116881E-01	-.389873E-06
SI	0.573566E+03	

A-reduced centrifugal distortion parameters

Parameter	(MHz)	(CM-1)
DJ	0.120161E-01	0.400814E-06
DK	0.934898E+00	0.311848E-04
DJK	0.124242E+00	0.414426E-05
DELJ	0.120423E-01	0.401687E-06
DELK	0.935029E+00	0.311892E-04
DELJK	0.124085E+00	0.413902E-05
delJ	0.287056E-03	0.957516E-08
delK	0.534269E-01	0.178213E-05

S-reduced centrifugal distortion parameters

Parameter	(MHz)	(CM-1)
DJ	0.119957E-01	0.400134E-06
DK	0.934796E+00	0.311814E-04
DJK	0.124364E+00	0.414834E-05
D1	-.287056E-03	-.957516E-08
D2	-.232872E-04	-.776777E-09

```
   Vibrational frequencies after rotational projection  of
   Cartesian force constants:
            1              0.0000i
            2              0.0000i
            3              0.0000i
            4              0.0000
            5              0.0000
            6              0.0000
            7            712.3812
            8            780.4613
            9            880.7088
           10           1073.0495
           11           1268.9923
           12           1324.5456
           13           2251.6699
           14           2361.6126
           15           3202.6950
  Zero-point vibrational energy:   19.8083 kcal/mol =   82.8780 kJ/mol.
               0.38 seconds walltime passed
 --executable xjoda finished with status            0
 --invoking executable xcubic
  @GETMEM-I,   Allocated   13351 MB of main memory.
  back



********************************************************************************
        PARAMETERS RELEVANT TO MOLECULAR STRUCTURE
********************************************************************************

  Force Field from Numerical Differentiation of Analytic Second Derivatives
  -----------------------------------------------------------------------------
 Cubic force constants written to file cubic.

---------------------------------------------------------------------------
  Mean and mean-square displacements in dimensionless normal coordinates

             Geometrical properties evaluated at     0.00 K

         Coordinate             <q>           <q**2>
---------------------------------------------------------------------------
              7             0.1321044667     0.4999999889
              8             0.0000000000     0.4999999889
              9            -0.0325827646     0.4999999889
             10             0.0058548311     0.4999999889
             11             0.0117484668     0.4999999889
             12             0.0000000000     0.4999999889
             13            -0.1289257363     0.4999999889
             14             0.0000000000     0.4999999889
             15             0.0784458787     0.4999999889
---------------------------------------------------------------------------

  ------------------------------------------------------
   ATOM              INTERNUCLEAR DISTANCE / Angstrom
  -------------------------------------------------------
  I    J           Re           Rg           Ra
  ------------------------------------------------------
  2    1        1.7826656    1.7908405    1.7905230
  3    1        1.0850773    1.1004844    1.0929669
  3    2        2.3617095    2.3756395    2.3738700
  4    1        1.0850773    1.1004844    1.0929669
  4    2        2.3617095    2.3756395    2.3738700
  4    3        1.7831251    1.8022880    1.7960060
  5    1        1.0850773    1.1062119    1.0942196
  5    2        2.3617095    2.3798556    2.3762750
  5    3        1.7831251    1.8062483    1.7970554
  5    4        1.7831251    1.8062483    1.7970554
  ------------------------------------------------------
********************************************************************************
     PARAMETERS RELEVANT TO ROTATIONAL SPECTROSCOPY
********************************************************************************


  ----------------------------------------------------------------------------
                                   VIB-ROT CONSTANT / (cm-1)
                                ------------------------------
    AXIS        MODE       CORIOLIS     QUADRATIC     ANHARMONIC    TOTAL
  ----------------------------------------------------------------------------
```

1	7	0.0005781	-0.0010753	0.0034441	0.0029469
1	8	0.0004244	-0.0000001	0.0007660	0.0011902
1	9	0.0000126	-0.0002156	0.0009607	0.0007576
1	10	0.0001089	-0.0000286	0.0001796	0.0002599
1	11	0.0057920	-0.0000722	0.0007530	0.0064729
1	12	-0.0060931	-0.0000097	-0.0000994	-0.0062021
1	13	-0.0002600	-0.0000264	0.0007028	0.0004165
1	14	-0.0004053	-0.0000046	0.0005239	0.0001140
1	15	-0.0000185	-0.0000039	-0.0000987	-0.0001210
2	7	0.0008124	-0.0000524	0.0033943	0.0041543
2	8	0.0248519	-0.0046983	-0.0185760	0.0015775
2	9	-0.0095056	-0.0035376	-0.0147321	-0.0277752
2	10	0.0237580	-0.0038669	-0.0057029	0.0141882
2	11	0.0403189	-0.0024479	-0.0033261	0.0345449
2	12	-0.0157676	-0.0001104	-0.0054424	-0.0213204
2	13	-0.0028686	-0.0198081	0.0525688	0.0298921
2	14	-0.0342101	-0.0001545	0.0504246	0.0160600
2	15	-0.0199155	-0.0029708	0.0383356	0.0154493
3	7	0.0002015	-0.0011337	0.0036063	0.0026741
3	8	0.0002804	-0.0005621	0.0009825	0.0007008
3	9	0.0015724	-0.0000282	0.0009613	0.0025055
3	10	-0.0011777	-0.0000169	0.0001295	-0.0010651
3	11	0.0000283	-0.0000005	0.0007154	0.0007433
3	12	-0.0001602	-0.0000232	0.0000097	-0.0001736
3	13	-0.0001817	-0.0000048	0.0002728	0.0000863
3	14	-0.0000928	-0.0000232	0.0003343	0.0002183
3	15	-0.0003402	-0.0000067	0.0002688	-0.0000781

Be, B0 AND B-B0 SHIFTS FOR SINGLY EXCITED VIBRATIONAL STATES (CM-1)

VIBRATION	X AXIS	Y AXIS	Z AXIS
Be	0.37398667	3.20837886	0.38377489
B0	0.37106931	3.17499356	0.38096914
Be-B0	0.00291737	0.03338530	0.00280576
B'	0.37106593	3.17499805	0.38097027
Be-B'	0.00292075	0.03338080	0.00280462
B''	0.37107242	3.17499885	0.38097355
Be-B''	0.00291425	0.03338001	0.00280135
B^A	0.37106389	3.17499804	0.38097231
Be-B^A	0.00292278	0.03338081	0.00280258
B^S	0.37106752	3.17499805	0.38096868
Be-B^S	0.00291915	0.03338080	0.00280621
7	-0.00294691	-0.00415432	-0.00267408
8	-0.00119022	-0.00157750	-0.00070081
9	-0.00075762	0.02777524	-0.00250551
10	-0.00025985	-0.01418821	0.00106506
11	-0.00647286	-0.03454485	-0.00074325
12	0.00620214	0.02132044	0.00017363
13	-0.00041647	-0.02989207	-0.00008631
14	-0.00011396	-0.01606001	-0.00021833
15	0.00012101	-0.01544933	0.00007809

Be, B0 AND B-B0 SHIFTS FOR SINGLY EXCITED VIBRATIONAL STATES (MHz)

VIBRATION	X AXIS	Y AXIS	Z AXIS
Be	11211.83844253	96184.77834540	11505.28190683
B0	11124.37790688	95183.91223521	11421.16745295
Be-B0	87.46053565	1000.86611019	84.11445388
B'	11124.27665565	95184.04700444	11421.20149975
Be-B'	87.56178688	1000.73134096	84.08040708
B''	11124.47135735	95184.07085256	11421.29955169
Be-B''	87.36708517	1000.70749284	83.98235514
B^A	11124.21569548	95184.04679310	11421.26267126
Be-B^A	87.62274704	1000.73155230	84.01923557
B^S	11124.32442335	95184.04702849	11421.15375508
Be-B^S	87.51401918	1000.73131691	84.12815175
7	-88.34618583	-124.54329642	-80.16682998
8	-35.68202622	-47.29236440	-21.00989520
9	-22.71279232	832.68088650	-75.11332186
10	-7.79023205	-425.35171364	31.92970464
11	-194.05143794	-1035.62869755	-22.28221090
12	185.93550006	639.17079973	5.20539649
13	-12.48532307	-896.14170414	-2.58754881

```
    14                  -3.41638743       -481.46686079         -6.54541333
    15                   3.62781349       -463.15926967          2.34121119
  ----------------------------------------------------------------------------
                 Vibrationally averaged dipole moment
  ------------------------------------------------------------------------
                  a.u.                            Debye
         x         y         z              x         y         z
  ------------------------------------------------------------------------
MU_e -0.03009   0.75412   0.00000       -0.07647   1.91666   0.00000
<MU> -0.02801   0.74857   0.00000       -0.07120   1.90256   0.00000

Equilibrium dipole moment:    0.75472 a.u. (   1.91818 D)
Equilibrium dipole moment:    0.74910 a.u. (   1.90389 D)
------------------------------------------------------------------------
********************************************************************************
    PARAMETERS RELEVANT TO VIBRATIONAL SPECTROSCOPY
********************************************************************************
  Quartic force constants written to file quartic
  ==============================================================
  Performing F(IIJJ)/F(JJII) consistency check
  Differences greater than 1 cm-1 will be printed.
  ==============================================================
  I    I    K    K       F(IIKK)       F(KKII)       Difference
  --------------------------------------------------------------
  --------------------------------------------------------------
  Largest absolute difference is    0.31976 cm-1.
  Largest relative difference is .11429D+00.
VPT2 vibrational analysis


 Thresholds for removing resonance denominators:
 Delta_omega = 50 cm-1; Phi(ijk) = 80 cm-1

-------------------------------
  ANHARMONICITY CONSTANTS X(ij)
                (cm-1)
-------------------------------
  (*) Near-zero denominators were removed
-------------------------------
          I  J          X(IJ)
-------------------------------
           7  7       -3.3481
           7  8       -3.6948
           7  9       -3.9650
           7 10       -1.4534
           7 11       -5.6018
           7 12       -0.9055
           7 13       -0.1031
           7 14        0.8213
           7 15        2.3412
           8  8       -0.5079
           8  9       -1.2732
           8 10       -4.0820
           8 11       -0.5338
           8 12       -3.4636
           8 13       -4.0065
           8 14       -3.1567
           8 15        0.4467
           9  9       -2.1431
           9 10       -1.8274
           9 11       -8.7271
           9 12       -4.4679
           9 13        5.4542
           9 14       -3.0450
           9 15       -3.0390
          10 10       -5.5267
          10 11       -1.9890
          10 12        4.3066
          10 13        3.6906
          10 14      -18.7134
          10 15       -1.0802
          11 11       -5.2879
          11 12        1.1453
          11 13        1.3074
          11 14       -2.3990
          11 15      -17.9713
          12 12       -5.5249
          12 13       -6.0738
```

12	14	-12.6646
12	15	-21.9955
13	13	-14.9604
13	14	-62.3383
13	15	-0.0810
14	14	-19.6513
14	15	1.3678
15	15	-61.3604

HARMONIC AND FUNDAMENTAL FREQUENCIES (cm-1) AND INTENSITIES (km/mol)

Mode	Harmonic Frequency	Fundamental Frequency	Anharmonic Contribution	Harmonic Intensity	Fundamental Intensity	Anharm Contrib
7	712.9446	699.9679	-12.9767	16.6408	17.6160	0.9751
8	781.1097	770.2120	-10.8977	0.2735	0.2760	0.0025
9	882.8539	868.1225	-14.7314	6.1520	5.9986	-0.1534
10	1073.3548	1051.7272	-21.6276	6.0453	5.6577	-0.3875
11	1278.6809	1250.7205	-27.9604	6.5254	5.8545	-0.6709
12	1332.5682	1299.4589	-33.1093	5.7335	5.8010	0.0675
13	2253.3224	2192.3264	-60.9961	12.1868	11.2170	-0.9698
14	2363.8791	2274.5125	-89.3666	2.5973	2.5674	-0.0299
15	3153.1882	3010.4616	-142.7266	9.2611	10.2584	0.9974

ZERO-POINT VIBRATIONAL ENERGIES

---

	kcal/mol	kJ/mol	Hartree	cm-1
Harmonic contribution :	19.7737	82.7330	0.03151139	6915.951
VPT2-correction :	-0.2334	-0.9764	-0.00037189	-81.620
Harm+VPT2 :	19.5403	81.7567	0.03113950	6834.331

MAXLEVEL set to 3

All levels with up to three quanta

MODE I	MODE J	MODE K	MODE L	MODE M	NI	NJ	NK	NL	NM	Anharmonic Frequency	Anharm Intensity	Harmonic Transition
7	0	0	0	0	1	0	0	0	0	699.967	17.615627	712.944633
8	0	0	0	0	1	0	0	0	0	770.211	0.275968	781.109677
9	0	0	0	0	1	0	0	0	0	868.122	5.998515	882.853857
10	0	0	0	0	1	0	0	0	0	1051.727	5.657621	1073.354769
11	0	0	0	0	1	0	0	0	0	1250.720	5.854368	1278.680931
12	0	0	0	0	1	0	0	0	0	1299.458	5.800851	1332.568230
7	0	0	0	0	2	0	0	0	0	1393.239	0.136363	1425.889266
8	7	0	0	0	1	1	0	0	0	1466.485	0.012819	1494.054310
8	0	0	0	0	2	0	0	0	0	1539.408	0.001635	1562.219355
9	7	0	0	0	1	1	0	0	0	1564.125	0.002722	1595.798490
9	8	0	0	0	1	1	0	0	0	1637.061	0.020584	1663.963534
9	0	0	0	0	2	0	0	0	0	1731.958	0.049948	1765.707714
10	7	0	0	0	1	1	0	0	0	1750.241	0.041943	1786.299402
10	8	0	0	0	1	1	0	0	0	1817.857	0.065377	1854.464446
10	9	0	0	0	1	1	0	0	0	1918.022	0.043487	1956.208626
11	7	0	0	0	1	1	0	0	0	1945.086	0.063661	1991.625564
12	7	0	0	0	1	1	0	0	0	1998.521	0.002750	2045.512863
11	8	0	0	0	1	1	0	0	0	2020.398	0.004672	2059.790609
12	8	0	0	0	1	1	0	0	0	2066.207	0.082832	2113.677908
7	0	0	0	0	3	0	0	0	0	2079.815	0.000000	2138.833899
10	0	0	0	0	2	0	0	0	0	2092.400	0.795952	2146.709538
11	9	0	0	0	1	1	0	0	0	2110.115	0.602942	2161.534788
8	7	0	0	0	1	2	0	0	0	2156.062	0.000000	2206.998943
12	9	0	0	0	1	1	0	0	0	2163.113	0.000308	2215.422087
13	0	0	0	0	1	0	0	0	0	2192.326	11.216781	2253.322441
8	7	0	0	0	2	1	0	0	0	2231.986	0.000000	2275.163987
9	7	0	0	0	1	2	0	0	0	2253.432	0.000000	2308.743123
14	0	0	0	0	1	0	0	0	0	2274.512	2.567300	2363.879130
11	10	0	0	0	1	1	0	0	0	2300.458	0.218491	2352.035700
8	0	0	0	0	3	0	0	0	0	2307.588	0.000000	2343.329032
9	8	7	0	0	1	1	1	0	0	2329.369	0.000000	2376.908167
12	10	0	0	0	1	1	0	0	0	2355.492	0.813067	2405.922999
9	8	0	0	0	1	2	0	0	0	2404.984	0.000000	2445.073212
9	7	0	0	0	2	1	0	0	0	2423.996	0.000000	2478.652347
10	7	0	0	0	1	2	0	0	0	2442.060	0.000000	2499.244035
11	0	0	0	0	2	0	0	0	0	2490.865	0.153707	2557.361863

9	8	0	0	0	2	1	0	0	0	2499.624	0.000000	2546.817391
10	8	7	0	0	1	1	1	0	0	2512.676	0.000000	2567.409079
12	11	0	0	0	1	1	0	0	0	2551.324	0.047762	2611.249162
10	8	0	0	0	1	2	0	0	0	2582.971	0.000000	2635.574123
12	0	0	0	0	2	0	0	0	0	2587.868	0.151044	2665.136461
9	0	0	0	0	3	0	0	0	0	2591.508	0.000000	2648.561571
10	9	7	0	0	1	1	1	0	0	2612.571	0.000000	2669.153259
11	7	0	0	0	1	2	0	0	0	2632.756	0.000000	2704.570197
10	9	8	0	0	1	1	1	0	0	2682.879	0.000000	2737.318303
12	7	0	0	0	1	2	0	0	0	2690.887	0.000000	2758.457496
11	8	7	0	0	1	1	1	0	0	2711.069	0.000000	2772.735242
12	8	7	0	0	1	1	1	0	0	2761.574	0.000000	2826.622541
10	9	0	0	0	1	2	0	0	0	2780.031	0.000000	2839.062483
11	8	0	0	0	1	2	0	0	0	2789.061	0.000000	2840.900286
10	7	0	0	0	2	1	0	0	0	2789.462	0.000000	2859.654171
11	9	7	0	0	1	1	1	0	0	2800.517	0.000000	2874.479421
12	8	0	0	0	1	2	0	0	0	2831.939	0.000000	2894.787585
10	8	0	0	0	2	1	0	0	0	2854.448	0.000000	2927.819215
12	9	7	0	0	1	1	1	0	0	2858.211	0.000000	2928.366720
11	9	8	0	0	1	1	1	0	0	2878.520	0.000000	2942.644466
13	7	0	0	0	1	1	0	0	0	2892.191	0.091639	2966.267073
12	9	8	0	0	1	1	1	0	0	2928.588	0.000000	2996.531765
10	9	0	0	0	2	1	0	0	0	2956.868	0.000000	3029.563395
13	8	0	0	0	1	1	0	0	0	2958.531	0.003002	3034.432118
11	9	0	0	0	1	2	0	0	0	2965.225	0.000000	3044.388646
14	7	0	0	0	1	1	0	0	0	2975.301	0.016703	3076.823763
11	10	7	0	0	1	1	1	0	0	2993.371	0.000000	3064.980333
15	0	0	0	0	1	0	0	0	0	3010.461	10.258234	3153.188205
12	9	0	0	0	1	2	0	0	0	3022.482	0.000000	3098.275944
14	8	0	0	0	1	1	0	0	0	3041.567	0.453122	3144.988807
12	10	7	0	0	1	1	1	0	0	3053.101	0.000000	3118.867632
13	9	0	0	0	1	1	0	0	0	3065.903	0.682443	3136.176298
11	10	8	0	0	1	1	1	0	0	3066.054	0.000000	3133.145378
12	10	8	0	0	1	1	1	0	0	3118.159	0.000000	3187.032677
10	0	0	0	0	3	0	0	0	0	3122.021	0.000000	3220.064307
14	9	0	0	0	1	1	0	0	0	3139.589	0.011129	3246.732987
11	10	9	0	0	1	1	1	0	0	3158.026	0.000000	3234.889557
11	7	0	0	0	2	1	0	0	0	3179.629	0.000000	3270.306496
12	10	9	0	0	1	1	1	0	0	3217.320	0.000000	3288.776856
12	11	7	0	0	1	1	1	0	0	3244.785	0.000000	3324.193795
13	10	0	0	0	1	1	0	0	0	3247.744	0.057780	3326.677209
11	8	0	0	0	2	1	0	0	0	3260.009	0.000000	3338.471540
12	7	0	0	0	2	1	0	0	0	3286.025	0.000000	3378.081094
14	10	0	0	0	1	1	0	0	0	3307.526	0.303881	3437.233899
12	11	8	0	0	1	1	1	0	0	3317.539	0.000000	3392.358839
11	10	0	0	0	1	2	0	0	0	3339.143	0.000000	3425.390469
11	9	0	0	0	2	1	0	0	0	3341.533	0.000000	3440.215720
12	8	0	0	0	2	1	0	0	0	3351.152	0.000000	3446.246138
12	10	0	0	0	1	2	0	0	0	3400.473	0.000000	3479.277768
12	11	9	0	0	1	1	1	0	0	3406.252	0.000000	3494.103019
13	11	0	0	0	1	1	0	0	0	3444.354	0.072345	3532.003372
12	9	0	0	0	2	1	0	0	0	3447.054	0.000000	3547.990318
13	12	0	0	0	1	1	0	0	0	3485.711	0.268554	3585.890671
14	11	0	0	0	1	1	0	0	0	3522.834	0.018815	3642.560061
11	10	0	0	0	2	1	0	0	0	3538.614	0.000000	3630.716632
14	12	0	0	0	1	1	0	0	0	3561.306	0.208402	3696.447360
13	7	0	0	0	1	2	0	0	0	3585.359	0.000000	3679.211706
12	11	10	0	0	1	1	1	0	0	3605.369	0.000000	3684.603931
12	10	0	0	0	2	1	0	0	0	3648.208	0.000000	3738.491230
13	8	7	0	0	1	1	1	0	0	3654.701	0.000000	3747.376751
14	7	0	0	0	1	2	0	0	0	3669.394	0.000000	3789.768396
15	7	0	0	0	1	1	0	0	0	3712.770	0.050255	3866.132837
11	0	0	0	0	3	0	0	0	0	3720.434	0.000000	3836.042794
13	8	0	0	0	1	2	0	0	0	3723.721	0.000000	3815.541795
14	8	7	0	0	1	1	1	0	0	3738.662	0.000000	3857.933440
13	9	7	0	0	1	1	1	0	0	3761.802	0.000000	3849.120930
15	8	0	0	0	1	1	0	0	0	3781.120	0.003430	3934.297882
12	11	0	0	0	1	2	0	0	0	3792.614	0.000000	3889.930093
14	8	0	0	0	1	2	0	0	0	3807.607	0.000000	3926.098484
13	9	8	0	0	1	1	1	0	0	3830.835	0.000000	3917.285975
14	9	7	0	0	1	1	1	0	0	3836.414	0.000000	3959.677620
12	11	0	0	0	2	1	0	0	0	3840.879	0.000000	3943.817392
12	0	0	0	0	3	0	0	0	0	3865.227	0.000000	3997.704691
15	9	0	0	0	1	1	0	0	0	3875.545	0.301901	4036.042062
14	9	8	0	0	1	1	1	0	0	3905.372	0.000000	4027.842664
13	9	0	0	0	1	2	0	0	0	3935.193	0.000000	4019.030155
13	10	7	0	0	1	1	1	0	0	3946.155	0.000000	4039.621842
14	9	0	0	0	1	2	0	0	0	4000.381	0.000000	4129.586844

14	10	7	0	0	1	1	1	0	0	4006.862	0.000000	4150.178532
13	10	8	0	0	1	1	1	0	0	4009.867	0.000000	4107.786887
15	10	0	0	0	1	1	0	0	0	4061.108	0.067870	4226.542973
14	10	8	0	0	1	1	1	0	0	4070.499	0.000000	4218.343576
13	10	9	0	0	1	1	1	0	0	4119.493	0.000000	4209.531066
13	11	7	0	0	1	1	1	0	0	4138.617	0.000000	4244.948005
14	10	9	0	0	1	1	1	0	0	4170.776	0.000000	4320.087756
13	12	7	0	0	1	1	1	0	0	4184.670	0.000000	4298.835304
13	11	8	0	0	1	1	1	0	0	4210.025	0.000000	4313.113049
14	11	7	0	0	1	1	1	0	0	4218.021	0.000000	4355.504694
15	11	0	0	0	1	1	0	0	0	4243.210	0.572439	4431.869136
13	12	8	0	0	1	1	1	0	0	4248.453	0.000000	4367.000348
14	12	7	0	0	1	1	1	0	0	4261.190	0.000000	4409.391993
15	12	0	0	0	1	1	0	0	0	4287.925	0.054615	4485.756435
14	11	8	0	0	1	1	1	0	0	4289.355	0.000000	4423.669739
13	10	0	0	0	1	2	0	0	0	4292.108	0.000000	4400.031978
13	11	9	0	0	1	1	1	0	0	4309.203	0.000000	4414.857229
14	12	8	0	0	1	1	1	0	0	4324.898	0.000000	4477.557038
14	10	0	0	0	1	2	0	0	0	4329.486	0.000000	4510.588668
13	0	0	0	0	2	0	0	0	0	4354.731	0.032386	4506.644881
13	12	9	0	0	1	1	1	0	0	4354.820	0.000000	4468.744528
14	11	9	0	0	1	1	1	0	0	4379.184	0.000000	4525.413918
14	13	0	0	0	1	1	0	0	0	4404.500	0.274182	4617.201570
15	7	0	0	0	1	2	0	0	0	4408.383	0.000000	4579.077470
14	12	9	0	0	1	1	1	0	0	4421.916	0.000000	4579.301217
15	8	7	0	0	1	1	1	0	0	4479.734	0.000000	4647.242515
13	11	10	0	0	1	1	1	0	0	4497.782	0.000000	4605.358141
14	0	0	0	0	2	0	0	0	0	4509.722	0.195675	4727.758260
13	12	10	0	0	1	1	1	0	0	4545.435	0.000000	4659.245440
15	8	0	0	0	1	2	0	0	0	4550.763	0.000000	4715.407559
14	11	10	0	0	1	1	1	0	0	4553.858	0.000000	4715.914830
15	9	7	0	0	1	1	1	0	0	4573.889	0.000000	4748.986694
14	12	10	0	0	1	1	1	0	0	4598.627	0.000000	4769.802129
15	9	8	0	0	1	1	1	0	0	4644.930	0.000000	4817.151739
13	11	0	0	0	1	2	0	0	0	4685.806	0.000000	4810.684303
15	9	0	0	0	1	2	0	0	0	4736.342	0.000000	4918.895919
13	12	11	0	0	1	1	1	0	0	4738.884	0.000000	4864.571602
14	11	0	0	0	1	2	0	0	0	4760.579	0.000000	4921.240993
15	10	7	0	0	1	1	1	0	0	4761.964	0.000000	4939.487606
13	12	0	0	0	1	2	0	0	0	4768.046	0.000000	4918.458901
14	12	11	0	0	1	1	1	0	0	4810.773	0.000000	4975.128292
15	10	8	0	0	1	1	1	0	0	4827.685	0.000000	5007.652651
14	12	0	0	0	1	2	0	0	0	4837.051	0.000000	5029.015591
15	10	9	0	0	1	1	1	0	0	4924.364	0.000000	5109.396831
15	11	7	0	0	1	1	1	0	0	4939.918	0.000000	5144.813769
15	12	7	0	0	1	1	1	0	0	4989.328	0.000000	5198.701068
15	11	8	0	0	1	1	1	0	0	5013.335	0.000000	5212.978813
13	7	0	0	0	2	1	0	0	0	5054.493	0.000000	5219.589514
15	12	8	0	0	1	1	1	0	0	5055.120	0.000000	5266.866112
15	11	9	0	0	1	1	1	0	0	5099.567	0.000000	5314.722993
15	10	0	0	0	1	2	0	0	0	5100.702	0.000000	5299.897742
14	13	7	0	0	1	1	1	0	0	5105.186	0.000000	5330.146203
13	8	0	0	0	2	1	0	0	0	5116.930	0.000000	5287.754558
15	12	9	0	0	1	1	1	0	0	5148.540	0.000000	5368.610292
14	13	8	0	0	1	1	1	0	0	5167.549	0.000000	5398.311248
15	13	0	0	0	1	1	0	0	0	5202.706	0.041943	5406.510645
14	7	0	0	0	2	1	0	0	0	5211.332	0.000000	5440.702893
13	9	0	0	0	2	1	0	0	0	5233.762	0.000000	5389.498738
14	8	0	0	0	2	1	0	0	0	5273.620	0.000000	5508.867937
14	13	9	0	0	1	1	1	0	0	5275.032	0.000000	5500.055428
15	14	0	0	0	1	1	0	0	0	5286.341	0.047032	5517.067335
15	11	10	0	0	1	1	1	0	0	5291.868	0.000000	5505.223905
15	12	10	0	0	1	1	1	0	0	5342.878	0.000000	5559.111204
14	9	0	0	0	2	1	0	0	0	5371.754	0.000000	5610.612117
13	10	0	0	0	2	1	0	0	0	5413.840	0.000000	5579.999650
14	13	10	0	0	1	1	1	0	0	5441.204	0.000000	5690.556339
15	11	0	0	0	1	2	0	0	0	5465.384	0.000000	5710.550067
15	12	11	0	0	1	1	1	0	0	5521.819	0.000000	5764.437366
14	10	0	0	0	2	1	0	0	0	5524.022	0.000000	5801.113029
15	12	0	0	0	1	2	0	0	0	5554.338	0.000000	5818.324665
13	11	0	0	0	2	1	0	0	0	5608.067	0.000000	5785.325813
13	12	0	0	0	2	1	0	0	0	5642.043	0.000000	5839.213111
14	13	11	0	0	1	1	1	0	0	5654.129	0.000000	5895.882502
14	13	12	0	0	1	1	1	0	0	5685.221	0.000000	5949.769801
14	11	0	0	0	2	1	0	0	0	5755.644	0.000000	6006.439191
14	12	0	0	0	2	1	0	0	0	5783.852	0.000000	6060.326490
15	0	0	0	0	2	0	0	0	0	5898.202	0.515636	6306.376409
15	13	7	0	0	1	1	1	0	0	5904.912	0.000000	6119.455278

```
   15   13    8    0    0  1   1   1   0   0  5969.359    0.000000   6187.620322
   15   14    7    0    0  1   1   1   0   0  5989.472    0.000000   6230.011967
   15   14    8    0    0  1   1   1   0   0  6053.843    0.000000   6298.177012
   15   13    9    0    0  1   1   1   0   0  6073.244    0.000000   6289.364502
   15   14    9    0    0  1   1   1   0   0  6148.380    0.000000   6399.921192
   15   13   10    0    0  1   1   1   0   0  6257.044    0.000000   6479.865414
   15   14   10    0    0  1   1   1   0   0  6318.275    0.000000   6590.422103
   15   13   11    0    0  1   1   1   0   0  6436.763    0.000000   6685.191577
   15   13   12    0    0  1   1   1   0   0  6474.096    0.000000   6739.078876
   13    0    0    0    0  3   0   0   0   0  6487.216    0.000000   6759.967322
   14   13    0    0    0  1   2   0   0   0  6504.567    0.000000   6870.524011
   15   14   11    0    0  1   1   1   0   0  6516.692    0.000000   6795.748266
   15   14   12    0    0  1   1   1   0   0  6551.140    0.000000   6849.635565
   14   13    0    0    0  2   1   0   0   0  6577.372    0.000000   6981.080700
   15    7    0    0    0  2   1   0   0   0  6602.852    0.000000   7019.321042
   15    8    0    0    0  2   1   0   0   0  6669.307    0.000000   7087.486086
   14    0    0    0    0  3   0   0   0   0  6705.629    0.000000   7091.637390
   15    9    0    0    0  2   1   0   0   0  6760.246    0.000000   7189.230266
   15   10    0    0    0  2   1   0   0   0  6947.769    0.000000   7379.731178
   15   11    0    0    0  2   1   0   0   0  7112.980    0.000000   7585.057341
   15   12    0    0    0  2   1   0   0   0  7153.670    0.000000   7638.944640
   15   13    0    0    0  1   2   0   0   0  7365.031    0.000000   7659.833086
   15   14   13    0    0  1   1   1   0   0  7416.249    0.000000   7770.389775
   15   14    0    0    0  1   2   0   0   0  7522.919    0.000000   7880.946464
   15   13    0    0    0  2   1   0   0   0  8090.366    0.000000   8559.698850
   15   14    0    0    0  2   1   0   0   0  8175.450    0.000000   8670.255539
   15    0    0    0    0  3   0   0   0   0  8663.222    0.000000   9459.564614
-------------------------------------------------------------------------------
 Dipole moment function written to files dipolex(y,z)
  @CHECKOUT-I, Total execution time :       0.0000 seconds.
 --executable xcubic finished with status            0
```

Table S.11. Part of the CFOUR output file of the CCSD(T)/VQZ-aV(Q+*d*)Z anharmonic frequency calculation for $^{13}CHD_2{}^{35}Cl$

```
   ***************************************************************************
         <<<     CCCCCC     CCCCCC   |||     CCCCCC     CCCCCC   >>>
       <<<      CCC        CCC       |||    CCC        CCC         >>>
      <<<      CCC        CCC        |||   CCC        CCC            >>>
     <<<       CCC        CCC        |||   CCC        CCC              >>>
      <<<      CCC        CCC        |||   CCC        CCC            >>>
       <<<      CCC        CCC       |||    CCC        CCC         >>>
         <<<     CCCCCC     CCCCCC   |||     CCCCCC     CCCCCC   >>>
   ***************************************************************************

     ****************************************************************
     * CFOUR Coupled-Cluster techniques for Computational Chemistry *
     ****************************************************************


   Department of Chemistry                Institut fuer Physikalische Chemie
   University of Texas at Austin          Universitaet Mainz
   Austin, TX  78712, USA                 D-55099 Mainz, Germany

                       Version 1.0



                                   Normal Coordinates

               A'                         A''                         A'
             705.66                      776.73                     870.68
           VIBRATION                   VIBRATION                  VIBRATION
 C       0.055 0.6785  0.0000    0.0000  0.0000  0.3681    0.4259 -0.3309  0.0000
 CL     -0.017-0.5762  0.0000    0.0000  0.0000 -0.1645   -0.1307  0.0791  0.0000
 H      -0.027 0.3140  0.0144   -0.0226  0.6362 -0.0859   -0.2736  0.4114  0.0401
 H      -0.027 0.3140 -0.0144    0.0226 -0.6362 -0.0859   -0.2736  0.4114 -0.0401
 H      -0.018 0.0686  0.0000    0.0000  0.0000 -0.1102    0.0136 -0.4406  0.0000

               A'                         A'                          A''
             1066.44                    1265.24                    1321.15
           VIBRATION                   VIBRATION                  VIBRATION
 C       0.260 0.2828  0.0000    0.0572 -0.2612  0.0000    0.0000  0.0000  0.2542
 CL      0.002 0.0153  0.0000    0.0494 -0.0428  0.0000    0.0000  0.0000  0.0048
 H      -0.339-0.3510  0.4251   -0.2648  0.1303  0.0727    0.3267 -0.1383 -0.0412
 H      -0.339-0.3510 -0.4251   -0.2648  0.1303 -0.0727   -0.3267  0.1383 -0.0412
 H       0.011-0.1139  0.0000    0.2518  0.8222  0.0000    0.0000  0.0000 -0.8248

               A'                         A''                         A'
             2244.05                    2344.63                    3192.88
           VIBRATION                   VIBRATION                  VIBRATION
 C       0.226 0.1779  0.0000    0.0000  0.0000  0.4182    0.2625 -0.0617  0.0000
 CL     -0.001-0.0031  0.0000    0.0000  0.0000 -0.0027   -0.0007 -0.0002  0.0000
 H      -0.313-0.2121 -0.5582   -0.3022 -0.2026 -0.5293   -0.0036 -0.0104 -0.0252
 H      -0.313-0.2121  0.5582    0.3022  0.2026 -0.5293   -0.0036 -0.0104  0.0252
 H       0.083-0.0210  0.0000    0.0000  0.0000  0.0104   -0.9286  0.2520  0.0000

 ...
 ...

  ----------------------------------------------------------------
                  Dipole Moment Function
                 (Normal Coordinate Basis)
  ----------------------------------------------------------------
    Mode     Symmetry    d(Mu(x))/dQ   d(Mu(y))/dQ   d(Mu(z))/dQ
  ----------------------------------------------------------------
     Q7         A'        -0.007921      0.132328      0.000000
     Q8        A''         0.000000      0.000000     -0.018180
     Q9         A'        -0.027896     -0.068388      0.000000
     Q10        A'        -0.047313      0.061442      0.000000
     Q11        A'        -0.008836     -0.076060      0.000000
     Q12       A''         0.000000      0.000000     -0.076288
     Q13        A'         0.021832      0.106563      0.000000
     Q14       A''         0.000000      0.000000      0.051678
     Q15        A'         0.056164     -0.075997      0.000000
  ----------------------------------------------------------------
---------------------------------------------------------------------------
------------------------------------------------------------
```

```
       Parameter                (MHz)                 (CM-1)
------------------------------------------------------------

          R6               -.126541E-04           -.422094E-09
          R5               -.114311E-01           -.381301E-06
          SI                           0.591847E+03

  A-reduced centrifugal distortion parameters

          DJ               0.117713E-01           0.392650E-06
          DK               0.934015E+00           0.311554E-04
          DJK              0.123706E+00           0.412638E-05
          DELJ             0.117967E-01           0.393494E-06
          DELK             0.934141E+00           0.311596E-04
          DELJK            0.123554E+00           0.412132E-05
          delJ             0.275559E-03           0.919165E-08
          delK             0.528193E-01           0.176186E-05

  S-reduced centrifugal distortion parameters

          DJ               0.117520E-01           0.392005E-06
          DK               0.933918E+00           0.311522E-04
          DJK              0.123822E+00           0.413025E-05
          D1               -.275559E-03           -.919165E-08
          D2               -.223112E-04           -.744222E-09
------------------------------------------------------------
   Vibrational frequencies after rotational projection  of
   Cartesian force constants:
           1              0.0000i
           2              0.0000i
           3              0.0000i
           4              0.0000
           5              0.0000
           6              0.0000
           7            705.6551
           8            776.7056
           9            870.6713
          10           1066.4358
          11           1265.2273
          12           1321.0638
          13           2244.0510
          14           2344.6326
          15           3192.8838
  Zero-point vibrational energy:   19.7099 kcal/mol =   82.4665 kJ/mol.
             0.12 seconds walltime passed
 --executable xjoda finished with status            0
 --invoking executable xcubic
  @GETMEM-I,   Allocated   13351 MB of main memory.
  back



********************************************************************************
        PARAMETERS RELEVANT TO MOLECULAR STRUCTURE
********************************************************************************

  Force Field from Numerical Differentiation of Analytic Second Derivatives
  ----------------------------------------------------------------------------
 Cubic force constants written to file cubic.

---------------------------------------------------------------------------
  Mean and mean-square displacements in dimensionless normal coordinates

             Geometrical properties evaluated at     0.00 K

         Coordinate             <q>           <q**2>
---------------------------------------------------------------------------
             7             0.1311925788     0.4999999889
             8             0.0000000000     0.4999999889
             9            -0.0278008593     0.4999999889
            10             0.0025194350     0.4999999889
            11             0.0141452783     0.4999999889
            12             0.0000000000     0.4999999889
            13            -0.1280562746     0.4999999889
            14             0.0000000000     0.4999999889
            15             0.0788742042     0.4999999889
---------------------------------------------------------------------------
```

ATOM		INTERNUCLEAR DISTANCE / Angstrom		
I	J	Re	Rg	Ra
2	1	1.7826656	1.7907040	1.7904117
3	1	1.0850773	1.1004048	1.0929183
3	2	2.3617095	2.3755677	2.3737726
4	1	1.0850773	1.1004048	1.0929183
4	2	2.3617095	2.3755677	2.3737726
4	3	1.7831251	1.8022035	1.7959046
5	1	1.0850773	1.1061524	1.0941738
5	2	2.3617095	2.3797714	2.3761631
5	3	1.7831251	1.8061640	1.7969527
5	4	1.7831251	1.8061640	1.7969527

****
PARAMETERS RELEVANT TO ROTATIONAL SPECTROSCOPY
****

AXIS	MODE	VIB-ROT CONSTANT / (cm-1) CORIOLIS	QUADRATIC	ANHARMONIC	TOTAL
1	7	0.0005478	-0.0010600	0.0034618	0.0029495
1	8	0.0004307	-0.0000001	0.0007531	0.0011836
1	9	0.0000013	-0.0002077	0.0008831	0.0006768
1	10	0.0001417	-0.0000256	0.0001792	0.0002952
1	11	0.0055757	-0.0000742	0.0007529	0.0062543
1	12	-0.0058904	-0.0000092	-0.0000902	-0.0059898
1	13	-0.0002602	-0.0000259	0.0006545	0.0003684
1	14	-0.0003913	-0.0000046	0.0004748	0.0000790
1	15	-0.0000182	-0.0000038	-0.0001243	-0.0001463
2	7	0.0006369	-0.0000356	0.0037281	0.0043294
2	8	0.0272787	-0.0046298	-0.0187689	0.0038800
2	9	-0.0112541	-0.0035610	-0.0152172	-0.0300323
2	10	0.0225705	-0.0037830	-0.0057242	0.0130634
2	11	0.0408410	-0.0023134	-0.0032037	0.0353239
2	12	-0.0150175	-0.0001197	-0.0052745	-0.0204116
2	13	-0.0034698	-0.0198774	0.0522981	0.0289510
2	14	-0.0342978	-0.0001554	0.0503647	0.0159115
2	15	-0.0197529	-0.0030404	0.0379118	0.0151184
3	7	0.0002014	-0.0011185	0.0036226	0.0027055
3	8	0.0002706	-0.0005468	0.0009650	0.0006887
3	9	0.0015110	-0.0000197	0.0008783	0.0023697
3	10	-0.0011370	-0.0000197	0.0001291	-0.0010277
3	11	0.0000314	-0.0000007	0.0007165	0.0007473
3	12	-0.0001499	-0.0000235	0.0000179	-0.0001556
3	13	-0.0001745	-0.0000046	0.0002407	0.0000616
3	14	-0.0000936	-0.0000230	0.0002867	0.0001701
3	15	-0.0003317	-0.0000066	0.0002295	-0.0001088

Be, B0 AND B-B0 SHIFTS FOR SINGLY EXCITED VIBRATIONAL STATES (CM-1)

VIBRATION	X AXIS	Y AXIS	Z AXIS
Be	0.36907372	3.20583433	0.37856773
B0	0.36623839	3.17276754	0.37584231
Be-B0	0.00283533	0.03306679	0.00272542
B'	0.36623510	3.17277194	0.37584341
Be-B'	0.00283863	0.03306238	0.00272432
B''	0.36624152	3.17277272	0.37584668
Be-B''	0.00283220	0.03306160	0.00272105
B^A	0.36623307	3.17277194	0.37584544
Be-B^A	0.00284065	0.03306239	0.00272229
B^S	0.36623666	3.17277194	0.37584185
Be-B^S	0.00283707	0.03306238	0.00272588
7	-0.00294953	-0.00432938	-0.00270552
8	-0.00118362	-0.00387996	-0.00068871
9	-0.00067676	0.03003230	-0.00236969
10	-0.00029521	-0.01306338	0.00102769
11	-0.00625435	-0.03532386	-0.00074725
12	0.00598978	0.02041159	0.00015557

```
    13                  -0.00036837         -0.02895096         -0.00006160
    14                  -0.00007896         -0.01591149         -0.00017014
    15                   0.00014634         -0.01511844          0.00010883
  ----------------------------------------------------------------------------
  ----------------------------------------------------------------------------
   Be, B0 AND B-B0 SHIFTS FOR SINGLY EXCITED VIBRATIONAL STATES (MHz)
  VIBRATION            X AXIS               Y AXIS                 Z AXIS
  ----------------------------------------------------------------------------
    Be              11064.55191046      96108.49529308       11349.17504372
    B0              10979.55081552      95117.17786932       11267.46898467
    Be-B0              85.00109494        991.31742376          81.70605905
    B'              10979.45202024      95117.30994995       11267.50190991
    Be-B'              85.09989022        991.18534313          81.67313381
    B''             10979.64459194      95117.33331453       11267.60001676
    Be-B''             84.90731852        991.16197855          81.57502696
    B^A             10979.39127325      95117.30974586       11267.56286098
    Be-B^A             85.16063721        991.18554722          81.61218274
    B^S             10979.49871862      95117.30997128       11267.45523528
    Be-B^S             85.05319184        991.18532180          81.71980844
     7                -88.42462801       -129.79163567         -81.10956897
     8                -35.48411736       -116.31836857         -20.64695858
     9                -20.28863936        900.34575510         -71.04163534
    10                 -8.85016870       -391.63017476          30.80928506
    11               -187.50056591      -1058.98264133         -22.40212944
    12                179.56919542        611.92418253           4.66374050
    13                -11.04333311       -867.92790966          -1.84680797
    14                 -2.36723808       -477.01458620          -5.10054933
    15                  4.38730524       -453.23946895           3.26250598
  ----------------------------------------------------------------------------
                 Vibrationally averaged dipole moment
------------------------------------------------------------------------
                 a.u.                            Debye
         x         y         z              x         y         z
------------------------------------------------------------------------
MU_e -0.02877   0.75417   0.00000       -0.07313   1.91679   0.00000
<MU> -0.02669   0.74853   0.00000       -0.06784   1.90244   0.00000

Equilibrium dipole moment:    0.75472 a.u. (   1.91818 D)
Equilibrium dipole moment:    0.74900 a.u. (   1.90365 D)
------------------------------------------------------------------------
********************************************************************************
    PARAMETERS RELEVANT TO VIBRATIONAL SPECTROSCOPY
********************************************************************************
  Quartic force constants written to file quartic
  ==============================================================
  Performing F(IIJJ)/F(JJII) consistency check
  Differences greater than 1 cm-1 will be printed.
  ==============================================================
  I    I    K    K       F(IIKK)       F(KKII)       Difference
  --------------------------------------------------------------
  --------------------------------------------------------------
  Largest absolute difference is    0.13200 cm-1.
  Largest relative difference is .60311D-02.
VPT2 vibrational analysis


 Thresholds for removing resonance denominators:
 Delta_omega = 50 cm-1; Phi(ijk) = 80 cm-1

-------------------------------
  ANHARMONICITY CONSTANTS X(ij)
                (cm-1)
-------------------------------
  (*) Near-zero denominators were removed
-------------------------------
          I  J         X(IJ)
-------------------------------
           7  7       -3.4443
           7  8       -3.7800
           7  9       -3.8597
           7 10       -1.4719
           7 11       -5.5830
           7 12       -0.9482
           7 13        0.1176
           7 14        1.0455
           7 15        2.3086
           8  8       -0.4996
           8  9       -1.0790
```

```
         8 10       -4.0201
         8 11       -0.3109
         8 12       -3.5775
         8 13       -3.9050
         8 14       -2.8279
         8 15        0.0328
         9  9       -1.8942
         9 10       -1.6963
         9 11       -7.7765
         9 12       -4.5293
         9 13        4.4647
         9 14       -3.1586
         9 15       -3.3092
        10 10       -5.1547
        10 11       -1.9946
        10 12        3.1032
        10 13        2.5110
        10 14      -17.1795
        10 15       -1.0973
        11 11       -5.2294
        11 12        1.1098
        11 13        0.7759
        11 14       -2.4499
        11 15      -17.9381
        12 12       -5.5046
        12 13       -6.0792
        12 14      -11.3850
        12 15      -21.5671
        13 13      -14.8593
        13 14      -61.9911
        13 15       -0.1415
        14 14      -19.1776
        14 15        1.1385
        15 15      -60.9724

 --------------------------------------------------------------------------------
          HARMONIC AND FUNDAMENTAL FREQUENCIES (cm-1) AND INTENSITIES (km/mol)
 --------------------------------------------------------------------------------

         Harmonic     Fundamental Anharmonic    Harmonic   Fundamental  Anharm
  Mode   Frequency    Frequency   Contribution  Intensity  Intensity    Contrib
    7    706.1993    693.2252       -12.9741     17.2023     18.1372      0.9349
    8    777.4431    766.7101       -10.7330      0.3050      0.3070      0.0019
    9    872.8407    858.5804       -14.2603      5.4155      5.2405     -0.1750
   10   1066.8187   1045.5865       -21.2322      5.7027      5.3312     -0.3715
   11   1274.8609   1247.3184       -27.5425      5.6992      5.0872     -0.6119
   12   1329.0386   1296.0926       -32.9459      5.7563      5.8193      0.0630
   13   2245.5778   2183.7352       -61.8426     11.5298     10.9347     -0.5951
   14   2346.6818   2259.9226       -86.7592      2.5575      2.7143      0.1568
   15   3143.5899   3001.3585      -142.2314      9.1337     10.9405      1.8068
 --------------------------------------------------------------------------------
 --------------------------------------------------------------------------------
                          ZERO-POINT VIBRATIONAL ENERGIES
 --------------------------------------------------------------------------------
                            kcal/mol      kJ/mol        Hartree        cm-1
 --------------------------------------------------------------------------------
 Harmonic contribution :       19.6752      82.3212      0.03135453      6881.525
 VPT2-correction       :       -0.2309      -0.9659     -0.00036789       -80.743
 --------------------------------------------------------------------------------
 Harm+VPT2             :       19.4444      81.3553      0.03098664      6800.782
 --------------------------------------------------------------------------------
  MAXLEVEL set to                    3
  -------------------------------------------------------------------------------
                  All levels with up to three quanta
 --------------------------------------------------------------------------------
  MODE MODE MODE MODE MODE                       Anharmonic    Anharm      Harmonic
   I    J    K    L    M   NI  NJ  NK  NL  NM Frequency   Intensity   Transition
 --------------------------------------------------------------------------------
    7    0    0    0    0  1   0   0   0   0    693.225   18.136805    706.199306
    8    0    0    0    0  1   0   0   0   0    766.710    0.306982    777.443141
    9    0    0    0    0  1   0   0   0   0    858.580    5.240395    872.840664
   10    0    0    0    0  1   0   0   0   0   1045.586    5.331136   1066.818661
   11    0    0    0    0  1   0   0   0   0   1247.318    5.087146   1274.860892
   12    0    0    0    0  1   0   0   0   0   1296.092    5.819221   1329.038582
    7    0    0    0    0  2   0   0   0   0   1379.561    0.115520   1412.398611
    8    7    0    0    0  1   1   0   0   0   1456.155    0.012279   1483.642447
    8    0    0    0    0  2   0   0   0   0   1532.421    0.001884   1554.886282
    9    7    0    0    0  1   1   0   0   0   1547.945    0.003517   1579.039969
```

9	8	0	0	0	1	1	0	0	0	1624.211	0.021054	1650.283805
9	0	0	0	0	2	0	0	0	0	1713.372	0.043297	1745.681328
10	7	0	0	0	1	1	0	0	0	1737.339	0.039930	1773.017967
10	8	0	0	0	1	1	0	0	0	1808.276	0.062890	1844.261802
10	9	0	0	0	1	1	0	0	0	1902.470	0.045468	1939.659325
11	7	0	0	0	1	1	0	0	0	1934.960	0.056656	1981.060198
12	7	0	0	0	1	1	0	0	0	1988.369	0.002654	2035.237887
11	8	0	0	0	1	1	0	0	0	2013.717	0.004013	2052.304033
7	0	0	0	0	3	0	0	0	0	2059.010	0.000000	2118.597917
12	8	0	0	0	1	1	0	0	0	2059.225	0.077002	2106.481723
10	0	0	0	0	2	0	0	0	0	2080.863	0.618706	2133.637323
11	9	0	0	0	1	1	0	0	0	2098.122	0.445147	2147.701556
8	7	0	0	0	1	2	0	0	0	2138.711	0.000000	2189.841752
12	9	0	0	0	1	1	0	0	0	2150.143	0.000170	2201.879246
13	0	0	0	0	1	0	0	0	0	2183.735	10.934464	2245.577792
8	7	0	0	0	2	1	0	0	0	2218.086	0.000000	2261.085588
9	7	0	0	0	1	2	0	0	0	2230.422	0.000000	2285.239275
14	0	0	0	0	1	0	0	0	0	2259.922	2.714252	2346.681846
11	10	0	0	0	1	1	0	0	0	2290.910	0.196328	2341.679553
8	0	0	0	0	3	0	0	0	0	2297.132	0.000000	2332.329423
9	8	7	0	0	1	1	1	0	0	2309.796	0.000000	2356.483111
12	10	0	0	0	1	1	0	0	0	2344.782	0.611380	2395.857243
9	8	0	0	0	1	2	0	0	0	2388.843	0.000000	2427.726946
9	7	0	0	0	2	1	0	0	0	2398.878	0.000000	2451.880633
10	7	0	0	0	1	2	0	0	0	2422.204	0.000000	2479.217273
9	8	0	0	0	2	1	0	0	0	2477.924	0.000000	2523.124469
11	0	0	0	0	2	0	0	0	0	2484.177	0.139390	2549.721784
10	8	7	0	0	1	1	1	0	0	2496.249	0.000000	2550.461108
12	11	0	0	0	1	1	0	0	0	2544.520	0.044213	2603.899474
9	0	0	0	0	3	0	0	0	0	2564.375	0.000000	2618.521992
10	8	0	0	0	1	2	0	0	0	2569.967	0.000000	2621.704943
12	0	0	0	0	2	0	0	0	0	2581.176	0.146784	2658.077163
10	9	7	0	0	1	1	1	0	0	2590.364	0.000000	2645.858631
11	7	0	0	0	1	2	0	0	0	2615.714	0.000000	2687.259503
10	9	8	0	0	1	1	1	0	0	2664.081	0.000000	2717.102466
12	7	0	0	0	1	2	0	0	0	2673.758	0.000000	2741.437193
11	8	7	0	0	1	1	1	0	0	2697.579	0.000000	2758.503339
12	8	7	0	0	1	1	1	0	0	2747.722	0.000000	2812.681028
10	9	0	0	0	1	2	0	0	0	2755.566	0.000000	2812.499989
10	7	0	0	0	2	1	0	0	0	2771.145	0.000000	2839.836628
11	8	0	0	0	1	2	0	0	0	2779.117	0.000000	2829.747174
11	9	7	0	0	1	1	1	0	0	2781.904	0.000000	2853.900862
12	8	0	0	0	1	2	0	0	0	2821.358	0.000000	2883.924864
12	9	7	0	0	1	1	1	0	0	2838.561	0.000000	2908.078551
10	8	0	0	0	2	1	0	0	0	2839.533	0.000000	2911.080464
11	9	8	0	0	1	1	1	0	0	2863.442	0.000000	2925.144697
13	7	0	0	0	1	1	0	0	0	2877.078	0.092500	2951.777097
12	9	8	0	0	1	1	1	0	0	2912.197	0.000000	2979.322387
10	9	0	0	0	2	1	0	0	0	2936.051	0.000000	3006.477987
11	9	0	0	0	1	2	0	0	0	2945.137	0.000000	3020.542220
13	8	0	0	0	1	1	0	0	0	2946.540	0.003892	3023.020933
14	7	0	0	0	1	1	0	0	0	2954.193	0.020158	3052.881152
11	10	7	0	0	1	1	1	0	0	2977.080	0.000000	3047.878859
12	9	0	0	0	1	2	0	0	0	3000.406	0.000000	3074.719909
15	0	0	0	0	1	0	0	0	0	3001.358	10.940258	3143.589896
14	8	0	0	0	1	1	0	0	0	3023.804	0.046757	3124.124987
12	10	7	0	0	1	1	1	0	0	3035.587	0.000000	3102.056549
13	9	0	0	0	1	1	0	0	0	3046.780	0.327968	3118.418456
11	10	8	0	0	1	1	1	0	0	3053.289	0.000000	3119.122694
12	10	8	0	0	1	1	1	0	0	3103.894	0.000000	3173.300384
10	0	0	0	0	3	0	0	0	0	3105.831	0.000000	3200.455984
14	9	0	0	0	1	1	0	0	0	3115.344	0.009531	3219.522510
11	10	9	0	0	1	1	1	0	0	3140.017	0.000000	3214.520217
11	7	0	0	0	2	1	0	0	0	3166.237	0.000000	3255.921090
12	10	9	0	0	1	1	1	0	0	3197.137	0.000000	3268.697907
12	11	7	0	0	1	1	1	0	0	3231.214	0.000000	3310.098779
13	10	0	0	0	1	1	0	0	0	3231.832	0.054257	3312.396453
11	8	0	0	0	2	1	0	0	0	3250.266	0.000000	3327.164925
12	7	0	0	0	2	1	0	0	0	3272.504	0.000000	3364.276469
14	10	0	0	0	1	1	0	0	0	3288.329	0.311145	3413.500508
12	11	8	0	0	1	1	1	0	0	3307.342	0.000000	3381.342615
11	10	0	0	0	1	2	0	0	0	3324.192	0.000000	3408.498215
11	9	0	0	0	2	1	0	0	0	3327.205	0.000000	3422.562448
12	8	0	0	0	2	1	0	0	0	3340.731	0.000000	3435.520304
12	10	0	0	0	1	2	0	0	0	3383.162	0.000000	3462.675905
12	11	9	0	0	1	1	1	0	0	3390.795	0.000000	3476.740138
12	9	0	0	0	2	1	0	0	0	3430.697	0.000000	3530.917827
13	11	0	0	0	1	1	0	0	0	3431.829	0.064190	3520.438684

13	12	0	0	0	1	1	0	0	0	3473.748	0.274987	3574.616373
14	11	0	0	0	1	1	0	0	0	3504.791	0.016698	3621.542738
11	10	0	0	0	2	1	0	0	0	3525.775	0.000000	3616.540446
14	12	0	0	0	1	1	0	0	0	3544.630	0.216222	3675.720428
13	7	0	0	0	1	2	0	0	0	3563.532	0.000000	3657.976403
12	11	10	0	0	1	1	1	0	0	3591.215	0.000000	3670.718135
12	10	0	0	0	2	1	0	0	0	3632.968	0.000000	3724.895825
13	8	7	0	0	1	1	1	0	0	3636.103	0.000000	3729.220238
14	7	0	0	0	1	2	0	0	0	3641.575	0.000000	3759.080458
15	7	0	0	0	1	1	0	0	0	3696.892	0.057035	3849.789202
13	8	0	0	0	1	2	0	0	0	3708.346	0.000000	3800.464074
11	0	0	0	0	3	0	0	0	0	3710.578	0.000000	3824.582676
14	8	7	0	0	1	1	1	0	0	3714.295	0.000000	3830.324293
13	9	7	0	0	1	1	1	0	0	3736.263	0.000000	3824.617761
15	8	0	0	0	1	1	0	0	0	3768.101	0.003322	3921.033038
12	11	0	0	0	1	2	0	0	0	3782.490	0.000000	3878.760366
14	8	0	0	0	1	2	0	0	0	3786.687	0.000000	3901.568128
14	9	7	0	0	1	1	1	0	0	3805.755	0.000000	3925.721816
13	9	8	0	0	1	1	1	0	0	3808.506	0.000000	3895.861597
12	11	0	0	0	2	1	0	0	0	3830.714	0.000000	3932.938055
12	0	0	0	0	3	0	0	0	0	3855.250	0.000000	3987.115745
15	9	0	0	0	1	1	0	0	0	3856.629	0.298736	4016.430560
14	9	8	0	0	1	1	1	0	0	3878.147	0.000000	3996.965651
13	9	0	0	0	1	2	0	0	0	3906.036	0.000000	3991.259120
13	10	7	0	0	1	1	1	0	0	3923.703	0.000000	4018.595759
14	9	0	0	0	1	2	0	0	0	3966.977	0.000000	4092.363174
14	10	7	0	0	1	1	1	0	0	3981.128	0.000000	4119.699813
13	10	8	0	0	1	1	1	0	0	3990.617	0.000000	4089.839594
15	10	0	0	0	1	1	0	0	0	4045.847	0.065020	4210.408558
14	10	8	0	0	1	1	1	0	0	4048.191	0.000000	4190.943649
13	10	9	0	0	1	1	1	0	0	4093.181	0.000000	4185.237117
13	11	7	0	0	1	1	1	0	0	4119.589	0.000000	4226.637989
14	10	9	0	0	1	1	1	0	0	4142.055	0.000000	4286.341172
13	12	7	0	0	1	1	1	0	0	4166.143	0.000000	4280.815679
14	11	7	0	0	1	1	1	0	0	4193.478	0.000000	4327.742044
13	11	8	0	0	1	1	1	0	0	4194.323	0.000000	4297.881825
15	11	0	0	0	1	1	0	0	0	4230.738	0.568254	4418.450789
13	12	8	0	0	1	1	1	0	0	4232.976	0.000000	4352.059515
14	12	7	0	0	1	1	1	0	0	4237.952	0.000000	4381.919734
14	11	8	0	0	1	1	1	0	0	4268.362	0.000000	4398.985879
13	10	0	0	0	1	2	0	0	0	4269.620	0.000000	4379.215115
15	12	0	0	0	1	1	0	0	0	4275.884	0.054785	4472.628478
13	11	9	0	0	1	1	1	0	0	4287.098	0.000000	4393.279348
14	12	8	0	0	1	1	1	0	0	4304.934	0.000000	4453.163569
14	10	0	0	0	1	2	0	0	0	4306.427	0.000000	4480.319169
13	12	9	0	0	1	1	1	0	0	4332.264	0.000000	4447.457037
13	0	0	0	0	2	0	0	0	0	4337.751	0.031431	4491.155584
14	11	9	0	0	1	1	1	0	0	4352.436	0.000000	4494.383402
14	13	0	0	0	1	1	0	0	0	4381.666	0.261157	4592.259638
15	7	0	0	0	1	2	0	0	0	4385.537	0.000000	4555.988508
14	12	9	0	0	1	1	1	0	0	4395.522	0.000000	4548.561092
15	8	7	0	0	1	1	1	0	0	4459.855	0.000000	4627.232343
13	11	10	0	0	1	1	1	0	0	4477.932	0.000000	4587.257345
14	0	0	0	0	2	0	0	0	0	4481.490	0.186380	4693.363693
13	12	10	0	0	1	1	1	0	0	4524.949	0.000000	4641.435035
14	11	10	0	0	1	1	1	0	0	4531.203	0.000000	4688.361400
15	8	0	0	0	1	2	0	0	0	4533.845	0.000000	4698.476179
15	9	7	0	0	1	1	1	0	0	4548.303	0.000000	4722.629866
14	12	10	0	0	1	1	1	0	0	4576.140	0.000000	4742.539089
15	9	8	0	0	1	1	1	0	0	4622.293	0.000000	4793.873701
13	11	0	0	0	1	2	0	0	0	4669.464	0.000000	4795.299576
15	9	0	0	0	1	2	0	0	0	4708.112	0.000000	4889.271224
13	12	11	0	0	1	1	1	0	0	4722.952	0.000000	4849.477266
14	11	0	0	0	1	2	0	0	0	4739.200	0.000000	4896.403630
15	10	7	0	0	1	1	1	0	0	4739.909	0.000000	4916.607863
13	12	0	0	0	1	2	0	0	0	4752.752	0.000000	4903.654955
14	12	11	0	0	1	1	1	0	0	4790.608	0.000000	4950.581320
15	10	8	0	0	1	1	1	0	0	4808.570	0.000000	4987.851699
14	12	0	0	0	1	2	0	0	0	4818.328	0.000000	5004.759010
15	10	9	0	0	1	1	1	0	0	4899.422	0.000000	5083.249222
15	11	7	0	0	1	1	1	0	0	4920.689	0.000000	5124.650094
15	12	7	0	0	1	1	1	0	0	4970.469	0.000000	5178.827784
15	11	8	0	0	1	1	1	0	0	4997.170	0.000000	5195.893930
13	7	0	0	0	2	1	0	0	0	5031.212	0.000000	5197.354889
15	12	8	0	0	1	1	1	0	0	5039.049	0.000000	5250.071619
14	13	7	0	0	1	1	1	0	0	5076.055	0.000000	5298.458944
15	11	9	0	0	1	1	1	0	0	5078.233	0.000000	5291.291452
15	10	0	0	0	1	2	0	0	0	5080.027	0.000000	5277.227219

13	8	0	0	0	2	1	0	0	0	5096.651	0.000000	5268.598725
15	12	9	0	0	1	1	1	0	0	5126.625	0.000000	5345.469142
14	13	8	0	0	1	1	1	0	0	5141.643	0.000000	5369.702779
14	7	0	0	0	2	1	0	0	0	5176.806	0.000000	5399.562998
15	13	0	0	0	1	1	0	0	0	5184.952	0.041488	5389.167688
13	9	0	0	0	2	1	0	0	0	5205.261	0.000000	5363.996248
14	13	9	0	0	1	1	1	0	0	5241.553	0.000000	5465.100302
14	8	0	0	0	2	1	0	0	0	5242.544	0.000000	5470.806834
15	14	0	0	0	1	1	0	0	0	5262.419	0.043044	5490.271743
15	11	10	0	0	1	1	1	0	0	5273.233	0.000000	5485.269450
15	12	10	0	0	1	1	1	0	0	5323.476	0.000000	5539.447140
14	9	0	0	0	2	1	0	0	0	5333.753	0.000000	5566.204357
13	10	0	0	0	2	1	0	0	0	5388.360	0.000000	5557.974245
14	13	10	0	0	1	1	1	0	0	5412.584	0.000000	5659.078300
15	11	0	0	0	1	2	0	0	0	5449.660	0.000000	5693.311681
14	10	0	0	0	2	1	0	0	0	5492.717	0.000000	5760.182354
15	12	11	0	0	1	1	1	0	0	5506.374	0.000000	5747.489370
15	12	0	0	0	1	2	0	0	0	5539.400	0.000000	5801.667060
13	11	0	0	0	2	1	0	0	0	5586.621	0.000000	5766.016476
13	12	0	0	0	2	1	0	0	0	5621.685	0.000000	5820.194165
14	13	11	0	0	1	1	1	0	0	5627.311	0.000000	5867.120530
14	13	12	0	0	1	1	1	0	0	5660.295	0.000000	5921.298220
14	11	0	0	0	2	1	0	0	0	5723.908	0.000000	5968.224585
14	12	0	0	0	2	1	0	0	0	5754.812	0.000000	6022.402274
15	13	7	0	0	1	1	1	0	0	5880.603	0.000000	6095.366994
15	0	0	0	0	2	0	0	0	0	5880.772	0.500211	6287.179793
15	13	8	0	0	1	1	1	0	0	5947.789	0.000000	6166.610829
15	14	7	0	0	1	1	1	0	0	5958.998	0.000000	6196.471048
15	14	8	0	0	1	1	1	0	0	6026.334	0.000000	6267.714884
15	13	9	0	0	1	1	1	0	0	6044.687	0.000000	6262.008352
15	14	9	0	0	1	1	1	0	0	6114.532	0.000000	6363.112407
15	13	10	0	0	1	1	1	0	0	6231.952	0.000000	6455.986350
15	14	10	0	0	1	1	1	0	0	6289.729	0.000000	6557.090404
15	13	11	0	0	1	1	1	0	0	6415.108	0.000000	6664.028580
15	13	12	0	0	1	1	1	0	0	6453.398	0.000000	6718.206270
13	0	0	0	0	3	0	0	0	0	6462.049	0.000000	6736.733375
14	13	0	0	0	1	2	0	0	0	6473.692	0.000000	6837.837430
15	14	11	0	0	1	1	1	0	0	6489.350	0.000000	6765.132635
15	14	12	0	0	1	1	1	0	0	6525.560	0.000000	6819.310325
14	13	0	0	0	2	1	0	0	0	6541.243	0.000000	6938.941485
15	7	0	0	0	2	1	0	0	0	6578.614	0.000000	6993.379099
15	8	0	0	0	2	1	0	0	0	6647.547	0.000000	7064.622934
14	0	0	0	0	3	0	0	0	0	6664.702	0.000000	7040.045539
15	9	0	0	0	2	1	0	0	0	6732.734	0.000000	7160.020457
15	10	0	0	0	2	1	0	0	0	6924.164	0.000000	7353.998454
15	11	0	0	0	2	1	0	0	0	7092.214	0.000000	7562.040685
15	12	0	0	0	2	1	0	0	0	7133.730	0.000000	7616.218375
15	13	0	0	0	1	2	0	0	0	7338.827	0.000000	7634.745480
15	14	13	0	0	1	1	1	0	0	7384.022	0.000000	7735.849535
15	14	0	0	0	1	2	0	0	0	7485.125	0.000000	7836.953589
15	13	0	0	0	2	1	0	0	0	8064.224	0.000000	8532.757585
15	14	0	0	0	2	1	0	0	0	8142.971	0.000000	8633.861639
15	0	0	0	0	3	0	0	0	0	8638.241	0.000000	9430.769689

```
--------------------------------------------------------------------------------
 Dipole moment function written to files dipolex(y,z)
  @CHECKOUT-I, Total execution time :       0.0000 seconds.
 --executable xcubic finished with status            0
```

Table S.12. Part of the CFOUR output file of the CCSD(T)/VQZ-aV(Q+*d*)Z anharmonic frequency calculation for $^{13}CHD_2{}^{37}Cl$

```
   *************************************************************************
         <<<     CCCCCC     CCCCCC   |||     CCCCCC     CCCCCC   >>>
       <<<      CCC        CCC       |||    CCC        CCC         >>>
      <<<      CCC        CCC        |||   CCC        CCC            >>>
    <<<        CCC        CCC        |||   CCC        CCC              >>>
      <<<      CCC        CCC        |||   CCC        CCC            >>>
       <<<      CCC        CCC       |||    CCC        CCC         >>>
         <<<     CCCCCC     CCCCCC   |||     CCCCCC     CCCCCC   >>>
   *************************************************************************

     ****************************************************************
     * CFOUR Coupled-Cluster techniques for Computational Chemistry *
     ****************************************************************


   Department of Chemistry                   Institut fuer Physikalische Chemie
   University of Texas at Austin             Universitaet Mainz
   Austin, TX  78712, USA                    D-55099 Mainz, Germany

                     Version 1.0



                                 Normal Coordinates

              A'                         A''                         A'
            699.29                     776.16                      870.13
           VIBRATION                  VIBRATION                  VIBRATION
 C      0.053 0.6862  0.0000    0.0000  0.0000  0.3684    0.4263 -0.3279  0.0000
 CL    -0.016-0.5661  0.0000    0.0000  0.0000 -0.1601   -0.1273  0.0746  0.0000
 H     -0.025 0.3147  0.0140   -0.0225  0.6367 -0.0860   -0.2736  0.4129  0.0402
 H     -0.025 0.3147 -0.0140    0.0225 -0.6367 -0.0860   -0.2736  0.4129 -0.0402
 H     -0.017 0.0738  0.0000    0.0000  0.0000 -0.1102    0.0132 -0.4414  0.0000

              A'                          A'                        A''
            1066.43                    1265.09                     1321.14
           VIBRATION                  VIBRATION                  VIBRATION
 C      0.261 0.2831  0.0000    0.0574 -0.2618  0.0000    0.0000  0.0000  0.2542
 CL     0.001 0.0147  0.0000    0.0480 -0.0413  0.0000    0.0000  0.0000  0.0047
 H     -0.339-0.3509  0.4251   -0.2649  0.1303  0.0727    0.3267 -0.1383 -0.0412
 H     -0.339-0.3509 -0.4251   -0.2649  0.1303 -0.0727   -0.3267  0.1383 -0.0412
 H      0.011-0.1139  0.0000    0.2520  0.8220  0.0000    0.0000  0.0000 -0.8248

              A'                         A''                         A'
            2244.05                    2344.63                     3192.88
           VIBRATION                  VIBRATION                  VIBRATION
 C      0.226 0.1779  0.0000    0.0000  0.0000  0.4182    0.2625 -0.0617  0.0000
 CL    -0.001-0.0030  0.0000    0.0000  0.0000 -0.0026   -0.0007 -0.0002  0.0000
 H     -0.313-0.2121 -0.5582   -0.3022 -0.2026 -0.5293   -0.0036 -0.0104 -0.0252
 H     -0.313-0.2121  0.5582    0.3022  0.2026 -0.5293   -0.0036 -0.0104  0.0252
 H      0.083-0.0210  0.0000    0.0000  0.0000  0.0104   -0.9285  0.2522  0.0000

 ...
 ...

  ----------------------------------------------------------------
                  Dipole Moment Function
                 (Normal Coordinate Basis)
  ----------------------------------------------------------------
    Mode     Symmetry    d(Mu(x))/dQ   d(Mu(y))/dQ   d(Mu(z))/dQ
  ----------------------------------------------------------------
     Q7          A'       -0.007736      0.131361      0.000000
     Q8         A''        0.000000      0.000000     -0.018460
     Q9          A'       -0.028180     -0.067431      0.000000
     Q10         A'       -0.047301      0.061575      0.000000
     Q11         A'       -0.008774     -0.076354      0.000000
     Q12        A''        0.000000      0.000000     -0.076280
     Q13         A'        0.021846      0.106546      0.000000
     Q14        A''        0.000000      0.000000      0.051673
     Q15         A'        0.056152     -0.076006      0.000000
  ----------------------------------------------------------------
 ---------------------------------------------------------------------------
 ------------------------------------------------------------
         Parameter              (MHz)                (CM-1)
```

```
------------------------------------------------------------

          R6                -.118171E-04           -.394176E-09
          R5                -.110721E-01           -.369326E-06
          SI                            0.614094E+03

  A-reduced centrifugal distortion parameters

          DJ                0.113937E-01           0.380053E-06
          DK                0.938099E+00           0.312916E-04
          DJK               0.119890E+00           0.399909E-05
          DELJ              0.114173E-01           0.380842E-06
          DELK              0.938217E+00           0.312955E-04
          DELJK             0.119748E+00           0.399436E-05
          delJ              0.262404E-03           0.875285E-08
          delK              0.511714E-01           0.170690E-05

  S-reduced centrifugal distortion parameters

          DJ                0.113757E-01           0.379452E-06
          DK                0.938008E+00           0.312886E-04
          DJK               0.119998E+00           0.400270E-05
          D1                -.262404E-03           -.875285E-08
          D2                -.208321E-04           -.694884E-09
------------------------------------------------------------
   Vibrational frequencies after rotational projection  of
   Cartesian force constants:
           1              0.0000i
           2              0.0000i
           3              0.0000i
           4              0.0000
           5              0.0000
           6              0.0000
           7            699.2864
           8            776.1374
           9            870.1265
          10           1066.4291
          11           1265.0819
          12           1321.0630
          13           2244.0502
          14           2344.6322
          15           3192.8838
  Zero-point vibrational energy:   19.6990 kcal/mol =   82.4209 kJ/mol.
              0.12 seconds walltime passed
 --executable xjoda finished with status            0
 --invoking executable xcubic
  @GETMEM-I,   Allocated   13351 MB of main memory.
  back



********************************************************************************
        PARAMETERS RELEVANT TO MOLECULAR STRUCTURE
********************************************************************************

  Force Field from Numerical Differentiation of Analytic Second Derivatives
  ----------------------------------------------------------------------------
 Cubic force constants written to file cubic.

---------------------------------------------------------------------------
  Mean and mean-square displacements in dimensionless normal coordinates

             Geometrical properties evaluated at     0.00 K

         Coordinate             <q>           <q**2>
---------------------------------------------------------------------------
             7            0.1312022034     0.4999999889
             8            0.0000000000     0.4999999889
             9           -0.0270787460     0.4999999889
            10            0.0025262379     0.4999999889
            11            0.0140444010     0.4999999889
            12            0.0000000000     0.4999999889
            13           -0.1279622719     0.4999999889
            14            0.0000000000     0.4999999889
            15            0.0788287507     0.4999999889
---------------------------------------------------------------------------

  ------------------------------------------------------
```

ATOM		INTERNUCLEAR DISTANCE / Angstrom		
I	J	Re	Rg	Ra
2	1	1.7826656	1.7906503	1.7903618
3	1	1.0850773	1.1004058	1.0929121
3	2	2.3617095	2.3755295	2.3737302
4	1	1.0850773	1.1004058	1.0929121
4	2	2.3617095	2.3755295	2.3737302
4	3	1.7831251	1.8021984	1.7958888
5	1	1.0850773	1.1061533	1.0941687
5	2	2.3617095	2.3797351	2.3761231
5	3	1.7831251	1.8061588	1.7969378
5	4	1.7831251	1.8061588	1.7969378

****

PARAMETERS RELEVANT TO ROTATIONAL SPECTROSCOPY

****

		VIB-ROT CONSTANT / (cm-1)			
AXIS	MODE	CORIOLIS	QUADRATIC	ANHARMONIC	TOTAL
1	7	0.0004846	-0.0010357	0.0033834	0.0028322
1	8	0.0004573	-0.0000001	0.0007437	0.0012009
1	9	0.0000059	-0.0002011	0.0008624	0.0006672
1	10	0.0001374	-0.0000247	0.0001779	0.0002906
1	11	0.0053732	-0.0000713	0.0007427	0.0060446
1	12	-0.0056781	-0.0000087	-0.0000871	-0.0057738
1	13	-0.0002518	-0.0000247	0.0006327	0.0003562
1	14	-0.0003782	-0.0000043	0.0004570	0.0000745
1	15	-0.0000176	-0.0000037	-0.0001253	-0.0001467
2	7	0.0005503	-0.0000329	0.0037414	0.0042588
2	8	0.0271967	-0.0045594	-0.0188098	0.0038276
2	9	-0.0110817	-0.0035325	-0.0153029	-0.0299171
2	10	0.0225715	-0.0037795	-0.0057234	0.0130687
2	11	0.0407914	-0.0023072	-0.0032044	0.0352798
2	12	-0.0149663	-0.0001176	-0.0052736	-0.0203575
2	13	-0.0034696	-0.0198777	0.0522993	0.0289519
2	14	-0.0343021	-0.0001527	0.0503675	0.0159128
2	15	-0.0197519	-0.0030392	0.0379076	0.0151164
3	7	0.0001844	-0.0010929	0.0035380	0.0026295
3	8	0.0002615	-0.0005292	0.0009489	0.0006813
3	9	0.0014670	-0.0000177	0.0008572	0.0023064
3	10	-0.0010952	-0.0000187	0.0001296	-0.0009842
3	11	0.0000302	-0.0000007	0.0007076	0.0007371
3	12	-0.0001449	-0.0000226	0.0000174	-0.0001500
3	13	-0.0001686	-0.0000043	0.0002326	0.0000597
3	14	-0.0000904	-0.0000222	0.0002750	0.0001624
3	15	-0.0003204	-0.0000062	0.0002160	-0.0001106

Be, B0 AND B-B0 SHIFTS FOR SINGLY EXCITED VIBRATIONAL STATES (CM-1)

VIBRATION	X AXIS	Y AXIS	Z AXIS
Be	0.36282140	3.20580736	0.37199210
B0	0.36004854	3.17273669	0.36932632
Be-B0	0.00277285	0.03307067	0.00266578
B'	0.36004535	3.17274096	0.36932738
Be-B'	0.00277604	0.03306640	0.00266472
B''	0.36005158	3.17274171	0.36933055
Be-B''	0.00276982	0.03306564	0.00266155
B^A	0.36004339	3.17274095	0.36932935
Be-B^A	0.00277801	0.03306641	0.00266275
B^S	0.36004686	3.17274096	0.36932587
Be-B^S	0.00277453	0.03306640	0.00266623
7	-0.00283224	-0.00425877	-0.00262947
8	-0.00120092	-0.00382755	-0.00068126
9	-0.00066721	0.02991711	-0.00230644
10	-0.00029057	-0.01306865	0.00098424
11	-0.00604458	-0.03527983	-0.00073713
12	0.00577385	0.02035752	0.00015000
13	-0.00035619	-0.02895194	-0.00005971

```
    14                -0.00007449         -0.01591280         -0.00016238
    15                 0.00014666         -0.01511642          0.00011060
  ---------------------------------------------------------------------------
  ---------------------------------------------------------------------------
   Be, B0 AND B-B0 SHIFTS FOR SINGLY EXCITED VIBRATIONAL STATES (MHz)
  VIBRATION            X AXIS              Y AXIS                 Z AXIS
  ---------------------------------------------------------------------------
    Be              10877.11179173      96107.68676687      11152.04253561
    B0              10793.98378386      95116.25315455      11072.12445102
    Be-B0              83.12800787        991.43361232         79.91808459
    B'              10793.88811594      95116.38106059      11072.15632479
    Be-B'              83.22367579        991.30570628         79.88621082
    B''             10794.07468242      95116.40368103      11072.25140244
    Be-B''             83.03710931        991.28308584         79.79113316
    B^A             10793.82925196      95116.38087001      11072.21537935
    Be-B^A             83.28253977        991.30589686         79.82715625
    B^S             10793.93335159      95116.38108049      11072.11111135
    Be-B^S             83.17844014        991.30568638         79.93142426
     7                -84.90838911       -127.67466944        -78.82957416
     8                -36.00275537       -114.74712255        -20.42366407
     9                -20.00251139        896.89240601        -69.14545447
    10                 -8.71104958       -391.78830211         29.50679051
    11               -181.21194371      -1057.66281883        -22.09858052
    12                173.09559065        610.30318938          4.49701089
    13                -10.67837820       -867.95724962         -1.79008378
    14                 -2.23325965       -477.05367813         -4.86817214
    15                  4.39668062       -453.17897935          3.31555856
  ---------------------------------------------------------------------------
                 Vibrationally averaged dipole moment
-------------------------------------------------------------------------
                  a.u.                        Debye
         x         y         z             x         y         z
-------------------------------------------------------------------------
MU_e -0.02866   0.75418   0.00000       -0.07285   1.91680   0.00000
<MU> -0.02658   0.74847   0.00000       -0.06756   1.90231   0.00000

Equilibrium dipole moment:    0.75472 a.u. (   1.91818 D)
Equilibrium dipole moment:    0.74895 a.u. (   1.90351 D)
-------------------------------------------------------------------------
*******************************************************************************
    PARAMETERS RELEVANT TO VIBRATIONAL SPECTROSCOPY
*******************************************************************************
  Quartic force constants written to file quartic
  ================================================================
  Performing F(IIJJ)/F(JJII) consistency check
  Differences greater than 1 cm-1 will be printed.
  ================================================================
  I    I    K    K       F(IIKK)       F(KKII)       Difference
  ---------------------------------------------------------------
  ---------------------------------------------------------------
  Largest absolute difference is    0.41293 cm-1.
  Largest relative difference is .68662D+00.
VPT2 vibrational analysis


 Thresholds for removing resonance denominators:
 Delta_omega = 50 cm-1; Phi(ijk) = 80 cm-1

-------------------------------
  ANHARMONICITY CONSTANTS X(ij)
                (cm-1)
-------------------------------
  (*) Near-zero denominators were removed
-------------------------------
          I  J         X(IJ)
-------------------------------
          7  7       -3.3714
          7  8       -3.7428
          7  9       -3.8168
          7 10       -1.4736
          7 11       -5.5934
          7 12       -0.9366
          7 13        0.1323
          7 14        1.0575
          7 15        2.2810
          8  8       -0.4962
          8  9       -1.0456
          8 10       -3.9938
```

```
          8 11       -0.3185
          8 12       -3.5968
          8 13       -3.9226
          8 14       -2.8244
          8 15        0.0167
          9  9       -1.8702
          9 10       -1.6960
          9 11       -7.7535
          9 12       -4.5592
          9 13        4.4334
          9 14       -3.1958
          9 15       -3.3331
         10 10       -5.1564
         10 11       -1.9978
         10 12        3.1020
         10 13        2.5136
         10 14      -17.1781
         10 15       -1.0941
         11 11       -5.2326
         11 12        1.1040
         11 13        0.7485
         11 14       -2.4503
         11 15      -17.9321
         12 12       -5.5073
         12 13       -6.0815
         12 14      -11.3931
         12 15      -21.5609
         13 13      -14.8594
         13 14      -61.9914
         13 15       -0.1682
         14 14      -19.1777
         14 15        1.1359
         15 15      -60.9723

--------------------------------------------------------------------------------
          HARMONIC AND FUNDAMENTAL FREQUENCIES (cm-1) AND INTENSITIES (km/mol)
--------------------------------------------------------------------------------

         Harmonic     Fundamental Anharmonic    Harmonic   Fundamental  Anharm
  Mode   Frequency    Frequency   Contribution  Intensity  Intensity    Contrib
    7     699.8107     687.0217      -12.7889    16.9459     17.8522     0.9063
    8     776.8756     766.1692      -10.7064     0.3148      0.3167     0.0019
    9     872.3265     858.1028      -14.2238     5.3103      5.1470    -0.1633
   10    1066.8127    1045.5910      -21.2217     5.7162      5.3460    -0.3702
   11    1274.7179    1247.1561      -27.5618     5.7411      5.1214    -0.6197
   12    1329.0379    1296.0622      -32.9757     5.7553      5.8177     0.0624
   13    2245.5771    2183.6904      -61.8866    11.5267     10.9388    -0.5879
   14    2346.6814    2259.9061      -86.7753     2.5570      2.7142     0.1571
   15    3143.5898    3001.3178     -142.2720     9.1337     10.9616     1.8278
--------------------------------------------------------------------------------
--------------------------------------------------------------------------------
                            ZERO-POINT VIBRATIONAL ENERGIES
--------------------------------------------------------------------------------
                              kcal/mol      kJ/mol        Hartree        cm-1
--------------------------------------------------------------------------------
Harmonic contribution :        19.6643     82.2756      0.03133717      6877.715
VPT2-correction       :        -0.2308     -0.9655     -0.00036774       -80.710
--------------------------------------------------------------------------------
Harm+VPT2             :        19.4336     81.3101      0.03096943      6797.004
--------------------------------------------------------------------------------
  MAXLEVEL set to                        3
 -------------------------------------------------------------------------------
                  All levels with up to three quanta
--------------------------------------------------------------------------------
  MODE MODE MODE MODE MODE                          Anharmonic   Anharm      Harmonic
   I    J    K    L    M   NI   NJ   NK   NL   NM Frequency   Intensity   Transition
--------------------------------------------------------------------------------
    7    0    0    0    0  1    0    0    0    0    687.021   17.851838    699.810666
    8    0    0    0    0  1    0    0    0    0    766.169    0.316657    776.875573
    9    0    0    0    0  1    0    0    0    0    858.102    5.146865    872.326546
   10    0    0    0    0  1    0    0    0    0   1045.590    5.345863   1066.812671
   11    0    0    0    0  1    0    0    0    0   1247.156    5.121331   1274.717879
   12    0    0    0    0  1    0    0    0    0   1296.062    5.817536   1329.037884
    7    0    0    0    0  2    0    0    0    0   1367.300    0.122705   1399.621332
    8    7    0    0    0  1    1    0    0    0   1449.448    0.013096   1476.686239
    8    0    0    0    0  2    0    0    0    0   1531.346    0.001854   1553.751146
    9    7    0    0    0  1    1    0    0    0   1541.307    0.003677   1572.137212
    9    8    0    0    0  1    1    0    0    0   1623.226    0.021214   1649.202119
```

9	0	0	0	0	2	0	0	0	0	1712.465	0.043109	1744.653092
10	7	0	0	0	1	1	0	0	0	1731.139	0.038765	1766.623337
10	8	0	0	0	1	1	0	0	0	1807.766	0.062566	1843.688244
10	9	0	0	0	1	1	0	0	0	1901.997	0.045577	1939.139217
11	7	0	0	0	1	1	0	0	0	1928.584	0.055823	1974.528545
12	7	0	0	0	1	1	0	0	0	1982.147	0.002558	2028.848550
11	8	0	0	0	1	1	0	0	0	2013.006	0.003929	2051.593453
7	0	0	0	0	3	0	0	0	0	2040.836	0.000000	2099.431998
12	8	0	0	0	1	1	0	0	0	2058.634	0.076742	2105.913457
10	0	0	0	0	2	0	0	0	0	2080.869	0.619114	2133.625342
11	9	0	0	0	1	1	0	0	0	2097.505	0.438577	2147.044425
8	7	0	0	0	1	2	0	0	0	2125.984	0.000000	2176.496905
12	9	0	0	0	1	1	0	0	0	2149.605	0.000194	2201.364430
13	0	0	0	0	1	0	0	0	0	2183.690	10.938549	2245.577064
8	7	0	0	0	2	1	0	0	0	2210.882	0.000000	2253.561812
9	7	0	0	0	1	2	0	0	0	2217.769	0.000000	2271.947878
14	0	0	0	0	1	0	0	0	0	2259.906	2.714097	2346.681387
11	10	0	0	0	1	1	0	0	0	2290.749	0.196357	2341.530550
8	0	0	0	0	3	0	0	0	0	2295.530	0.000000	2330.626720
9	8	7	0	0	1	1	1	0	0	2302.688	0.000000	2349.012785
12	10	0	0	0	1	1	0	0	0	2344.755	0.611399	2395.850555
9	8	0	0	0	1	2	0	0	0	2387.357	0.000000	2426.077693
9	7	0	0	0	2	1	0	0	0	2391.853	0.000000	2444.463758
10	7	0	0	0	1	2	0	0	0	2409.944	0.000000	2466.434003
9	8	0	0	0	2	1	0	0	0	2476.543	0.000000	2521.528665
11	0	0	0	0	2	0	0	0	0	2483.846	0.138774	2549.435759
10	8	7	0	0	1	1	1	0	0	2489.571	0.000000	2543.498910
12	11	0	0	0	1	1	0	0	0	2544.322	0.044133	2603.755763
9	0	0	0	0	3	0	0	0	0	2563.086	0.000000	2616.979638
10	8	0	0	0	1	2	0	0	0	2568.949	0.000000	2620.563817
12	0	0	0	0	2	0	0	0	0	2581.109	0.146811	2658.075768
10	9	7	0	0	1	1	1	0	0	2583.728	0.000000	2638.949883
11	7	0	0	0	1	2	0	0	0	2603.270	0.000000	2674.339211
12	7	0	0	0	1	2	0	0	0	2661.489	0.000000	2728.659216
10	9	8	0	0	1	1	1	0	0	2663.127	0.000000	2716.014790
11	8	7	0	0	1	1	1	0	0	2690.692	0.000000	2751.404118
12	8	7	0	0	1	1	1	0	0	2740.976	0.000000	2805.724123
10	9	0	0	0	1	2	0	0	0	2754.663	0.000000	2811.465763
10	7	0	0	0	2	1	0	0	0	2764.943	0.000000	2833.436008
11	9	7	0	0	1	1	1	0	0	2775.116	0.000000	2846.855091
11	8	0	0	0	1	2	0	0	0	2777.865	0.000000	2828.469026
12	8	0	0	0	1	2	0	0	0	2820.214	0.000000	2882.789031
12	9	7	0	0	1	1	1	0	0	2831.874	0.000000	2901.175096
10	8	0	0	0	2	1	0	0	0	2839.050	0.000000	2910.500915
11	9	8	0	0	1	1	1	0	0	2862.310	0.000000	2923.919999
13	7	0	0	0	1	1	0	0	0	2870.844	0.091567	2945.387730
12	9	8	0	0	1	1	1	0	0	2911.132	0.000000	2978.240003
10	9	0	0	0	2	1	0	0	0	2935.579	0.000000	3005.951888
11	9	0	0	0	1	2	0	0	0	2944.114	0.000000	3019.370971
13	8	0	0	0	1	1	0	0	0	2945.937	0.003908	3022.452638
14	7	0	0	0	1	1	0	0	0	2947.985	0.019956	3046.492053
11	10	7	0	0	1	1	1	0	0	2970.703	0.000000	3041.341216
12	9	0	0	0	1	2	0	0	0	2999.408	0.000000	3073.690976
15	0	0	0	0	1	0	0	0	0	3001.317	10.961334	3143.589841
14	8	0	0	0	1	1	0	0	0	3023.250	0.043478	3123.556960
12	10	7	0	0	1	1	1	0	0	3029.366	0.000000	3095.661221
13	9	0	0	0	1	1	0	0	0	3046.226	0.313656	3117.903610
11	10	8	0	0	1	1	1	0	0	3052.606	0.000000	3118.406123
12	10	8	0	0	1	1	1	0	0	3103.333	0.000000	3172.726128
10	0	0	0	0	3	0	0	0	0	3105.834	0.000000	3200.438013
14	9	0	0	0	1	1	0	0	0	3114.813	0.009377	3219.007933
11	10	9	0	0	1	1	1	0	0	3139.402	0.000000	3213.857096
11	7	0	0	0	2	1	0	0	0	3159.682	0.000000	3249.246425
12	10	9	0	0	1	1	1	0	0	3196.602	0.000000	3268.177101
12	11	7	0	0	1	1	1	0	0	3224.814	0.000000	3303.566429
13	10	0	0	0	1	1	0	0	0	3231.794	0.054309	3312.389735
11	8	0	0	0	2	1	0	0	0	3249.379	0.000000	3326.311332
12	7	0	0	0	2	1	0	0	0	3266.258	0.000000	3357.886434
14	10	0	0	0	1	1	0	0	0	3288.318	0.311454	3413.494058
12	11	8	0	0	1	1	1	0	0	3306.576	0.000000	3380.631337
11	10	0	0	0	1	2	0	0	0	3324.029	0.000000	3408.343221
11	9	0	0	0	2	1	0	0	0	3326.442	0.000000	3421.762305
12	8	0	0	0	2	1	0	0	0	3340.085	0.000000	3434.951341
12	10	0	0	0	1	2	0	0	0	3383.135	0.000000	3462.663226
12	11	9	0	0	1	1	1	0	0	3390.112	0.000000	3476.082309
12	9	0	0	0	2	1	0	0	0	3430.094	0.000000	3530.402314
13	11	0	0	0	1	1	0	0	0	3431.595	0.064443	3520.294944
13	12	0	0	0	1	1	0	0	0	3473.671	0.274985	3574.614948

14	11	0	0	0	1	1	0	0	0	3504.611	0.016774	3621.399266
11	10	0	0	0	2	1	0	0	0	3525.442	0.000000	3616.248430
14	12	0	0	0	1	1	0	0	0	3544.575	0.216256	3675.719271
13	7	0	0	0	1	2	0	0	0	3551.255	0.000000	3645.198396
12	11	10	0	0	1	1	1	0	0	3591.017	0.000000	3670.568434
14	7	0	0	0	1	2	0	0	0	3629.321	0.000000	3746.302719
13	8	7	0	0	1	1	1	0	0	3629.348	0.000000	3722.263303
12	10	0	0	0	2	1	0	0	0	3632.904	0.000000	3724.888439
15	7	0	0	0	1	1	0	0	0	3690.620	0.057330	3843.400507
13	8	0	0	0	1	2	0	0	0	3707.191	0.000000	3799.328211
14	8	7	0	0	1	1	1	0	0	3707.587	0.000000	3823.367626
11	0	0	0	0	3	0	0	0	0	3710.072	0.000000	3824.153638
13	9	7	0	0	1	1	1	0	0	3729.563	0.000000	3817.714276
15	8	0	0	0	1	1	0	0	0	3767.503	0.003355	3920.465414
12	11	0	0	0	1	2	0	0	0	3782.117	0.000000	3878.473643
14	8	0	0	0	1	2	0	0	0	3785.603	0.000000	3900.432533
14	9	7	0	0	1	1	1	0	0	3799.075	0.000000	3918.818599
13	9	8	0	0	1	1	1	0	0	3807.427	0.000000	3894.779184
12	11	0	0	0	2	1	0	0	0	3830.473	0.000000	3932.793648
12	0	0	0	0	3	0	0	0	0	3855.142	0.000000	3987.113652
15	9	0	0	0	1	1	0	0	0	3856.087	0.296873	4015.916387
14	9	8	0	0	1	1	1	0	0	3877.112	0.000000	3995.883506
13	9	0	0	0	1	2	0	0	0	3905.022	0.000000	3990.230156
13	10	7	0	0	1	1	1	0	0	3917.475	0.000000	4012.200401
14	9	0	0	0	1	2	0	0	0	3965.979	0.000000	4091.334479
14	10	7	0	0	1	1	1	0	0	3974.924	0.000000	4113.304724
13	10	8	0	0	1	1	1	0	0	3990.047	0.000000	4089.265308
15	10	0	0	0	1	1	0	0	0	4045.814	0.064879	4210.402512
14	10	8	0	0	1	1	1	0	0	4047.669	0.000000	4190.369631
13	10	9	0	0	1	1	1	0	0	4092.635	0.000000	4184.716281
13	11	7	0	0	1	1	1	0	0	4113.155	0.000000	4220.105609
14	10	9	0	0	1	1	1	0	0	4141.529	0.000000	4285.820604
13	12	7	0	0	1	1	1	0	0	4159.888	0.000000	4274.425614
14	11	7	0	0	1	1	1	0	0	4187.097	0.000000	4321.209932
13	11	8	0	0	1	1	1	0	0	4193.523	0.000000	4297.170517
15	11	0	0	0	1	1	0	0	0	4230.541	0.569197	4418.307720
14	12	7	0	0	1	1	1	0	0	4231.717	0.000000	4375.529937
13	12	8	0	0	1	1	1	0	0	4232.320	0.000000	4351.490522
14	11	8	0	0	1	1	1	0	0	4267.638	0.000000	4398.274839
13	10	0	0	0	1	2	0	0	0	4269.586	0.000000	4379.202406
15	12	0	0	0	1	1	0	0	0	4275.819	0.054823	4472.627725
13	11	9	0	0	1	1	1	0	0	4286.377	0.000000	4392.621490
14	12	8	0	0	1	1	1	0	0	4304.323	0.000000	4452.594844
14	10	0	0	0	1	2	0	0	0	4306.419	0.000000	4480.306729
13	12	9	0	0	1	1	1	0	0	4331.648	0.000000	4446.941494
13	0	0	0	0	2	0	0	0	0	4337.662	0.031431	4491.154129
14	11	9	0	0	1	1	1	0	0	4351.765	0.000000	4493.725812
15	7	0	0	0	1	2	0	0	0	4373.180	0.000000	4543.211173
14	13	0	0	0	1	1	0	0	0	4381.605	0.261159	4592.258451
14	12	9	0	0	1	1	1	0	0	4394.922	0.000000	4548.045817
15	8	7	0	0	1	1	1	0	0	4453.063	0.000000	4620.276080
13	11	10	0	0	1	1	1	0	0	4477.701	0.000000	4587.107614
14	0	0	0	0	2	0	0	0	0	4481.456	0.186408	4693.362774
13	12	10	0	0	1	1	1	0	0	4524.877	0.000000	4641.427619
14	11	10	0	0	1	1	1	0	0	4531.026	0.000000	4688.211937
15	8	0	0	0	1	2	0	0	0	4532.697	0.000000	4697.340987
15	9	7	0	0	1	1	1	0	0	4541.573	0.000000	4715.727053
14	12	10	0	0	1	1	1	0	0	4576.090	0.000000	4742.531942
15	9	8	0	0	1	1	1	0	0	4621.227	0.000000	4792.791960
13	11	0	0	0	1	2	0	0	0	4669.034	0.000000	4795.012823
15	9	0	0	0	1	2	0	0	0	4707.116	0.000000	4888.242933
13	12	11	0	0	1	1	1	0	0	4722.679	0.000000	4849.332828
15	10	7	0	0	1	1	1	0	0	4733.643	0.000000	4910.213178
14	11	0	0	0	1	2	0	0	0	4738.852	0.000000	4896.117146
13	12	0	0	0	1	2	0	0	0	4752.637	0.000000	4903.652832
14	12	11	0	0	1	1	1	0	0	4790.384	0.000000	4950.437150
15	10	8	0	0	1	1	1	0	0	4808.006	0.000000	4987.278085
14	12	0	0	0	1	2	0	0	0	4818.229	0.000000	5004.757155
15	10	9	0	0	1	1	1	0	0	4898.888	0.000000	5082.729058
15	11	7	0	0	1	1	1	0	0	4914.251	0.000000	5118.118386
15	12	7	0	0	1	1	1	0	0	4964.185	0.000000	5172.438391
15	11	8	0	0	1	1	1	0	0	4996.409	0.000000	5195.183293
13	7	0	0	0	2	1	0	0	0	5024.948	0.000000	5190.964794
15	12	8	0	0	1	1	1	0	0	5038.408	0.000000	5249.503298
14	13	7	0	0	1	1	1	0	0	5069.816	0.000000	5292.069117
15	11	9	0	0	1	1	1	0	0	5077.557	0.000000	5290.634266
15	10	0	0	0	1	2	0	0	0	5079.998	0.000000	5277.215183
13	8	0	0	0	2	1	0	0	0	5095.986	0.000000	5268.029702

15	12	9	0	0	1	1	1	0	0	5126.029	0.000000	5344.954271
14	13	8	0	0	1	1	1	0	0	5141.027	0.000000	5369.134024
14	7	0	0	0	2	1	0	0	0	5170.593	0.000000	5393.173440
15	13	0	0	0	1	1	0	0	0	5184.840	0.041497	5389.166905
13	9	0	0	0	2	1	0	0	0	5204.631	0.000000	5363.480675
14	13	9	0	0	1	1	1	0	0	5240.945	0.000000	5464.584997
14	8	0	0	0	2	1	0	0	0	5241.977	0.000000	5470.238347
15	14	0	0	0	1	1	0	0	0	5262.359	0.043043	5490.271228
15	11	10	0	0	1	1	1	0	0	5273.040	0.000000	5485.120391
15	12	10	0	0	1	1	1	0	0	5323.417	0.000000	5539.440396
14	9	0	0	0	2	1	0	0	0	5333.167	0.000000	5565.689320
13	10	0	0	0	2	1	0	0	0	5388.280	0.000000	5557.966799
14	13	10	0	0	1	1	1	0	0	5412.531	0.000000	5659.071122
15	11	0	0	0	1	2	0	0	0	5449.300	0.000000	5693.025600
14	10	0	0	0	2	1	0	0	0	5492.691	0.000000	5760.175445
15	12	11	0	0	1	1	1	0	0	5506.147	0.000000	5747.345604
15	12	0	0	0	1	2	0	0	0	5539.305	0.000000	5801.665609
13	11	0	0	0	2	1	0	0	0	5586.315	0.000000	5765.872008
13	12	0	0	0	2	1	0	0	0	5621.561	0.000000	5820.192013
14	13	11	0	0	1	1	1	0	0	5627.059	0.000000	5866.976331
14	13	12	0	0	1	1	1	0	0	5660.192	0.000000	5921.296335
14	11	0	0	0	2	1	0	0	0	5723.712	0.000000	5968.080653
14	12	0	0	0	2	1	0	0	0	5754.732	0.000000	6022.400658
15	13	7	0	0	1	1	1	0	0	5874.275	0.000000	6088.977571
15	0	0	0	0	2	0	0	0	0	5880.691	0.500230	6287.179682
15	13	8	0	0	1	1	1	0	0	5947.103	0.000000	6166.042478
15	14	7	0	0	1	1	1	0	0	5952.720	0.000000	6190.081894
15	14	8	0	0	1	1	1	0	0	6025.721	0.000000	6267.146801
15	13	9	0	0	1	1	1	0	0	6044.043	0.000000	6261.493451
15	14	9	0	0	1	1	1	0	0	6113.933	0.000000	6362.597774
15	13	10	0	0	1	1	1	0	0	6231.850	0.000000	6455.979576
15	14	10	0	0	1	1	1	0	0	6289.678	0.000000	6557.083899
15	13	11	0	0	1	1	1	0	0	6414.812	0.000000	6663.884785
15	13	12	0	0	1	1	1	0	0	6453.259	0.000000	6718.204789
13	0	0	0	0	3	0	0	0	0	6461.914	0.000000	6736.731193
14	13	0	0	0	1	2	0	0	0	6473.585	0.000000	6837.835516
15	14	11	0	0	1	1	1	0	0	6489.133	0.000000	6764.989107
15	14	12	0	0	1	1	1	0	0	6525.468	0.000000	6819.309112
14	13	0	0	0	2	1	0	0	0	6541.164	0.000000	6938.939838
15	7	0	0	0	2	1	0	0	0	6572.274	0.000000	6986.990348
15	8	0	0	0	2	1	0	0	0	6646.893	0.000000	7064.055255
14	0	0	0	0	3	0	0	0	0	6664.652	0.000000	7040.044161
15	9	0	0	0	2	1	0	0	0	6732.127	0.000000	7159.506228
15	10	0	0	0	2	1	0	0	0	6924.093	0.000000	7353.992353
15	11	0	0	0	2	1	0	0	0	7091.982	0.000000	7561.897561
15	12	0	0	0	2	1	0	0	0	7133.631	0.000000	7616.217566
15	13	0	0	0	1	2	0	0	0	7338.643	0.000000	7634.743969
15	14	13	0	0	1	1	1	0	0	7383.890	0.000000	7735.848292
15	14	0	0	0	1	2	0	0	0	7485.046	0.000000	7836.952615
15	13	0	0	0	2	1	0	0	0	8064.045	0.000000	8532.756746
15	14	0	0	0	2	1	0	0	0	8142.869	0.000000	8633.861069
15	0	0	0	0	3	0	0	0	0	8638.119	0.000000	9430.769523

```
--------------------------------------------------------------------------------
Dipole moment function written to files dipolex(y,z)
 @CHECKOUT-I, Total execution time :       0.0000 seconds.
--executable xcubic finished with status           0
```
